# Comparative efficacy and acceptability of non-invasive neuromodulation technologies and botulinum toxin injections for post-stroke spasticity and motor function: a network meta-analysis of randomised controlled trials

**DOI:** 10.1016/j.eclinm.2024.103034

**Published:** 2024-12-27

**Authors:** Jiapeng Huang, Chuncha Bao, Yin Chen, Wenyi Zhu, Kexin Zhang, Chunlong Liu, Chunzhi Tang

**Affiliations:** a Clinical Medical College of Acupuncture-Moxibustion and Rehabilitation, Guangzhou University of Chinese Medicine, Guangzhou, Guangdong, China; b Department of Rehabilitation Medicine, West China Hospital of Sichuan University, Chengdu, Sichuan, China; c China Institute of Sport Science, Beijing, China

**Keywords:** Neuromodulation, Botulinum toxin, Post-stroke spasticity, Motor function, Network meta-analysis

## Abstract

**Background:**

Non-invasive neuromodulation is a promising approach for improving spasticity and motor function after stroke. However, it is still unclear which type of non-invasive neuromodulation is effective and evidence of important differences between them and botulinum toxin (BoNT) injection is limited. We aimed to assess the comparative efficacy and acceptability of non-invasive neuromodulation technologies and BoNT for post-stroke spasticity and motor function.

**Methods:**

In this network meta-analysis, Cochrane Library, EMBASE, MEDLINE, Web of Science, Scopus, CNKI, and Wan Fang Data were searched from the earliest records to 8 October 2024. Randomised controlled trials that compared any type of non-invasive neuromodulation therapies, BoNT, and control treatments (including sham or no stimulation/injection) for post-stroke spasticity measured by modified Ashworth scale (MAS) were included. MAS, motor function, and acceptability were pooled using random-effects model with summary weighted mean difference (WMD) or risk ratios (RR) alongside 95% confidence interval (CI). Ranking probabilities of the treatments were estimated. Clinical importance was categorized as definite, probable, possible, or definitely not, considering the relationship between effect measures (95% CI) and minimal clinically important difference (1, 6, and 1.5 points for MAS, motor function, and acceptability, respectively). The quality of evidence was assessed using CINeMA online web. PROSPERO registration CRD42024543494.

**Findings:**

6260 studies were identified and 185 trials (11,185 participants; 12 interventions) were included. Compared with control treatments, BoNT, high- and low-frequency repetitive transcranial magnetic stimulation (HFrTMS and LFrTMS), and anodal, cathodal, and dual transcranial direct current stimulation (atDCS, ctDCS, and dtDCS) significantly improved spasticity at short-term follow-up (WMD range −0.81 to −0.31), but did not achieve clinical importance. At mid-term, ctDCS (WMD = −2.00; 95% CI: −3.03, −0.97) and dtDCS (WMD = −1.62; 95% CI: −3.22, −0.02) were more efficacious than control treatments in reducing post-stroke spasticity with probable clinical importance. For motor function, atDCS, ctDCS, and dtDCS were more efficacious than control treatments (WMD range 6.29–13.00), with probable clinical importance, while BoNT, HFrTMS, and LFrTMS with possible clinical importance (WMD range 3.42–5.28). Various modalities have comparable acceptability to control treatments (RR range 0.48–1.46). Confidence in accordance with CINeMA ranged from high to low. Sensitivity and meta-regression analyses on limb measured, cointervention, and stroke stage confirmed the main findings of this study.

**Interpretation:**

Taken together with clinical importance, evidence available supports three forms of tDCS as effective treatments for post-stroke spasticity and/or motor impairments, whereas BoNT, HFrTMS, and LFrTMS for motor impairments. These modalities could be considered alongside rehabilitation interventions as core treatments for post-stroke spasticity and motor impairments.

**Funding:**

10.13039/501100002858China Postdoctoral Science Foundation (2024M752230).

**Research in context**

**Evidence before this study**

Cochrane Library, EMBASE, MEDLINE, Web of Science, Scopus, CNKI, and Wan Fang Data were searched from inception to 8 October 2024 (full search strategy and terms are included in Appendix 1) for randomised controlled trials (RCTs) that compared any type of non-invasive neuromodulation therapies, botulinum toxin (BoNT), and control treatments for post-stroke spasticity measured by modified Ashworth scale. Previous meta-analyses dispersedly reported the efficacy of non-invasive neuromodulation therapies and BoNT. However, while supportive evidence was found, challenges still need to be addressed in achieving clinically relevant and these reviews reported limited evidence of crucial differences between BoNT, certain types of neuromodulation technologies, and other control treatments (including sham or no stimulation/injection) for post-stroke spasticity and motor function, thus requiring a comprehensive network meta-analysis on this topic.

**Added value of this study**

To our knowledge, this is the first and most comprehensive network meta-analysis to date, investigating the comparative efficacy and acceptability of non-invasive neuromodulation technologies and BoNT in treating post-stroke spasticity and motor impairments. Our findings have added to the current knowledge regarding this issue, supporting anodal, cathodal and dual transcranial direct current stimulation (atDCS, ctDCS, and dtDCS) as effective and acceptable treatments for post-stroke spasticity and/or motor impairments, as well as BoNT and high- and low-frequency transcranial magnetic stimulation for motor impairments, compared with control treatments.

**Implications of all the available evidence**

We suggest policymakers and healthcare providers consider using and promoting tDCS alongside rehabilitation interventions such as exercise therapy and occupational therapy in appropriate patient populations. However, the absence of evidence prevents further analysis, especially the role of atDCS and dtDCS in treating post-stroke spasticity and motor impairment at various follow-up frames. More well-designed trials comprising head-to-head comparisons are needed.

## Introduction

Spasticity is one of the most common complications of stroke, occurring in approximately 39.5% of stroke survivors,^1^ and if left untreated, it may amplify motor dysfunction or hinder a person’s ability to recover and function, which jeopardises stroke survivors’ activities of daily living.^2^^,^^3^ Effective management of spasticity has the potential to enhance motor function throughout the long-term period after stroke,^2^ and thus seeking appropriate and acceptable treatment options for post-stroke spasticity is of clinical significance, especially for decision-making amidst post-stroke rehabilitation.

Post-stroke spasticity is currently managed by non-surgical therapies such as physiotherapy, orthosis, oral medications, neurolysis, and botulinum toxin (BoNT) injection. However, oral medications directly used to relieve post-stroke spasticity do not improve motor function, but in turn, even cause weakness, lethargy, and drowsiness, which might hinder the recovery of function.^4^^,^^5^ Neurolysis may cause side effects such as sensory loss and dysesthesia on the injected extremity.^6^ BoNT injection is recommended as first-line treatment and one of the most effective treatments for spasticity,^7^ but carries the risk of post-injection weakness and the formation of neutralising antibodies following repeated injections,^8^ and uncertainty persists about its contribution to motor functional recovery.^2^

Neuromodulation with electrical or magnetic stimulation is an easy-to-use technique with autonomous control potential, which has become an attractive choice for post-stoke spasticity due to its non-invasive method, acceptability, reversibility, and positive outcomes.^9^, ^10^, ^11^, ^12^ Non-invasive neuromodulation technologies mainly include high-frequency repetitive transcranial magnetic stimulation (HFrTMS), low-frequency repetitive transcranial magnetic stimulation (LFrTMS), continuous theta-burst stimulation (cTBS), intermittent theta-burst stimulation (iTBS), anodal transcranial direct current stimulation (atDCS), cathodal transcranial direct current stimulation (ctDCS), dual transcranial direct current stimulation (dtDCS), neuromuscular electrical stimulation (NMES), transcutaneous electrical nerve stimulation (TENS), repetitive peripheral magnetic stimulation (rPMS), vagus nerve stimulation (VNS), interferential current therapy (ICT), etc. Compared with BoNT injections, these neuromodulation methods are non-invasive and non-drug treatments with no risk of eliciting neuromuscular denervation. However, there is limited evidence to support the use of one type of non-invasive neuromodulation technologies or BoNT over another, as traditional pairwise meta-analysis cannot answer key questions about which therapy works best due to the lack of direct comparison studies, leaving the clinicians and patients to determine which type of treatment to deliver based on preferences. Therefore, comparative evidence on the efficacy and acceptability of BoNT and the aforementioned neuromodulation technologies for post-stroke spasticity and motor function is crucial.

Here, we hypothesise that non-invasive neuromodulation technologies may be comparable to BoNT in reducing spasticity and may better improve motor function, as well as have better acceptability and longer-lasting efficacy. As such, the specific research question for this network meta-analysis of randomised controlled trials (RCTs) was: What are the effects and acceptability of BoNT, HFrTMS, LFrTMS, cTBS, iTBS, atDCS, ctDCS, dtDCS, NMES, TENS, rPMS, VNS, and ICT for post-stroke spasticity and motor function, and which is the optimal option?

## Methods

### Study design

A network meta-analysis of RCTs was conducted and reported according to the guidelines of PRISMA for Network Meta-Analyses.^13^ The study protocol was registered in PROSPERO (CRD42024543494).

### Search strategy and selection criteria

JPH and CCB independently searched the Cochrane Library, EMBASE, MEDLINE, Web of Science, Scopus, CNKI, and Wan Fang Data from the earliest records to 8 October 2024 using the following key terms: “stroke”, “electric stimulation therapy”, “magnetic field therapy”, “botulinum toxins”, “muscle spasticity”, “randomised”, and their expansions combined in algorithms (Appendix 1). Relevant systematic reviews and references in the included studies were thoroughly examined to identify any possible citations.

Inclusion criteria: (1) individuals with post-stroke spasticity; (2) studies should include at least two arms of non-invasive neuromodulation (including HFrTMS, LFrTMS, cTBS, iTBS, atDCS, ctDCS, dtDCS, NMES, TENS, rPMS, VNS, and ICT), BoNT, and control (including sham stimulation/injection and no stimulation/injection); (3) studies comparing interventions of interest plus other therapies with other therapies alone were acceptable and were deemed as interventions of interest vs. control; (4) studies have to evaluate spasticity on hemiplegic limbs measured by the modified Ashworth scale (MAS); (5) RCTs, randomised crossover trials, and quasi-RCTs. Definitions for the conditions and interventions were detailed in Appendix 2.

Exclusion criteria: (1) individuals with aetiologies other than stroke or mixed other aetiologies were excluded if data of patients without stroke were reported together; (2) studies using combinational therapy (such as BoNT plus HFrTMS) and studies in which neuromodulation was delivered through acupuncture points; (3) individuals received only a single session of neuromodulation or studies only comparing the same type of intervention of interest; (4) review, editorial, and conference abstract, unpublished, or non-peer-reviewed reports, and non-English/Chinese publications; (5) second analysis of the included studies.

### Procedures

JPH and CCB screened the titles and abstracts of the retrieved results to exclude irrelevant studies, followed by a full-text retrieval of the remaining studies. YC, WYZ, and KXZ scrutinised the articles for final inclusions. Discrepancies and uncertainties were discussed and settled by consulting CLL.

JPH, CCB, and YC independently extracted data from included studies. Data on study characteristics, population characteristics, intervention characteristics, efficacy endpoints, follow-up, adverse events, and all-cause discontinuation were extracted. When quantitative data were only available in figures, they were extracted as numerical data using Engauge Digitizer.^14^ Missing data items were not requested from authors as the data has not been peer-reviewed. Discrepancies and uncertainties were resolved by consulting CZT.

### Outcomes

Our primary outcome of interest was spasticity on hemiplegic limbs measured by MAS. Secondary outcomes included motor function assessed by Fugl-Meyer Assessment (FMA) and acceptability (treatment discontinuation measured by the proportion of patients who withdrew for any reason during the treatment period). All-cause discontinuation was used as an assessment for the acceptability of treatments, as it contains efficacy and tolerability.^15^ If reported, data were extracted preferentially from intention-to-treat results or study-reported modified results, or per-protocol results with or without an imputation were extracted.^16^

### Statistics

YC, WYZ, and KXZ separately performed a risk of bias assessment of included studies using the Cochrane Collaboration’s tool for evaluating risk of bias (RoB 2.0).^17^ Each study included was classified as high-bias risk, low-bias risk, or some concerns. Any discordances and uncertainties were resolved by the judgment of CLL.

To improve comparability, MAS, motor function, and acceptability were analysed in short- and mid-terms, which were defined as 0–3 weeks and 4–6 weeks post-treatment, respectively. In the case of outcomes within the same follow-up frame, the timepoint closest to the 3 weeks or 6 weeks was chosen. Pairwise and network meta-analyses were used to estimate effect sizes for each outcome. Outcome change from baseline was adopted. We estimated summary risk ratio (RR; Mantel-Haenszel method) alongside 95% confidence interval (CI) for dichotomous outcomes, and a standard mean difference (SMD; Hedges’g) alongside 95% CI was calculated if the methods of outcome measurement were different among studies included; otherwise, we estimated a weighted mean difference (WMD) alongside 95% CI.^18^ For multi-arm studies, we merged similar intervention arms into a single group for analysis, to minimise the number of comparisons.

Pairwise meta-analyses for direct comparisons were conducted utilising a random-effects model according to the method of DerSimonian-Laird method with Hartung-Knapp-Sidik-Jonkman variance correction.^19^^,^^20^ To determine the heterogeneity of direct comparisons, the Cochrane Q statistics and I^2^ were adopted, in which I^2^ more than 50% was deemed as significant heterogeneity.

Network meta-analyses were conducted to estimate the comparative efficacy of the therapies on spasticity and motor function, as well as their acceptability, with a frequentist framework utilising a random-effects (restricted maximum likelihood estimation) meta-analysis model via the “mvmeta” and “network” packages in the statistical software package Stata.^21^^,^^22^ Network geometry graphs were drawn to explore the comparative relationship among the available evidence.^23^ To identify the superiority of each intervention over each other intervention, mean rankings and surface under the cumulative ranking curve (SUCRA) were estimated.^24^

The clinical importance of each outcome at each follow-up was considered as a difference in spasticity of 1 point, a difference in motor function of 6 points, and a difference in acceptability of 1.5 points.^25^, ^26^, ^27^, ^28^, ^29^ Subsequently, the clinical importance was interpreted according to the method proposed by Man-Son-Hing et al.,^30^ considering effect measures with 95% CI.^31^ Briefly, the clinical importance of the results was defined into four categories from high to low: definite, probable, possible, and definitely not, which was detailed in Appendix 3.

We assessed whether the synthesis of the direct comparisons of interventions was performed in comparable baseline clinical characteristics (age, sex proportion, ischemic stroke proportion, time since stroke, and baseline MAS value), considering intervention node, pairwise comparisons, and each network analysis (each outcome at each follow-up).^32^ This evaluation of network transitivity and taking theoretical mechanisms of potential effect modifiers into account led us to explore the effect of baseline MAS value in sensitivity analyses.

To evaluate the statistical heterogeneity in the entire network, τ^2^ across all treatment comparisons was calculated by using network meta-analysis models, explaining correlations caused by multi-arm studies.^33^, ^34^, ^35^ Global and local inconsistencies were assessed by using the design-by-treatment interaction model (global χ^2^ test) and node splitting method for each pairwise comparison, respectively.^36^, ^37^, ^38^ When significant global inconsistency was identified, several strategies were employed, such as checking data, splitting nodes to possibly eliminate sources of the issue, and inspecting the influence of effect modifiers using the meta-regression analysis.^32^

Sensitivity analyses were performed to estimate whether the inclusion of studies with lower extremities measured and cointervention, as well as imbalance baseline clinical characteristics impacted the results. Meta-regression analyses were performed to estimate whether limb measured (upper or lower), cointervention (with or without), and stroke stage (acute, subacute, or chronic) impacted the finding of each outcome. A regression model with a normal likelihood distribution and identity link was conducted for continuous outcomes, while a binomial likelihood and a logistic link were used for dichotomous outcomes.

Publication bias was assessed for small study effects using comparisons-adjusted funnel plots and Egger’s test in case there were more than ten trials in a particular outcome, otherwise, we did not analyse the publication bias.^39^

Statistics were conducted using Stata 13.1 (StataCorp LP, College Station, TX). *p* < 0.05 was deemed as statistically significant.

JPH and CLL independently assessed the quality of the evidence in findings from the primary network meta-analyses utilising the Confidence in Network Meta-Analysis (CINeMA) online platform.^40^ Imprecision, heterogeneity, and incoherence were evaluated concerning the clinical relevance (Appendix 4).^32^ The confidence rating of each comparison was graded as very low-, low-, moderate-, or high-quality evidence in accordance with the standard Grading of Recommendations Assessment, Development and Evaluation (GRADE) assessment. Discrepancies were resolved through consultation with CZT.

### Ethics

No ethical approval was required as this study used publicly available data.

### Role of the funding source

The funders of the study had no role in the study design, data collection, data analysis, data interpretation, or writing of the study.

## Results

### Study selection

**Fig. 1:**
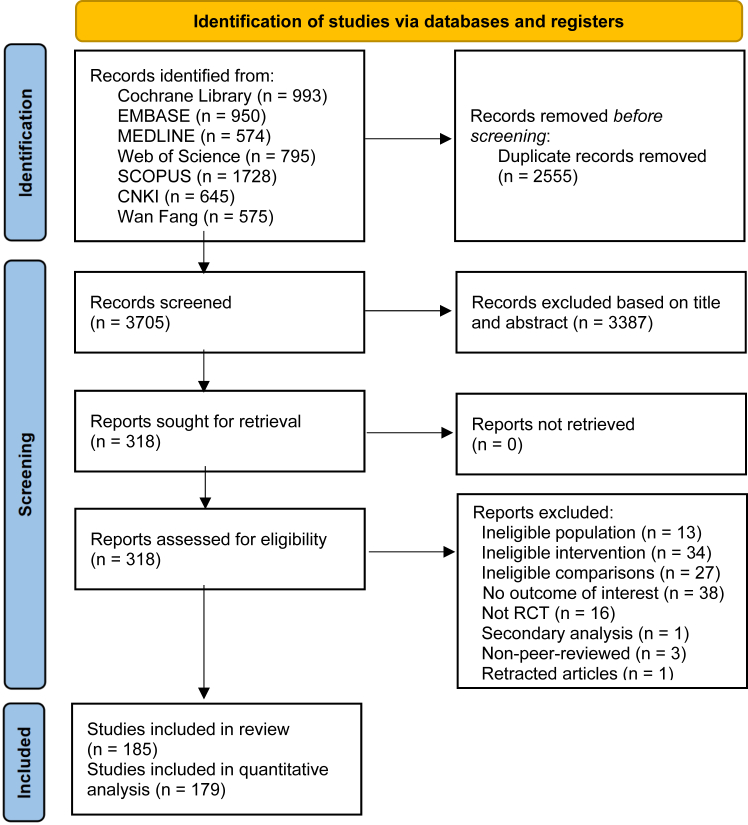
Flow diagram for search strategy and study selection.

A primary retrieval of the databases yielded 6260 related records (Fig. 1). After removing 2555 duplicates, screening of title and abstract resulted in 3387 irrelevant studies. The remaining 318 articles were full-text scrutinised, of which 185 studies met the inclusion criteria (Appendix 5 and 6).

### General characteristics of included studies

A total of 11,185 participants were enrolled in 185 trials (385 arms, 12 interventions) published between 1998 and 2024 (Table 1 and Appendix 7). The sample size per arm ranged from 5 to 170 participants, with a median of 22 participants, whereas the arms’ mean age was 58.36 (6.45) years. The median percentage of males was 60.00 (IQR 55.00 to 68.50) and the median percentage of ischemic stroke was 70.00 (IQR 57.00 to 92.75). The median of mean time since stroke was 2.80 months (IQR 1.55 to 9.39) and the baseline mean spasticity was 2.32 (0.97) on the 0-to-4 scale. Twelve types of intervention were observed, including Control, BoNT, HFrTMS, LFrTMS, cTBS, iTBS, atDCS, ctDCS, dtDCS, NMES, TENS, and rPMS. Variations in the dosage or parameters of therapies were observed in the included studies. Of the included studies, 67.03% focused on upper limb, 23.78% concentrated on lower limb, and the remaining studies paid attention to both upper and lower limbs simultaneously. Most arms (94.29%) included cointervention such as rehabilitation, medication, and usual care.

**Table 1:** General characteristics of the included studies.

Characteristic	Trials (n = 185); Arms (n = 385)
Year of publication, n (%)^a^	
1990–1999	3 (1.62)
2000–2009	25 (13.51)
2010–2019	92 (49.73)
2020–2024	65 (35.14)
Total participants at baseline	
Pooled	11,185
Per arm, median (IQR; range)	22 (16–40; 5 to 170)
Age of participants (y), mean (SD)	58.36 (6.45)
Male participants (%), median (IQR; range)	60.00 (55.00–68.50; 13.00 to 91.00)
Participants with ischemic stroke (%), median (IQR; range)	70.00 (57.00–92.75; 0.00 to 100.00)
Time since stroke (m), median (IQR; range)	2.80 (1.55–9.39; 0.12 to 84.90)
Baseline mean spasticity measured by MAS, mean (SD)	2.32 (0.97)
Interventions, n (%)^b^	
Control	180 (46.75)
BoNT	76 (19.74)
HFrTMS	14 (3.64)
LFrTMS	37 (9.61)
cTBS	1 (0.26)
iTBS	7 (1.82)
atDCS	10 (2.60)
ctDCS	9 (2.34)
dtDCS	6 (1.56)
NMES	34 (8.83)
TENS	8 (2.08)
rPMS	3 (0.78)
Paired with cointervention, n (%)^b^	
Yes	363 (94.29)
No	22 (5.71)
Limb measured, n (%)^a^	
Upper	124 (67.03)
Lower	44 (23.78)
Mixed	17 (9.19)

BoNT = botulinum toxin; HFrTMS = high-frequency transcranial magnetic stimulation; LFrTMS = low-frequency transcranial magnetic stimulation; cTBS = continuous theta-burst stimulation; iTBS = intermittent theta-burst stimulation; atDCS = anodal transcranial direct current stimulation; ctDCS = cathodal transcranial direct current stimulation; dtDCS = dual transcranial direct current stimulation; IQR = interquartile rage; MAS = modified Ashworth scale; NMES = neuromuscular electrical stimulation; rPMS = repetitive peripheral magnetic stimulation; SD = standard deviation; TENS = transcutaneous electrical nerve stimulation.

a Out of 185 trials.

b Out of 385 arms.

### Risk of bias assessment

Of the included trials, 160 were graded as having some concerns, whereas the other 25 were rated as low risk of bias (Appendix 8). We judged 69.19% of trials to have some risk of bias for the randomisation process, 16.76% of trials to have some risk of bias for the deviations from intend interventions, 8.65% of trials to have some risk of bias due to more than 5% of participants being lost after randomisation, 56.22% of trials to have some risk of bias because of insufficient information about the blinding of the outcome assessor, and 78.92% of trials to have some risk of bias for selection of the reported results.

### Assessment of network transitivity

We found no evidence of violating the transitivity assumption when evaluating in terms of mean age, sex proportion, ischemic stroke proportion, and time since stroke. No serious violations were observed when exploring based on the baseline MAS value, but concerns were raised regarding the higher baseline MAS value at short-term follow-up in BoNT vs. Control compared with HFrTMS vs. Control, LFrTMS vs. Control, NMES vs. Control, and rPMS vs. Control, which was the reason for conducting a sensitivity analysis by excluding trials with BoNT vs. Control (Appendix 9).

### Effects on spasticity

Post-stroke spasticity measured by MAS was investigated in 152 trials comprising 8939 participants at short-term follow-up and 58 trials involving 3517 participants at mid-term follow-up.

**Fig. 2:**
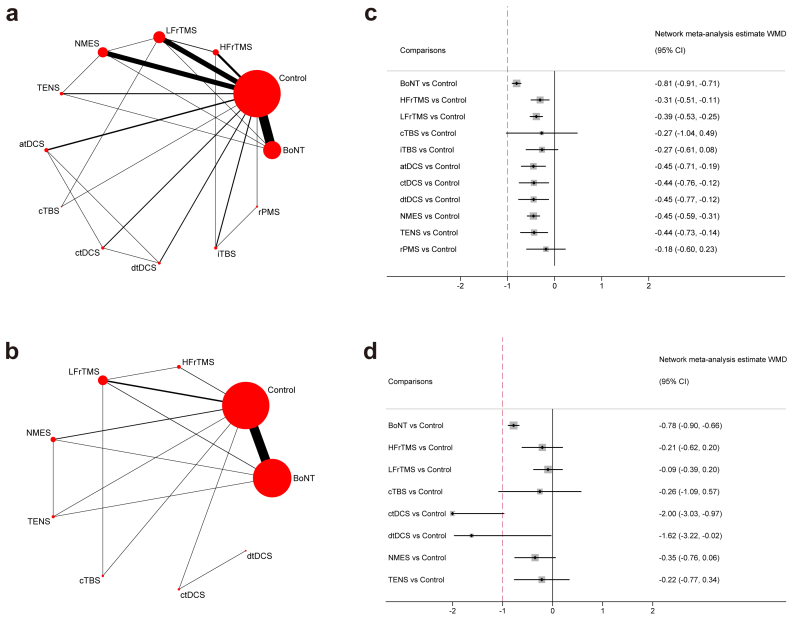
Network graphs and summary network meta-analysis results for each intervention of interest compared with control treatments for post-stroke spasticity. a. Network plots of eligible direct comparisons for post-stroke spasticity at short-term follow-up. b. Network plots of eligible direct comparisons for post-stroke spasticity at mid-term follow-up. The size of the nodes is proportional to the number of studies included in each intervention, and the line width corresponds to studies directly comparing the two interventions. c. Summary network meta-analysis results for each intervention of interest compared with control treatments for post-stroke spasticity at short-term follow-up. d. Summary network meta-analysis results for each intervention of interest compared with control treatments for post-stroke spasticity at mid-term follow-up. Effects are expressed as weighted mean difference (WMD) and 95% CI. The hashed line indicates clinically important difference. Abbreviations: BoNT = botulinum toxin; HFrTMS = high-frequency transcranial magnetic stimulation; LFrTMS = low-frequency transcranial magnetic stimulation; cTBS = continuous theta-burst stimulation; iTBS = intermittent theta-burst stimulation; atDCS = anodal transcranial direct current stimulation; ctDCS = cathodal transcranial direct current stimulation; dtDCS = dual transcranial direct current stimulation; NMES = neuromuscular electrical stimulation; TENS = transcutaneous electrical nerve stimulation; rPMS = repetitive peripheral magnetic stimulation.

Short-term: Pairwise meta-analysis indicated that BoNT, HFrTMS, LFrTMS, atDCS, ctDCS, dtDCS, NMES, TENS, and rPMS were more efficacious than Control (WMD range −0.80 to −0.18). Moreover, BoNT was associated with higher efficacy than LFrTMS (WMD = −1.00; 95% CI, −1.25, −0.75) and TENS (WMD = −0.43; 95% CI, −0.75, −0.11), whereas there was no significant difference between other active interventions (Appendix 10 and 11). The network meta-analysis of spasticity at short-term follow-up provided data on 23 direct comparisons and 43 indirect comparisons between 12 various treatment nodes (Fig. 2). Loop-specific coherence for 14 of 15 loops and local consistency for 22 of 23 direct comparisons were found (Appendix 12 and 13). However, no global inconsistency was found (Appendix 14). All interventions were associated with higher efficacy than Control (BoNT: WMD = −0.81, 95% CI -0.91 to −0.71; HFrTMS: −0.31, −0.51 to −0.11; LFrTMS: −0.39, −0.53 to −0.25; atDCS: −0.45, −0.71 to −0.19; ctDCS: −0.44, −0.76 to −0.12; dtDCS: −0.45, −0.77 to −0.12; NMES: −0.45, −0.59, −0.31; TENS: −0.44, −0.73 to −0.14)), except for cTBS (−0.27, −1.04 to 0.49), iTBS (−0.27, −0.61 to 0.08), and rPMS (−0.18, −0.60 to 0.23), but no clinical importance was observed (Fig. 2). BoNT demonstrated a significantly higher improvement than HFrTMS, LFrTMS, iTBS, atDCS, ctDCS, dtDCS, NMES, TENS, and rPMS (WMD range −0.62 to −0.36). No other statistical differences were observed between active treatments (Appendix 15). SURCA ranked BoNT (98.7%), then NMES (64.3%), atDCS (62.5%), dtDCS (60.8%), ctDCS (59.6%), TENS (59.0%), LFrTMS (51.6%), and HFrTMS (37.3%) for spasticity at short-term follow-up (Appendix 16).

Mid-term: In the pairwise meta-analysis, BoNT, HFrTMS, LFrTMS, ctDCS, and NMES were significantly superior to Control (WMD range −2.00 to −0.25), whereas BoNT was related to higher efficacy than LFrTMS (WMD = −1.06; 95% CI, −1.43, −0.69). No significant difference was found between other active interventions (Appendix 10 and 11). The network meta-analysis of spasticity at 4–6 weeks follow-up provided data on 14 direct comparisons and 22 indirect comparisons between nine various treatment nodes (Fig. 2). Loop-specific coherence for six of seven loops and local consistency for 13 of 14 direct comparisons were found (Appendix 12 and 13). Under global consistency (Appendix 14), we found BoNT (WMD = −0.78; 95% CI, −0.90, −0.66), ctDCS (WMD = −2.00; 95% CI, −3.03, −0.97), and dtDCS (WMD = −1.62; 95% CI, −3.22, −0.02) significantly reduced spasticity compared with control treatments, of which ctDCS and dtDCS with probable clinical importance (Fig. 2). In the comparisons between two active therapies, ctDCS was associated with higher efficacy than others except dtDCS with probable clinical importance (MD range −1.91 to −1.22), whereas BoNT was better than HFrTMS, LFrTMS, NMES, and TENS (MD range −0.69 to −0.43). No evidence was observed to suggest any other significant differences between active therapies (Appendix 15). SURCA ranked ctDCS (96.4%), then dtDCS (85.0%) and BoNT (75.1%) for spasticity at mid-term follow-up (Appendix 16).

### Effects on motor function

Motor function measured by FMA was investigated in 114 trials comprising 7003 participants at short-term follow-up and 37 trials involving 1979 participants at mid-term follow-up.

**Fig. 3:**
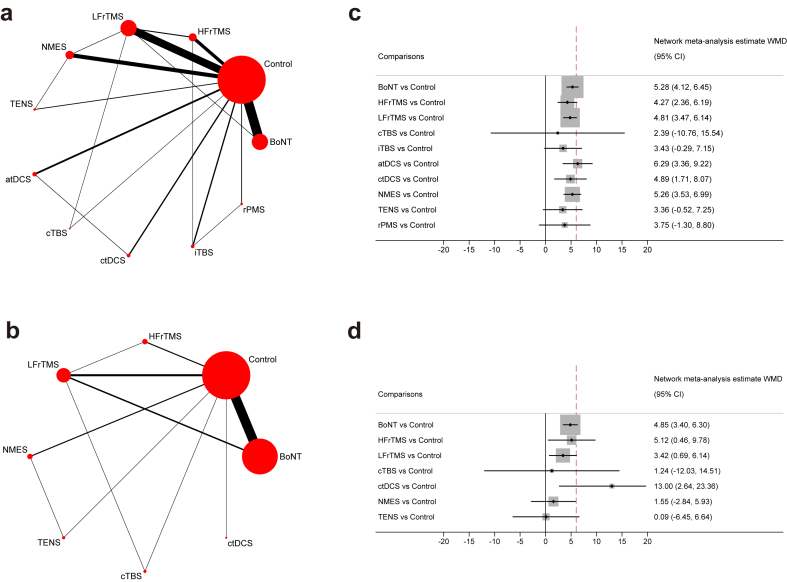
Network graphs and summary network meta-analysis results for each intervention of interest compared with control treatments for motor function. a. Network plots of eligible direct comparisons for motor function at short-term follow-up. b. Network plots of eligible direct comparisons for motor function at mid-term follow-up. The size of the nodes is proportional to the number of studies included in each intervention, and the line width corresponds to studies directly comparing the two interventions. c. Summary network meta-analysis results for each intervention of interest compared with control treatments for motor function at short-term follow-up. d. Summary network meta-analysis results for each intervention of interest compared with control treatments for motor function at mid-term follow-up. Effects are expressed as weighted mean difference (WMD) and 95% CI. The hashed line indicates clinically important difference. Abbreviations: BoNT = botulinum toxin; HFrTMS = high-frequency transcranial magnetic stimulation; LFrTMS = low-frequency transcranial magnetic stimulation; cTBS = continuous theta-burst stimulation; iTBS = intermittent theta-burst stimulation; atDCS = anodal transcranial direct current stimulation; ctDCS = cathodal transcranial direct current stimulation; NMES = neuromuscular electrical stimulation; TENS = transcutaneous electrical nerve stimulation; rPMS = repetitive peripheral magnetic stimulation.

Short-term: In the pairwise meta-analysis, BoNT, HFrTMS, LFrTMS, iTBS, atDCS, ctDCS, dtDCS, NMES, and TENS were found to significantly improve motor function compared with control treatments (MD range 2.41–9.77), whereas there was no difference between active treatments (Appendix 10 and 11). The network meta-analysis of motor function at 0–3 weeks period provided data on 18 direct comparisons and 37 indirect comparisons between 11 various treatment nodes (Fig. 3). Loop-specific coherence for seven of eight loops and local consistency for 17 of 18 direct comparisons were found (Appendix 12 and 13). Under adjusted global consistency (Appendix 14), results showed that BoNT (WMD = 5.28; 95% CI, 4.12, 6.45), HFrTMS (WMD = 4.27; 95% CI, 2.36, 6.19), LFrTMS (WMD = 4.81; 95% CI, 3.47, 6.14), atDCS (WMD = 6.29; 95% CI, 3.66, 9.22), ctDCS (WMD = 4.89; 95% CI, 1.71, 8.07), and NMES (WMD = 5.26; 95% CI, 3.53, 6.99) were associated with higher efficacious than control treatments, with clinical importance ranging from probable (atDCS) to possible (Fig. 3). No evidence was observed to suggest any significant difference between active therapies (Appendix 15). SURCA ranked atDCS (81.4%), then BoNT (69.0%), NMES (67.8%), ctDCS (60.0%), LFrTMS (57.9%), and HFrTMS (46.8%) for motor function at short-term follow-up (Appendix 16).

Mid-term: Pairwise meta-analysis showed that BoNT, HFrTMS, LFrTMS, and ctDCS were significantly superior to Control (MD range 3.46–13), whereas there was no statistical difference between other active interventions (Appendix 10 and 11). The network meta-analysis of motor function at 4–6 weeks follow-up provided data on 11 direct comparisons and 17 indirect comparisons between eight various treatment nodes (Fig. 3). Under loop-specific coherence and global and local consistencies (Appendix 12–14), BoNT (WMD = 4.85; 95% CI, 3.40, 6.30), HFrTMS (WMD = 5.12; 95% CI, 0.46, 9.78), LFrTMS (WMD = 3.42; 95% CI, 0.69, 6.14), and ctDCS (WMD = 13.00; 95% CI, 2.64, 23.36) were found to significantly improve motor function compared with control treatments, with clinical importance ranging from probable (ctDCS) to possible (Fig. 3). ctDCS was associated with higher efficacy than NMES (WMD = 11.45; 95% CI, 0.21, 22.70) and TENS (WMD = 12.91; 95% CI, 0.66, 25.16) with possible clinical importance, while no other evidence suggested any significant difference between active treatments (Appendix 15). SURCA ranked ctDCS (95.0%), then BoNT (69.9%), HFrTMS (68.7%), and LFrTMS (52.4%) for motor function at mid-term follow-up (Appendix 16).

### Acceptability

**Fig. 4:**
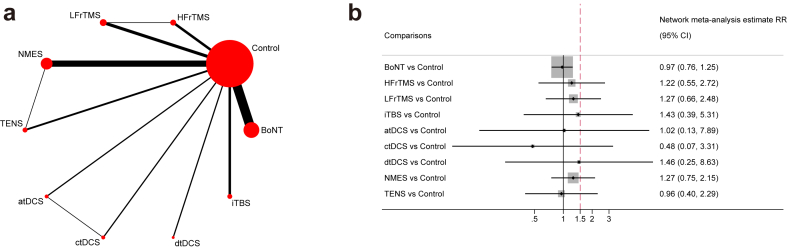
Network graphs and summary network meta-analysis results for each intervention of interest compared with control treatments for acceptability. a. Network plots of eligible direct comparisons for acceptability. The size of the nodes is proportional to the number of studies included in each intervention, and the line width corresponds to studies directly comparing the two interventions. b. Summary network meta-analysis results for each intervention of interest compared with control treatments for acceptability. Effects are expressed as risk ratio (RR) and 95% CI. The hashed line indicates clinically important difference. Abbreviations: BoNT = botulinum toxin; HFrTMS = high-frequency transcranial magnetic stimulation; LFrTMS = low-frequency transcranial magnetic stimulation; iTBS = intermittent theta-burst stimulation; atDCS = anodal transcranial direct current stimulation; ctDCS = cathodal transcranial direct current stimulation; dtDCS = dual transcranial direct current stimulation; NMES = neuromuscular electrical stimulation; TENS = transcutaneous electrical nerve stimulation.

All-cause discontinuation during treatment was reported in 44 trials comprising 2679 participants. No evidence was found to suggest any significant differences between BoNT, neuromodulation and control treatments in the pairwise meta-analysis (RR range 0.53–1.63) (Appendix 10 and 11). The network meta-analysis of acceptability provided data on 12 direct comparisons and 33 indirect comparisons between ten various treatment nodes (Fig. 4). No loop-specific incoherence and global and local inconsistencies were found (Appendix 12–14). Similar to pairwise meta-analysis, the results of network meta-analysis suggested no significant difference between each pair of two treatments (RR range 0.48–1.43) (Fig. 4 and Appendix 15). SURCA ranked ctDCS (79.0%), then BoNT (61.5%) and Control (58.3%) (Appendix 16).

### Sensitivity analyses and meta-regression

Overall, almost all the results persisted in any sensitivity analyses, and minimal observed change in heterogeneity or incoherence for any sensitivity analyses was observed (Appendix 17). For the univariant and multivariant meta-regression analyses, no significant findings were made in terms of limb measured, with or without cointervention, and stroke stage (Appendix 18).

### Publication bias

Publication bias was not observed with comparisons-adjusted funnel plots and Egger’s test for all outcomes at each follow-up (Appendix 19).

### Certainty of the network meta-analysis evidence

Overall, low-to high-quality evidence was found (Appendix 20). In the analysis of spasticity at short-term follow-up (n = 66 comparisons: 1 for high-quality, 63 for moderate-quality, 2 for low-quality), the evidence was mainly downgraded with major concerns for heterogeneity (n = 2 comparisons) and with some concerns for within-study bias (n = 2 comparisons), heterogeneity (n = 29 comparisons), reporting bias (n = 20 comparisons), imprecision (n = 2 comparisons), and incoherence (n = 1 comparison). In the analysis of spasticity at mid-term follow-up (n = 36 comparisons: 4 for high-quality, 31 for moderate-quality, 1 for low-quality), the evidence was mainly downgraded with major concerns for heterogeneity (n = 1 comparison) and with some concerns for within-study bias (n = 30 comparisons), heterogeneity (n = 15 comparisons), reporting bias (n = 14 comparisons), imprecision (n = 10 comparisons), and incoherence (n = 2 comparisons). For motor function at short-term follow-up (n = 55 comparisons: 33 for moderate-quality, 22 for low-quality), the evidence was mainly downgraded with major concerns for heterogeneity (n = 11 comparisons), reporting bias (n = 1 comparison), and imprecision (n = 10 comparisons), and with some concerns for within-study bias (n = 50 comparisons), heterogeneity (n = 28 comparisons), reporting bias (n = 9 comparisons), imprecision (n = 13 comparisons), and incoherence (n = 1 comparison). For motor function at mid-term follow-up (n = 28 comparisons: 20 for moderate-quality, 8 for low-quality), the evidence was mainly downgraded with major concerns for heterogeneity (n = 1 comparison) and imprecision (n = 7 comparisons), and with some concerns for within-study bias (n = 23 comparisons), heterogeneity (n = 14 comparisons), reporting bias (n = 9 comparisons), and imprecision (n = 15 comparisons). In the analysis of acceptability (n = 45 comparisons: 1 for high-quality, 2 for moderate-quality, 42 for low-quality), the evidence was mainly downgraded with major concerns for reporting bias (n = 1 comparison) and imprecision (n = 42 comparisons), and with some concerns for within-study bias (n = 44 comparisons), reporting bias (n = 11 comparisons), and imprecision (n = 2 comparisons).

## Discussion

**Fig. 5:**
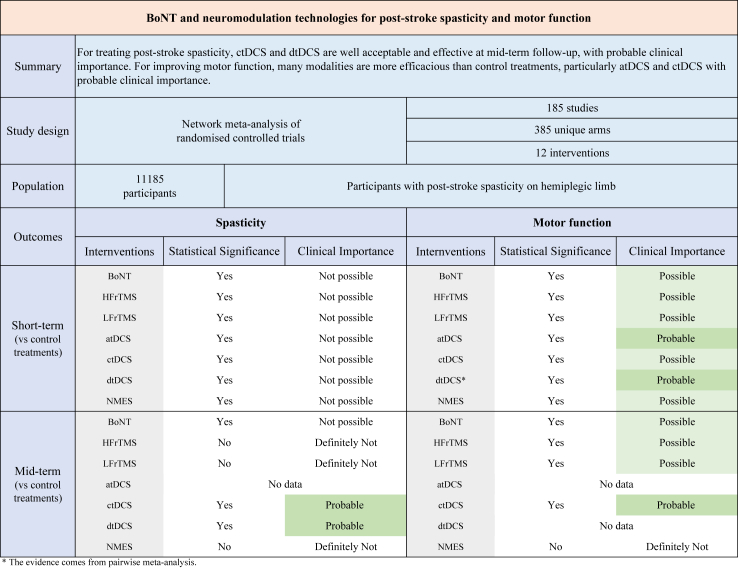
Visual summary of findings. Abbreviations: BoNT = botulinum toxin; HFrTMS = high-frequency transcranial magnetic stimulation; LFrTMS = low-frequency transcranial magnetic stimulation; atDCS = anodal transcranial direct current stimulation; ctDCS = cathodal transcranial direct current stimulation; dtDCS = dual transcranial direct current stimulation; NMES = neuromuscular electrical stimulation.

It is believed that this is the first and most comprehensive network meta-analysis in terms of comparative efficacy and acceptability of BoNT, ten electrical and magnetic neuromodulation technologies, and control treatments for spasticity and motor function in 11,185 participants with post-stroke spasticity. Overall, compared with control treatments, BoNT, HFrTMS, LFrTMS, atDCS, ctDCS, dtDCS, and NMES significantly improved spasticity at short-term follow-up without achieving clinical importance, and ctDCS and dtDCS were more efficacious than control treatments in reducing post-stroke spasticity with probable clinical importance at mid-term follow-up. For motor function, atDCS, ctDCS, and dtDCS were more efficacious than control treatments with probable clinical importance, while BoNT, HFrTMS, and LFrTMS with possible clinical importance (Fig. 5). Various modalities have comparable acceptability to control treatments. Certainty of evidence ranged from high to low. Sensitivity and meta-regression analyses on limb measured, cointervention, and stroke stage appeared to confirm the main findings of this study. These modalities could be considered alongside rehabilitation interventions as core treatments for post-stroke spasticity and motor impairments.

Numerous systematic reviews have evaluated BoNT and non-invasive neuromodulation therapies for post-stroke spasticity and motor function, including Cochrane Reviews^41^ and other systematic reviews outside the Cochrane Library.^9^^,^^12^^,^^42^, ^43^, ^44^, ^45^, ^46^, ^47^, ^48^, ^49^, ^50^, ^51^, ^52^, ^53^ These reviews have included five to 38 trials on treatments for spasticity and motor dysfunction in survivors with stroke, of which a small proportion of the trials are enrolled here, with more concentrated review questions and criteria for study selection, and methodological differences. Overall, the beneficial effect of BoNT, rTMS, tDCS, and NMES in improving post-stroke spasticity and motor function compared with control treatments aligns with findings from the most latest reviews, as well as our previous one.^12^ However, despite this progress, challenges still need to be addressed in achieving clinically relevant and these reviews reported limited evidence of crucial differences between BoNT, certain types of neuromodulation technologies, and other control treatments.

This study provided some different findings compared with other previous studies. Unlike the previous reviews,^9^^,^^49^ the present findings did not favour BoNT and LFrTMS as being associated with reduced spasticity, as they failed to achieve clinical significance. Apart from that, this study supported the use of ctDCS and dtDCS for post-stroke spasticity at mid-term follow-up, with probable clinical importance. The difference in findings could be attributed to the variations in methods and specific criteria for inclusion between the studies, with this study focused on comparative efficacy between interventions of interest by the use of a network meta-analysis model, which incorporates both direct and indirect evidence and makes it possible to include trials comparing various sub-types of tDCS. Additionally, the search was updated to enrol more recent evidence up to October 2024, while the previous reviews were dated to December 2021 and only included three trials for ctDCS and none for dtDCS. Sparse studies and 95% CI close to zero indicate that the findings are prone to change if more trials are included. Notably, the findings of previous reviews in ctDCS and dtDCS reveal trends in reducing spasticity, although they did not reach statistical significance.^12^ Study follow-up may also affect the treatment effects, as the present study indicated that ctDCS and dtDCS at mid-term follow-up were compatible with clinical importance, while the previous reviews solely focused on immediate effect. A most recent meta-analysis also supports the use of ctDCS in reducing post-stroke spasticity.^54^

It is noted that BoNT and non-invasive neuromodulation technologies were not consistently effective across time points. Considering clinical importance, this study found that BoNT, HFrTMS, LFrTMS, ctDCS, dtDCS, and NMES were not associated with clinically improved post-stroke spasticity at short-term follow-up, whereas ctDCS and dtDCS were more efficacious than control treatments with probable clinical importance at mid-term follow-up. One explanation might be the spontaneous recovery and the synergistic effects of cointervention, which could mask the efficacy of the above interventions at short-term follow-up due to the ceiling effect, making it unclear whether there is no efficacy at all or a “true efficacy” is too small to be detected.^55^ It should also be noted that trials comparing interventions of interest plus other therapies with other therapies alone were acceptable and were deemed as interventions of interest vs. control in this study and trials directly comparing interventions of interest with other therapies were excluded, to improve comparability, and therefore most arms (94.29%) included cointervention such as rehabilitation, medication, and usual care. However, sensitivity and meta-regression analyses on cointervention confirmed the main findings of this study. Hence, these interventions should be considered as adjunctive or synergistic therapies to rehabilitation, medication, or usual care. Another explanation might be the dynamic mechanisms of post-stoke rehabilitation, including repair of injured brain network, activation of compensatory areas in the affected hemisphere, inhibition of over-activated unaffected hemisphere, or activation of previously functionally inactive pathways. As a form of non-invasive brain stimulation, tDCS has the potential to inhibit the over-activated unaffected hemisphere through cathodal stimulation, activate the damaged hemisphere through anodal stimulation, or activate and inhibit bilateral brain regions simultaneously through dual stimulation, thereby achieving longer-lasting therapeutic effects. Most notably, tDCS is an intervention that is well acceptable and low-cost, and it may benefit from the possibility of self-administration at home when the patient is discharged. However, due to a lack of evidence available, the mid-term effects of atDCS and dtDCS need further well-designed trials to be determined. If the observed spasticity and motor function align with the survivors’ goals, it may be appropriate to recommend these treatments if they are available.

In terms of motor impairment after stroke, BoNT, HFrTMS, LFrTMS, atDCS, ctDCS, and NMES were associated with beneficial effects compared with control treatments, which were also found in the latest systematic reviews. Varvarousis and colleagues (2021) conducted a meta-analysis of BoNT for motor impairment in survivors with post-stroke spasticity, and their findings favour the use of BoNT for improving motor function.^49^ Li and colleagues (2024) published a meta-analysis of non-invasive brain stimulation, in which HFrTMS, LFrTMS, atDCS, ctDCS, and dtDCS were found to better improve motor function than control treatments post-intervention,^54^ while another network meta-analysis of non-invasive brain stimulation favours the application of HFrTMS, LFrTMS, ctDCS, and dtDCS in improving motor function at the end of treatment.^56^ Findings from Tang et al. show that both atDCS and ctDCS were effective against upper limb motor dysfunction post-intervention,^57^ whereas another meta-analysis found a non-significant improvement trend towards ctDCS compared with atDCS.^58^ Monte-Silva and colleague^48^ found NMES is effective in improving upper limb motor impairment in the short term, rather than other terms, which is consistent with our findings. However, inhomogeneity in the findings should be noted. One plausible explanation is the difference in study aim and inclusion criteria, with this study focusing on the recovery of motor function in participants with post-stroke spasticity, while the previous reviews simultaneously included patients with or without post-stroke spasticity. Another reason may be due to the difference in the assessment scale used.

The strength of our study lies in the comprehensive retrieval, robust selection criteria, the largest number and most relevant RCTs included, and detailed data extraction process. Moreover, to the best of our knowledge, this study represents the first network meta-analysis to compare BoNT and all kinds of non-invasive electrical and magnetic neuromodulation for spasticity and motor impairment in participants with post-stroke spasticity, and our findings have the potential to inform future clinical practice and research. Apart from that, it used the best methods to comprehensively assess methodological issues, test transitivity and incoherence, analyse data available, explore potential effect modifiers, and consider both statistical significance and clinical importance. Although there was some evidence of imbalanced baseline characteristics (e.g., higher baseline MAS value) that may indicate a lack of transitivity in the network, conducting sensitivity and meta-regression analysis did not modify our overall conclusions. Lastly, it was reported in a transparent way.

Notwithstanding its significant findings, several limitations inevitably exist in our study. Most trials included have some concerns for within-study bias, which lowered the certainty of evidence; however, we presented full details about the risk of bias of all trials included and incorporated the certainty of evidence into each finding. Sparse nodes (rPMS investigated by three studies) and a relative scantiness of head-to-head comparison evidence were also found, leaving some comparisons between treatments indirect, some studies not being included in the network meta-analysis at a certain follow-up, and further stratified analysis for effect modifiers impossible. The inclusion of trials with small sample size may also inevitably lead to small-sample bias and inaccurate estimation of the between-study heterogeneity, although comparisons-adjusted funnel plots, Egger’s test, and Hartung-Knapp-Sidik-Jonkman variance correction were performed. We also observed that too few studies assessed long-term effects, especially studies investigating neuromodulation technologies. Therefore, the findings from this study cannot be generalised to long-term periods. The studies incorporated in this meta-analysis relied on a subjective approach (MAS) to measure spasticity, and did not adopt other scales such as modified Tardieu scale to improve comparability. Although MAS is the predominant outcome measure in both clinical and research environments and is backed by evidence for its reliability,^59^ it is inherently subjective.

We analysed only average treatment effects and were unable to assess potentially crucial clinical and demographical modifiers of treatment response for post-stroke spasticity at the individual patient level, such as type of stroke, duration of illness, severity of symptoms, or limb measured, as the lack of stratified data and rare studies focused on lower limb prevented further subgroup analysis. Fortunately, the transitivity assumption based on these factors was mostly assured in the network, and sensitivity and meta-regression analyses on limb measured and stroke stage also confirmed the main findings of this study.

The broad scope of this study might have affected the clinical implications. Of the included studies, the most commonly used cointervention was rehabilitation intervention, mainly including exercise therapy such as task-oriented training, Bobath, and virtual reality, as well as occupational therapy. However, while sensitivity and meta-regression analyses on cointervention confirmed the main findings of this study, another limitation was that trials using different rehabilitation interventions apart from interventions of interest were pooled together, so this study is unable to answer whether a specific rehabilitation intervention is more effective than another one when combined with BoNT or non-invasive neuromodulation. The diversity, multifaceted, and combined nature of these interventions make it hard to identify which components may have led to the desired outcomes. As this study focused on the efficacy of BoNT and non-invasive neuromodulation, the final determination of which rehabilitation intervention works best would require information beyond the scope of this study about the detailed protocol of rehabilitation intervention. Additionally, due to the insufficient quantity of trials retrieved and the paucity of information reported in the original trials, we were unable to define a more detailed classification of interventions of interest, but it was intended as a methodological strength to avoid a scarce network. In this study, only studies that combined interventions of interest with cointervention to compare the same cointervention were acceptable (e.g., BoNT plus rehabilitation vs. rehabilitation) to ensure that the add-on effects of the target intervention can be determined. We are confident that it might not compromise the clinical practice and recommendation of BoNT, HFrTMS, LFrTMS, and tDCS, as they are usually in combination with rehabilitation interventions, in clinical scenarios.

Additionally, we did not separate no treatment from control treatments, as rare studies reported a no treatment control to allow the analysis. Similarly, imaging or electrophysiological guided BoNT was not separated from BoNT,^60^ because scarce trials reported landmarks were used and it was beyond the scope of this study. A scarce network was also avoided by not separating them. Of note, sensitivity and meta-regression analyses were conducted to explore the impact of no treatment control, and the results made no significant differences.

Further trials should improve the planning conduct and reporting, such as stratifying the stroke based on the type of disease (ischemic, hemorrhagic, or embolic), syndrome severity (mild, moderate, or severe), stroke stage (acute, subacute, or chronic), and limb measured (upper or lower limbs), and conducting trials in a double-blind fashion, to increase our confidence in estimating the effects of BoNT and neuromodulation therapies. Additionally, more trials are warranted to determine which kind and component of rehabilitation intervention is beneficial for exerting the effects of BoNT and non-invasive neuromodulation for patients with post-stroke spasticity. Moreover, future trials should assess the long-term follow-up, cost-effectiveness, satisfaction, and other relevant outcomes aligned with the proposed intervention mechanisms, especially interventions observed in the sparse nodes, and if possible, provide stratified results in terms of effect modifiers, as this will guide clinicians and participants in their selection of the best therapy. Finally, reporting according to clinical reporting guidelines such as CONSORT in future research is strongly recommended to facilitate future systematic reviews and thus allow more robust and precise findings.

The study highlights the potential of three forms of tDCS as effective treatments for post-stroke spasticity and/or motor impairments, whereas BoNT, HFrTMS, and LFrTMS for motor impairments, considering clinical importance. However, due to the broad scope of this study, the vast majority of included trials involved cointerventions, which might have affected the clinical implications; therefore, we suggest policymakers and healthcare providers consider using and promoting tDCS alongside rehabilitation interventions such as exercise therapy and occupational therapy in appropriate patient populations. It is also unclear which kind and component of rehabilitation intervention works best in combination with neurostimulation, more well-designed trials comprising head-to-head comparisons are needed.

## Contributors

All authors contributed to the conception and design of the study and reviewed all documents and materials. JPH and CCB conceived the study. JPH, CCB, and YC collected the data and wrote the manuscript. JPH, CCB, WYZ conducted the statistical analysis. YC, WYZ, and KXZ prepared the tables and figures. CCB and JPH prepared the revised version of the manuscript. CLL and CZT critically revised the manuscript for important intellectual content and provided funding for editorial support. All authors read and approved the final version of the manuscript. JPH and CLL had full access to all of the data in the study and take responsibility for the integrity of the data and the accuracy of the data analysis.

## Data sharing statement

Data are presented in the current manuscript, its Supplementary Appendix 1, or within the manuscripts or appendices of the included studies.

## Declaration of interests

All authors declare no competing interests.
