# Supplementary material for: Comparative efficacy and acceptability of non-invasive neuromodulation technologies and botulinum toxin injections for post-stroke spasticity and motor function: a network meta-analysis of randomised controlled trials
Source: eClinicalMedicine. 2024 Dec 27;80:103034. doi: 10.1016/j.eclinm.2024.103034 (PMC11741030; doi:10.1016/j.eclinm.2024.103034)
Supplement: Appendix 1–20 [file mmc1.docx]

**Supplementary Materials**

**Comparative efficacy and acceptability of non-invasive neuromodulation technologies and botulinum toxin injections for post-stroke spasticity and motor function: a network meta-analysis of randomised controlled trials**

**Table of contents**

[Appendix 1. Literature search strategy used in the current review. 1](#_Toc183268951)

[Appendix 2. Definitions for the conditions and interventions. 4](#_Toc183268952)

[Appendix 3. Interpretation of the clinical importance. 6](#_Toc183268953)

[Appendix 4. Description of CINeMA domain evaluation. 7](#_Toc183268954)

[Appendix 5. Citations for 185 included trials. 9](#_Toc183268955)

[Appendix 6. List of excluded articles and reasons for exclusion. 24](#_Toc183268956)

[Appendix 7. Detailed table of included studies. 32](#_Toc183268957)

[Appendix 8. Risk of bias graph summary of the included studies. 59](#_Toc183268958)

[Appendix 9. Assessment of transitivity assumption. 64](#_Toc183268959)

[9.1 Studies and participants characteristics by interventions 64](#_Toc183268960)

[9.2 Studies and participants characteristics by pairwise comparisons 69](#_Toc183268961)

[9.2.1 Age 69](#_Toc183268962)

[9.2.2 Percentage of male 70](#_Toc183268963)

[9.2.3 Percentage of ischemic stroke 71](#_Toc183268964)

[9.2.4 Time since stroke 72](#_Toc183268965)

[9.2.5 Baseline MAS value 73](#_Toc183268966)

[9.3 Studies and participants characteristics by network 74](#_Toc183268967)

[9.3.1 Spasticity at short-term follow-up 74](#_Toc183268968)

[9.3.2 Spasticity at mid-term follow-up 79](#_Toc183268969)

[9.3.3 Motor function at short-term follow-up 84](#_Toc183268970)

[9.3.4 Motor function at mid-term follow-up 88](#_Toc183268971)

[9.3.5 Acceptability 92](#_Toc183268972)

[Appendix 10. Summary forest plots for each pairwise comparison. 96](#_Toc183268973)

[10.1 Spasticity at short-term follow-up 96](#_Toc183268974)

[10.2 Spasticity at mid-term follow-up 97](#_Toc183268975)

[10.3 Motor function at short-term follow-up 98](#_Toc183268976)

[10.4 Motor function at mid-term follow-up 99](#_Toc183268977)

[10.5 Acceptability 100](#_Toc183268978)

[Appendix 11. Detailed heterogeneities for direct meta-analyses. 102](#_Toc183268979)

[11.1 Heterogeneity for spasticity at short-term follow-up 102](#_Toc183268980)

[11.2 Heterogeneity for spasticity at mid-term follow-up 102](#_Toc183268981)

[11.3 Heterogeneity for motor function at short-term follow-up 103](#_Toc183268982)

[11.4 Heterogeneity for motor function at mid-term follow-up 103](#_Toc183268983)

[11.5 Heterogeneity for acceptability 104](#_Toc183268984)

[Appendix 12. Results of loop-specific heterogeneity estimates. 105](#_Toc183268985)

[12.1 Loop-specific heterogeneity estimates for spasticity at short-term follow-up 105](#_Toc183268986)

[12.2 Loop-specific heterogeneity estimates for spasticity at mid-term follow-up 105](#_Toc183268987)

[12.3 Loop-specific heterogeneity estimates for motor function at short-term follow-up 105](#_Toc183268988)

[12.4 Loop-specific heterogeneity estimates for motor function at mid-term follow-up 106](#_Toc183268989)

[12.5 Loop-specific heterogeneity estimates for acceptability 106](#_Toc183268990)

[Appendix 13. Results of node splitting. 107](#_Toc183268991)

[13.1 Results of node splitting for spasticity at short-term follow-up 107](#_Toc183268992)

[13.2 Results of node splitting for spasticity at mid-term follow-up 108](#_Toc183268993)

[13.3 Results of node splitting for motor function at short-term follow-up 108](#_Toc183268994)

[13.4 Results of node splitting for motor function at mid-term follow-up 109](#_Toc183268995)

[13.5 Results of node splitting for acceptability 109](#_Toc183268996)

[Appendix 14. Results of design-by-treatment interaction model. 110](#_Toc183268997)

[Appendix 15. Network meta-analysis results for each intervention for each outcome. 111](#_Toc183268998)

[15.1 Spasticity at short-term follow-up 111](#_Toc183268999)

[15.2 Spasticity at mid-term follow-up 114](#_Toc183269000)

[15.3 Motor function at short-term follow-up 116](#_Toc183269001)

[15.4 Motor function at mid-term follow-up 118](#_Toc183269002)

[15.5 Acceptability 120](#_Toc183269003)

[Appendix 16. Surface Under the Cumulative Ranking (SUCRA) and mean rank. 122](#_Toc183269004)

[16.1 Relative ranking of treatments of interest on spasticity at short-term follow-up 122](#_Toc183269005)

[16.2 Relative ranking of treatments of interest on spasticity at mid-term follow-up 123](#_Toc183269006)

[16.3 Relative ranking of treatments of interest on motor function at short-term follow-up 124](#_Toc183269007)

[16.4 Relative ranking of treatments of interest on motor function at mid-term follow-up 125](#_Toc183269008)

[16.5 Relative ranking of treatments of interest on acceptability 126](#_Toc183269009)

[Appendix 17. Sensitivity analysis. 127](#_Toc183269010)

[17.1 Exclusion of studies that measured spasticity on lower extremity 127](#_Toc183269011)

[17.1.1 Spasticity at short-term follow-up 127](#_Toc183269012)

[17.1.2 Spasticity at mid-term follow-up 135](#_Toc183269013)

[17.1.3 Motor function at short-term follow-up 141](#_Toc183269014)

[17.1.4 Motor function at mid-term follow-up 147](#_Toc183269015)

[17.1.5 Acceptability 151](#_Toc183269016)

[17.2 Exclusion of studies without additional co-intervention 157](#_Toc183269017)

[17.2.1 Spasticity at short-term follow-up 157](#_Toc183269018)

[17.2.2 Spasticity at mid-term follow-up 163](#_Toc183269019)

[17.2.3 Motor function at short-term follow-up 168](#_Toc183269020)

[17.2.4 Motor function at mid-term follow-up 174](#_Toc183269021)

[17.2.5 Acceptability 178](#_Toc183269022)

[17.3 Exclusion of studies with BoNT vs. Control comparison at short-term follow-up 184](#_Toc183269023)

[17.3.1 Spasticity at short-term follow-up 184](#_Toc183269024)

[17.3.2 Motor function at short-term follow-up 191](#_Toc183269025)

[17.3.3 Acceptability 195](#_Toc183269026)

[Appendix 18. Meta-regression analysis. 199](#_Toc183269027)

[18.1 Univariate meta-analysis analysis based on limb measured 199](#_Toc183269028)

[18.1.1 Spasticity at short-term follow-up 199](#_Toc183269029)

[18.1.2 Spasticity at mid-term follow-up 201](#_Toc183269030)

[18.1.3 Motor function at short-term follow-up 202](#_Toc183269031)

[18.1.4 Motor function at mid-term follow-up 203](#_Toc183269032)

[18.1.5 Acceptability 204](#_Toc183269033)

[18.2 Univariate meta-analysis analysis based on with or without cointervention 205](#_Toc183269034)

[18.2.1 Spasticity at short-term follow-up 205](#_Toc183269035)

[18.2.2 Spasticity at mid-term follow-up 206](#_Toc183269036)

[18.2.3 Motor function at short-term follow-up 207](#_Toc183269037)

[18.2.4 Motor function at mid-term follow-up 207](#_Toc183269038)

[18.2.5 Acceptability 207](#_Toc183269039)

[18.3 Univariate meta-analysis analysis based on stroke stage 208](#_Toc183269040)

[18.3.1 Spasticity at short-term follow-up 208](#_Toc183269041)

[18.3.2 Spasticity at mid-term follow-up 210](#_Toc183269042)

[18.3.3 Motor function at short-term follow-up 211](#_Toc183269043)

[18.3.4 Motor function at mid-term follow-up 213](#_Toc183269044)

[18.3.5 Acceptability 215](#_Toc183269045)

[18.4 Multivariate meta-analysis analysis based on limb measured, with or without cointervention, and stroke stage 216](#_Toc183269046)

[18.4.1 Spasticity at short-term follow-up 216](#_Toc183269047)

[18.4.2 Spasticity at mid-term follow-up 219](#_Toc183269048)

[18.4.3 Motor function at short-term follow-up 219](#_Toc183269049)

[18.4.4 Motor function at mid-term follow-up 221](#_Toc183269050)

[18.4.5 Acceptability 221](#_Toc183269051)

[Appendix 19. Publication bias. 223](#_Toc183269052)

[19.1 Spasticity at short-term follow-up 223](#_Toc183269053)

[19.2 Spasticity at mid-term follow-up 224](#_Toc183269054)

[19.3 Motor function at short-term follow-up 225](#_Toc183269055)

[19.4 Motor function at mid-term follow-up 227](#_Toc183269056)

[19.5 Acceptability 228](#_Toc183269057)

[Appendix 20. Grading for certainty of evidence (CINeMA assessment). 230](#_Toc183269058)

[20.1 Spasticity at short-term follow-up 230](#_Toc183269059)

[20.2 Spasticity at mid-term follow-up 233](#_Toc183269060)

[20.3 Motor function at short-term follow-up 235](#_Toc183269061)

[20.4 Motor function at mid-term follow-up 239](#_Toc183269062)

[20.5 Acceptability 240](#_Toc183269063)

# Appendix 1. Literature search strategy used in the current review.

| 1. **Cochrane Library (from inception to 8 October 2024)** | |
| --- | --- |
| #1 | MeSH descriptor: [Stroke] explode all trees |
| #2 | stroke OR cerebrovascular accident* OR CVA OR CVAs OR brain vascular accident* OR cerebrovascular disease OR cerebral infarction OR intracerebral hemorrhage OR hemiplegia OR hemiparesis OR apoplexy |
| #3 | MeSH descriptor: [Electric Stimulation Therapy] explode all trees |
| #4 | MeSH descriptor: [Magnetic Field Therapy] explode all trees |
| #5 | MeSH descriptor: [Botulinum Toxins] explode all trees |
| #6 | electrical Neuromodulation OR Electrical Stimulation OR ES OR Electrotherapy OR Electrical nerve stimulation OR ENS OR Neuromuscular Electrical Stimulation OR NMES OR Functional Electrical Stimulation OR FES OR Transcutaneous Electrical Nerve Stimulation OR TENS OR Transcutaneous Electric* Nerve Stimulation OR Transcutaneous Nerve Stimulation OR TNS OR Transcranial Direct Current Stimulation OR tDCS OR Transcranial Electrical Stimulation OR Vagal Nerve Stimulation OR VNS OR Interferential Current Therapy OR ICT OR Transcranial Magnetic Stimulation OR TMS OR Repetitive Transcranial Magnetic Stimulation OR rTMS OR Theta Burst Stimulation OR TBS OR Continuous Theta Burst Stimulation OR cTBS OR Intermittent Theta Burst Stimulation OR iTBS OR High Frequency Repetitive Transcranial Magnetic Stimulation OR HFrTMS OR Low Frequency Repetitive Transcranial Magnetic Stimulation OR LFrTMS OR Peripheral Magnetic Stimulation OR PMS OR Peripheral Magnetic Theta Burst Stimulation OR piTBS OR Repetitive Peripheral Magnetic Stimulation OR rPMS OR Botulinum Toxins OR Botulinum Toxin OR Botulinum Toxin Type A OR Botulinum Toxin A OR BoNTA OR BoNT OR BTX |
| #7 | MeSH descriptor: [Muscle Spasticity] explode all trees |
| #8 | Spasticity OR Muscle Tone OR Hypertonia OR Hypertonicity OR Spasm OR Spastic Muscle OR Equinus OR Equinovarus |
| #9 | Random or Random Allocation or Randomized controlled trials |
| #10 | #1 or #2 |
| #11 | #3 or #4 or #5 or #6 |
| #12 | #7 or #8 |
| #13 | #10 and #11 and #12 and #9 |
| 1. **MEDLINE (from inception to 8 October 2024)** | |
| #1 | (stroke[MeSH Terms]) OR (stroke OR cerebrovascular accident* OR CVA OR CVAs OR brain vascular accident* OR cerebrovascular disease OR cerebral infarction OR intracerebral hemorrhage OR hemiplegia OR hemiparesis OR apoplexy) |
| #2 | (((Electric Stimulation Therapy[MeSH Terms]) OR (Magnetic Field Therapy[MeSH Terms])) OR (Botulinum Toxins[MeSH Terms])) OR (Electrical Neuromodulation OR Electrical Stimulation OR ES OR Electrotherapy OR Electrical nerve stimulation OR ENS OR Neuromuscular Electrical Stimulation OR NMES OR Functional Electrical Stimulation OR FES OR Transcutaneous Electrical Nerve Stimulation OR TENS OR Transcutaneous Electric* Nerve Stimulation OR Transcutaneous Nerve Stimulation OR TNS OR Transcranial Direct Current Stimulation OR tDCS OR Transcranial Electrical Stimulation OR Vagal Nerve Stimulation OR VNS OR Interferential Current Therapy OR ICT OR Transcranial Magnetic Stimulation OR TMS OR Repetitive Transcranial Magnetic Stimulation OR rTMS OR Theta Burst Stimulation OR TBS OR Continuous Theta Burst Stimulation OR cTBS OR Intermittent Theta Burst Stimulation OR iTBS OR High Frequency Repetitive Transcranial Magnetic Stimulation OR HFrTMS OR Low Frequency Repetitive Transcranial Magnetic Stimulation OR LFrTMS OR Peripheral Magnetic Stimulation OR PMS OR Peripheral Magnetic Theta Burst Stimulation OR piTBS OR Repetitive Peripheral Magnetic Stimulation OR rPMS OR Botulinum Toxins OR Botulinum Toxin OR Botulinum Toxin Type A OR Botulinum Toxin A OR BoNTA OR BoNT OR BTX) |
| #3 | (muscle spasticity[MeSH Terms]) OR (spasticity OR Muscle Tone OR Hypertonia OR Hypertonicity OR Spasm OR Spastic Muscle OR Equinus OR Equinovarus) |
| #4 | random* |
| #5 | #1 AND #2 AND #3 AND #4 |
| 1. **EMBASE (from inception to 8 October 2024)** | |
| #1 | 'stroke'/exp |
| #2 | stroke OR cerebrovascular accident* OR CVA OR CVAs OR brain vascular accident* OR cerebrovascular disease OR cerebral infarction OR intracerebral hemorrhage OR hemiplegia OR hemiparesis OR apoplexy |
| #3 | 'electric stimulation therapy'/exp |
| #4 | 'magnetic field therapy'/exp |
| #5 | 'botulinum toxins'/exp |
| #6 | electrical Neuromodulation OR Electrical Stimulation OR ES OR Electrotherapy OR Electrical nerve stimulation OR ENS OR Neuromuscular Electrical Stimulation OR NMES OR Functional Electrical Stimulation OR FES OR Transcutaneous Electrical Nerve Stimulation OR TENS OR Transcutaneous Electric* Nerve Stimulation OR Transcutaneous Nerve Stimulation OR TNS OR Transcranial Direct Current Stimulation OR tDCS OR Transcranial Electrical Stimulation OR Vagal Nerve Stimulation OR VNS OR Interferential Current Therapy OR ICT OR Transcranial Magnetic Stimulation OR TMS OR Repetitive Transcranial Magnetic Stimulation OR rTMS OR Theta Burst Stimulation OR TBS OR Continuous Theta Burst Stimulation OR cTBS OR Intermittent Theta Burst Stimulation OR iTBS OR High Frequency Repetitive Transcranial Magnetic Stimulation OR HFrTMS OR Low Frequency Repetitive Transcranial Magnetic Stimulation OR LFrTMS OR Peripheral Magnetic Stimulation OR PMS OR Peripheral Magnetic Theta Burst Stimulation OR piTBS OR Repetitive Peripheral Magnetic Stimulation OR rPMS OR Botulinum Toxins OR Botulinum Toxin OR Botulinum Toxin Type A OR Botulinum Toxin A OR BoNTA OR BoNT OR BTX |
| #7 | 'muscle spasticity'/exp |
| #8 | spasticity OR Muscle Tone OR Hypertonia OR Hypertonicity OR Spasm OR Spastic Muscle OR Equinus OR Equinovarus |
| #9 | random* |
| #10 | #1 OR #2 |
| #11 | #3 OR #4 OR #5 OR #6 |
| #12 | #7 OR #8 |
| #13 | #9 AND #10 AND #11 AND #12 |

# Appendix 2. Definitions for the conditions and interventions.

| **Vocabulary** | **Vocabulary definitions** |
| --- | --- |
| Post-stroke spasticity | Post-stroke spasticity refers to a velocity-dependent increase in resistance during passive stretch, resulting from hyperexcitability of the stretch reflex following stroke. In this study, post-stroke spasticity was measured by modified Ashworth scale. |
| Motor function | Motor function refers to the ability of the body to perform movements through the coordination of muscles, bones, and the nervous system. In this study, motor function was measured by Fugl-Meyer Assessment. |
| Botulinum toxin injection | Botulinum toxin injection refers to a medical treatment that involves the use of botulinum toxin to reduce spasticity and/or improve motor impairment that occurs after a stroke. |
| High-frequency repetitive transcranial magnetic stimulation | 5 Hz or more defines high-frequency repetitive transcranial magnetic stimulation. |
| Low-frequency repetitive transcranial magnetic stimulation | 1 Hz or less defines low-frequency repetitive transcranial magnetic stimulation. |
| Continuous theta-burst stimulation | Continuous theta-burst stimulation is a form of repetitive transcranial magnetic stimulation that uses continuous theta-burst stimulation. |
| Intermittent theta-burst stimulation | Continuous theta-burst stimulation is a form of repetitive transcranial magnetic stimulation that uses intermittent theta-burst stimulation. |
| Anodal transcranial direct current stimulation | Anodal transcranial direct current stimulation is a form of transcranial direct current stimulation that places the anode electrode near the target area, delivering a weak electric direct current through the scalp. |
| Cathodal transcranial direct current stimulation | Cathodal transcranial direct current stimulation is a form of transcranial direct current stimulation that places the cathodal electrode near the target area, delivering a weak electric direct current through the scalp. |
| Dual transcranial direct current stimulation | Dual transcranial direct current stimulation is a form of transcranial direct current stimulation that simultaneously places both anodal and cathodal electrodes over the brain area, delivering a weak electric direct current through the scalp. |
| Neuromuscular electrical stimulation | Neuromuscular electrical stimulation uses a lightweight, battery-powered stimulator unit which, via self-adhesive electrodes, produces a controlled and comfortable contraction and relaxation of the underlying muscles. |
| Transcutaneous electrical nerve stimulation | Transcutaneous electrical nerve stimulation is a non-invasive technique that delivery pulsed electrical currents across the skin surface to stimulate peripheral nerves through electrode pads. |
| Repetitive peripheral magnetic stimulation | rPMS is a non-invasive technique that uses electromagnetic induction to generate electric currents in peripheral neuromuscular tissues and penetrates deeper conductive structures with relatively painless stimulation. |
| Cointerventions | Given the comparability, in this study, trials comparing interventions of interest plus other therapies with other therapies alone were acceptable and were deemed as interventions of interest versus control. Trials directly comparing interventions of interest with other therapies were excluded. |

# Appendix 3. Interpretation of the clinical importance.

Clinical importance was determined based on the guidelines proposed by Man-Son-Hing et al. which considers the relationship between the MCID of the treatment effect and the CI and designated to one of the following four different levels: (1) Definite – the MCID is smaller than the lower limit of the CI of the treatment effect, (2) Probable – the MCID is greater than the lower limit of the CI of the treatment effect, but smaller than the treatment effect, (3) Possible – the MCID is less than the upper limit of the CI of the treatment effect, but greater than the treatment effect, and (4) Definitely Not – the MCID is greater than the upper limit of the CI of the treatment effect.


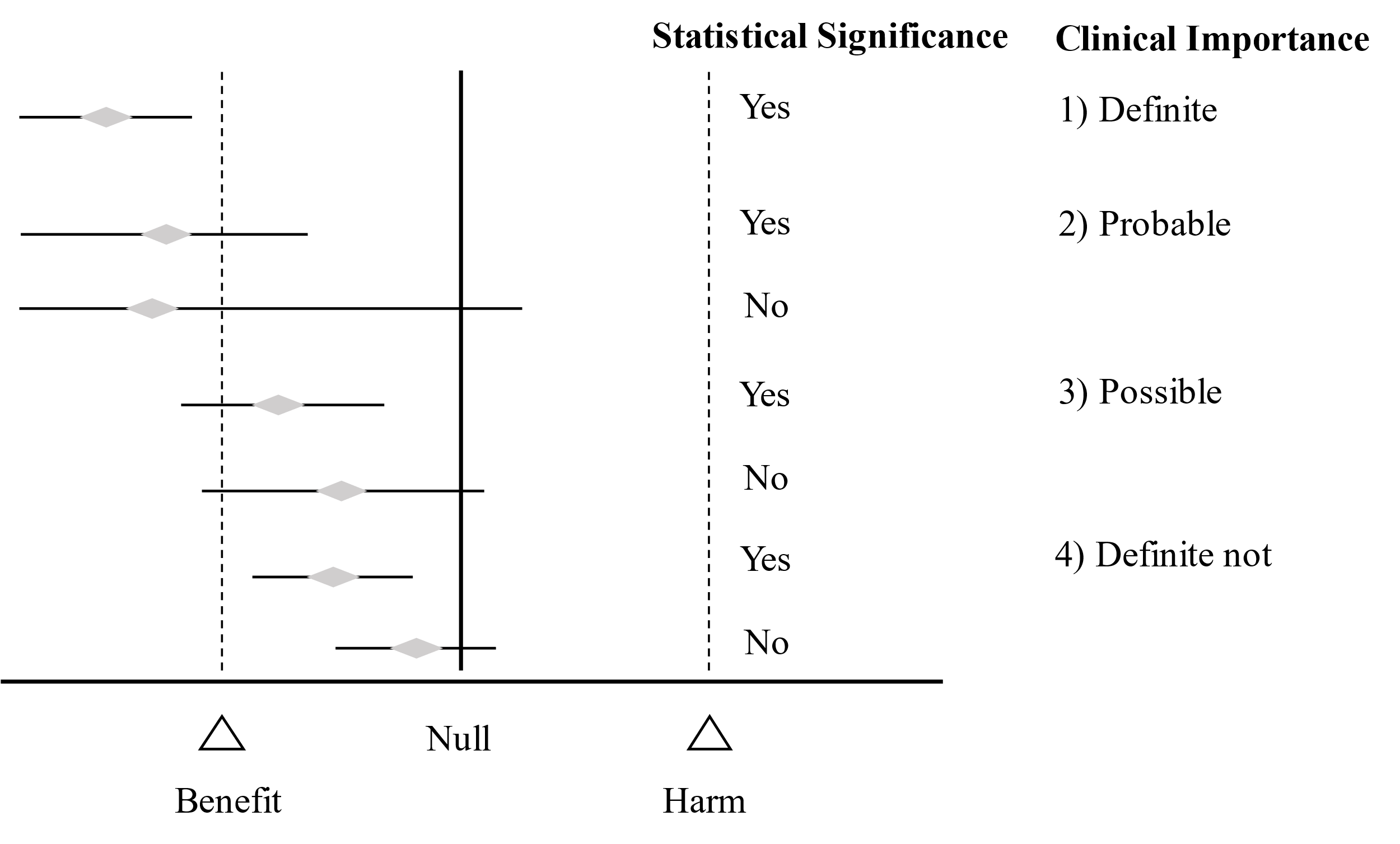


- Man-Son-Hing M, Laupacis A, O’Rourke K, Molnar FJ, Mahon J, Chan KBY, et al. Determination of the clinical importance of study results. J Gen Intern Med. 2002;17:469–476.

# Appendix 4. Description of CINeMA domain evaluation.

| **CINeMA** | **Description of assessment** |
| --- | --- |
| Within-study bias | We downgraded by one level when the contributions from low Risk of bias (RoB) comparisons were less than 25% and contributions from moderate or high RoB comparisons were 75% or greater. |
| Reporting bias | We carefully assessed the RoB due to missing evidence in NMA using ROB-MEN. We also assessed suspicion of publication bias by observation of patterns of results of small and large studies using comparisons-adjusted funnel plots and Egger’s test for pairwise comparisons with more than ten studies available. We rated pairwise comparisons as having suspected publication bias if the p-value <=0.10. However, we cannot completely rule out the possibility that some studies  are still missing. Considering that studies investigating active treatment versus control treatment are prone to publication bias, the review team decided by default to downgrade all these studies for potential publication bias by one level. |
| Indirectness | The population of interest was individuals with post-stroke spasticity. Age, sex proportion, ischemic stroke proportion, time since stroke, and baseline symptom severity were relevant for this study. Pairwise comparison indirectness was computed for the studies contributing to the direct and indirect meta-analyses using the CINeMA online platform based on individual study ratings. The interventions of interest were botulinum toxin (BoNT) and non-invasive neuromodulation, variants in dosages and parameters were observed. Selection criteria for this study were broad and heterogeneous and all studies were judged to provide relevant evidence. Transitivity was investigated by considering the distribution of baseline clinical characteristics, to modify this assessment; however, we did not modify this assessment if balanced clinical characteristics were found. |
| Impression | We considered the treatment effects included in the 95% confidence interval (CI) relative to potentially clinically important differences to assess the precision of the NMA estimate for each pairwise comparison. We defined a clinically important difference as 1 point for MAS, 6 points for motor function, and 1.5 points for acceptability. We rated estimates as ‘no concerns’ if the confidence interval was entirely on one side of the no effect line and did not cross clinically important effect. We rated estimates as ‘some concerns’ if the confidence interval extended into clinically important effects. We rated estimates as ‘major concerns’ if the confidence interval extended into clinically important effects in both directions. |
| Heterogeneity | We assessed heterogeneity by comparing the 95% CI of the pairwise NMA estimate with the prediction interval for NMA estimate. We judged heterogeneity in accordance with CINeMA recommendations, by assessing how many crossings of the interval with unimportant effects and clinically important value (one point for MAS, 6 points for motor function, and 1.5 points for acceptability) in opposite direction as point estimate. We judged estimates as ‘no concerns’ if the confidence interval and prediction interval are same; ‘some concerns’ if the prediction interval crosses one more zone than the confidence interval; ‘major concerns’ if the prediction interval extended into clinically important effects in both directions (e.g., CI are all negative; prediction interval extends to clinically important positive effect, i.e., crosses 0 to 1 and extends beyond 1). |
| Incoherence | We assessed incoherence for the evaluation of certainty of evidence using the design-by-treatment interaction model and node split approach. The node split approach separates the indirect meta-analysis effect from mixed network meta-analysis effect. We assessed incoherence using the approach recommended by CINeMA, which estimates an inconsistency factor with 95% CI (ratio of direct and indirect estimates) and assesses the difference between direct and indirect estimates considering the range of equivalence based on a clinically important difference of 1 point for MAS, 6 points for motor function, and 1.5 points for acceptability. |

# Appendix 5. Citations for 185 included trials.

1. Abramovich SG, Drobyshev VA, Pyatova AE, Yumashev AV, Koneva ES. Comprehensive Use of Dynamic Electrical Neurostimulation and Botulinum Toxin Therapy in Patients with Post-Stroke Spasticity. J Stroke Cerebrovasc Dis. 2020;29:105189.

2. Andrade SM, Batista LM, Nogueira L, et al. Constraint-induced movement therapy combined with transcranial direct current stimulation over premotor cortex improves motor function in severe stroke: a pilot randomized controlled trial. Rehabil Res Pract. 2017;2017:6842549.

3. Aşkın A, Tosun A, Demirdal Ü S. Effects of low-frequency repetitive transcranial magnetic stimulation on upper extremity motor recovery and functional outcomes in chronic stroke patients: A randomized controlled trial. Somatosens Mot Res. 2017;34:102-107.

4. Bakheit AM, Pittock S, Moore AP, et al. A randomized, double-blind, placebo-controlled study of the efficacy and safety of botulinum toxin type A in upper limb spasticity in patients with stroke. Eur J Neurol. 2001;8:559-565.

5. Bakheit AM, Thilmann AF, Ward AB, et al. A randomized, double-blind, placebo-controlled, dose-ranging study to compare the efficacy and safety of three doses of botulinum toxin type A (Dysport) with placebo in upper limb spasticity after stroke. Stroke. 2000;31:2402-2406.

6. Bakhtiary AH, Fatemy E. Does electrical stimulation reduce spasticity after stroke? A randomized controlled study. Clin Rehabil. 2008;22:418-425.

7. Barker RN, Brauer SG, Carson RG. Training of reaching in stroke survivors with severe and chronic upper limb paresis using a novel nonrobotic device: a randomized clinical trial. Stroke. 2008;39:1800-1807.

8. Barros Galvão SC, Borba Costa dos Santos R, Borba dos Santos P, Cabral ME, Monte-Silva K. Efficacy of coupling repetitive transcranial magnetic stimulation and physical therapy to reduce upper-limb spasticity in patients with stroke: a randomized controlled trial. Arch Phys Med Rehabil. 2014;95:222-229.

9. Bauer P, Krewer C, Golaszewski S, Koenig E, Müller F. Functional electrical stimulation-assisted active cycling--therapeutic effects in patients with hemiparesis from 7 days to 6 months after stroke: a randomized controlled pilot study. Arch Phys Med Rehabil. 2015;96:188-196.

10. Bian L, Zhang L, Huang G, et al. Effects of Priming Intermittent Theta Burst Stimulation With High-Definition tDCS on Upper Limb Function in Hemiparetic Patients With Stroke: A Randomized Controlled Study. Neurorehabil Neural Repair. 2024;38:268-278.

11. Boyaci A, Topuz O, Alkan H, et al. Comparison of the effectiveness of active and passive neuromuscular electrical stimulation of hemiplegic upper extremities: a randomized, controlled trial. Int J Rehabil Res. 2013;36:315-322.

12. Chan MK, Tong RK, Chung KY. Bilateral upper limb training with functional electric stimulation in patients with chronic stroke. Neurorehabil Neural Repair. 2009;23:357-365.

13. Chen SC, Chen YL, Chen CJ, Lai CH, Chiang WH, Chen WL. Effects of surface electrical stimulation on the muscle-tendon junction of spastic gastrocnemius in stroke patients. Disabil Rehabil. 2005;27:105-110.

14. Chen Y, Wei QC, Zhang MZ, et al. Cerebellar Intermittent Theta-Burst Stimulation Reduces Upper Limb Spasticity After Subacute Stroke: A Randomized Controlled Trial. Front Neural Circuits. 2021;15:655502.

15. Chen YH, Chen CL, Huang YZ, et al. Augmented efficacy of intermittent theta burst stimulation on the virtual reality-based cycling training for upper limb function in patients with stroke: a double-blinded, randomized controlled trial. J Neuroeng Rehabil. 2021;18:91.

16. Chen YJ, Huang YZ, Chen CY, et al. Intermittent theta burst stimulation enhances upper limb motor function in patients with chronic stroke: a pilot randomized controlled trial. BMC Neurol. 2019;19:69.

17. Chervyakov AV, Poydasheva AG, Lyukmanov RH, et al. Effects of Navigated Repetitive Transcranial Magnetic Stimulation After Stroke. J Clin Neurophysiol. 2018;35:166-172.

18. Chieffo R, Giatsidis F, Santangelo R, Alyagon U, Comola M, Zangen A. Repetitive Transcranial Magnetic Stimulation With H-Coil Coupled With Cycling for Improving Lower Limb Motor Function After Stroke: An Exploratory Study. Neuromodulation. 2021;24:916-922.

19. Childers MK, Brashear A, Jozefczyk P, et al. Dose-dependent response to intramuscular botulinum toxin type A for upper-limb spasticity in patients after a stroke. Arch Phys Med Rehabil. 2004;85:1063-1069.

20. de Melo Carvalho Rocha E, Riberto M. Use of Botulinum Toxin as a Treatment of Hemiplegic Shoulder Pain Syndrome: A Randomized Trial. Toxins. 2023;15.

21. Del Felice A, Daloli V, Masiero S, Manganotti P. Contralesional cathodal versus dual transcranial direct current stimulation for decreasing upper limb spasticity in chronic stroke individuals: a clinical and neurophysiological study. Journal of Stroke and Cerebrovascular Diseases. 2016;25:2932-2941.

22. Dos Santos RBC, Galvão SCB, Frederico LMP, et al. Cortical and spinal excitability changes after repetitive transcranial magnetic stimulation combined to physiotherapy in stroke spastic patients. Neurol Sci. 2019;40:1199-1207.

23. Ganesh GS, Kumari R, Pattnaik M, et al. Effectiveness of Faradic and Russian currents on plantar flexor muscle spasticity, ankle motor recovery, and functional gait in stroke patients. Physiother Res Int. 2018;23:e1705.

24. Gottlieb A, Boltzmann M, Schmidt SB, et al. Treatment of upper limb spasticity with inhibitory repetitive transcranial magnetic stimulation: A randomized placebo-controlled trial. NeuroRehabilitation. 2021;49:425-434.

25. Hara Y, Ogawa S, Tsujiuchi K, Muraoka Y. A home-based rehabilitation program for the hemiplegic upper extremity by power-assisted functional electrical stimulation. Disabil Rehabil. 2008;30:296-304.

26. Hesse S, Mach H, Fröhlich S, Behrend S, Werner C, Melzer I. An early botulinum toxin A treatment in subacute stroke patients may prevent a disabling finger flexor stiffness six months later: a randomized controlled trial. Clin Rehabil. 2012;26:237-245.

27. Hesse S, Reiter F, Konrad M, Jahnke MT. Botulinum toxin type A and short-term electrical stimulation in the treatment of upper limb flexor spasticity after stroke: a randomized, double-blind, placebo-controlled trial. Clin Rehabil. 1998;12:381-388.

28. Hesse S, Waldner A, Mehrholz J, Tomelleri C, Pohl M, Werner C. Combined transcranial direct current stimulation and robot-assisted arm training in subacute stroke patients: an exploratory, randomized multicenter trial. Neurorehabil Neural Repair. 2011;25:838-846.

29. Hokazono A, Etoh S, Jonoshita Y, Kawahira K, Shimodozono M. Combination therapy with repetitive facilitative exercise program and botulinum toxin type A to improve motor function for the upper-limb spastic paresis in chronic stroke: A randomized controlled trial. J Hand Ther. 2022;35:507-515.

30. Hua Q, Xia WG, Li BB, Liu FX, XY C. Effects of tDCS combined with virtual scene interactive training on upper limb function and ADL in hemiplegic patients with cerebral infraction (in Chinese). Chinese Journal of Rehabilitation. 2020;35:15-18.

31. Jahangir AW, Tan HJ, Norlinah MI, et al. Intramuscular injection of botulinum toxin for the treatment of wrist and finger spasticity after stroke. Med J Malaysia. 2007;62:319-322.

32. Kaji R, Osako Y, Suyama K, Maeda T, Uechi Y, Iwasaki M. Botulinum toxin type A in post-stroke lower limb spasticity: a multicenter, double-blind, placebo-controlled trial. J Neurol. 2010;257:1330-1337.

33. Kaji R, Osako Y, Suyama K, Maeda T, Uechi Y, Iwasaki M. Botulinum toxin type A in post-stroke upper limb spasticity. Curr Med Res Opin. 2010;26:1983-1992.

34. Kerzoncuf M, Viton JM, Pellas F, et al. Poststroke Postural Sway Improved by Botulinum Toxin: A Multicenter Randomized Double-blind Controlled Trial. Arch Phys Med Rehabil. 2020;101:242-248.

35. Kim TH, In TS, Cho HY. Task-related training combined with transcutaneous electrical nerve stimulation promotes upper limb functions in patients with chronic stroke. Tohoku J Exp Med. 2013;231:93-100.

36. Kuzu Ö, Adiguzel E, Kesikburun S, Yaşar E, Yılmaz B. The Effect of Sham Controlled Continuous Theta Burst Stimulation and Low Frequency Repetitive Transcranial Magnetic Stimulation on Upper Extremity Spasticity and Functional Recovery in Chronic Ischemic Stroke Patients. J Stroke Cerebrovasc Dis. 2021;30:105795.

37. Lee SJ, Chun MH. Combination transcranial direct current stimulation and virtual reality therapy for upper extremity training in patients with subacute stroke. Arch Phys Med Rehabil. 2014;95:431-438.

38. Lee YY, Lin KC, Cheng HJ, Wu CY, Hsieh YW, Chen CK. Effects of combining robot-assisted therapy with neuromuscular electrical stimulation on motor impairment, motor and daily function, and quality of life in patients with chronic stroke: a double-blinded randomized controlled trial. J Neuroeng Rehabil. 2015;12:96.

39. Lin Z, Yan T. Long-term effectiveness of neuromuscular electrical stimulation for promoting motor recovery of the upper extremity after stroke. J Rehabil Med. 2011;43:506-510.

40. Mangold S, Schuster C, Keller T, Zimmermann-Schlatter A, Ettlin T. Motor training of upper extremity with functional electrical stimulation in early stroke rehabilitation. Neurorehabil Neural Repair. 2009;23:184-190.

41. Marciniak CM, Harvey RL, Gagnon CM, et al. Does botulinum toxin type A decrease pain and lessen disability in hemiplegic survivors of stroke with shoulder pain and spasticity?: a randomized, double-blind, placebo-controlled trial. Am J Phys Med Rehabil. 2012;91:1007-1019.

42. Mazzoleni S, Tran VD, Dario P, Posteraro F. Effects of Transcranial Direct Current Stimulation (tDCS) Combined With Wrist Robot-Assisted Rehabilitation on Motor Recovery in Subacute Stroke Patients: A Randomized Controlled Trial. IEEE Trans Neural Syst Rehabil Eng. 2019;27:1458-1466.

43. McCrory P, Turner-Stokes L, Baguley IJ, et al. Botulinum toxin A for treatment of upper limb spasticity following stroke: a multi-centre randomized placebo-controlled study of the effects on quality of life and other person-centred outcomes. J Rehabil Med. 2009;41:536-544.

44. Mesci N, Ozdemir F, Kabayel DD, Tokuc B. The effects of neuromuscular electrical stimulation on clinical improvement in hemiplegic lower extremity rehabilitation in chronic stroke: a single-blind, randomised, controlled trial. Disabil Rehabil. 2009;31:2047-2054.

45. Nakipoğlu Yuzer GF, Köse Dönmez B, Özgirgin N. A Randomized Controlled Study: Effectiveness of Functional Electrical Stimulation on Wrist and Finger Flexor Spasticity in Hemiplegia. J Stroke Cerebrovasc Dis. 2017;26:1467-1471.

46. Ochi M, Saeki S, Oda T, Matsushima Y, Hachisuka K. Effects of anodal and cathodal transcranial direct current stimulation combined with robotic therapy on severely affected arms in chronic stroke patients. J Rehabil Med. 2013;45:137-140.

47. Özkeskin M, Öztürk V, Cakmur R, Bilge K, Küçük F. The Effects of Navigated Repetitive Transcranial Magnetic Simulation and Brunnstrom Movement Therapy on Upper Extremity Proprioceptive Sense and Spasticity in Stroke Patients: A Double-Blind Randomized Trial. Journal of Basic and Clinical Health Sciences. 2017;1:29-35.

48. Park J, Seo D, Choi W, Lee S. The effects of exercise with TENS on spasticity, balance, and gait in patients with chronic stroke: a randomized controlled trial. Med Sci Monit. 2014;20:1890-1896.

49. Picelli A, Dambruoso F, Bronzato M, Barausse M, Gandolfi M, Smania N. Efficacy of therapeutic ultrasound and transcutaneous electrical nerve stimulation compared with botulinum toxin type A in the treatment of spastic equinus in adults with chronic stroke: a pilot randomized controlled trial. Top Stroke Rehabil. 2014;21 Suppl 1:S8-16.

50. Prazeres A, Lira M, Aguiar P, Monteiro L, Vilasbôas Í, Melo A. Efficacy of physical therapy associated with botulinum toxin type A on functional performance in post-stroke spasticity: A randomized, double-blinded, placebo-controlled trial. Neurol Int. 2018;10:7385.

51. Qin Y, Liu X, Zhang Y, Wu J, Wang X. Effects of transcranial combined with peripheral repetitive magnetic stimulation on limb spasticity and resting-state brain activity in stroke patients. Front Hum Neurosci. 2023;17:992424.

52. Qu YP, Wu DY, Tu XQ, Qian L, Yang YB, H G. Effects of transcranial direct current stimulation on relieving upper-limb spasticity after stroke (in Chinese). Chin J Cerebrovasc. 2009;6:586-589.

53. Rastgoo M, Naghdi S, Nakhostin Ansari N, et al. Effects of repetitive transcranial magnetic stimulation on lower extremity spasticity and motor function in stroke patients. Neuromodulation. 2016;38:1918-1926.

54. Rosales RL, Balcaitiene J, Berard H, et al. Early AbobotulinumtoxinA (Dysport(®)) in Post-Stroke Adult Upper Limb Spasticity: ONTIME Pilot Study. Toxins. 2018;10.

55. Sabut SK, Sikdar C, Kumar R, Mahadevappa M. Functional electrical stimulation of dorsiflexor muscle: effects on dorsiflexor strength, plantarflexor spasticity, and motor recovery in stroke patients. NeuroRehabilitation. 2011;29:393-400.

56. Sahin N, Ugurlu H, Albayrak I. The efficacy of electrical stimulation in reducing the post-stroke spasticity: a randomized controlled study. Disabil Rehabil. 2012;34:151-156.

57. Sentandreu-Mañó T, Tomás JM, Ricardo Salom Terrádez J. A randomised clinical trial comparing 35 Hz versus 50 Hz frequency stimulation effects on hand motor recovery in older adults after stroke. Sci Rep. 2021;11:9131.

58. Shaw L, Rodgers H, Price C, et al. BoTULS: a multicentre randomised controlled trial to evaluate the clinical effectiveness and cost-effectiveness of treating upper limb spasticity due to stroke with botulinum toxin type A. Health Technol Assess. 2010;14:1-113, iii-iv.

59. Sonde L, Gip C, Fernaeus SE, Nilsson CG, Viitanen M. Stimulation with low frequency (1.7 Hz) transcutaneous electric nerve stimulation (low-tens) increases motor function of the post-stroke paretic arm. Scand J Rehabil Med. 1998;30:95-99.

60. Tan B, Jia L. Ultrasound-Guided BoNT-A (Botulinum Toxin A) Injection Into the Subscapularis for Hemiplegic Shoulder Pain: A Randomized, Double-Blind, Placebo-Controlled Trial. Stroke. 2021;52:3759-3767.

61. Tekeoğlu Y, Adak B, Göksoy T. Effect of transcutaneous electrical nerve stimulation (TENS) on Barthel Activities of Daily Living (ADL) index score following stroke. Clin Rehabil. 1998;12:277-280.

62. Viana RT, Laurentino GEC, Souza RJP, et al. Effects of the addition of transcranial direct current stimulation to virtual reality therapy after stroke: A pilot randomized controlled trial. NeuroRehabilitation. 2014;34:437-446.

63. Wang RF, Zhang KQ, Wu WY, et al. Clinical Study of Transcranial Direct Current Electrical Stimulation Combined with Exercise Therapy on Limb Dysfunction in Stroke Patients (in Chinese). Journal of Changzhi Medical College. 2019;33:353-356.

64. Watanabe K, Kudo Y, Sugawara E, et al. Comparative study of ipsilesional and contralesional repetitive transcranial magnetic stimulations for acute infarction. J Neurol Sci. 2018;384:10-14.

65. Wolf SL, Milton SB, Reiss A, Easley KA, Shenvi NV, Clark PC. Further assessment to determine the additive effect of botulinum toxin type A on an upper extremity exercise program to enhance function among individuals with chronic stroke but extensor capability. Arch Phys Med Rehabil. 2012;93:578-587.

66. Wu D, Qian L, Zorowitz RD, Zhang L, Qu Y, Yuan Y. Effects on decreasing upper-limb poststroke muscle tone using transcranial direct current stimulation: a randomized sham-controlled study. Arch Phys Med Rehabil. 2013;94:1-8.

67. Xu Q, Guo F, Salem HMA, Chen H, Huang X. Effects of mirror therapy combined with neuromuscular electrical stimulation on motor recovery of lower limbs and walking ability of patients with stroke: a randomized controlled study. Clin Rehabil. 2017;31:1583-1591.

68. Yang YR, Mi PL, Huang SF, Chiu SL, Liu YC, Wang RY. Effects of neuromuscular electrical stimulation on gait performance in chronic stroke with inadequate ankle control - A randomized controlled trial. PLOS one. 2018;13:e0208609.

69. Youssef H, Mohamed NAE, Hamdy M. Comparison of bihemispheric and unihemispheric M1 transcranial direct current stimulations during physical therapy in subacute stroke patients: A randomized controlled trial. Neurophysiol Clin. 2023;53:102895.

70. Zhou M, Li F, Lu W, Wu J, Pei S. Efficiency of Neuromuscular Electrical Stimulation and Transcutaneous Nerve Stimulation on Hemiplegic Shoulder Pain: A Randomized Controlled Trial. Arch Phys Med Rehabil. 2018;99:1730-1739.

71. Wang L. Effect of rTMS at different frequencies on limb function in poststroke hemiplegia patients (in Chinese). Henan Medical Research. 2021;30:66-68.

72. Li J, Wang W, Guo Y. Effect of transcranial direct current stimulation on the reduction of hand muscle tension in patients with stroke (in Chinese). Chin Heal Care Nutrit. 2017;14:144-145.

73. Zhao J, Shi M, Fan W, Chen B, Jin L. Rehabilitation effect of transcranial direct current stimulation on increased muscle tone after stroke and brain network mechanism (in Chinese). Clin Edu Gene Pract. 2023;21:407-409.

74. Zhang W. Clinical observation of EMG-guided botulinum toxin type A treatment for limb muscle spasm after stroke (in Chinese). Chin J Pract Nerv Dis. 2010;13:74-75.

75. Li J, Teng A, Bai H. Botox A injection combined with rehabilitation for spastic foot drop and foot varus after hemiplegia (in Chinese). J Xinxiang Med Coll. 2009;26:512-514.

76. Zhu Y, Shao M, Sun M, Zhang Z, Ma Q, Lin L. Efficacy analysis of repeated transcranial magnetic stimulation combined with rhythmic exercise training on lower limb function in patients with stroke (in Chinese). Modern Practical Medicine. 2023;35:1028-1031.

77. Zhu X, Zhang F, Dai J, et al. Therapeutic effect of acupoint catgut embedding combined with neuromuscular electrical stimulation in treating post-stroke upper limb spasm (in Chinese). JCAM. 2019;35:37-40.

78. Zhou Z, Shen X, Xiong L, et al. Effects of High-frequency Repetitive Transcranial Magnetic Stimulation to Premotor Areas on Upper Limb Motor Dysfunction after Stroke (in Chinese). Chin J Rehabil Theory Pract. 2020;26:697-702.

79. Zhou H, Duan H, Zhao Z, Hu J. The effect of upper limb robot training combined with multi-channel funetional electrical stimulation on upper limb motor function in elderly stroke patients (in Chinese). Geriatr Health Care. 2023;29:690-695.

80. Zhao R, Yan M. Application effect of transcranial direct current stimulation combined with Bobath technique in patients with upper limb spastic hemiplegia after hemorrhagic stroke (in Chinese). Clinical Research and Practice. 2021;6:166-168.

81. Zhao J. The curative effect of low-frequency repetitive rTMS on hemiplegia upper limb spasm and limb motor function in stroke patients (in Chinese). Clinical Research. 2021;29:83-85.

82. Zhang X. Effect of EMG-guided botulinum toxin A combined with conventional rehabilitation training on muscle spasm after stroke. World Latest Medicine Information. 2014;14:35-36.

83. Zhang S. Botulinum toxin A injection in the treatment of post-stroke patients with spsatic foot drop (in Chinese). National Medical Frontiers of China. 2010;5:11-12+12.

84. Zhang P, Zhao G. Effect of repetitive peripheral magnetic stimulation on spasm of flexor elbow muscles in stroke paralysis (in Chinese). Journal of Taizhou Polytechnic College. 2023;23:78-82.

85. Zhang M. Efficacy of acupuncture combined with botulinum toxin A injection for upper limb spasticity after stroke (in Chinese). Chin J Misdiagn. 2010;10:1286-1287.

86. Zhang L, Gong X, He L, Liu D, An H, Wang J. Observation of changes in cortical and spinal cord excitability after transcranial magnetic stimulation combined with physical therapy in stroke patients with spastic paralysis of the upper limbs (in Chinese). Guizhou Medical Journal. 2020;44:91-92.

87. Zhang D, Zhou J, Zhang M. Influence of cycling synchronized with functional electrical stimulation on upper limb function in post stroke patients (in Chinese). Chin J Rehabil Med. 2018;33:1050-1054.

88. Yuan M, Guo X, Zhang C, Qin Y. The effect of low-frequency repetitive transcranial magnetic stimulation combined with muscle energy technique for upper limb spasm after stroke (in Chinese). Shandong Medical Journal. 2020;60:63-66.

89. Yu F, Zhu Y, Liang S, et al. A randomized controlled study of transcranial and peripheral magnetic stimulation for upper limb motor dysfunction after stroke (in Chinese). Chin J Rehab Med. 2021;36:538-545.

90. Yu S. Effect observation of medium frequency electrical stimulation of peroneus longus and brevis muscles in auxiliary treatment of patients with post-stroke strephenopodia (in Chinese). Chin J Mod Drug Appl. 2022;16:61-63.

91. You H, Li X, Nie M, Zhang Y, Xie J, Wang Z. The effect of botulinum toxin type A on tibial nerve F-wave and motor function in patients with lower limb spasticity after stroke (in Chinese). Modern Medicine and Health Research. 2020;4:10-12.

92. Yi Y, Zou Y. The curative effect of transcranial direct current stimulation combined with muscle energy technique in the treatment of upper limb spasm after stroke (in Chinese). Chin J Phys Med Rehabil. 2021;43:1007-1009.

93. Yang Y, Liang Q, Wan X, et al. Safety and efficacy of botulinum toxin type A made in China for treatment of post-stroke upper limb spasticity: a randomized double-blind controlled trial (in Chinese). Chin J Neurol. 2018;51:355-363.

94. Yang Y, Hu L, Zou L, Hao Y, Zhang W, Cai X. Effect of repetitive transcranial magnetic stimulation combined with electromyographic biofeedback on upper limb functional recovery in stroke patients (in Chinese). Chin J Phys Med Rehabil. 2020;42:415-418.

95. Yang X, Zhang C, Liu M, Guo X, Liu X, Qin Y. Efficacy of rTMS combined with modern rehabilitation exercise therapy for spastic paralysis of the upper limb after stroke (in Chinese). World Latest Medicne Information (Electronic Version). 2021;21:104-105,138.

96. Yang W, Li Z. Effect of proprioceptive neuromuscular facilitation technology combined with functional electrical stimulation treadmill on upper limb function in stroke patients (in Chinese). J Mod Med Health. 2023;39:568-571.

97. Tao L, Xu H, Long X, Long Y. The effect of transcranial magnetic stimulation of vestibular balance projection points combined with enhanced vestibular sensory integration training on balance function and motor ability in stroke patients with hemiplegia (in Chinese). RARM. 2023;4:68-70,78.

98. Xia X, Xue M, Yang C, Ma J, Yao X. Ultrasound guidance combined with botulinum toxin type A in the treatment of post-stroke upper limb spasticity. Journal of Hebei Medical University. 2021;42:1194-1197.

99. Yang Q, Pan S. Effect of transcranial magnetic stimulation combined with multisensory game training on upper limb motor function in patients with upper limb spasticity after stroke (in Chinese). RARM. 2023;4:75-77,81.

100. Yang J, Zhang Q, Li W. Clinical efficacy of antagonistic facilitation acupuncture plus agonistic muscle botulinum toxin type A injection on upper extremity spasm in the recovery period of cerebral infarction (in Chinese). CJCM. 2021;13:91-94.

101. Xu J, Tan Y, Ao L. The effeet of Botulinum toxin type A (BTXA) on Brunnstrom stages of upper limb in stroke patients (in Chinese). Chin J Phys Med Rehabil. 2004;26:613-617.

102. Xiong W, Tang Q, Zhou Q. Effect of local injection of botulinum toxin combined with joint mobilization training in the treatment of hemiplegic limb spasm after stroke (in Chinese). Chinese Community Doctors. 2020;36:23-24+26.

103. Xiao C, Pan C, Chen Y, et al. Effect of high-frequency repetitive transcranial magnetic stimulation on hand function in patients after stroke (in Chinese). Chin J Rehabil Theory Pract. 2018;24:179-183.

104. Xiao C, Pan C, Chen Y, et al. Effects of high-frequency repetitive transcranial magnetic stimulation in different frequencies on upper limb function after ischemic stroke (in Chinese). Chin J Rehabil Theory Pract. 2019;25:557-563.

105. Xia X, Yang X, Xue M, Yang C. The application effects of ultrasound elastography in evaluating the curative effects of botulinum toxin A injection on forearm flexor spasm after cerebral stroke (in Chinese). Hebei Medical Journal. 2022;44:2622-2625,2629.

106. Xia M, Xie Y, Li X, Xie G. Efficacy of botulinum toxin A injection combined with rehabilitation training in 32 cases of muscle spasm after stroke (in Chinese). J Clin Res. 2014;31:602-604.

107. Xia M, Lin Z, Zhan Z, Jiang Y, Wei W. Clinical effect of local injection of btx-a and stimulated strengthen training in the treatment of foot drop and strephenopodia for stroke patients (in Chinese). China Health Standard Management. 2016;7:99-101.

108. Xia J, Chen M, Lin M, Xu Z, Hao Y. Effect of high frequency and low frequency repetitive transcranial magnetic stimulation in the treatment of post-stroke spasticity: A comparative study (in Chinese). Clinical Focus. 2022;37:427-430.

109. Xia J, Hao Y, Chen M, Shao Y. Clinical study of high-frequency repetitive transcranial magnetic stimulation combined with peripheral magnetic stimulation for the treatment of spasticity after stroke. Neural Injury And Functional Reconstruction. 2022;17:478-481.

110. Wu Y, Yang Y. The effect of botulinum toxin type A on upper limb spasticity and upper limb activity function in stroke patients (in Chinese). Health Care Today. 2015:43.

111. Wu T, Dong Y, Li J, Xu Z, Jiang D, Ye Y. Effect of early botulinum toxin intervention on gait and quality of life in stroke patients (in Chinese). Chin J Phys Med Rehabil. 2014;36:280-282.

112. Wu M, Jin Y. Efficacy of botulinum toxin injection combined with rehabilitation in treatment of foot drop after stroke (in Chinese). Zhejiang Medical Journal. 2019;41:1868-1871.

113. Wu D, Chen B, Zhang Y, Shao B. Curative effect observation of botulinum toxin a injections combined rehabilitation training in the treatment of upper limb spasm after cerebral apoplexy (in Chinese). Journal of Mathematical Medicine. 2017;30:4-6.

114. Wang Y, Xi J, Zong L, Su M. Curative effect of low-frequency neuromuscular electrical stimulation combined with baclofen in treating stroke-induced hemiplegia patients accompanied with myospasm (in Chinese). PJCCPVD. 2018;26:80-83.

115. Wang R. The improvement effect of intramuscular injection of botulinum toxin type A combined with rehabilitation training on upper limb spasticity in stroke patients (in Chinese). Chinese Journal of Gerontology. 2013;33:5354-5355.

116. Wang M, Yang W, Wang B, Cai C. Ultrasound-guided botulinum toxin A injection to improve the motor function of the affected side in stroke patients (in Chinese). Electronic Journal of Clinical Medical Literature. 2022;9:53-55.

117. Wang J, Wang X, Song N, Zhang X, Wu S. Effects of different transcranial magnetic stimulation modes on motor function of patients with lower limb dyskinesia after stroke (in Chinese). Journal of Guizhou Medical University. 2023;48:702-709.

118. Wang H, Yuan H, Mou X, et al. The effect of botulinum toxin type A combined with repetitive transcmnial magnetic stimulation on spas ticity of lower limbs in stroke patients (in Chinese). Chin J Rehabil Med. 2016;31:936-940.

119. Wang H, Zhao C, Yuan H, et al. The effect of botulinum toxin type A combined with extracOrpOreal shock wave therapy on spasticity of lower limbs in stroke patients (in Chinese). Chin J Rehabil Med. 2017;32:773-778.

120. Wang G, Jia J. Efficacy of functional electrical stimulation on stroke patients with foot drop and varus ankle (in Chinese). Chin J Rehabil. 2016;31:434-437.

121. Wang F, Wang Q, Wang D. Clinical analysis of Botox A injection combined with rehabilitation training for spastic foot drop in stroke (in Chinese). Zhejiang Clinical Medical Journal. 2016;18:2304-2306.

122. Tao J, Wei Y. Long term efficacy and safety of repetitive transcranial magnetic stimulation combined with repeated injection of botulinum toxin type A in the treatment of spasticity of lower limb muscles spasm after stroke (in Chinese). Journal of Brain and Nervous Diseases. 2018;26:272-276.

123. Tang N. Effect of high-frequency repetitive transcranial magnetic stimulation on the functional recovery of upper limbs in stroke patients (in Chinese). Chinese and Foreign Medical Research. 2021;19:158-160.

124. Sun W, Zhao C, Mou X, Liu W, Yuan H. Clinical study of low frequency repetitive transcranial magnetic stimulation in the treatment of upper limb spasm in patients with stroke (in Chinese). Chin J Rehabil. 2017;32:102-105.

125. Sun F, Ding Y, Xie J. Efficacy of ultrasound-guided BTX-A combined with acupuncture on gait function in patients with spastic foot drop and foot varus in stroke (in Chinese). Zhejiang Clinical Medicine Journal. 2023;25:1166-1168.

126. Su S, Long Z. Efficacy of ultrasound-guided botulinum toxin injection on lower limb spasticity (in Chinese). Journal of North Pharmacy. 2021;18:83-84+124.

127. Song G, Wang S, Zhao Y, Men Y. Efficacy and safety of neuromuscular electrical stimulation combined with baclofen in the treatment of spasticity after stroke (in Chinese). Chin J Rehabil Med. 2014;29:978-980.

128. Shan L, Cui L, Yang Y. Application of botulinum toxin A on recovery of limb spasticity after stroke (in Chinese). Neural InjuryAnd Functional Reconstruction. 2016;11:236-237,277.

129. Ren L, Han M, Bai R, Feng H, Shao Y. Research on botulinum toxin type A in the treatment of spastic foot drop and inversion after stroke (in Chinese). Chinese Journal of Practical Nervous Diseases. 2008;11:11-13.

130. Qu Y, Shan L, Liu C, Zhao K, Xiao R, Shen X. Analysis of the clinical effect of botulinum toxin type a (Botox-A) combined with electromyographic biofeedback therapy on the upper limb muscle spasm after stroke (in Chinese). Progress in Modern Biomedicine. 2017;17:5323-5326.

131. Peng H, Yang L, Zhou C. Clinical effect of ultrasound-guided injection of botulinum toxin type A in the treatment of post-stroke spasticity (in Chinese). Contemporary Medicine. 2022;28:91-95.

132. Peng Y, Fu L. Effect of Botulinum Toxin A on Spastic Upper Limbs Dysfunction after Stroke (in Chinese). Hebei Medicine. 2014;20:1968-1970.

133. Pan A, Chen Y, Chen J, Zhou Y, Liu Y, Chen R. Effect of combining CIMT and tDCS on upper limb motor function and degree of spasticity in elderly stroke patients (in Chinese). Chinese Journal of Gerontology. 2023;43:5513-5516.

134. Dou W, Liu X, Ma Y, Gong W. The effect and mechanism analysis of A-type botulinum toxin combined with virtual training on upper limb function recovery after stroke (in Chinese). Chinese Journal of Trauma and Disability Medicine. 2019;27:84-86.

135. Pan H. Clinical analysis of Botox injection combined with acupuncture for the treatment of increased limb muscle tone after stroke (in Chinese). Modern Practical Medicine. 2016;28:454-456.

136. Meng L, Lin G, Tang X. Therapeutic effect of botulinum toxin type A on upper extremity spasticity after stroke (in Chinese). Chin J Neuromed. 2008;7:740-742.

137. Meng J, Li R. Effect of low-frequency electrical stimulation to antagonist muscles on the hemiparetic limb function in stroke patients (in Chinese). Chinese General Practice. 2009;12:1623-1624.

138. Ma S, Xu J, Ma N, Lin W, Fu S, Tao G. Efficacy of repeated injeetion of botulinum toxin type A under ultrasound guidance combined with comprehensive rehabilitation for lower limb spasticity in poststroke patients (in Chinese). Chin J Rehabil. 2019;34:567-570.

139. Luo S, Wang J, Wu X, Cheng D, Long Y. The study of botulinum toxin type A combinedwith rehabilitation training for stroke patients'lowerextremities with spastic paralysis (in Chinese). Chin J Rehabil Med. 2009;24:817-820.

140. Liu Z, Yang Y, Zhao Y, Gao H. Clinical study of transcranial direct current stimulation combined with myoelectric biofeedback in the treatment of upper extremity spasticity after stroke (in Chinese). Chinese Journal of Practical Nervous Diseases. 2022;25:1250-1255.

141. Liu Y, Zhang C, Qin Y. Therapeutic effect of high frequency rTMS on upper limb spasticity after stroke (in Chinese). Chin Fore Med Treat. 2019;17:11-13.

142. Liu Y, Wang X, Zhang C, et al. Effects of low-frequency repetitive transcranial magnetic stimulation on upper limb spasticity after stroke: a task-state functional magnetic resonance study (in Chinese). Chin J Rehabil Theory Pract. 2018;24:828-833.

143. Liu X, Wang M, Zhu G. Clinical effect of MOTOmed intelligent training combined with spastic myoelectric stimulation in the treatment of lower limb muscle spasticity in stroke patients (in Chinese). J Bengbu Med Coll. 2019;44:317-319,324.

144. Liu X, Wang L, Hong P, Liu Z. Efficacy of Botox and rehabilitation training in the treatment of upper limb spasm after stroke (in Chinese). Chin J Phys Med Rehabil. 2006;28:712-714.

145. Liu S, Li Z, Guo G, et al. Clinical research on the influence of low-frequency repetitive transeranial magnetie stimulation on spastici.ty and motor function of patients after stroke (in Chinese). Chin J Rehabil Med. 2019;34:1328-1332.

146. Liu S, Li Z, Liu Q, Guo G, LI Y, Hao D. Low-frequency repetitive transcranial magnetic stimulation combined with electromyographic biofeedback therapy for upper limb function after stroke (in Chinese). Chin J Rehabil. 2018;33:451-454.

147. Liu S, Ye T, Shen J, Yang J, Chen C. The effect of repetitive transcranial magnetic stimulation combined with electroacupuncture on upper limb motor function and serum BDNF, NGF in patients with stroke hemiplegia during recovery period (in Chinese). Chinese Journal of Gerontology. 2023;43:2578-2581.

148. Liang J, Li T, Wang Y, Liu S, Lv M. Effects of 1 Hz low- frequency repetitive transcranial magnetic stimulation combined with exogenous nerve growth factor therapy on limbs dyskinesia in stroke patients (in Chinese). Smart Healthcare. 2023;9:141-144,148.

149. Liang H. Effect of rTMS therapy in patients with upper limb hemiplegia after stroke (in Chinese). Practical Clinical Journal of Integrated Traditional Chinese and Western Medicine. 2024;24:52-54.

150. Li Y, Xiao Y, Yu L. Clinical study of botulinum toxin type A injection combined with exercise therapy in the treatment of spastic hemiplegia after stroke (in Chinese). Jiangxi Medical Journal. 2019;54:326-328.

151. Li Q, Cheng R, Wen W, Ye X. Evaluation on the efficacy of repetitive facilitative exercise combined with low-frequency repetitiVe transcranial magnetic stimulation for post-stroke upper extremity dysfunction (in Chinese). China Modern Doctor. 2018;56:1-5,10.

152. Li J, Hu Y. Efficacy of Botox A and rehabilitation training for upper limb spasticity after stroke (in Chinese). PJCCPVD. 2018;26:148-150.

153. Li J. Clinical analysis of Botox injection A combined with acupuncture for the treatment of spastucuty after stroke (in Chinese). J clin Med. 2017;4:14288-14289.

154. Le L, Guo G, Li Z. Ultrasound-guided botulinum toxin therapy for 34 cases of upper limb spasticity after cerebral hemorrhage (in Chinese). Chinese Journal of Gerontology. 2012;32:190-191.

155. Lan Y, Xu G, Hu X, LI K, Huang D. Effects of Botulinum toxin type A (BX-A) injection and comprehensive anti-spasticity treatment on qualityof life of stroke survivors (in Chinese). Chin J Rehabil Med. 2007;22:912-914.

156. Lan Y, Dou Z, Hu X, Xu G, Qiu W. Clinical study of Botulinum toxin type A injection in the treatment of upper limb spasticity in stroke patients (in Chinese). Chin J Phys Med Rehabil. 2007;29:754-757.

157. Jiang Y, Xie R, Cao H, Liu Y, Zhang Y, Peng L. Effect of type A botulinus toxin combined with targeted training on the wrist function and pain indexes inpatients with stroke complicated by spasm (in Chinese). Hainan Med J. 2016;27:1445-1447.

158. Jiang Y, Wang J, Wang M, et al. Effect of electroacupuncture combined with low-frequency repetitive TMS on limb spasticity and motor function in stroke patients (in Chinese). Guangxi Journal of Traditional Chinese Medicine. 2023;46:37-40.

159. Jiang X, Wang W. Curative effect analysis of low frequency repetitive transcranial magnetic stimulation combined with low frequency spasmodic muscle in the treatment of upper limb spasticity after stroke (in Chinese). Journal of Jinzhou Medical University. 2020;41:52-55.

160. Huang J, Xia Y, Liu B, Wang B, He Y, Zhang Y. Efficacy of electrical stimulation-guided botulinum toxin A injection for hemiplegia spasm after stroke (in Chinese). Chin J Phys Med Rehabil. 2018;40:350-352.

161. Huang G, Xu Y, Ren C, et al. Functional electrical stimulation on lower limb spasticity and its function in stroke patients evaluated by surface electro myography in combination with isokinetic tester (in Chinese). Chin J Rehabli. 2022;37:17-20.

162. Huang C, Han B, Wang H, Sun W. Clinical observation of acupuncture combined with transcranial magnetic stimulation in the treatment of hemiplegic foot drop after stroke (in Chinses). Journal of Guangzhou University of Traditional Chinese Medicine. 2023;40:1960-1967.

163. Hu Q, Ge L, Jiang H, Wu H. Effect of botulinum toxin injection combined with functional training on motor function in patients with cerebral infarction (in Chinses). Chin J Rehabil. 2014;29:107-108.

164. Hu H, Yao F, Chen J, et al. Clinical study of transcranial direct current combined with rehabilitation training on post-stroke dysfunction (in Chinese). Medical Innovation of China. 2023;20:49-53.

165. Hong H, Xu J, Shen X, et al. Effect of early precision injection of EMG-guided botulinum toxin A on upper limb functional recovery in the hemiplegic side of stroke (in Chinese). Chin J Phys Med Rehabil. 2018;40:499-502.

166. Hao S, YH. W, ZH. L, et al. Observation on the curative effect of botulinum toxin-A combined with magnetic stimulation on upper extremity motor dysfunction after stroke (in Chinese). Modern Journal of Integrated Traditional Chinese and Western Medicine. 2022;31:1939-1944+1949.

167. Fu Q, Chen G, Meng F, Fu L. Effect of type A borulinus toxin on immunological function in the treatment of post-stroke limb spasticity: a randomized,double blind, placebo-controlled trial (in Chinese). Chin J Clin Rehabil. 2005;9:16-17.

168. Deng L, Wu X, Xu M. Efficacy of high-frequency repetitive transcranial magnetic stimulation combined with task-oriented training on hand function rehabilitation in stroke patients (in Chinese). Journal of China Prescription Drug. 2019;17:132-134.

169. Cui Y, Hu F, Wang H. Study on botulinum toxin type A combined with rehabilitaiton for the treatment of lower limb muscle spasms in stroke patients (in Chinese). Biped and Health. 2019;28:47-48.

170. Cheng R, Tang H, Zhang Y, Chen W. Effect of low frequency repetitive transcranial magnetic stimulation on patients with spastic dyskinesia after ischemic stroke (in Chinese). Journal of Beihua University (Natural Science). 2022;23:79-83.

171. Cui L, Zhang T. Domesitical botulinum toxin type A injection in the treatment of post-stroke patients with upper extremity spasticity (in Chinese). Chin J Neurol. 2006;39:463-466.

172. Chen G, LI L, Cui W, Li Z. Clinical curative effect observation of precise botulinum toxin injection combined with small needle knife in the treatment of post-stroke upper extremity spasticity (in Chinese). Guid J Trad Chin Med Pharm. 2018;24:53-55.

173. Cheng P, Jin H, Zheng J, Liu Z. Clinical research on transcranial direct current stimulation and electrical acupuncture therapy for upper limb spasticity after stroke (in Chinese). Chin Arch Trad Chin Med. 2015;33:1994-1997.

174. Chen S. Study of botulinum toxin type A combined with Bobath technique for ankle dysfunction in spastic stroke patients (in Chinese). J Nor Pharm. 2016;13:133-134.

175. Cheng G, Cai H, Yin L, Ouyang W, Yuan W, Wu J. Effect of Botox combined with rehabilitation training for upper limb spasticity in early hemiplegia patients (in Chinese). Chin Fore Med Treat. 2010;29:56-57.

176. Chen Y. Effects of repetitive transcranial magnetic stimulation on spasm and motor function of lower limbs in patients with stroke (in Chinese). Chongqing Med J. 2018;47:3292-3295+3298.

177. Chen X, Wang Q, Zhang Y, Dong Y, Yang F. The effect of different frequency of functional electrical stimulation therapy on lower limb function in stroke patients with hemiplegia (in Chinese). Chin J Phys Med Rehabil. 2015;37:421-423.

178. Chen X, Guan D, Yang L, Cao Z. Effect of neuromuscular electrical stimulation combined with rehabilitation care extension training in patients with muscle spasm after stroke (in Chinese). J Qilu Nurs. 2021;27:56-59.

179. Chen F, Chen Z, Liang X, Lin H. Botulinum toxin type A for limb functional recover in high spasticity patients with stroke (in Chinese). Clin J Clin Rehabil 2003;7:3478-3479.

180. Chen F, Huang H, Chen Z, Ni G. Effects of low—frequency repetitive transcranial magnetic stimulation combined with MOTOmed gracile on upper limb spasticity after stroke (in Chinese). Chin J Rehabil Med. 2021;36:437-442.

181. Bu L. Effect of neuromuscular electrical stimulation therapy with rehabilitation nursing on limb function recovery in elderly hemiplegia after stroke (in Chinese). Life Sci Inst. 2022;S1:10-12.

182. Zhou JS, LV X, Zhang LL, et al. Application of transcranial direct current stimulation combined with conventional adjuvant therapy in the rehabilitation of upper limb function in stroke patients with hemiplegia (in Chinese). Modern practical medicine. 2020;32:579-580.

183. Liu J, Qian Q, He Q, Qin J. Effect of Botulinum Toxin A Combmed wlth Exerclse Program Sit-to-stand Tralnlng on Lower Limb Spasticlty and BaIance Ability in Patients with stroke. J Med Res 2024; 53(6): 109-13.

184. Fujimura K, Kagaya H, Itoh R, Endo C, Tanikawa H, Maeda H. Repetitive peripheral magnetic stimulation for preventing shoulder subluxation after stroke: a randomized controlled trial. Eur J Phys Rehabil Med 2024; 60(2): 216-24.

185. Abdelkader AA, Afifi LM, Maher EA, Atteya AA, El Salmawy DA. Comparison of Bilateral Versus Unilateral 5 Hz or 1 Hz Repetitive Transcranial Magnetic Stimulation in Subacute Stroke: Assessment of Motor Function in a Randomized Controlled Study. Journal of clinical neurophysiology: official publication of the American Electroencephalographic Society 2024; 41(5): 478-83.

# Appendix 6. List of excluded articles and reasons for exclusion.

| **First author,**  **year** | **Title** |
| --- | --- |
| **1. Ineligible population** | |
| Esquenazi, 2021 | The Effect of Repeated abobotulinumtoxinA (Dysport®) Injections on Walking Velocity in Persons with Spastic Hemiparesis Caused by Stroke or Traumatic Brain Injury |
| de Sousa, 2016 | Functional electrical stimulation cycling does not improve mobility in people with acquired brain injury and its effects on strength are unclear: a randomised trial |
| Embrey, 2010 | Functional electrical stimulation to dorsiflexors and plantar flexors during gait to improve walking in adults with chronic hemiplegia |
| Shen, 2018 | Effects of functional electrical stimulation cycling on the mobilisation of upper limbs in patients with early stroke (in Chinese) |
| Tian, 2020 | Effects of active functional electrical stimulation cycling on upper limbs function in patients with moderate-to-severe hemiplegia (in Chinese) |
| Xu, 2019 | The effects of multi-muscles co-contraction based functional electrical stimulation on the lower extremities’ motor function among early stroke patients (in Chinese) |
| Xu, 2022 | Effect of neuromuscular electrical stimulation combined with Rood technology on upper limb function in stroke patients with flaccid paralysis (in Chinese) |
| El Nahas, 2022 | Peripheral magnetic theta burst stimulation to muscles can effectively reduce spasticity: a randomized controlled trial |
| Krewer, 2014 | Effects of repetitive peripheral magnetic stimulation on upper-limb spasticity and impairment in patients with spastic hemiparesis: a randomized, double-blind, sham-controlled study |
| Werner, 2016 | Repetitive peripheral magnetic stimulation (rpMS) in combination with muscle stretch decreased the wrist and finger flexor muscle spasticity in chronic patients after CNS lesion |
| Xie, 2023 | Combining low-frequency transcranial magnetic stimulation with acupuncture in treating upper limb motor dysfunction after a stroke (in Chinese) |
| Ertzgaard, 2018 | Evaluation of a self-administered transcutaneous electrical stimulation concept for the treatment of spasticity: a randomized placebo-controlled trial |
| Li, 2009 | Transcutaneous nerve electrical stimulation combined with rehabilitation training for the treatment of spastic limb paralysis (in Chinese) |
| **2. Ineligible intervention** | |
| Marco, 2007 | Is botulinum toxin type A effective in the treatment of spastic shoulder pain in patients after stroke? A double-blind randomized clinical trial |
| Zhang, 2018 | Effect of Botulinum toxin A for stroke in different periods (in Chinese) |
| Suh, 2014 | Immediate therapeutic effect of interferential current therapy on spasticity, balance, and gait function in chronic stroke patients: a randomized control trial |
| Baricich, 2019 | Electrical stimulation of antagonist muscles after botulinum toxin type A for post-stroke spastic equinus foot. A randomized single-blind pilot study |
| Etoh, 2019 | Effects of concomitant neuromuscular electrical stimulation during repetitive transcranial magnetic stimulation before repetitive facilitation exercise on the hemiparetic hand |
| Hara, 2006 | Hybrid power-assisted functional electrical stimulation to improve hemiparetic upper-extremity function |
| Nam, 2022 | An Exoneuromusculoskeleton for Self-Help Upper Limb Rehabilitation After Stroke |
| Ring, 2005 | Controlled study of neuroprosthetic functional electrical stimulation in sub-acute post-stroke rehabilitation |
| Straudi, 2020 | Effects of a Robot-Assisted Arm Training Plus Hand Functional Electrical Stimulation on Recovery After Stroke: A Randomized Clinical Trial |
| Cheng, 2016 | Functional electrical stimulation and weight loss treadmill synchronous training after botulinum toxin injection can relieve foot-drop and strephenopodia among stroke survivors (in Chinese) |
| Lou, 2015 | The effect of early rehabilitation plus low frequency electrical stimulation in restoring the movement function and quality of life of patients with hemiplegia resulting from acute stroke (in Chinese) |
| Mao, 2007 | Staged treatment of different muscle tone in hemiplegic limbs after stroke using electrical stimulation (in Chinese) |
| Wan, 2013 | Observation of the therapeutic effect of transient electrical stimulation on post-stroke paralysis (in Chinese) |
| Qin, 2023 | Effects of transcranial combined with peripheral repetitive magnetic stimulation on limb spasticity and resting-state brain activity in stroke patients |
| Li, 2018 | Immediate Effects of Repetitive Peripheral Magnetic Stimulation on Upper Limb Spasticity and Motor Function for Stroke Patients (in Chinese) |
| Xu, 2021 | The effect of peripheral magnetic stimulation combined with transcranial magnetic stimulation on upper limb spasm after stroke |
| Etoh, 2013 | Effects of repetitive transcranial magnetic stimulation on repetitive facilitation exercises of the hemiplegic hand in chronic stroke patients |
| Şengül, 2023 | Anti-spastic effect of contralesional dorsal premotor cortex stimulation in stroke patients with moderate-to-severe spastic paresis: a randomized, controlled pilot trial |
| da Cunha, 2022 | Bicephalic Transcranial Direct-Current Stimulation Does Not Add Benefits to a Footdrop Stimulator for Improving Functional Mobility in People With Chronic Hemiparesis After Stroke: A Double-Blind, Randomized Controlled Trial |
| Ehsani, 2022 | The effects of concurrent M1 anodal tDCS and physical therapy interventions on function of ankle muscles in patients with stroke: a randomized, double-blinded sham-controlled trial study |
| Halakoo, 2021 | Does anodal trans-cranial direct current stimulation of the damaged primary motor cortex affects wrist flexor muscle spasticity and also activity of the wrist flexor and extensor muscles in patients with stroke?: a Randomized Clinical Trial |
| Huang, 2022 | High-Definition Transcranial Direct Current with Electrical Theta Burst on Post-Stroke Motor Rehabilitation: A Pilot Randomized Controlled Trial |
| Koh, 2017 | Effects of Transcranial Direct Current Stimulation With Sensory Modulation on Stroke Motor Rehabilitation: A Randomized Controlled Trial |
| Shaheiwola, 2018 | Using tDCS as an Add-On Treatment Prior to FES Therapy in Improving Upper Limb Function in Severe Chronic Stroke Patients: A Randomized Controlled Study |
| Zheng, 2020 | Effect of transcranial direct current stimulation combined with staging acupuncture on upper limb motor function in stroke patients with hemiplegia (in Chinese) |
| Baricich, 2008 | A single-blinded, randomized pilot study of botulinum toxin type A combined with non-pharmacological treatment for spastic foot |
| Cho, 2013 | A single trial of transcutaneous electrical nerve stimulation (TENS) improves spasticity and balance in patients with chronic stroke |
| Deshmukh, 2013 | Application of transcutaneous electrical stimulation on lower limb acupoints as an important adjunctive tool in stroke rehabilitation program & its effects on spasticity and functional ability |
| Hussain, 2013 | The effect of transcutaneous electrical nerve stimulation (TENS) combined with Bobath on post stroke spasticity. A randomized controlled study |
| Kumar, 2014 | Comparison between electrical stimulation over motor point and TENS over acupuncture point in reducing spasticity and improving function after stroke: randomized clinical trial |
| Wang, 2023 | Effects of transcutaneous electrical acupoint stimulation on upper-limb impairment after stroke: A randomized, controlled, single-blind trial |
| Feng, 2019 | Study on the efficacy of acupoint application combined with transcutaneous electrical nerve stimulation in the treatment of upper limb and hand dysfunction after stroke (in Chinese) |
| Yan, 2010 | Clinical observation of variable frequency transcutaneous electrical nerve stimulation for upper limb flexor spasm after stroke (in Chinese) |
| Hu, 2024 | Effects of high-definition tDCS targeting individual motor hotspot with EMG-driven robotic hand training on upper extremity motor function: a pilot randomized controlled trial |
| **3. Ineligible comparison** | |
| Abo, 2020 | Efficacy and Safety of OnabotulinumtoxinA 400 Units in Patients with Post-Stroke Upper Limb Spasticity: Final Report of a Randomized, Double-Blind, Placebo-Controlled Trial with an Open-Label Extension Phase |
| Cotinat, 2024 | Robotic gait training and botulinum toxin injection improve gait in the chronic post-stroke phase: A randomized controlled trial |
| Giray, 2020 | Effects of dynamic lycra orthosis as an adjunct to rehabilitation after botulinum toxin-A injection of the upper-limb in adults following stroke: A single-blinded randomized controlled pilot study |
| Hauret, 2023 | Effectiveness of Ultrasound-guided VS Electrical-stimulation-guided Botulinum Toxin Injections in Triceps Surae Spasticity after Stroke: A Randomized Controlled Study |
| Hung, 2021 | The Effects of Distributed vs. Condensed Schedule for Robot-Assisted Training with Botulinum Toxin A Injection for Spastic Upper Limbs in Chronic Post-Stroke Subjects |
| Hung, 2022 | A Pilot Randomized Controlled Trial of Botulinum Toxin Treatment Combined with Robot-Assisted Therapy, Mirror Therapy, or Active Control Treatment in Patients with Spasticity Following Stroke |
| Lannin, 2022 | Long-term effect of additional rehabilitation following botulinum toxin-A on upper limb activity in chronic stroke: the InTENSE randomised trial |
| Lannin, 2020 | Effect of Additional Rehabilitation After Botulinum Toxin-A on Upper Limb Activity in Chronic Stroke: The InTENSE Trial |
| Lee, 2022 | Safety and efficacy of MT10107 in post-stroke upper limb spasticity treatment: A phase I randomized controlled trial |
| Maulet, 2021 | Self-rehabilitation combined with botulinum toxin to improve arm function in people with chronic stroke. A randomized controlled trial |
| Munari, 2020 | Combined effects of backward treadmill training and botulinum toxin type A therapy on gait and balance in patients with chronic stroke: A pilot, single-blind, randomized controlled trial |
| Turna, 2020 | The effects of different injection techniques of botulinum toxin a in post-stroke patients with plantar flexor spasticity |
| Ye, 2023 | A Randomized, Double-Blind, Active Control, Multicenter, Phase 3 Study to Evaluate the Efficacy and Safety of Liztox(®) versus Botox(®) in Post-Stroke Upper Limb Spasticity |
| Zhang, 2021 | Improving Botulinum Toxin Efficiency in Treating Post-Stroke Spasticity Using 3D Innervation Zone Imaging |
| Li, 2020 | A synergistic rehabilitation training for lower limb muscle spasm in stroke patients (in Chinese) |
| Yuan, 2016 | Efficacy of botulinum toxin A injection combined with exercise therapy for elbow flexion spasm after stroke (in Chinese) |
| de Jong, 2013 | Combined arm stretch positioning and neuromuscular electrical stimulation during rehabilitation does not improve range of motion, shoulder pain or function in patients after stroke: a randomised trial |
| de Kroon, 2004 | Electrical stimulation of the upper limb in stroke: stimulation of the extensors of the hand vs. alternate stimulation of flexors and extensors |
| Hesse, 1995 | Short-term electrical stimulation enhances the effectiveness of Botulinum toxin in the treatment of lower limb spasticity in hemiparetic patients |
| Johnson, 2004 | The effect of combined use of botulinum toxin type A and functional electric stimulation in the treatment of spastic drop foot after stroke: a preliminary investigation |
| Kim, 2015 | Mirror therapy combined with biofeedback functional electrical stimulation for motor recovery of upper extremities after stroke: a pilot randomized controlled trial |
| Ng, 2021 | Effect of cycling and functional electrical stimulation with linear and interval patterns of timing on gait parameters in patients after stroke: a randomized clinical trial |
| Qian, 2019 | Distal versus proximal - an investigation on different supportive strategies by robots for upper limb rehabilitation after stroke: a randomized controlled trial |
| Sharif, 2017 | Effectiveness of Functional Electrical Stimulation (FES) versus Conventional Electrical Stimulation in Gait Rehabilitation of Patients with Stroke |
| Zhang Y, 2019 | Effects of transcranial direct current electrical stimulation combined with upper limb robots in upper limb motor function and surface electromyogram signal in patients with cerebral infraction during convalescence (in Chinese) |
| Cui, 2023 | Effect of transcranial direct current stimulation on upper limb dysfunction in stroke based on the bimodal balance-recovery model (in Chinese) |
| Ersoy, 2023 | Comparison of transcutaneous electrical stimulation and suprascapular nerve blockage for the treatment of hemiplegic shoulder pain |
| **4. No outcome of interest** | |
| Lindsay, 2021 | Can the early use of botulinum toxin in post stroke spasticity reduce contracture development? A randomised controlled trial |
| Ding, 2014 | The effect of botulinum toxin type A injection with ankle-foot orthosis on patients with post-stroke lower limb spasticity (in Chinese) |
| Carda, 2017 | Electrically Assisted Movement Therapy in Chronic Stroke Patients with Severe Upper Limb Paresis: A Pilot, Single-Blind, Randomized Crossover Study |
| Cheng, 2010 | Effects of combining electric stimulation with active ankle dorsiflexion while standing on a rocker board: a pilot study for subjects with spastic foot after stroke |
| Malhotra, 2013 | A randomized controlled trial of surface neuromuscular electrical stimulation applied early after acute stroke: effects on wrist pain, spasticity and contractures |
| Rosewilliam, 2012 | Can surface neuromuscular electrical stimulation of the wrist and hand combined with routine therapy facilitate recovery of arm function in patients with stroke? |
| van Bloemendaal, 2021 | Feasibility and Preliminary Efficacy of Gait Training Assisted by Multichannel Functional Electrical Stimulation in Early Stroke Rehabilitation: A Pilot Randomized Controlled Trial |
| Wang, 2016 | Full-movement neuromuscular electrical stimulation improves plantar flexor spasticity and ankle active dorsiflexion in stroke patients: a randomized controlled study |
| Yan, 2005 | Functional electrical stimulation improves motor recovery of the lower extremity and walking ability of subjects with first acute stroke: a randomized placebo-controlled trial (in Chinese) |
| You, 2014 | Functional electrical stimulation early after stroke improves lower limb motor function and ability in activities of daily living (in Chinese) |
| Chen, 2015 | Effect of Functional Electrical Stimulation on Hemiplegic Gait of Acute Stroke Patients (in Chinese) |
| Huang, 2021 | Influence of FES combined with treadmill exercise on recovery of lower limbs function in stroke patients (in Chinese) |
| Li, 2018 | Effects of Functional Electrical Stimulation Combined with Treadmill Training with Body Weight Support on Walking Ability in Patients with Stroke (in Chinese) |
| Li, 2017 | Effects of functional electrical stimulation combined with cycling active exercise of lower limbs in patients with early stroke (in Chinese) |
| Wang, 2021 | Electroacupuncture enhances the effectiveness of electrical stimulation in treating spastic foot drop (in Chinese) |
| Wu, 2012 | The curative effect of early intervention of electro-acupuncture combined with functional electric stimulation on the treatment of spastic dysarthria in acute cerebral infarction patients (in Chinese) |
| Yan, 2006 | Effects of functional electrical stimulation on the improvement of motor function of patients with stroke: a randomized controlled trial (in Chinese) |
| You, 2013 | Effectiveness of Functional Electrical Stimulation on Motor and Functional Recovery of the Lower Extremity in Subject with Early Stroke: A Randomized Controlled Trial (in Chinese) |
| You, 2007 | Effectiveness of functional electrical stimulation on functional recovery of the lower extremity in subject with early stroke: a randomized controlled trial (in Chinese) |
| Zhao, 2022 | Efficacy of low-frequency neuromuscular electrical stimulation combined with baclofen for hemiplegic muscle spasm in stroke patients (in Chinese) |
| Jiang, 2022 | A Randomized Controlled Trial of Repetitive Peripheral Magnetic Stimulation applied in Early Subacute Stroke: Effects on Severe Upper-limb Impairment |
| Ma, 2017 | Repetitive magnetic stimulation can reduce elbow flexor spasticity after stroke (in Chinese) |
| Song, 2017 | Repeated magnetic stimulation improves the effectiveness of exercise therapy in relieving ankle spasticity after stroke (in Chinese) |
| Wang, 2021 | Effect of repetitive peripheral magnetic stimulation combined with rehabilitation training on upper and lower limb spasticity and motor function in stroke patients (in Chinese) |
| Wen, 2022 | Clinical study of the influence of low-frequency rTMS combined with peripheral magnetic stimulation on lower limb dysfunction in patients with cerebral infarction (in Chinese) |
| Wu, 2021 | Clinical effect of Jin's 3-Needle combined with repetitive transcranial magnetic stimulation for spastic hemiplegia after stroke (in Chinese) |
| Yang, 2015 | Effect of rTMS on the recovery of limb function in patients with post-stroke lower limb spasm (in Chinese) |
| Beaulieu, 2019 | Efficacy, safety, and tolerability of bilateral transcranial direct current stimulation combined to a resistance training program in chronic stroke survivors: A double-blind, randomized, placebo-controlled pilot study |
| Bornheim, 2020 | Transcranial direct current stimulation associated with physical-therapy in acute stroke patients - A randomized, triple blind, sham-controlled study |
| Bradnam, 2012 | Contralesional hemisphere control of the proximal paretic upper limb following stroke |
| Cattagni, 2019 | A single session of bihemispheric transcranial direct current stimulation does not improve quadriceps muscle spasticity in people with chronic stroke |
| Liao, 2020 | Timing-dependent effects of transcranial direct current stimulation with mirror therapy on daily function and motor control in chronic stroke: a randomized controlled pilot study |
| Jung, 2017 | Effects of sit-to-stand training combined with transcutaneous electrical stimulation on spasticity, muscle strength and balance ability in patients with stroke: A randomized controlled study |
| Laddha, 2016 | Effect of Transcutaneous Electrical Nerve Stimulation on Plantar Flexor Muscle Spasticity and Walking Speed in Stroke Patients |
| Ng, 2007 | Transcutaneous electrical nerve stimulation combined with task-related training improves lower limb functions in subjects with chronic stroke |
| Yan, 2009 | Transcutaneous electrical stimulation on acupuncture points improves muscle function in subjects after acute stroke: a randomized controlled trial |
| Liu, 2011 | Effect of transcutaneous electrical nerve stimulation therapy combined with ankle dorsiflexion maneuver rehabilitation exercises on lower limb function in stroke patients (in Chinese) |
| Tong, 2018 | Comparative study of shock wave therapy combined with transcutaneous nerve electrical stimulation for the treatment of post-stroke gastrocnemius muscle spasm (in Chinese) |
| **5. Not RCT** | |
| Chen, 2020 | The Effects of Botulinum Toxin Injections on Spasticity and Motor Performance in Chronic Stroke with Spastic Hemiplegia |
| Dressler, 2015 | Long-term efficacy and safety of incobotulinumtoxinA and conventional treatment of poststroke arm spasticity: a prospective, non-interventional, open-label, parallel-group study |
| Hara, 2022 | Prognosis prediction of the effect of botulinum toxin therapy and intensive rehabilitation on the upper arm function in post-stroke patients using hierarchical cluster analysis |
| Lee, 2022 | Safety and Efficacy of HU-014 in the Treatment of Post-Stroke Upper Limb Spasticity: A Phase I Pilot Study |
| Milte, 2020 | Protocol for the economic evaluation of the InTENSE program for rehabilitation of chronic upper limb spasticity |
| Wei, 2017 | The therapeutic effect of botox local injection combined with rehabilitation training on hemiplegia spastic foot drop after stroke (in Chinese) |
| Liu, 2023 | Effect of low-frequency neuromuscular electrical stimulation on hemiplegic muscle spasm after stroke (in Chinese) |
| Xing, 2021 | Effect of electrical stimulation combined with movement intervention on hemiplegic hypermyotonia (in Chinese) |
| Zhao, 2020 | Application of botulinum toxin type A in the treatment of lower limb muscle spasms after stroke (in Chinese) |
| Chen, 2020 | Electroencephalography Mu Rhythm Changes and Decreased Spasticity After Repetitive Peripheral Magnetic Stimulation in Patients Following Stroke |
| Kinoshita, 2020 | Repetitive peripheral magnetic stimulation combined with intensive physical therapy for gait disturbance after hemorrhagic stroke: an open-label case series |
| Kinoshita, 2020 | Dose-response of rPMS for upper Limb hemiparesis after stroke |
| Zifko, 2002 | Repetitive peripheral magnetic stimulation is effective in the rehabilitation of the paretic arm |
| Málly, 2008 | Recovery of motor disability and spasticity in post-stroke after repetitive transcranial magnetic stimulation (rTMS) |
| Chen, 2017 | The effect of transcranial direct current stimulation combined with task-oriented training on upper limb and hand dysfunction in patients with chronic stroke (in Chinese) |
| Palmcrantz, 2020 | Feasibility and potential effects of using the electro-dress Mollii on spasticity and functioning in chronic stroke |
| **6. Secondary analysis** | |
| Lindsay, 2023 | Estimating the cost consequence of the early use of botulinum toxin in post-stroke spasticity: Secondary analysis of a randomised controlled trial |
| **7. non-peer-reviewed articles** | |
| Yan, 2002 | The effectiveness of early neuromuscular electrical stimulation on lower extremity functions of stroke patient (in Chinese) |
| Nuer, 2016 | Study on the effects of transcranial direct current stimulation and functional electrical stimulation on upper limb rehabilitation in stroke patients (in Chinese) |
| Zhang, 2019 | Mechanism study of clinical application of transcranial direct current stimulation in upper limb spasticity after stroke (in Chinese) |
| **8. Retracted article** | |
| Xu, 2021 | Comparative Analysis of the Effect of Low-Frequency Repeated Transcranial Magnetic Stimulation and Extracorporeal Shock Wave on Improving the Spasm of Flexor after Stroke |

# Appendix 7. Detailed table of included studies.

| First Author, Year | Study Arm | Participants | | | | Intervention | | | | Main outcome measures | Follow-up | Adverse events |
| --- | --- | --- | --- | --- | --- | --- | --- | --- | --- | --- | --- | --- |
|  |  | *N* (Male /Female) | Age (Years) [Mean (SD)/Median (Q1, Q3)] | Type of Stroke (ischemic/ hemorrhagic/ embolic) | Disease Duration [Mean (SD)/Median (Q1, Q3)] | Parameters | Application site | Protocol | Co-  intervention |  |  |  |
| Abramovich, 2020 | G1: Control | 24 | 60.51(4.9) | All ischemic strokes | 3.2 (0.5) and  4.6 (0.3) months in 45.8% and 54.2% of the patients | No injection/TENS | N/A | N/A | Basic therapy | MAS  RMA  ROM | Post-treatment | NR |
|  | G2: BoNTA | 24 |  |  |  | At a dose of 200 U (incobotulotoxinA) in moderate spasticity and at a dose of 300-400 U in severe spasticity | Upper limb (Forearm and shoulder) | A single injection of under ultrasound control | Basic therapy |  |  |  |
|  | G3: TENS | 24 |  |  |  | 77 Hz, mean exposure intensity of 40 conventional units | Upper limb (forearm  and shoulder) | 5 min for each area, 60 min in total, 20 sessions | Basic therapy |  |  |  |
| Bakheit, 2001 | G1: Control | 15/17 | 67.0 (11.1) | 15/3/11 | NR | 1000 U of placebo were injected into five muscles of the affected arm | Upper limb (Biceps brachii and forearm) | Dysport was injected into two sites of biceps brachii and one site of the forearm using landmarks. | None | MAS*, Pain, BI,  Goal attainment scale,  ORS | 4, 8, 12, and 16 weeks after treatment | 20 in the placebo group and 16 in the BoNTA group, but no fatal, life-threatening adverse events were recorded. |
|  | G2: BoNTA | 11/16 | 64.1 (13.2) | 14/8/2 | NR | 1000 U of Dysport were injected into five muscles of the affected arm |  |  | None |  |  |  |
| Bakheit, 2000 | G1: Control | 12/7 | 63.6 (14.1) | 8/3/4 | NR | Identical volume of placebo | Upper limb (Biceps brachii and forearm) | The injections were  placed in the motor endplate zone with the use of anatomic landmarks. | None | MAS, ROM, pain, RMA, BI, and the difficulties encountered  in performing 3 functional activities | 2, 4, 8, 12, and 16 weeks after treatment | No fatal, life threatening adverse  events were recorded.  No statistically significant differences between  the study groups. |
|  | G2: BoNTA | 39/24 | 63.2 (13.0) | 36/12/10 | NR | BoNTA (Dysport):  - 500 U: biceps brachii 200U, FDP 75, FDS 75, FCU 75, FCR 75  - 1000 U: biceps brachii 400U, FDP 150, FDS 150, FCU 150, FCR 150  - 1500 U: biceps brachii 600U, FDP 225, FDS 225, FCU 225, FCR 225 |  |  | None |  |  |  |
| Childers, 2004 | G1: Control | 13/13 | 60.3 (33.8, 76.0) | NR | 26.6 (2.1, 211.7) m | Identical volume of placebo | Upper limb (Biceps brachii and forearm) | All injections were given under electromyographic guidance. | Concurrent therapies could implement except during the first week after injection. | MAS*, Disability Scale, pain, FIM,  SF-36, Global assessment of  response to treatment | Every week for the first 6 weeks and then after 9, 12, 18, and 24 weeks in the study. | Serious adverse events were reported in 15 patients during the study, but none were considered treatment-related. |
|  | G2: BoNTA | 48/17 | 59.3 (30.4, 76.1)  61.1 (39.6, 79.4)  59.0 (35.4, 77.7) | NR | 28.7 (0.9, 108.5) m  31.2 (1.2, 226.9) m  16.5 (2.6, 99.2) m | BoNTA (Botox):  -90 U: biceps brachii 50U, FCR 15U, FCU 10U, FDP 7.5U, FDS 7.5U  -180 U: biceps brachii 100U, FCR 30U, FCU 20U, FDP 15U, FDS 15U  -360 U: biceps brachii 200U, FCR 60U, FCU 40U, FDP 30U, FDS 30U |  |  |  |  |  |  |
| Rocha, 2023 | G1: Control | 8/4 | 61 (12.1) | 11/1 | 19.4 (22.9) | Identical volume of placebo | Upper limb (pectoralis major and subscapularis  Muscles) | All injections were given under electrostimulation guidance. | None | VAS, ROM, MAS, McGill questionnaire, FMA-UE | 1 and 4 months after treatment | NR |
|  | G2: BoNTA | 8/4 | 56.5 (10.2) | 11/1 | 26.1 (36.8) | 200 U of abobotulinum at two different points per muscle with the same dosage (100U per point) |  |  | None |  |  |  |
| Hesse, 2012 | G1: Control | 6/3 | 66 (11) | 7/2 | 5.6 (1.1) w | No injection | Upper limb (finger and wrist) | One injection site per muscle under electrostimulation guidance. | Comprehensive rehabilitation | MAS*, REPAS, FMA-UE, Disability scale | 1 and 6 months after injection | NR |
|  | G2: BoNTA | 6/3 | 57 (11) | 6/3 | 5.8 (1.3) w | 150 U BoNTA (Xeomin):  deep and superficial finger (100 units) and wrist flexors (50 units) |  |  |  |  |  |  |
| Hesse, 1998 | G1: Control | 6 | 42.7 (38, 61) | All ischemic strokes | 7.45 (6, 11) m | Identical volume of placebo | Upper limb (Biceps brachii, brachialis, and forearm) | All injections were given under electromyographic guidance. | Physiotherapy | MAS, Limb position at rest, ORS | 2. 6, 12 weeks after treatment. | No study-related side-effects were observed. |
|  | G2: BoNTA | 6 | 54.3 (32, 73) |  |  | 1000 U Dysport: Mm. biceps brachii, brachialis (each 250 U)  FCU, FCR, FDP, and FDS (each 125 U)  Two sites per muscle. |  |  |  |  |  |  |
|  | G3: NMES | 6 | 53.6 (39-64) |  |  | 20 Hz, 200 µs, 50–90 mA | Arm (Mm. biceps and triceps) and forearm (wrist and finger flexors and extensors) | Half an hour, three times per day during the three days after the injection. |  |  |  |  |
| Hokazono, 2022 | G1: Control | 14/6 | 66 (41, 80) | 6/14 | 32.5 (7, 157) m | No injection | N/A | N/A | Repetitive facilitative exercise | FMA-UE*, ARAT, BBT, and MAS | Post-treatment | NR |
|  | G2: BoNTA | 11/9 | 60.5 (19, 75) | 7/13 | 40.5 (12, 209) m | Onabotulinumtoxin A: (Botox) maximum dose, 240 U | Upper-limb (muscles whose hypertonicity disturbed the patient’s intended movements and ADL.) | Injections were given under electromyographic guidance. |  |  |  |  |
| Jahangir, 2007 | G1: Control | 15/10 | 60.48 (11.6) | 22/3 | 40.36 (44.5) m | Identical volume of placebo | Upper limb (FCR, FCU, FDS, and FDP) | NR | Physiotherapy | MAS, BI, and EQ-5D | 1 and 3 months after treatment | No adverse events reported |
|  | G2: BoNTA | 18/9 | 61.08 (10.9) | 27/0 | 49.70 (35.5) m | BoNTA (Botox):  20 U for each muscle |  |  |  |  |  |  |
| Kaji, 2010 | G1: Control | 16/46 | 62.5 (9.3) | NR | 72.0 (60.3) m | Identical volume of placebo | Lower limb (medial head of gastrocnemius,  lateral head of gastrocnemius, soleus, and tibialis posterior) | A single injection under electrostimulation guidance. | None | MAS*, Gait pattern scale, gait speed, clinical global impression | 1, 4, 6, 8, and  12 weeks after treatment | Investigator-determined treatment-related adverse  events were reported  in 12% in the BoNTA group and 11% in the  placebo group. |
|  | G2: BoNTA | 8/50 | 62.4 (8.7) | NR | 80.8 (72.8) m | 300 U BoNTA (Xeomin):  75 U for each muscle, divided into three sites per muscle |  |  | None |  |  |  |
| Kaji, 2010 (CMRO) | G1: Control | 19/18 | 63.2 (10.5) | NR | 79.2 (61.0) m | Identical volume of placebo | Upper limb (wrist, finger, thumb) | A single injection under electrostimulation guidance. | None | MAS*, disability assessment scale, clinical global impression | 0, 1, 4, 6, 8, and  12 weeks after treatment | No clinically relevant changes were reported. |
|  | G2: BoNTA | 55/17 | 63.3 (9.4) | NR | 84.9 (77.8) m | BoNTA (Botox)  -High dose (200 U): 50 U FCR, 50 U FCU, 50 U FDP, 50 U FDS +/- 20 U FPL, 20 U adductor pollicis (if thumb spasticity MAS≥2)  -Low dose (120 U): 30 U FCR, 30 U FCU, 30 U FDP, 30 U FDS +/- 15 U FPL, 15 U adductor pollicis (if thumb spasticity MAS≥2) |  |  | None |  |  |  |
| Kerzoncuf, 2020 | G1: Control | 12/14 | 50.69 (12.94) | 14/12 | 71.14 (67.05) m | Identical volume of placebo | Lower limb (soleus, gastrocnemius, tibialis posterior) | Injection was given under electrostimulation guidance. | Any rehabilitation procedures, antispastic drugs, and orthoses was continued. | Quantified balance assessment*, MAS, occurrence of a clonus, ROM, proprioceptive and dis- criminative sensitivity, occurrence of falls, walking speed, functional ambulation classification, FIM | 4-6 weeks after treatment | NR |
|  | G2: BoNTA | 12/11 | 53.43 (14.76) | 10/13 | 50.04 (28.67) m | BoNTA (Botox) max dose 300 U:  50-160 U soleus, 50-100 U gastrocnemius, tibialis posterior and FDL, 25-50U FHL and 25 U EHL |  |  |  |  |  |  |
| Marciniak, 2012 | G1: Control | 6/4 | 59.8 (10.3) | NR | NR | Identical volume of saline | Upper limb (shoulder) | Electromyographic guidance was used for the injections. | None | VAS*, MAS, disability assessment scale, FMA, ROM, McGill Pain Questionnaire short Form, FIM (hygiene) | 2, 4, and 12 weeks after the injections | Ten adverse events were reported in seven subjects. |
|  | G2: BoNTA | 7/4 | 60.2 (7.8) | NR | NR | BoNTA (Botox) mean 188 U (range 140-200 U):  100-150 U pectoralis major +/- 40-60 U teres major |  |  | None |  |  |  |
| McCrory, 2009 | G1: Control | 26/16 | 58.4 (14.6) | NR | 6.6 (12.6) years | Identical volume of placebo | Upper limb (elbow, wrist and finger joints) | Electromyographic guidance was used for the injections.  Patients received re-treatment with the same agent at week 12 (500–1000 U). | None | AQoL*, Pain, Hospital Anxiety and Depression  Rating Scale, Goal Attainment Scaling, MAS, Modified Motor Assessment Scale, Carer Burden scale, Patient disability scale, Global  Assessment of Benefit | 8,12,20, and 24 weeks post-injection | 67% of the BoNTA and 62% of the placebo group experienced at  least one adverse event, which were generally mild. These  were considered treatment-related in 5.5% of BoNTA-treated patients and 9.5% placebo-treated patients. |
|  | G2: BoNTA | 32/22 | 59.7 (12.2) | NR | 5.3 (8.7) years | BoNTA (Dysport) 750-1000U to distal upper limb |  |  | None |  |  |  |
| Picelli, 2014 | G1: BoNTA | 8/2 | 65.2 (5.5) | NR | 4.6 (1.3) y | BoNTA (Botox) 200 U:  100 U for the gastrocnemius medialis  100 U for the gastrocnemius lateralis | Lower limb (Gastrocnemius muscle origin and the mid-belly of the gastrocnemius bulk) | B-mode real-time ultrasonography guidance was used for the injections. | Daily 30-min  sessions of stretching exercises | MAS and ROM | 15, 30, and 90 days after the  first clinical evaluation | NR |
|  | G2: TENS | 5/5 | 62.7 (12.9) | NR | 3.9 (1.6) y | 100 Hz, 0.3 ms, 50 mA that would not cause contraction |  | 15 min daily, 5 days a week for 2 consecutive weeks |  |  |  |  |
| Prazeres, 2018 | G1: Control | 12/8 | 52.05 (12.51) | 19/1 | 32.05 (14.89) m | Identical volume of saline | Upper limb (wrist, elbow) | NR | Physiotherapy | Time up and go test*, 6-minute walking test*, FMA*, MAS | 3, 6, and 9 months | NR |
|  | G2: BoNTA | 12/8 | 52.5 (11.01) | 16/4 | 34.15 (21.43) m | BoNTA (Dysport): lack of detail information |  | NR | Physiotherapy |  |  |  |
| Rosales, 2018 | G1: Control | 10/4 | 56.5 (9.7) | 10/4 | 6.52 (2.53) w | Identical volume of placebo | Upper limb | Doses were administered per muscle according to investigators’ judgements. | Most patients participated in occupational and physiotherapy practices. | Time to re-injection, MAS, motor recovery score, Global assessment | 4,6,8,10 and 12 weeks | No study-related side-effects were observed. |
|  | G2: BoNTA | 23/5 | 61.5 (13.2) | 20/8 | 6.18 (2.87) w | BoNTA (Dysport): 500 U in targeted muscle of upper limb |  |  |  |  |  |  |
| Shaw, 2010 | G1: Control | 115/47 | 66 (59.8, 72.3) | 131/24 | 280 (148.8, 1145.8) d | No injection | N/A |  | Physiotherapy | Action Research Arm Test*, MAS, Motricity Index, MVG, Nine Hole Peg Test, ORS, BI, pain | 1, 3 and 12 months | There were no significant differences in the number and type of serious adverse events between groups. |
|  | G2: BoNTA | 110/60 | 67 (58.8, 74) | 140/27 | 324 (128.5, 1387.5) d | BoNTA (Dysport) median dose 200 units (range 100-300 U) | Upper limb (elbow) | Injections delivered were according to the spasticity pattern using landmarks. | Physiotherapy |  |  |  |
| Tan, 2021 | G1: Control | 12/6 | 53.9 (13.0) | 11/7 | 5.7 (3.3) m | Identical volume of saline | Upper limb (shoulder) | Ultrasonography guidance was used for the injections. | Exercise + physiotherapy | VAS*, MAS, ROM, FMA-UE, and SSQoL | 1, 4, 12, and 24 weeks after treatment. | No BoNTA injection–related adverse events were found. |
|  | G2: BoNTA | 15/3 | 51.1 (11.4) | 6/12 | 5.3 (3.2) m | BoNTA (Hengli) 100 U: 2 points with each injection point receiving 50 U |  |  | Exercise + physiotherapy |  |  |  |
| Wolf, 2012 | G1: Control | 8/5 | 49.8 (13.7) | NR | NR | Identical volume of saline | Upper limb (wrist and finger) | NR | Exercise | Wolf Motor Function Test*, Stroke Impact Scale, MAS, ROM | 1,2 and 3 months | A BTX-A patient  showed swelling and a localized hematoma after injections. |
|  | G2: BoNTA | 7/5 | 48.8 (15.6) | NR | NR | BoNTA (Botox) max dose 300 U over wrist and finger muscles |  | NR | Exercise |  |  |  |
| Chen, 2003 | G1: Control | 22/18 | 64 (4) | 23/17 | 10~180 d | No injection | N/A | NR | Medication and exercise therapy | MAS, NIHSS, FMA, BI | Post-treatment | No adverse events were observed. |
|  | G2: BoNTA | 23/19 | 63 (5) | 24/18 |  | BoNTA (CBTX-A): 50~100 U for each muscle, maximum dose 400 U | Upper limb and lower limb | NR | Medication and exercise therapy |  |  |  |
| Chen, 2018 | G1: Control | 7/9 | 60.50 (6.71) | NR | 18.37 (3.36) w | No injection | N/A | Ultrasonography and electrostimulation guidance were used for the injections. | Physiotherapy | MAS  FMA-UE | 2 and 4 weeks after treatment | NR |
|  | G2: BoNTA | 7/8 | 56.80 (6.41) | NR | 15.93 (2.66) w | BoNTA (CBTX-A): 45~100 U for each muscle, maximum dose 400 U | Upper limb (shoulder, forearm, and hand) |  | Physiotherapy |  |  |  |
| Cheng, 2010 | G1: Control | 8 | NR | NR | NR | No injection | N/A | N/A | Rehabilitation | MAS  BI | Immediate, 1, 2, 6, 12 weeks after treatment | NR |
|  | G2: BoNTA | 8 | NR | NR | NR | BoNTA (CBTX-A): 5~10 U for each site, 10~15 U each muscle group, maximum dose 600 U | Upper limb (Biceps brachii and forearm) | A single injection using landmarks. | Rehabilitation |  |  |  |
| Cheng, 2016 | G1: Control | 20/11 | 54.1 (6.8) | 14/17 | 173.8 (26.5) d | No injection | N/A | N/A | Bobath | ROM, MAS, walking velocity, step length, and stride width | 1, 2, and 4 weeks after treatment | NR |
|  | G2: BoNTA | 18/14 | 56.2 (5.2) | 16/16 | 195.2 (18.7) d | BoNTA: maximum dose less than 300 U | Lower limb (Adductor femoral muscle, posterior tibialis muscle, gastrocnemius muscle, and Achilles tendon) | Ultrasonography guidance was used for the injections. | Bobath |  |  |  |
| Cui, 2006 | G1: Control | 18/4 | 58.9 (13.4) | 13/9 | 7.38 (2.05) m | No injection | N/A | N/A | Bobath | ROM, MAS, FMA-UE, and FIM-UE | 2, 4, 8, and 12 weeks after treatment | No systemic adverse events were observed. |
|  | G2: BoNTA | 19/2 | 46.1 (15.2) | 12/9 | 7.57 (2.74) m | BoNTA (CBTX-A): Biceps brachii 100 U |  | Electrostimulation guidance was used for the injections. | Bobath |  |  |  |
| Cui, 2019 | G1: Control | 44/31 | 60.2 (6.5) | 60/15 | 42.1 (7.9) d | No injection | N/A | N/A | Usual care +  rehabilitation | MAS, BBS, FMA, BI | 1, 3, and 5 months after treatment | Ten adverse events were reported in BoNT group, but all were settled. |
|  | G2: BoNTA | 45/30 | 59.3 (7.3) | 62/13 | 42.30 (7.60) d | BoNTA: 50~100 U for each muscle, mean dose 380 (48) U | Upper limb and lower limb | Injection was given using landmarks. | Usual care +  rehabilitation |  |  |  |
| Dou, 2019 | G1: Control | 12/8 | 61.18 (3.87) | NR | NR | No injection | N/A | N/A | Rehabilitation | FMA-UE  MAS | Post-treatment | NR |
|  | G2: BoNTA | 11/9 | 61.24 (3.92) | NR | NR | BoNTA (CBTX-A): 5~10 U for each site, maximum dose 600 U | Upper limb (Biceps brachii and forearm) | NR | Rehabilitation |  |  |  |
| Fu, 2005 | G1: Control | 30 | 56.00 (4.9) | 20/10 | 2.70 (1.21) years | Identical volume of saline | Upper limb and lower limb | Re-injection was administrated if the improvement of elbow flexion stiffness or spasticity of biceps brachii was not satisfied. | Medication + physiotherapy | MAS, muscle strength, and immunologic function | Post-treatment | No adverse events were observed. |
|  | G2: BoNTA | 30 | 58.24 (5.28) | 20/10 | 2.23 (1.59) years | BoNTA (CBTX-A): 5~10 U for each site, 2~6 sites for each muscle, maximum dose less than 200 U |  |  | Medication + physiotherapy |  |  |  |
| Hao, 2022 | G1: Control | 16/14 | 60.9 (5.6) | 19/11 | 1.66 (0.51) m | No injection or LFrTMS | N/A | N/A | Rehabilitation | NIHSS, MAS, FMA-UE, BI, MEP, and CMCT | Immediate and 4 weeks after treatment | No adverse events were observed. |
|  | G2: BoNTA | 15/15 | 59.2 (6.7) | 21/9 | 1.52 (0.88) m | BoNTA (CBTX-A): 5~10 U for each site, 2~4 sites for each muscle, maximum dose less than 300 U | Upper limb (Biceps brachii and forearm) | Electromyographic guidance was used for the injections. | Rehabilitation |  |  |  |
|  | G3: LFrTMS | 14/16 | 58.9 (7.3) | 18/12 | 1.55 (0.69) m | 1 Hz, 5T, 90% RMT, 1200 pulses | M1 of unaffected hemisphere | 20 min each time, once daily for 6 d weekly for 4 weeks | Rehabilitation |  |  |  |
| Hong, 2018 | G1: Control | 14/6 | 58.57 (7.97) | 12/8 | 43.21 (7.12) d | No injection | N/A | N/A | Medication + physiotherapy | MAS, ROM, FMA-UE, BI | Post-treatment | NR |
|  | G2: BoNTA | 24/16 | 57.98 (9.35) | 26/17 | 43.1 (9.06) d | BoNTA (CBTX-A): maximum dose less than 600 U | Upper limb (Biceps brachii and forearm) | Electromyographic guidance and landmarks were used for the injections. | Medication + physiotherapy |  |  |  |
| Hu, 2014 | G1: Control | 22/20 | 59.98 (4.95) | All ischemic strokes | 96.67 (35.78) d | Identical volume of saline | Upper limb and lower limb | Injection was given using landmarks. | Motor function training | MAS, FMA | 1 week, 6 weeks, and 3 months after treatment | No adverse events were observed. |
|  | G2: BoNTA | 24/21 | 62.45 (5.67) | All ischemic strokes | 104.56 (27.65) d | BoNTA (CBTX-A): 5~10 U for each site, 10~15 sites for each muscle group, maximum dose less than 500 U |  |  | Motor function training |  |  |  |
| Huang, 2018 | G1: Control | 24/16 | 45.8 (6.5) | 23/17 | 2.8 (1.5) m | No injection | N/A | N/A | Rehabilitation | MAS, FMA-UE, BI, SAS, and SDS | Post-treatment | NR |
|  | G2: BoNTA | 49/31 | 45.7 (6.0) | 46/34 | 2.9 (1.2) m | BoNTA (CBTX-A): maximum dose 50 U for each site, 10~15 sites for each muscle group, maximum dose 500 U | Upper limb (Biceps brachii and forearm) | A single injection using landmarks and electrostimulation. | Rehabilitation |  |  |  |
| Jiang, 2016 | G1: Control | 23/12 | 59.51 (4.75) | 24/11 | NR | No injection | N/A | N/A | Rehabilitation | FMA-UE, MAS, BI, and VAS | Post-treatment | NR |
|  | G2: BoNTA | 21/14 | 57.82 (4.26) | 25/10 | NR | BoNTA (CBTX-A) | Upper limb (Biceps brachii and forearm) | Ultrasonography guidance was used for the injections. | Rehabilitation |  |  |  |
| Lan, 2007 | G1: Control | 10/6 | 55.07 (10.17) | 10/6 | NR | No injection | N/A | N/A | Rehabilitation | MAS, FMA-UE, BI | 1, 2, 6, and 12 weeks after treatment | No adverse events were observed. |
|  | G2: BoNTA | 8/8 | 56.15 (7.29) | 9/7 | NR | BoNTA (CBTX-A): 5~10 U for each site, 10~15 sites for each muscle group, maximum dose less than 600 U | Upper limb (Biceps brachii and forearm) | Injection was given using landmarks. | Rehabilitation |  |  |  |
| Lan, 2007 (CJRM) | G1: Control | 16 | 55.07 (8.07) | 19/13 | NR | No injection | N/A | N/A | Rehabilitation | MAS  SIS | 1, 2, 6, and 12 weeks after treatment | No adverse events were observed. |
|  | G2: BoNTA | 16 |  |  | NR | BoNTA (CBTX-A): 5~10 U for each site, 10~15 sites for each muscle group, maximum dose less than 600 U | Upper limb (Biceps brachii and forearm) | Injection was given using landmarks. | Rehabilitation |  |  |  |
| Le, 2012 | G1: Control | 20 | 58 (6.1) | NR | NR | No injection | N/A | N/A | Rehabilitation | MAS  FMA-UE | 2, 4, 12 weeks after treatment | No adverse events were observed. |
|  | G2: BoNTA | 34 |  | NR | NR | BoNTA (CBTX-A): 2.5~5 U for each site, maximum dose less than 300 U | Upper limb (Biceps brachii and forearm) | Ultrasonography guidance was used for the injections. | Rehabilitation |  |  |  |
| Li, 2017 | G1: Control | 20/11 | 54.9 (2.7) | 20/11 | 36.9 (4.2) d | No injection | N/A | N/A | Acupuncture + Rehabilitation | FMA-UE  MAS  BI | Post-treatment | NR |
|  | G2: BoNTA | 19/12 | 54.6 (2.5) | 21/10 | 35.2 (4.6) d | BoNTA (CBTX-A): 12.5~16.5 U for each site, 6~8 sites in total | Upper limb (Biceps brachii) | Injection was given using landmarks. | Acupuncture + Rehabilitation |  |  |  |
| Li, 2009 | G1: Control | 25/5 | 36.05 (12.39) | NR | 11.12 (0.6) m | No injection | N/A | N/A | MRP, Bobath, Brunnstrom | MAS  ROM | 1 month after treatment | NR |
|  | G2: BoNTA | 22/6 | 36.85 (12.08) | NR | 11.15 (0.4) m | BoNTA (CBTX-A): 50~100 U and 2~4 sites for each muscle | Lower limb (Post calf muscle group) | Electromyographic guidance was used for the injections. | MRP, Bobath, Brunnstrom |  |  |  |
| Li, 2018 (PJCCPVD) | G1: Control | 22/18 | 65.3 (2.1) | NR | Less than 6 m | No injection | N/A | N/A | Rehabilitation | MAS | Post-treatment | NR |
|  | G2: BoNTA | 20/20 | 65.9 (2.2) | NR | Less than 6 m | BoNTA (CBTX-A): 3~5 muscles for each time, 4 or 6 sites for each muscle group, maximum dose less than 200 U | Upper limb (Biceps brachii and forearm) | NR | Rehabilitation |  |  |  |
| Li, 2019 | G1: Control | 10/2 | 63 (48-78) | 10/2 | NR | No injection | N/A | N/A | Exercise | MAS  FMA | 1 week, 1, 2, and 3 months after treatment | One case experiencing local injection site soreness, which disappeared automatically after 3 days. |
|  | G2: BoNTA | 5/7 | 62 (45~82) | 8/4 | NR | BoNTA (CBTX-A): 50~100 U for each muscle, maximum dose less than 300 U | Upper limb and lower limb | NR | Exercise |  |  |  |
| Liu, 2006 | G1: Control | 31/20 | 63 (6) | 45/6 | 3.2 (1.5) m | No injection | N/A | N/A | Rehabilitation | MAS  FMA-UE | 2, 4, and 12 weeks after treatment | NR |
|  | G2: BoNTA | 29/24 | 65 (7) | 40/13 | 3.6 (1.7) m | BoNTA (CBTX-A): 5~10 U for each site, 4~6 sites for each muscle, maximum dose 200 U | Upper limb (Biceps brachii and forearm) | Injection was given using landmarks. | Rehabilitation |  |  |  |
| Luo, 2009 | G1: Control | 43/20 | 50.4 (6.1) | 55/8 | 42.8 (9.7) d | No injection | N/A | N/A | Rehabilitation | MAS  FMA-LE  BI  BBS | 1, 3, and 5 months after treatment | Ten adverse events were reported in BoNTA group. |
|  | G2: BoNTA | 42/21 | 51.2 (5.3) | 54/9 | 41.8 (8.9) d | BoNTA (CBTX-A): 73 (5.8) U for each muscle, 3~7 muscles in total, 380 (47) U per person | Lower limb | Landmarks were utilised. | Rehabilitation |  |  |  |
| Ma, 2019 | G1: Control | 45/19 | 66.0 (7.8) | 51/13 | 3 months to 2 years | No injection | N/A | N/A | Rehabilitation | MAS  FMA  10MWT  Wear of ankle-foot orthosis | Post-treatment | NR |
|  | G2: BoNTA | 48/16 | 63.0 (6.2) | 47/17 |  | BoNTA (CBTX-A): maximum dose less than 600 U | Lower limb | Ultrasonography guidance was used for the injections. | Rehabilitation |  |  |  |
| Meng, 2008 | G1: Control. | 27 | 47.5 (14.3) | 30/24 | 7.8 (1.2) m | No injection | N/A | N/A | Rehabilitation | MAS  FMA-UE  BI | 2, 4, and 12 weeks after treatment | NR |
|  | G2: BoNTA | 27 |  |  |  | BoNTA (CBTX-A): 25 U for each site, 4 sites in total | Upper limb (Biceps brachii) | Landmarks were utilised. | Rehabilitation |  |  |  |
| Pan, 2016 | G1: Control | 25/15 | 48.3 (5.4) | 28/12 | 35.5 (7.4) d | No injection | N/A | N/A | Acupuncture + rehabilitation | MAS  FMA-UE  BI | 2, 6, and 12 weeks after treatment | Some participants experiencing local injection site pain, 3 patients had cold-like symptoms, and the other patients had no obvious adverse reactions. |
|  | G2: BoNTA | 27/13 | 49.7 (6.1) | 27/13 | 35.8 (6.9) d | BoNTA: 25~75 U for each site, 4~6 sites for each muscle, 1~5 muscles in total, maximum dose less than 400 U | Upper limb (Biceps brachii and forearm) | Landmarks were utilised. | Acupuncture + rehabilitation |  |  |  |
| Peng, 2014 | G1: Control | 22/14 | 58.5 (5.2) | NR | NR | No injection | N/A | N/A | Message + rehabilitation | MAS  UEFT | 2, 4, and 8 weeks after treatment | NR |
|  | G2: BoNTA | 20/16 | 59.3 (4.8) | NR | NR | BoNTA (BOTOX): 12~16 U for each size, 6~8 sizes in total, maximum dose 200 U | Upper limb (Biceps brachii) | Landmarks were utilised. | None |  |  |  |
| Peng, 2022 | G1: Control | 11/9 | 58.80 (2.12) | NR | 5.59 (0.14) m | No injection | N/A | N/A | Rehabilitation | MAS  BI  iEMG | 2 and 4 weeks after treatment | No adverse events were observed. |
|  | G2: BoNTA | 22/18 | 58.65 (2.11) | NR | 5.63 (0.11) m | BoNTA (CBTX-A): less than 50 U for each size, maximum dose 600 U | Upper limb and lower limb | Landmarks and ultrasonography were utilised. | Rehabilitation |  |  |  |
| Qu, 2017 | G1: Control | 26/16 | 49.38 (13.11) | 27/15 | 55.64 (18.50) d | No injection | N/A | N/A | Usual care + electromyographic biofeedback therapy | MAS  FMA-UE  ROM  BI | 2 and 4 weeks after treatment | NR |
|  | G2: BoNTA | 27/15 | 50.13 (13.68) | 25/17 | 56.27 (19.58) d | BoNTA (CBTX-A): 25~75 U for each site, 4~6 sites for each muscle, 1~5 muscles in total, maximum dose less than 400 U | Upper limb | Landmarks were utilised. | Usual care+ electromyographic biofeedback therapy |  |  |  |
| Ren, 2008 | G1: Control | 10/8 | 53.1 (9.3) | NR | 2.5 m | No injection | N/A | N/A | Rehabilitation | MAS  GMFM-D  GMFM-E | 2 weeks, 1 month, and 3 months | NR |
|  | G2: BoNTA | 9/8 | 55.1 (11.5) | NR |  | BoNTA (CBTX-A): 50~100 U for each muscle, maximum dose less than 400 U | Lower limb | Landmarks were utilised. | Rehabilitation |  |  |  |
| Shan, 2016 | G1: Control | 32/18 | 50.2 (8.3) | NR | 41.8 (17.7) d | No injection | N/A | N/A | Rehabilitation | MAS  FMA  BI  Footprint analysis | 2, 6, and 12 weeks after treatment | Six mild adverse events were reported in BoNTA group, no severe adverse events were observed. |
|  | G2: BoNTA | 31/19 | 49.1 (7.7) | NR | 42.9 (18.5) d | BoNTA (BOTOX): maximum dose 500 U | Upper limb and lower limb | Electromyographic guidance was used for the injections. | Rehabilitation |  |  |  |
| Su, 2021 | G1: Control | 28/17 | 62.26 (3.65) | 25/20 | NR | No injection | N/A | N/A | Rehabilitation | MAS  10MWT  FMA-LE  BBS  Wear of orthosis | Post-treatment | NR |
|  | G2: BoNTA | 30/15 | 62.34 (3.75) | 26/19 | NR | BoNTA (CBTX-A): maximum dose 600 U | Lower limb | Ultrasonography guidance was used for the injections. | Rehabilitation |  |  |  |
| Sun, 2023 | G1: Control | 23/9 | 55.46 (7.13) | NR | 43.21 (5.65) d | No injection | N/A | N/A | Rehabilitation | MAS  FMA-LE  10MWT  FAC  BI | Post-treatment | NR |
|  | G2: BoNTA | 21/11 | 52.09 (3.57) | NR | 40.21 (5.50) d | BoNTA (CBTX-A): 30~50 U for each site, 1~2 sites for each muscle, maximum dose 500 U | Lower limb | Ultrasonography guidance was used for the injections. | Rehabilitation |  |  |  |
| Tao, 2018 | G1: Control | 12/12 | 57.33 (12.00) | NR | 3.58 (2.44) d | No injection or LFrTMS | N/A | N/A | Rehabilitation | MAS  FMA-LE  BI  BBS | 1, 6, and 12 months after treatment | Four patients in BoNTA group experienced muscle pain and fatigue. |
|  | G2: BoNTA | 11/13 | 55.93 (13.88) | NR | 4.33 (2.57) d | 1 Hz, 90% RMT, 1200 pulses | M1 of the unaffected hemisphere | 15 min daily | Rehabilitation |  |  |  |
|  | G3: LFrTMS | 10/14 | 56.55 (13.11) | NR | 4.01 (2.89) d | BoNTA (BOTOX): 5~10 U for each site, 10~15 sites for each muscle, maximum dose 300~400 U |  | Landmarks were utilised.  Re-injection was administrated at the same dose 3 months later. | Rehabilitation |  |  |  |
| Wang (ZJCMJ), 2016 | G1: Control | 30/10 | 54.05 (14.30) | NR | NR | No injection | N/A | N/A | Rehabilitation | MAS, Strength, BI, Motor assessment scale, BBS, and ROM | Post-treatment | NR |
|  | G2: BoNTA | 24/16 | 49.67 (15.02) | NR | NR | BoNTA (BOTOX): 5~10 U for each site, 20~50 U and 4~5 sites for each muscle, 2~4 muscles in total, maximum dose 200 U | Lower limb | Electromyographic guidance was used for the injections. | Rehabilitation |  |  |  |
| Wang, 2016 | G1: Control | 11/9 | 61.23 (14.24) | NR | 3.98 (2.05) d | No injection or LFrTMS | N/A | N/A | Rehabilitation | MAS  FMA-LE  BI | 4, 8, and 12 weeks after treatment | NR |
|  | G2: BoNTA | 12/8 | 60.89 (15.16) | NR | 4.02 (3.17) d | BoNTA (BOTOX): 5~10 U for each site, 10~15 sites for each muscle, maximum dose 600 U | Lower limb | Electromyographic guidance was used for the injections. | Rehabilitation |  |  |  |
|  | G3: LFrTMS | 10/10 | 62.18 (13.66) | NR | 4.61 (2.50) d | 1 Hz, 3T, 90% RMT, pulses | M1 of unaffected hemisphere | 20 min each time, once daily, 6 days weekly for 4 weeks | Rehabilitation |  |  |  |
| Wang, 2017 | G1: Control | 11/9 | 51.23 (14.24) | NR | 7.98 (3.05) m | No injection | N/A | N/A | Rehabilitation | MAS  FMA-LE  BI | 1, 4, and 16 weeks after treatment | NR |
|  | G2: BoNTA | 12/8 | 50.89 (15.16) | NR | 8.02 (3.17) m | BoNTA (BOTOX): 600 U in total | Lower limb | Landmarks were utilised. | Rehabilitation |  |  |  |
| Wang, 2022 | G1: Control | 20 | 62.18 (1.26) | NR | NR | No injection | N/A | N/A | Rehabilitation | MAS  BI | Post-treatment | NR |
|  | G2: BoNTA | 20 | 61.75 (1.34) | NR | NR | BoNTA (CBTX-A): 5~15 U for each site | Upper limb (Biceps brachii, brachialis, etc.) | Ultrasonography guidance was used for the injections. | Rehabilitation |  |  |  |
| Wang, 2013 | G1: Control | 18/12 | 56.8 (9.3) | 20/10 | NR | No injection | N/A | N/A | Rehabilitation | MAS  SIS | 2, 6, and 12 weeks after injection | NR |
|  | G2: BoNTA | 20/10 | 55.6 (8.7) | 21/9 | NR | BoNTA (CBTX-A): 5~10 U for each site, 10~15 sites for each muscle, maximum dose 600 U | Upper limb (Biceps brachii and forearm) | Landmarks were utilised. | Rehabilitation |  |  |  |
| Wu, 2017 | G1: Control | 19/15 | 65.8 (2.1) | NR | 3.7 (0.5) m | No injection | N/A | N/A | Rehabilitation | MAS  BI | Post-treatment | NR |
|  | G2: BoNTA | 18/16 | 65.2 (2.0) | NR | 3.8 (0.6) m | BoNTA (CBTX-A): maximum dose 600 U | Upper limb (Biceps brachii and forearm) | Ultrasonography guidance was used for the injections. | Rehabilitation |  |  |  |
| Wu, 2019 | G1: Control | 26/24 | 60.57 (6.04) | All ischemic strokes | 20.32 (2.89) d | No injection | N/A | N/A | Rehabilitation | MAS*  GMFM  Gait analysis  FMA-LE | Post-treatment | NR |
|  | G2: BoNTA | 29/21 | 58.30 (5.12) | All ischemic strokes | 21.78 (3.16) d | BoNTA (CBTX-A): 50 U for each site, 2~4 sites for each muscle | Lower limb | Ultrasonography guidance was used for the injections.  Re-injection was administrated 3 months later. | Rehabilitation |  |  |  |
| Wu, 2014 | G1: Control | 8/4 | 58 (14) | 7/5 | 23.2 (17.2) d | Identical volume of saline | Lower limb | Electromyographic and electrostimulation guidance was used for the injections. | Rehabilitation | FMA-LE  MAS  BI  Gait analysis  6MWT | Post-treatment | NR |
|  | G2: BoNTA | 7/4 | 55 (12) | 6/5 | 24.2 (12.2) d | BoNTA (BOTOX): 50 U*2 for medial and lateral heads of gastrocnemius, 25 U*2 for soleus, and 50 U for tibial posterior muscle |  |  | Rehabilitation |  |  |  |
| Wu, 2015 | G1: Control | 20/15 | 59.5 (4.9) | NR | NR | No injection | N/A | N/A | Rehabilitation | UEFT  MAS | Post-treatment | NR |
|  | G2: BoNTA | 21/14 | 59.8 (5.2) | NR | NR | BoNTA (CBTX-A): 12~16 U for each site, 6~8 sites om total | Upper limb (Biceps brachii) | Landmarks were utilised.  Once a week. | None |  |  |  |
| Xia, 2016 | G1: Control | 17 | 57.3 (4.8) | NR | NR | No injection | N/A | N/A | Primus RS | MAS  BBS  ROM | Post-treatment | NR |
|  | G2: BoNTA | 17 |  | NR | NR | BoNTA (BOTOX): 2~3 sites for each muscle, maximum dose for each site 50 U | Lower limb | Electrostimulation guidance was used for the injections. | Primus RS |  |  |  |
| Xia, 2014 | G1: Control | 14/18 | 55.0 (4.2) | 14/18 | 1~5 m | No injection | N/A | N/A | Rehabilitation | MAS  FMA-LE  BI | Post-treatment | NR |
|  | G2: BoNTA | 17/15 | 57.0 (3.6) | 16/16 | 1~5 m | BoNTA (CBTX-A): 10~20 for each site, 2~4 sites for each muscle group, maximum dose 400 U | Upper limb and lower limb | Electromyographic and electrostimulation guidance was used for the injections. | Rehabilitation |  |  |  |
| Xia, 2021 | G1: Control | 18/12 | 66.47 (6.39) | 17/13 | NR | No injection | N/A | N/A | Rehabilitation | MAS, CSI, Fiber length, Muscular thickness, Modulus of rigidity, ROM, and FMA-UE | Post-treatment | NR |
|  | G2: BoNTA | 17/13 | 65.22 (6.41) | 16/14 | NR | BoNTA (BOTOX): maximum dose for each site 30 U, 600 U in total | Upper limb (Biceps brachii and forearm) | Ultrasonography guidance was used for the injections. | Rehabilitation |  |  |  |
| Xia, 2022 (HBMJ) | G1: Control | 25/21 | 65.87 (5.09) | 29/17 | NR | No injection | N/A | N/A | Rehabilitation | Fascicle length, muscular thickness, modulus of rigidity, cross-sectional area, MAS, and CSI | 2 and 4 weeks after treatment | NR |
|  | G2: BoNTA | 26/20 | 65.92 (5.27) | 31/15 | NR | BoNTA (BOTOX):  100U: Biceps brachii  80 U: FCU  70 U: FCR, FDP, FDS  60 U: FHL and brachialis  50 U: brachioradialis  40 U: palmaris longus  Each site less than 30 U, maximum dose 600 U in total | Upper limb | Ultrasonography guidance was used for the injections. | Rehabilitation |  |  |  |
| Xiong, 2020 | G1: Control | 13/7 | 56.7 (4.5) | 9/11 | NR | No injection | N/A | N/A | Rehabilitation | MAS  Brunnstrom stage | 2 and 4 weeks after treatment | No adverse events were observed. |
|  | G2: BoNTA | 12/8 | 58.3 (3.6) | 7/13 | NR | BoNTA (CBTX-A): 5~10 for each site, 20~30 U and 3 sites for each muscle, maximum dose 500 U | Upper limb and lower limb | Electromyographic and electrostimulation guidance was used for the injections. | Rehabilitation |  |  |  |
| Xu, 2004 | G1: Control | 12/3 | 59.07 (9.37) | 6/9 | NR | No injection | N/A | N/A | Rehabilitation | MAS  Brunnstrom stage  FMA-UE  FIM | 3 days, 1, 2, 3 months after injection | No adverse events were observed. |
|  | G2: BoNTA | 13/2 | 57.47 (7.83) | 4/11 | NR | BoNTA (CBTX-A): 25~75 for each muscle, 1~5 muscles in total |  | Landmarks were utilised.  Re-injection was administrated within 1 week if necessary, but the maximum dose should not exceed 400 U. | Rehabilitation |  |  |  |
| Yang, 2021 (CJCM) | G1: Control | 14/10 | 45.83 (6.56) | All ischemic strokes | 4.4 (1.53) m | No injection | N/A | N/A | Acupuncture + rehabilitation | FMA-UE  MAS  BI  Root mean square | 2 and 4 weeks after treatment | NR |
|  | G2: BoNTA | 12/12 | 45.41 (5.76) | All ischemic strokes | 4.4 (1.65) m | BoNTA (CBTX-A): 2~4 for each site, no more than 50 U for each muscle, maximum dose 400 U in total | Upper limb | Ultrasonography guidance was used for the injections. | Acupuncture + rehabilitation |  |  |  |
| Yang, 2018 (CJN) | G1: Control | 46/14 | 51.17 (13.53) | NR | More than 3 months | Identical volume of placebo | Upper limb | Electromyographic guidance was used for the injections. | None | MAS*, area under the curve, and global assessment scale | 1, 4, 6, 8, 12, 16, and 18 weeks after injection | 10 and 3 adverse events were reported in BoNTA and Control groups, respectively. |
|  | G2: BoNTA | 89/29 | 53.01 (13.38) | NR |  | BoNTA (CBTX-A): 200 U in total, 240 U for subjects with concomitant thumb muscle tone disorders |  |  | None |  |  |  |
| You, 2020 | G1: Control | 22/14 | 45.18 (3.48) | NR | NR | No injection | N/A | N/A | Medication | Effective rate  F/M wave  MAS  FMA | 1 and 4 weeks after treatment | NR |
|  | G2: BoNTA | 20/16 | 45.96 (3.17) | NR | NR | BoNTA (BOTOX): upper limb 100 U, lower limb 100U | Upper limb and lower limb | A single injection was given under electromyographic guidance | Medication |  |  |  |
| Zhang, 2010 | G1: Control | 19/11 | 65.1 | 20/10 | NR | No injection | N/A | N/A | Acupuncture | MAS  FMA-UE | Post-treatment | NR |
|  | G2: BoNTA | 17/13 | 62.4 | 22/8 | NR | BoNTA (CBTX-A): 5~10 for each site, 4 sites for each muscle, 2~3 muscles each time, maximum dose 100 U | Upper limb | Landmarks were utilised. | Acupuncture |  |  |  |
| Zhang, 2010 (NMFC) | G1: Control | 13/4 | 44.52 (11.9) | 8/9 | 7.1 (1.54) m | No injection | N/A | N/A | Rehabilitation | MAS  FMA-LE  10MWT | 2 and 4 weeks after treatment | No systemic side effects were observed, only 5 cases experienced mild soreness. |
|  | G2: BoNTA | 15/3 | 41.39 (14.3) | 6/12 | 7.5 (2.04) m | BoNTA (CBTX-A): 2~4 sites for each muscle, no more than 100 U for each muscle, maximum dose 400 U | Lower limb | Eectrostimulation guidance was used for the injections.  Re-injection was given within 1 weeks if necessary. | Rehabilitation |  |  |  |
| Zhang, 2010 (CJPND) | G1: Control | 34 | 34 (6.2) | NR | NR | No injection | N/A | N/A | Rehabilitation | MAS | 7 days, 2 weeks, 1, 3, and 6 months after injection | NR |
|  | G2: BoNTA | 36 |  | NR | NR | BoNTA (CBTX-A): maximum dose 300 U | NR | Electromyographic guidance was used. | Rehabilitation |  |  |  |
| Zhang, 2014 | G1: Control | 21/16 | 53.21 (7.83) | 22/15 | 126.7 (23.1) d | No injection | N/A | N/A | Rehabilitation | MAS | 1 week, 1, 2, and 3 months after treatment | NR |
|  | G2: BoNTA | 20/15 | 52.69 (8.01) | 22/13 | 127.5 (22.4) d | BoNTA (CBTX-A): 5~20 U for each site, 3~5 sites for each muscle, maximum dose 400 U | Upper limb (Biceps brachii and forearm) | Electromyographic guidance was used. | Rehabilitation |  |  |  |
| Aşkın, 2017 | G1: Control | 15/5 | 58.80 (12.02) | All ischemic strokes | 24.35 (15.39) m | No LFrTMS | N/A | N/A | Physiotherapy | Brunnstrom stage, FMA-UE, BBT, MAS, FIM, MMSE, and FAC | Post-treatment | No adverse events were reported. |
|  | G2: LFrTMS | 14/6 | 56.75 (11.46) | All ischemic strokes | 28.35 (15.34) m | 1 Hz, 90% RMT, 1200 pulses | M1 of the unaffected hemisphere | 20 min, 10 sessions in 2 weeks (5 days/week) | Physiotherapy |  |  |  |
| Barros Galvão, 2014 | G1: Control | 7/3 | 64.6 (6.8) | 8/2 | 58.9 (27.2) m | Sham stimulation | M1 of the unaffected hemisphere | 10 sessions, 3days/week | Physiotherapy | MAS*, FMA-UE, ROM, FIM, and SSQoL | Post-treatment and 1 month follow-up | No adverse events were reported. |
|  | G2: LFrTMS | 6/4 | 57.4 (12.0) | 9/1 | 47.8 (43.2) m | 1 Hz, 90% RMT, 1500 pulses | M1 of the unaffected hemisphere | 10 sessions, 3days/week | Physiotherapy |  |  |  |
| Bian, 2024 | G1: Control | 16/5 | 59.95 (9.18) | 16/5 | 29 (23.5) d | Sham stimulation | M1 of the affected hemisphere | One session per day, 5 days per week, for 3 consecutive weeks, 15 sessions in total | Rehabilitation | FMA-UE*, NIHSS, Hong Kong version of the functional test for the hemiplegic upper extremity, upper extremity  strength index, BI, and MAS | Post-treatment | No significant adverse effects were  reported in either group. |
|  | G2: iTBS | 16/4 | 65.20 (7.09) | 17/3 | 26 (31.5) d | 80% AMT, 3 pulses at 50 Hz repeated at 5Hz; a 2 seconds train of TBS was repeated every 10 seconds for 192 seconds | M1 of the affected hemisphere | One session per day, 5 days per week, for 3 consecutive weeks, 15 sessions in total | Rehabilitation |  |  |  |
| Chen, 2021 | G1: Control | 12/4 | 51.44 (9.19) | 8/8 | 101.50 (54.15) d | Sham stimulation | Ipsilesional lateral cerebellum (1 cm inferior and 3 cm lateral to the inion) | One session per day, 5 days per week, for 2 consecutive weeks, 10 sessions in total | Physiotherapy | MAS*, MTS*, shear wave velocity*, *H*_max_/*M*_max_, neurophysiological parameters, and BI | Post-treatment | The whole procedure was well-tolerated, and no adverse events were reported in either group. |
|  | G2: iTBS | 13/3 | 57.38 (8.04) | 10/6 | 80.13 (35.19) d | a total of 600 pulses over 200 seconds delivered at 80% AMT |  |  | Physiotherapy |  |  |  |
| Chen, 2021 (JNER) | G1: Control | 10/1 | 48.95 (9.63) | 2/9 | 7.99 (5.41) m | Sham stimulation | Hand motor area of the affected hemisphere | 15 consecutive work days. | Virtual reality‑based cycling training | MAS*, FMA-UE, ARAT, BBT, NHPT, MAL, and SIS | Within 3 days after completing  the therapy | All patients could tolerate the intervention without significant iTBS-related adverse effects throughout the study. |
|  | G2: iTBS | 8/4 | 54.36 (10.56) | 6/6 | 5.01 (4.39) m | Two sessions with a 10-min break, a session comprised 2-s train of bursts, containing 3 pulses at 50 Hz, repeated at intervals of 200 ms, every 10 s for 20 times (a total of 600 pulses), delivered at 80% AMT |  |  | Virtual reality‑based cycling training |  |  |  |
| Chen, 2019 | G1: Control | 7/4 | 52.6 (8.3) | 8/3 | ≥ 6 months | Sham stimulation | Hand motor area of the affected hemisphere | 5 times/week for 2 consecutive weeks | Rehabilitation | MAS  FMA-UE  ARAT  BBT  MAL | Within 3 days after completing | No adverse events throughout the study course. |
|  | G2: iTBS | 7/4 | 52.9 (11.1) | 9/2 |  | A 2-s train of bursts, which contained three 50-Hz pulses repeated every 200 ms (i.e., 5 Hz) at an intensity of 80% AMT, every 10 s for 20 times (600 pulses in total) |  |  | Rehabilitation |  |  |  |
| Chervyakov, 2018 | G1: Control | 5/5 | 61.4 (11.4) | NR | 7.9 (8.4) m | Sham stimulation | M1 of affected hemisphere | 10 minutes per session, five times a week for two consecutive weeks | Rehabilitation | FMA-UE  MAS  BI | Post-treatment | 1 case presented with seizures during single-pulse stimulation for diagnostic mapping.  1 case developed seizures in the HFrTMS group.  29 patients, including 73% in HFrTMS group, showed increased paroxysmal or newly emerged epileptiform EEG activity after 10 sessions.  One patient experienced an aggravated somatic status involving lower extremity deep vein thrombosis and thrombus flotation. |
|  | G2: HFrTMS | 10/3 | 58.6 (10.4) | NR | 5.8 (4.6) m | 200 stimuli of 10 Hz rTMS at 80% of RMT to the affected hemisphere for 10 minutes | M1 of affected hemisphere | 10 minutes per session, five times a week for two consecutive weeks | Rehabilitation |  |  |  |
|  | G3: LFrTMS | 5/6 | 54.2 (11.1) | NR | 5.1 (4.8) m | A single train of 1200 stimuli of 1 Hz rTMS at 100% RMT | M1 of unaffected hemisphere | 20 minutes per session, five times a week for two consecutive weeks | Rehabilitation |  |  |  |
| Chieffo, 2021 | G1: Control | 2/4 | 61.17 (8.70) | 3/3 | 41.00 (23.74) m | Sham stimulation | Leg motor cortex of the two sides, up to 3 cm within the brain | 15 min per session, 3-week cycles of 11 rTMS sessions (5 in the first week and 3 in the second and third weeks) | Cycling | NIHSS  FMA-LE  MAS  10MWT  6MWT | Post-treatment | No serious adverse events were reported. 3 subjects reported transitory dizziness, and one showed muscle twitches on shoulders during real rTMS. |
|  | G2: HFrTMS | 4/2 | 58.67 (10.33) | 4/2 | 41.5 (26.77) m | 40 2s-trains at 20 Hz, 20 sec inter-train interval, total number of pulses 1600 at 90% RMT |  |  | Cycling |  |  |  |
| Dos Santos, 2019 | G1: Control | 7/3 | 64.6 (6.8) | 8/2 | 50.1 (27.2) m | Sham stimulation | A coil disconnected was held over the scalp, while a second coil connected to the stimulator was positioned behind the patient’s head without touching the scalp | three times per week, 10 sessions in total | Physiotherapy | MAS and cortical excitability | Post-treatment | No adverse events were reported by any of the participants. |
|  | G2: LFrTMS | 6/4 | 52.4 (12) | 9/1 | 47.8 (43.2) m | 1-Hz, 1500 pulses with an intensity  of 90% RMT | M1 of the unaffected hemisphere |  | Physiotherapy |  |  |  |
| Gottlieb, 2021 | G1: Control | 3/11 | 62.43 (11.46) | 12/2 | 44.21 (35.17) d | Sham stimulation | The sham coil elicited  the pulses in the opposite direction | Ten sessions were employed  over a period of 12 days (5 sessions per week). | Physiotherapy | MAS*  FAM-UE  Resting-state fMRI data | Post-treatment | The frequency of reported adverse events was equal in both groups. |
|  | G2: LFrTMS | 9/5 | 63.93 (10.91) | 13/1 | 41.86 (30.85) d | A single train with a rate of 1 Hz, 1200 pulses with an intensity of 100% RMT | Hand area of M1 of the unaffected hemisphere |  | Physiotherapy |  |  |  |
| Kuzu, 2021 | G1: Control | 2/4 | 65.0 (4.6) | All ischemic strokes | 14.5 (2) m | Sham cTBS | M1 of the unaffected hemisphere | 10 sessions | Physiotherapy | MAS*  FAM-UE*  FIM  MAL  Brunnstrom stage | Post-treatment and 4 weeks after treatment | No side effects were observed in any of the  patients during and after TMS applications. |
|  | G2: LFrTMS | 4/3 | 56.3 (11.5) | All ischemic strokes | 16.4 (2.5) m | 1 Hz, 1200 pulses, 90% RMT |  | 20 min per session, 10 sessions in total | Physiotherapy |  |  |  |
|  | G3: cTBS | 6/1 | 61.3 (9.8) | All ischemic strokes | 14.5 (1.6) m | 3 burst stimulations of 50 Hz repeated every 200 ms for 40 s at 80% RMT |  | 10 sessions | Physiotherapy |  |  |  |
| Özkeskin, 2017 | G1: Control | 6/5 | 64.54 (9.38) | All ischemic strokes | 24.50 (23.88) m | Sham stimulation | M1 of unaffected hemisphere | 25 min each session, 5 consecutive days with two days intervals, followed by another five consecutive days | Brunnstrom movement therapy + Do-it-yourself exercises | MMSE  Brunnstrom stage  Proprioceptive sense  Figer touch localisation  MAS | 10 days, 1, and 3 months post-treatment | NR |
|  | G2: LFrTMS | 7/3 | 55.70 (14.92) | All ischemic strokes | 10.45 (21.80) m | 1 Hz rTMS at 90% of RMT with the TMS device (1500 pulses). |  |  | Brunnstrom movement therapy + Do-it-yourself exercises |  |  |  |
| Qin, 2023 | G1: Control | 11/3 | 59.43 (9.12) | All ischemic strokes | 2.85 (1.74) m | No stimulation | N/A | N/A | Rehabilitation | MAS  FAM-UE  BI  fMRI data | Post-treatment | NR |
|  | G2: LFrTMS | 9/6 | 55.87 (10.50) | All ischemic strokes | 3.20 (1.93) m | 1 Hz at 90% RMT, 10 pulses were delivered in each sequence with an interval of 2 s, 1,200 pulses in total | M1 of the unaffected hemisphere | Once a day, 5 days a week,  for 8 weeks | Rehabilitation |  |  |  |
| Rastgoo, 2016 | G1: Control | 16/4 | 53.15 (11.63) | 15/5 | 28.8 (18.76) m | Sham stimulation | Lower limb motor cortex of the unaffected hemisphere | 20 min, 5 consecutive daily sessions | None | MAS, H-reflex, FMA-LE, and TUG | Post-treatment and 1 week after treatment | No adverse events were reported by any of the patients throughout the study. |
|  | G2: LFrTMS |  |  |  |  | a train of 1000 pulses of 1-Hz rTMS with an intensity of 90% of the tibialis anterior RMT |  |  | None |  |  |  |
| Watanabe, 2018 | G1: Control | 3/3 | 75.2 (5.5) | All ischemic strokes | Within 7 days | Sham stimulation (600 pulses and 80% RMT with a plastic board) | Hand area of M1 of affected hemisphere | Once a day for 10 days | Rehabilitation + medication | FMA-UE  SIS  MAS  Grip strength  MEP | 12 weeks after stroke onset. | No complication occurred, and no patients had symptoms suggestive of seizure. |
|  | G2: LFrTMS | 6/1 | 67.6 (6.4) | All ischemic strokes |  | 1 Hz, 1200 pulses, 110% RMT | Hand area of M1 of unaffected hemisphere |  | Rehabilitation + medication |  |  |  |
|  | G3: iTBS | 5/3 | 72.5 (6.5) | All ischemic strokes |  | 80% RMT, 600 pulses | Hand area of M1 of affected hemisphere |  | Rehabilitation + medication |  |  |  |
| Chen, 2021 (CJRM) | G1: Control | 20/10 | 61.37 (11.90) | 23/7 | 2.17 (11.1) m | Sham stimulation | Hand area of M1 of unaffected hemisphere | 20 min each session, once a day, 5 days a week, 4 weeks in total | Rehabilitation + medication | MAS, F-wave, *H*_max_/*M*_max_, FMA-UE, and BI | Post-treatment | No adverse events were reported. |
|  | G2: LFrTMS | 20/10 | 64.13 (13.20) | 26/4 | 2.00 (1.34) m | 1 Hz, 90% RMT, 1200 pulses |  |  | Rehabilitation + medication |  |  |  |
| Chen, 2018 | G1: Control | 56/14 | 51.3 (12.1) | 48/22 | 27.6 (19.3) m | Sham stimulation | Lower limb motor cortex of the unaffected hemisphere | 20 min each session, once a day for 5 days | Rehabilitation | MAS, *H*_max_/*M*_max_, TUG, and FMA-LE | Post-treatment and 1 week follow-up | No adverse events were reported. |
|  | G2: LFrTMS | 49/21 | 55.2 (11.5) | 51/19 | 31.6 (17.9) m | 1 Hz, 90% RMT of tibial anterior |  |  | Rehabilitation |  |  |  |
| Cheng, 2022 | G1: Control | 83/37 | 61.75 (3.97) | All ischemic strokes | More than 3 months | No stimulation | N/A | N/A | Rehabilitation | MEP, MAS, FMA-UE, BI, and fMRI | Post-treatment | NR |
|  | G2: LFrTMS | 81/39 | 61.58 (4.06) | All ischemic strokes |  | 1 Hz, 90% RMT, 1200 pulses | M1 of unaffected hemisphere | Once a day, 5 days/week, for 8 weeks | Rehabilitation |  |  |  |
| Deng, 2019 | G1: Control | 19/19 | 55.85 (7.01) | 21/17 | 62 (29.56) d | No stimulation | N/A | N/A | Medication | FMA-UE  MAS | Post-treatment | NR |
|  | G2: HFrTMS | 21/17 | 56.01 (6.97) | 19/19 | 61 (29.98) d | 5 Hz, 90% RMT, 900 pulses | M1 of affected hemisphere | 30 min each session, once a day, 5 days/week, for 6 weeks | Medication |  |  |  |
| Huang, 2023 | G1: Control | 22/18 | 60.23 (5.43) | 30/10 | 46.74 (8.32) d | No stimulation | N/A | N/A | Usual care + acupuncture | iEMG  Strength  MAS  Gait analysis  FMA-LE  BI | Post-treatment | No adverse events were reported. |
|  | G2: LFrTMS | 20/20 | 61.47 (6.74) | 28/12 | 45.39 (7.96) d | 1 Hz, 110% RMT, 1200 pulses | M1 of unaffected hemisphere | Once a day, 5 days/week with an interval of 2 days, for 4 consecutive weeks | Usual care + acupuncture |  |  |  |
| Jiang, 2023 | G1: Control | 17/8 | 54.56 (12.68) | NR | 2.66 (1.12) m | No stimulation | N/A | N/A | Medication + rehabilitation + acupuncture | MAS  FMA-UE  BI  RMS | Post-treatment | No adverse events were observed, but 1 case presented with mild pain on the rTMS application site. |
|  | G2: LFrTMS | 13/12 | 56.72 (10.50) | NR | 2.62 (1.18) m | 1 Hz, 90% RMT, 1200 pulses | M1 of unaffected hemisphere | 20 min per sessions, once a day, 5 days/week, for 4 weeks |  |  |  |  |
| Li, 2018 | G1: Control | 5/5 | 64.9 (8.0) | 4/6 | 46.7 (21.7) d | No stimulation | N/A | N/A | Medication + rehabilitation | FMA-UE  ARAT  MAS | Post-treatment | No adverse events were observed. |
|  | G2: LFrTMS | 4/6 | 63.2 (8.9) | 5/5 | 48.6 (17.0) d | 1 Hz, 90% RMT, 224 pulses | Upper limb motor cortex of the unaffected hemisphere | 20 min each session, once a day, 5 days/week, for 4 weeks | Medication + rehabilitation |  |  |  |
| Liang, 2024 | G1: Control | 21/19 | 55.21 (2.06) | All ischemic strokes | 3.25 (1.03) y | No stimulation | N/A | N/A | Medication + rehabilitation | MAS, ROM, VAS, FMA, BBS, BI, complication | Post-treatment | No significant difference was found between groups. |
|  | G2: LFrTMS | 22/18 | 52.32 (3.08) | All ischemic strokes | 3.46 (1.02) y | 1 Hz, 90% RMT, 1250 pulses | Hand area of M1 of unaffected hemisphere | 25 min per session, once a day, 5 days/week with an interval of 2 days, for 1 month | Medication + rehabilitation |  |  |  |
| Liang, 2023 | G1: Control | 39/25 | 57.8 (12.6) | 35/29 | 4.8 (3.0) w | Sham stimulation | M1 of unaffected hemisphere | Once a day, 6 days/week, for 4 weeks | Medication + rehabilitation + NGF | MAS  FMA  FIM | Post-treatment | No adverse events were observed. |
|  | G2: LFrTMS | 37/27 | 55.9 (11.8) | 31/33 | 4.6 (2.9) w | 1 Hz, 90% RMT, 600 pulses |  |  |  |  |  |  |
| Liu, 2023 | G1: Control | 30/20 | 72.37 (5.63) | NR | 20.21 (5.44) d | No stimulation | N/A | N/A | Acupuncture | BDNF, NGF, Blood flow velocity, MAS, FMA-UE, and NIHSS | Post-treatment | No adverse events were observed. |
|  | G2: LFrTMS | 28/22 | 73.05 (6.31) | NR | 21.41 (5.61) d | 1 Hz, 1200 pulses | M1 of unaffected hemisphere | 20 min per session, once a day, 5 days/week, for 4 weeks | Acupuncture |  |  |  |
| Liu, 2019 | G1: Control | 11/9 | 55.00 (11.86) | NR | 3.11 (1.37) m | No stimulation | N/A | N/A | Usual care | MAS, FMA-UE, BI, RMS, MEP | Post-treatment | NR |
|  | G2: LFrTMS | 7/13 | 61.35 (9.43) | NR | 2.81 (1.27) m | 1 Hz, 120% AMT, 1200 pulses | M1 of unaffected hemisphere | 20 min per session, once a day, 6 days/week, for 4 weeks | Usual care |  |  |  |
| Liu, 2018 (CJR) | G1: Control | 9/6 | 56.13 (8.97) | All ischemic strokes | 2.87 (1.51) m | No stimulation | N/A | N/A | Medication + physiotherapy | MAS  RMS  FMA-UE  BI | Post-treatment | No adverse events were observed. |
|  | G2: LFrTMS | 8/7 | 55.67 (11.59) | All ischemic strokes | 2.67 (1.59) m | 1 Hz, 80% RMT, 1200 pulses | M1 of unaffected hemisphere | 20 min per session, once a day, 6 days/week, for 4 weeks | Medication + physiotherapy |  |  |  |
| Liu, 2018 | G1: Control | 9/4 | 55.38 (8.40） | All ischemic strokes | 4.85 (2.08) m | No stimulation | N/A | N/A | Rehabilitation | MAS  FMA-UE  BI  fMRI | Post-treatment | NR |
|  | G2: LFrTMS | 5/5 | 56.90 (9.02) | All ischemic strokes | 4.50 (1.90) m | 1 Hz, 90% RMT, 1200 pulses | M1 of unaffected hemisphere | 24 min per session, once a day, 5 days/week, for 8 weeks | Rehabilitation |  |  |  |
| Liu, 2019 (CFMR) | G1: Control | 11/9 | 58.05 (8.48) | All ischemic strokes | 2.78 (1.70) m | Sham stimulation | M1 of unaffected hemisphere | 20 min each session, once a day, 5 days/week, for 8 weeks | Rehabilitation | MAS  FMA-UE  BI | Post-treatment | NR |
|  | G2: HFrTMS | 12/9 | 55.43 (6.72) | All ischemic strokes | 1.51 (0.51) m | 10 Hz, 80% RMT, 1500 pulses | M1 of unaffected hemisphere |  | Rehabilitation |  |  |  |
| Sun, 2017 | G1: Control | 15/5 | 53.5 (7.9) | 10/10 | 1.8 (1.1) m | Sham stimulation | Upper limb of M1 of the unaffected hemisphere | Once a day, 6 days/week, for 4 weeks | Medication + rehabilitation | MAS, FMA-UE, BI, MEP, and F-wave | Post-treatment and 2 weeks follow-up | No adverse events were observed, but 1 case in rTMS group experienced a headache lasting for 5 min. |
|  | G2: LFrTMS | 17/3 | 55.1 (8.5) | 12/8 | 2.0 (1.5) m | 1 Hz, 80% RMT, 1200 pulses |  |  | Medication + rehabilitation |  |  |  |
| Tang, 2021 | G1: Control | 11/9 | 64.25 (15.91) | 8/12 | 39.81 (10.36) d | No stimulation | N/A | N/A | Medication + rehabilitation | FMA-UE  BI  MAS | Post-treatment | No adverse events were observed. |
|  | G2: HFrTMS | 12/8 | 60.91 (16.82) | 7/13 | 36.65 (11.52) d | 10 Hz, 90% RMT, 1200 pulses | M1 of affected hemisphere | 20 min each session, once a day, 5 days/week, for 4 weeks | Medication + rehabilitation |  |  |  |
| Tao, 2023 | G1: Control | 30/28 | 67.04 (4.46) | All ischemic strokes | NR | No stimulation | N/A | N/A | Rehabilitation | MAS  BI  BBS  FMA-UE  FMA-LE | Post-treatment | NR |
|  | G2: HFrTMS | 31/27 | 66.45 (422) | All ischemic strokes | NR | 10 Hz, 80% RMT, 1410 pulses | M1 of affected or unaffected hemisphere | 15 min each session, once a day, 5 days/week, for 21 days | Rehabilitation |  |  |  |
| Wang, 2023 | G1: Control | 12/6 | 53.44 (16.60) | 10/8 | 7.17 (2.26) w | Sham stimulation | Leg motor cortex of the two sides | Once a day, 6 days/week, for 3 weeks | Rehabilitation | Strength  FMA-LE  BBS  MAS  MEP  CMCT | Post-treatment | NR |
|  | G2: HFrTMS | 12/6 | 56.72 (14.20) | 12/6 | 6.83 (2.15) w | 10 Hz, 80% RMT, 1200 pulses |  | 12 min each session, once a day, 6 days/week, for 3 weeks | Rehabilitation |  |  |  |
|  | G3: iTBS | 13/5 | 54.83 (12.57) | 12/6 | 6.61 (1.69) w | 40 trains of 10 bursts at a frequency of 5 Hz, 3 pulses at 35 Hz in each burst, 70% RMT, 1200 pulses |  | 400 s each session, once a day, 6 days/week, for 3 weeks | Rehabilitation |  |  |  |
| Wang, 2021 | G1: HFrTMS | 33/29 | 63.32 (3.84) | 55/7 | 2.62 (0.25) m | 10 Hz, 90% RMT, 1.5 s of stimulation with an interval of 10 s | M1 of affected hemisphere | 20 min each session, once a day, 5 days/week with an interval of 2 days, for 2 weeks | Medication + rehabilitation | FMA, WMFT, MAS  MEP, and CMCT | Post-treatment | NR |
|  | G2: LFrTMS | 32/30 | 63.19 (3.78) | 54/8 | 2.56 (0.21) m | 1 Hz, 90% RMT, 1000 pulses | M1 of unaffected hemisphere |  | Medication + rehabilitation |  |  |  |
| Xia, 2022 | G1: Control | 10/8 | 69.6 (1.7) | NR | 74.1 (33.9) d | No stimulation | N/A | N/A | Medication + rehabilitation | MAS  CSI  FNA-UE  BI  MEP  CMCT | Post-treatment | 2 cases and 1 case experienced temporary headaches in HFrTMS group and LFrTMS group, respectively. |
|  | G2: HFrTMS | 10/8 | 69.1 (1.9) | NR | 76.5 (35.7) d | 10 Hz, 90% RMT, 1200 pulses | M1 of affected hemisphere | Once a day, 5 days/week, for 4 weeks | Medication + rehabilitation |  |  |  |
|  | G3: LFrTMS | 11/7 | 70.4 (2.1) | NR | 78.2 (37.5) d | 1 Hz, 90% RMT, 1200 pulses | M1 of unaffected hemisphere |  | Medication + rehabilitation |  |  |  |
| Xia, 2022 (NIFR) | G1: Control | 22/18 | 69.9 (1.8) | All ischemic strokes | 72.3 (34.8) d | No stimulation | N/A | N/A | Medication + rehabilitation | MAS, CSI, FMA-UE, BI, MEP, and CMCT | Post-treatment | 2 cases experienced temporary headaches in HFrTMS group. |
|  | G2: HFrTMS | 25/15 | 69.3 (1.9) | All ischemic strokes | 76.6 (39.3) d | 20 Hz, 90% RMT | M1 of affected hemisphere | Once a day, 5 days/week, for 4 weeks | Medication + rehabilitation |  |  |  |
| Xiao, 2019 | G1: Control | 12/7 | 57.68 (10.66) | All ischemic strokes | 72.95 (41.38) d | Sham stimulation | M1 of unaffected hemisphere | 9 min each session, once a day, 5 days/week, for 2 weeks | Medication + rehabilitation | RMT  MAS  FMA-UE  BI | 2 days and 6 weeks after treatment | No adverse events were observed. |
|  | G2: HFrTMS | 26/15 | 59.53 (10.78) | All ischemic strokes | 66.61 (30.78) d | 3 Hz/10 Hz, 90% RMT, 900 pulses |  | 30 min/9 min each session, once a day, 5 days/week, for 2 weeks | Medication + rehabilitation |  |  |  |
| Xiao, 2018 | G1: Control | 8/6 | 60.9 (10.2) | All ischemic strokes | 74.7 (40.3) d | Sham stimulation | M1 of affected hemisphere | 30 min each session, once a day, 5 days/week, for 2 weeks | Medication + rehabilitation | FMA-UE  MAS  BI | Post-treatment | No adverse events were observed, but 3 patients in rTMS group experienced temporary headache. |
|  | G2: HFrTMS | 8/5 | 59.1 (9.3) | All ischemic strokes | 71.8 (28.0) d | 3 Hz, 90% RMT, 900 pulses |  |  | Medication + rehabilitation |  |  |  |
| Yang, 2023 | G1: Control | 16/13 | 45.66 (2.17) | 15/14 | 3.33 (0.29) m | No stimulation | N/A | N/A | Rehabilitation | MAS  FMA-UE  BI | Post-treatment | NR |
|  | G2: LFrTMS | 17/12 | 46.02 (2.07) | 13/16 | 3.36 (0.24) m | 1 Hz, 80% RMT | Parietal lobe | 20 min each session, once a day, 5 days/week, for 4 weeks | Rehabilitation |  |  |  |
| Yang, 2021 (WLMI) | G1: Control | 4/7 | 66.09 (7.44) | NR | Within 3 months | Sham stimulation | Upper limb of M1 of the unaffected hemisphere | 24 min each session, once a day, 5 days/week, for 8 weeks | Rehabilitation | FMA-UE  MAS  BI | Post-treatment | No adverse events were observed. |
|  | G2: LFrTMS | 8/6 | 60.86 (12.40) | NR |  | 1 Hz, 90% RMT, 1200 pulses |  |  | Rehabilitation |  |  |  |
| Yang, 2020 | G1: Control | 26/19 | 54.5 (9.8) | 25/20 | 53.0 (31.0) d | No stimulation | N/A | N/A | Medication + rehabilitation | MEP  CMCT  iEMG  FMA-UE  MAS | Post-treatment | NR |
|  | G2: LFrTMS | 22/23 | 53.4 (9.8) | 23/22 | 55.9 (32.3) d | 1 Hz, 80% RMT, 1000 pulses | M1 of the unaffected hemisphere | 20 min each session, once a day, 5 days/week, for 4 weeks | Medication + rehabilitation |  |  |  |
| Yu, 2021 | G1: Control | 12/2 | 55.57 (9.43) | NR | 81.86 (47.99) d | No stimulation | N/A | N/A | Rehabilitation | FMA-UE*, BI*, MAS, MMSE, and MEP | 1 day after treatment | No adverse events were observed. |
|  | G2: iTBS | 12/3 | 51.60 (12.78) | NR | 74.27 (42.50) d | 20 bursts at a frequency of 5 Hz, 3 pulses at 50 Hz in each burst, 70% RMT, 600 pulses | M1 of the affected hemisphere | 200 s each session, once a day, 5 days/week, for 2 weeks | Rehabilitation |  |  |  |
|  | G3: rPMS | 11/2 | 55.85 (6.71) | NR | 85.08 (50.41) d | 10 Hz, 20%~35% maximal stimulation output, 1200 pulses | Erb’s point | 440 s each session, once a day, 5 days/week, for 2 weeks | Rehabilitation |  |  |  |
| Yuan, 2020 | G1: Control | 15 | 30~80 | All ischemic strokes | Within 3 months | Sham stimulation | M1 of the unaffected hemisphere | 24 min each session, once a day, 5 days/week, for 8 weeks | Rehabilitation | MAS  FMA-UE  BI | Post-treatment | NR |
|  | G2: LFrTMS | 15 |  | All ischemic strokes |  | 1 Hz, 90% RMT, 1200 pulses |  |  | Rehabilitation |  |  |  |
| Zhang, 2020 | G1: Control | 29/11 | 52.41 (12.49) | 35/5 | 50.14 (13.47) d | No stimulation | N/A | N/A | Physiotherapy | MAS, maximal stimulation output, *Hmax*/*M*_max_, | Post-treatment | NR |
|  | G2: LFrTMS | 28/12 | 50.14 (11.24) | 33/7 | 48.19 (12.49) d | 1 Hz, 90% RMT, 1500 pulses | NR | Once a day, 3 days/week, 10 sessions in total | Physiotherapy |  |  |  |
| Zhao, 2021 (CR) | G1: Control | 35/15 | 56.29 (7.88) | All ischemic strokes | 2.81 (0.79) m | Sham stimulation | M1 of the unaffected hemisphere | Once a day, 6 days/week, for 4 weeks | Physiotherapy | MAS  FMA-UE  BI | Post-treatment and 2 weeks follow-up | NR |
|  | G2: LFrTMS | 36/14 | 56.32 (7.83) | All ischemic strokes | 2.87 (0.82) m | 1 Hz, 80% RMT, 1200 pulses |  |  | Physiotherapy |  |  |  |
| Zhou, 2020 | G1: Control | 19/9 | 61.75 (11.43) | 16/12 | 10.50 (7.38) m | Sham stimulation | Premotor cortex of the affected hemisphere | Once a day, 5 days/week, for 4 weeks | Medication + rehabilitation | FMA-UE  Brunnstrom stage  MAS  BI  WMFT | Post-treatment | No seizure was observed, but 8 cases felt mild headache and local skin pain and burning sensation |
|  | G2: HFrTMS | 22/8 | 59.73 (10.41) | 20/10 | 10.70 (7.57) m | 5 Hz, 80% RMT, 1200 pulses |  |  | Medication + rehabilitation |  |  |  |
| Zhu, 2023 | G1: Control | 14/6 | 64.7 (4.3) | 18/2 | 3.94 (0.58) m | No stimulation | N/A | N/A | Rehabilitation | MAS, BBS, BI, CMCT, NIHSS, MEP, and FMA-LE | Post-treatment | NR |
|  | G2: LFrTMS | 15/11 | 64.6 (5.4) | 23/3 | 4.12 (1.01) m | 1 Hz, 90% RMT, 1200 pulses | M1 of the unaffected hemisphere | 20 min each session, once a day, 5 days/week, for 3 weeks | Rehabilitation |  |  |  |
| Andrade, 2017 | G1: Control | 12/8 | 54.76 (4.28) | 15/5 | 1.92 (1.36) m | Sham stimulation | M1/premotor cortex of affected hemisphere | 10 min each session, 5 consecutive days for two weeks | Constraint- induced movement therapy | BI*  FMA-UE  MAS  BBT  MRC | Post-treatment | 16 out of 60 patients (13 in tDCS group, and 3 in sham group) had mild skin redness, mild headache and sleepiness after stimulation. |
|  | G2: atDCS | 22/18 | 54.08 (3.85) | 25/15 | 1.82 (1.62) m | 0.7 mA, electrode size: 16 cm^2^ |  |  |  |  |  |  |
| Del Felice, 2016 | G1: ctDCS | 7/3 | 62 (40, 80) | 8/2 | 2.3 years (range 9 months to 4 years) | 1 mA, electrode size: 25 cm^2^ | M1 of the unaffected hemisphere | 20 for 5 consecutive days | Medication | MRC, MAS, finger flexion scale, postural assessment scale for stroke patient, ARAT, European stroke scale, HRSD, BI, ROM, and Neurophysiology examination | Post-treatment | NR |
|  | G2: dtDCS |  |  |  |  |  | M1 of the unaffected (cathodal) and affected (anodal) hemisphere |  | Medication |  |  |  |
| Hesse, 2011 | G1: Control | 21/11 | 65.6 (10.3) | All ischemic strokes | 3.8 (1.5) w | Sham stimulation, 0 mA, | Hand area of M1 of affected/unaffected hemisphere | 20 min each session, once a day, 5 days/week, for 6 weeks, 30 sessions in total | Arm robot training + comprehensive rehabilitation | FMA-UE*  MRC  MAS  BBT | Post-treatment and 3 months after treatment | Relevant side effects did not occur. |
|  | G2: atDCS | 20/12 | 63.9 (10.5) | All ischemic strokes | 3.4 (1.8) w | 2 mA, electrode size: 35 cm^2^ | Hand area of M1 of affected hemisphere |  |  |  |  |  |
|  | G3: ctDCS | 18/14 | 65.4 (8.6) | All ischemic strokes | 3.8 (1.4) w |  | Hand area of M1 of unaffected hemisphere |  |  |  |  |  |
| Hua, 2020 | G1: Control | 22/18 | 54.60 (8.61) | All ischemic strokes | 41.40 (7.07) d | Sham stimulation | M1 of the unaffected (cathodal) and affected (anodal) hemisphere | 20 min each session, once a day, 5 days/week, for 4 weeks, 20 sessions in total | Medication + rehabilitation | MAS, FMA-UE, ARAT, and BT | Post-treatment | NR |
|  | G2: dtDCS | 26/14 | 55.08 (7.14) | All ischemic strokes | 43.95 (5.76) d | 1.2~1.4 mA, 0.05 mA/cm^2^ |  |  | Medication + rehabilitation |  |  |  |
| Lee, 2014 | G1: Control | 9/11 | 60.6 (14.1) | 14/6 | 16.9 (5.5) d | No stimulation | N/A | N/A | Virtual reality + rehabilitation | MAS, strength, manual functional test, FMA-UE, BBT, and BI | Post-treatment | No major adverse effects were reported by any of the patients  who completed all 15 consecutive sessions. |
|  | G2: ctDCS | 12/8 | 63.1 (10.3) | 12/8 | 17.8 (7.3) d | 2 mA, electrode size: 25 cm^2^ | Hand area of M1 of unaffected cortex | 15 sessions (20 min each session, 30 min/day, and 5 times/week for 3 weeks) | Virtual reality + rehabilitation |  |  |  |
| Mazzoleni, 2019 | G1: Control | 7/12 | 68.74 (15.83) | 16/3 | 25 (7) d | Sham stimulation | M1 of affected cortex | 20 min each session, once a day for five days/week over six weeks. | Wrist robot training | FMA-UE, MAS, Motricity index, BBT, and kinematic parameters. | Post-treatment | No adverse events were reported. |
|  | G2: atDCS | 8/12 | 67.50 (16.30) | 13/7 |  | 2 mA, electrode size: 35 cm^2^ |  |  | Wrist robot training |  |  |  |
| Ochi, 2013 | G1: atDCS | 14/4 | 61.1 (10.0) | 7/11 | 4.4 (3.4) y | 1 mA, electrode size: 25 cm^2^ | M1 of affected cortex | 10 min each session, once a day for five days. | Arm training | FMA-UE, MAS, and MAL | Post-treatment | No adverse events were reported. |
|  | G2: ctDCS |  |  |  |  |  | M1 of unaffected cortex |  | Arm training |  |  |  |
| Qu, 2009 | G1: Control | 22/3 | 45 (14) | 15/10 | 4 (3, 12) m | No stimulation | N/A | N/A | Exercise training | MAS  FMA-UE  BI | Post-treatment | 5 patients experienced mild local tingling and itching. |
|  | G2: ctDCS | 21/4 | 45 (11) | 15/10 | 6 (3, 36) m | 0.5 mA, electrode size: 18 cm^2^ | Primary sensorimotor cortex of affected cortex | 20 min each session, once a day for 5 days/weeks over 4 weeks | Exercise training |  |  |  |
| Viana, 2014 | G1: Control | 7/3 | 55.0 (12.2) | 10/0 | 35 (20.3) m | Sham stimulation | M1 of affected cortex | 13 min each session, 3 days a week for 5 weeks | Virtual reality | FMA-UE*, WMFT*, MAS, strength, and SSQoL | Post-treatment | No adverse events  were reported by any of the participants. |
|  | G2: atDCS | 9/1 | 56.0 (10.2) | 9/1 | 31.9 (18.2) m | 2 mA, electrode size: 35 cm^2^ |  |  | Virtual reality |  |  |  |
| Wang, 2019 | G1: Control | 21/9 | 50.10 (12.44) | NR | 1.67 (0.91) y | No stimulation | N/A | N/A | Rehabilitation | Brunnstrom stage FMA  MAS | Post-treatment | NR |
|  | G2: atDCS | 16/14 | 53.65 (10.02) | NR | 1.54 (0.82) y | NR | M1 of affected cortex | Once a day for 4 weeks (30 min each time) | Rehabilitation |  |  |  |
| Wu, 2013 | G1: Control | 35/10 | 49.3 (12.6) | 26/19 | 4.9 (2.9) m | Sham stimulation | Primary sensorimotor cortex of affected hemisphere | 20 minutes once daily, 5 days per week, for 4 weeks | Physiotherapy | MAS  FMA-UE  BI | Post-treatment and 4-week follow up | Several subjects felt a slight itching or tingling in the scalp under the electrode. |
|  | G2: ctDCS | 34/11 | 45.9 (11.2) | 27/18 | 4.9 (3.0) m | 1.2 mA, electrode size: 25 cm^2^ |  |  | Physiotherapy |  |  |  |
| Youssef, 2023 | G1: Control | 7/4 | 66.0 (62, 69) | All ischemic strokes | 3~6 months | Sham stimulation | M1 of affected cortex | 3 sessions per week, 12 sessions over 1 month (45 min each time) | Physiotherapy | FMA-UE*  FMA-LE*  MAS  BBS  BDNF | Post-treatment | No adverse events were reported, only participants in tDCS group experienced temporary redness, mild tingling sensation, and slight itching sensation. |
|  | G2: atDCS | 9/4 | 56.0 (47.5, 64) |  |  | 2 mA, electrode size: 49 cm^2^ | M1 of affected cortex |  | Physiotherapy |  |  |  |
|  | G3: dtDCS | 8/3 | 65.0 (54.5, 68) |  |  |  | M1 of the unaffected (cathodal) and affected (anodal) hemisphere |  | Physiotherapy |  |  |  |
| Cheng, 2015 | G1: Control | 11/8 | 57.27 (6.87) | 10/9 | 48.00 (16.45) d | No stimulation | N/A | N/A | Rehabilitation | MAS  Brunnstrom stage  FMA-UE | Post-treatment | NR |
|  | G2: dtDCS | 10/9 | 61.08 (7.34) | 11/8 | 51.12 (19.40) d | 1.2 mA, 0.11 mA/cm^2^ | M1 of the unaffected (cathodal) and affected (anodal) hemisphere | Once a day for 6 days/week over 6 weeks (20 min each session) | Rehabilitation |  |  |  |
| Hu, 2023 | G1: Control | 21/19 | 60.23 (1.74) | 23/17 | 35.23 (1.41) d | No stimulation | N/A | N/A | Rehabilitation | FMA-UE, MAS, mean velocity, maximum velocity, and perfusion index of blood flow | Post-treatment | NR |
|  | G2: dtDCS | 23/17 | 61.45 (1.63) | 21/19 | 36.41 (1.72) d | 1.2 mA | M1 of the unaffected (cathodal) and affected (anodal) hemisphere | Once a day for 5 days/week over 4 weeks (20 min each session) | Rehabilitation |  |  |  |
| Li, 2017 (CHCN) | G1: Control | 9/7 | 72.81 (6.76) | NR | 3~6 months | No stimulation | N/A | N/A | Exercise therapy | MAS  BI | Post-treatment | NR |
|  | G2: ctDCS | 10/11 | 69.76 (7.65) | NR |  | 0.05 mA/cm^2^, 0.06 C/cm^2^, electrode size: 18 cm^2^ | Hand area of M1 of unaffected cortex | Twice a day for 5 days/week over 1 month (20 min each session) | Exercise therapy |  |  |  |
| Liu, 2022 | G1: Control | 32/21 | 56.02 (9.91) | 25/28 | NR | No stimulation | N/A | N/A | Biofeedback therapy | CSI, MAS, FMA-UE, Young's modulus, and serum markers | Post-treatment | No significant difference was found between groups (5 *vs*. 7 cases). |
|  | G2: atDCS | 28/25 | 57.87 (9.98) | 22/31 | NR | 1.0 mA, 0.03~.05 mA/cm^2^ | Upper limb of M1 of the affected side | 5 days/week with an interval of 2 days, for 6 weeks (15~20 min each session) | Biofeedback therapy |  |  |  |
| Pan, 2023 | G1: Control | 27/23 | 69.11 (8.12) | 33/17 | 50.12 (12.25) d | No stimulation | N/A | N/A | Rehabilitation | FMA-UE, ARAT, MAS, NIHSS, BI, SSQoL, serum markers | Post-treatment | 1 case experienced focal itching and 2 cases presented with mild tingling. No serious side effects were observed. |
|  | G2: ctDCS | 26/24 | 68.27 (7.83) | 36/14 | 51.67 (13.89) d | 1.5 mA, electrode size: 35 cm^2^ | M1 of unaffected cortex | 5 times/week, for 4 consecutive weeks | Rehabilitation |  |  |  |
| Yi, 2021 | G1: Control | 30/25 | 48.7 (6.8) | All ischemic strokes | 45.8 (10.6) d | No stimulation | N/A | N/A | Rehabilitation | MAS, FMA-UE, BI, serum markers, and Youth’s modulus | Post-treatment |  |
|  | G2: atDCS | 32/23 | 49.2 (7.0) |  | 46.5 (11.4) d | 2 mA, electrode size: 35 cm^2^ | M1 of affected cortex | Once a day for 5 days/week over 8 weeks (20 min each session) | Rehabilitation |  |  |  |
| Zhao, 2021 | G1: Control | 30/25 | 62.5 (7.9) | All hemorrhagic strokes | 47.8 (14.4) d | No stimulation | N/A | N/A | Medication + rehabilitation | iEMG  ROM  MAS  FMA-UE  BDNF  NGF | Post-treatment | NR |
|  | G2: dtDCS | 28/27 | 61.9 (7.8) |  | 48.2 (14.5) d | 1.2 mA, 0.11 mA/cm^2^ | M1 of the unaffected (cathodal) and affected (anodal) hemisphere | Once a day for 8 weeks (20 min each session) | Medication + rehabilitation |  |  |  |
| Zhao, 2023 | G1: Control | 12/8 | 54.98 (4.32) | 10/10 | 13.65 (2.31) d | No stimulation | N/A | N/A | Rehabilitation | MAS  FMA-UE  BI  fMRI | Post-treatment | NR |
|  | G2: atDCS/  ctDCS | 10/10 | 54.37 (4.54) | 9/11 | 13.87 (2.42) d | 1.0 mA | M1 of the unaffected (cathodal) or affected (anodal) hemisphere | 5 days/week with an interval of 2 days over 4 weeks (20 min each session) | Rehabilitation |  |  |  |
| Zhou JS, 2020 | G1: Control | 32/18 | 55.1 (9.2) | 18/32 | 2.08 (1.15) m | No stimulation | N/A | N/A | Rehabilitation | MAS  FMA-UE  FIM | Post-treatment | NR |
|  | G2: atDCS | 33/17 | 552. (8.4) | 15/35 | 3.14 (1.04) m | 1.0 mA | M1 of affected cortex | Once a day, 20 min each session, 40 sessions in total | Rehabilitation |  |  |  |
| Bakhtiary, 2008 | G1: Control | 20 | 55 (42~65) | NR | NR | No stimulation | N/A | N/A | Physiotherapy | MAS, ROM, strength, H-reflex, and M-wave | Post-treatment | NR |
|  | G2: NMES | 20 |  | NR | NR | 100 Hz, pulse duration 0.1 ms, pulse interval 0.9 ms, at an intensity of 25 % over the intensity of maximum contraction muscle | Tibialis anterior muscle (cathode) and fibula head (anode) | 9 min each session, 20 sessions daily | Physiotherapy |  |  |  |
| Barker, 2008 | G1: Control | 11/2 | 67 (8) | NR | 3.4 (2.6) y | No stimulation | N/A | N/A | Arm training | Motor assessment scale*, strength, MAS, peak isometric force, distance reached, MAS, | Within 3 days after intervention | No adverse effects reported. |
|  | G2: NMES | 6/4 | 61 (16) | NR | 5 (4.9) y | 50 Hz pulse stimulation, 0.2 ms pulse duration, 5-10 sec of contraction and 10-20 sec of rest | Lateral head of triceps brachii | 12 sessions over 4 weeks | Arm training |  |  |  |
| Bauer, 2015 | G1: Control | 9/9 | 64 (11) | 10/8 | 42 (45) d | No stimulation | N/A | N/A | Cycling + rehabilitation | FAC*, performance-oriented mobility assessment*, motricity index, MAS, and 10MWT. | Post-treatment and 2 weeks after intervention | Side-effects were not observed. |
|  | G2: NMES | 12/7 | 59 (14) | 15/4 | 62 (43) d | 25 Hz, 250 μs | Paretic lower limb (m. vastus medialis and rectus of the quadriceps femoris and dorsally on the semitendinosus and biceps femoris) | 19 min each session, 3 times/week, for a total of 12 sessions | Cycling + rehabilitation |  |  |  |
| Boyaci, 2013 | G1: Control | 4/6 | 57.6 (16.4) | 7/3 | 22.1 (24.1) w | Sham stimulation, just above sensory threshold without motor activation | Away from all motor points | 5 sessions/week with 45min of duration over 3 weeks | Neurophysiologic exercise program | FMA-UE, FIM, MAL, MAS, ROM, grip strength, and EMG potentials | 24 h after the end of therapy | Well tolerated and no complications, patients’ complaints, and side effects were recorded. |
|  | G2: NMES | 14/7 | 60.05 (9.04) | 16/5 | 36.00 (19.51) w | 50 Hz, 0.2 ms, 14 sec of contraction or  50 Hz, 0.2 ms | Extensor digitorum communis and extensor carpi ulnaris |  | Neurophysiologic exercise program |  |  |  |
| Chan, 2009 | G1: Control | 6/4 | 45 (16) | NR | 12.1 (11.9) m | At an intensity did not trigger any muscle movement (only had a slight sensation of electric stimulation) | Extensor digitorium superficialis and abductor pollicis longus | 20 min each session, for a total of 15 sessions | Rehabilitation | Functional test for the hemiplegic upper extremity*, FMA-UE*, distance reached*, grip power*, ROM*, FIM, and MAS | Post-treatment | NR |
|  | G2: NMES | 5/5 | 46 (17) | NR | 18.1 (16.1) m | 40 Hz, 0.2 ms |  |  | Rehabilitation |  |  |  |
| Ganesh, 2018 | G1: Control | 17/9 | 58.33 (8.30) | 7/19 | More than 3 months | No stimulation | N/A | N/A | Task‐oriented training | ROM, MAS, and modified emory functional ambulation profile | Post-treatment | NR |
|  | G2: NMES | 37/20 | 56.54 (8.39) | 10/47 |  | 100 Hz, pulse duration 0.1 ms, pulse interval 0.9 ms; 50 Hz, pulse duration 200 ms; at an intensity could produce muscle contraction with participants’ tolerance limit | The anode was placed over the common peroneal nerve and the cathode over the motor point of tibialis anterior | 10 min each session, 5 times/week for 6 weeks | Task‐oriented training |  |  |  |
| Hara, 2008 | G1: Control | 6/4 | 60.5 | NR | 13 m | No stimulation | N/A | N/A | Rehabilitation | RMS  ROM  MAS  Ten-Cup-Moving Test  9HPT | Post-treatment | No adverse effects. |
|  | G2: NMES | 8/2 | 56.0 (24, 77) | NR | 13 (12, 16) m | A train of biphasic rectangular electric impulses via surface electrodes with a pulse width of 50 ms. | extensor carpi radialis longus, extensor carpi radialis brevis, extensor digitorum communis, extensor indicis proprius, and deltoid | 30~60 min each session at home about 6 days/week | Rehabilitation |  |  |  |
| Lee, 2015 | G1: Control | 14/5 | 53.75 (9.11) | 11/8 | 27.95 (16.20) m | Sham stimulation | Paretic arm | 5 days/week for 4 weeks | Rehabilitation | FMA-UE, MAS, WMFT, MAL, and SIS | Post-treatment and 3 months after intervention | NR |
|  | G2: NMES | 15/5 | 54.07 (11.85) | 10/10 | 25.40 (17.09) m | 30 Hz, 0.2 ms | Paretic arm |  | Rehabilitation |  |  |  |
| Lin, 2011 | G1: Control | 11/7 | 66.0 (9.6) | 12/6 | 41.3 (26.5) d | No stimulation | N/A | N/A | Rehabilitation | MAS  FMA-UE  BI | Post-treatment, 1, 3, and 6 months after treatment | NR |
|  | G2: NMES | 11/8 | 62.2 (8.7) | 13/6 | 43.5 (25.2) d | 30 Hz, 0.3 ms | Motor points near the middle of the supraspinatus muscle and the deltoid muscle on the paretic side, as well as over the wrist extensor | 30 min, 5 days a week for 3 weeks | Rehabilitation |  |  |  |
| Mangold, 2009 | G1: Control | 7/4 | 62 (16.2) | 9/2 | 7.3 (5.8, 8.2) w | No stimulation | N/A | N/A | Occupational therapy | BI, Chedoke Mcmaster stroke assessment, and MAS | Post-treatment | NR |
|  | G2: NMES | 10/2 | 57.5 (16.7) | 10/2 | 6.7 (6.4, 7.3) w | 25 Hz, the pulse width varied between 0 and 250 microseconds to achieve controlled muscle contraction | Proximal muscles (eg, anterior deltoid muscle, m. triceps brachii) and distal muscles (finger extensors and finger flexors) | 45 min each session, 3-5 sessions/week of 45 min over 4 weeks, 12 sessions in total | Occupational therapy |  |  |  |
| Mesci, 2009 | G1: Control | 11/9 | 59.10 (8.58) | 11/4/5 | 7.30 (4.42) m | No stimulation | N/A | N/A | Exercise | ROM, MAS, Brunnstrom stage, FIM, Rivermead motor assessment, and FAC | Post-treatment | NR |
|  | G2: NMES | 12/8 | 62.65 (7.52) | 8/4/8 | 9.45 (4.80) m | 50 Hz, 0.4 ms | Foot dorsiflexors | 20 min each session, 5 days a week as 1 session per day for a total of 20 sessions | Exercise |  |  |  |
| Nakipoğlu Yuzer, 2017 | G1: Control | 9/6 | 57.66 (10.63) | 13/2 | 4.86 (1.49) m | No stimulation | N/A | N/A | None | ROM, MAS, Rivermead motor assessment, Brunnstrom stage, BI, and UEFT | Post-treatment | NR |
|  | G2: NMES | 8/7 | 60.20 (12.20) | 11/4 | 4.60 (1.33) m | 30 Hz, 0.3 ms | Motor points of the extensor carpi radialis longus, extensor carpi radialis brevis, extensor carpi ulnaris, and extensor digitorum communis | 30 min a day for 5 days a week for a total of 20 sessions per patient. | None |  |  |  |
| Sabut, 2011 | G1: Control | 2/14 | 50.1 (10.4) | NR | 18.2 (11.8) m | No stimulation | N/A | N/A | Rehabilitation | MAS. Strength, ROM, FMA-LE | Post-treatment | NR |
|  | G2: NMES | 4/12 | 49.1 (8.8) | NR | 17.3 (18.8) m | 35 Hz, 0.28 ms | Common peroneal nerve | 1 hr per day, 5 days a week, for 12 weeks | Rehabilitation |  |  |  |
| Sahin, 2012 | G1: Control | 12/9 | 59.3 (9.3) | NR | 35.1 (24.4) m | No stimulation | N/A | N/A | PNF | MAS, F_max_/M_max_, ROM, FIM, and Brunnstrom stage | Post-treatment | NR |
|  | G2: NMES | 11/10 | 60.2 (6.2) | NR | 25.0 (14.6) m | 100 Hz, with a pulse duration of 0.1 ms  and pulse intervals of 0.9 ms, in cycles of 3 ms, and a resting duration of 9 seconds | Cathode was placed on the most excitable region of the muscle and anode was placed on a region close to the lateral epicondyle | 15 min each session, 5 days a week, 20 sessions in total | PNF |  |  |  |
| Sentandreu-Mañó, 2021 | G1: Control | 12/8 | 71.50 (7.56) | 16/4 | 5.8 (3.24) m | No stimulation | N/A | N/A | Physiotherapy | ROM, strength, MAS, EMG activity | Post-treatment and 1 month after treatment | No adverse events related to the trial. |
|  | G2: NMES | 24/17 | 70.68 (7.06) | 28/13 | 5.76 (3.17) m | 50 Hz/ 35 Hz, 0.3 ms | Extensor muscles of the wrist and fingers, | 20 min for the first 2 sessions and 30 min for subsequent sessions, 3 days per week, a total of 24 sessions | Physiotherapy |  |  |  |
| Xu, 2017 | G1: Control | 16/7 | 53.7 (8.98) | 18/5 | 42.76 (5.65) d | No stimulation | N/A | N/A | Rehabilitation +  mirror therapy | 10MWT*, Brunnstrom stage*, MAS, and ROM | Post-treatment | NR |
|  | G2: NMES | 16/7 | 55.00 (10.98) | 16/7 | 43.25 (5.95) d | 50 Hz, 10 mA | Common peroneal nerve and midpoint of the anterior tibialis muscle of the affected leg | 30 min each session, 5 days/week for 4 weeks | Rehabilitation +  mirror therapy |  |  |  |
| Yang, 2018 | G1: Control | 6/2 | 50.8 (3.8) | 4/4 | 31.8 (6.1) m | No stimulation | N/A | N/A | ROM, stretching, and ambulation training | Gait analysis*, MAS, EMG, lengthening velocity, maximal position, and strength | Within 7 days after treatment | NR |
|  | G2: NMES | 15/2 | 53.04 (4.37) | 10/7 | 44.72 (8.47) m | 50 Hz with a 0.2 ms pulse width | Tibialis anterior or medial gastrocnemius | 20 min each time, 3 times per week for 7 weeks w | Ambulation training |  |  |  |
| Zhou, 2018 | G1: Control | 15/3 | 63.78 (11.17) | 12/6 | 105.89 (142.80) d | No stimulation | N/A | N/A | Rehabilitation | Numerical rating scale*, ROM, FMA-UE, MAS, BI, and SSQoL | 2 and 4 weeks after 4 weeks of treatment | No adverse events were observed. |
|  | G2: NMES | 21/10 | 59.36 (10.78) | 19/12 | 73.61 (53.40) d | 15 Hz, 0.2 ms | Supraspinatus and deltoids | 20 sessions of 20 sessions of 1-hr stimulation for 4 weeks, consecutively | Rehabilitation |  |  |  |
|  | G3: TENS | 26/6 | 58.50 (9.07) | 13/19 | 100.88 (103.32) d | 100 Hz, 0.1 ms | Supraspinatus and deltoids |  | Rehabilitation |  |  |  |
| Bu, 2022 | G1: Control | 27/13 | 58.24 (3.28) | All ischemic strokes | 4.19 (0.40) m | No stimulation | N/A | N/A | Rehabilitation | FMA  MAS | Post-treatment | NR |
|  | G2: NMES | 25/15 | 58.65 (3.45) | All ischemic strokes | 4.25 (0.36) m | 20~30 Hz | Upper and lower limbs | 1 time per day, 5 times/week for 3 months | Rehabilitation |  |  |  |
| Chen, 2015 | G1: Control | 9/6 | 50.00 (11.31) | 9/6 | 59.27 (41.23) d | No stimulation | N/A | N/A | Medication + rehabilitation | TUG, 10MWT, 6MWT, MAS, and Holden walk ability, | Post-treatment | NR |
|  | G2: NMES | 17/13 | 51 (9.82) | 17/13 | 66.27 (43.78) d | NR | Common peroneal nerve | 20 min each time, 1/2 times per day, for 2 weeks | Medication + rehabilitation |  |  |  |
| Chen, 2021 (JQN) | G1: Control | 23/19 | 63.06 (9.15) | 32/10 | 3.12 (0.63) m | No stimulation | N/A | N/A | Usual care | MAS  CSI  FMA | Post-treatment and 2 months after intervention | NR |
|  | G2: NMES | 25/17 | 63.03 (9.08) | 34/8 | 3.03 (0.53) m | 30 Hz, 20~30 mA, 0.3 ms | Extensor wrist, triceps brachii, tibialis anterior, and hamstring of the affected side | 20 min each time, 1/2 times per day, for 30 days | Usual care |  |  |  |
| Huang, 2022 | G1: Control | 16/7 | 58.13 (9.46) | 17/6 | 60.34 (14.73) d | Sham stimulation | Motor points of the tibialis anterior, quadriceps, gastrocnemius, and hamstring | 20 min each time, 1 time per day, 6 days/week, for 4 weeks | Usual therapy | MAS, FMA-LE, 10MWT, RMS, and iEMG | Post-treatment | NR |
|  | G2: NMES | 15/7 | 58.82 (11.12) | 15/7 | 59.77 (14.65) d | 30 Hz, 0.2 ms |  |  | Usual therapy |  |  |  |
| Jiang, 2020 | G1: LFrTMS | 10/4 | 56.36 (16.23) | All ischemic strokes | 2.66 (1.30) m | 1 Hz, 90% RMT, 1000 pulses | M1 of unaffected hemisphere | 21 min each session, once per day, 5 days/week, for 8 consecutive weeks | Rehabilitation | BI  MAS  FMA-UE  MEP | Post-treatment | NR |
|  | G2: NMES | 9/5 | 52.50 (13.69) | All ischemic strokes | 3.19 (1.79) m | NR | The tendon of the biceps brachii and the belly of triceps brachii | 15 min each session, once per day, 5 days/week, for 8 consecutive weeks | rehabilitation |  |  |  |
| Liu, 2019 (JBBMC) | G1: Control | 16/15 | 52.81 (11.25) | NR | 58.58 (17.86) d | No stimulation | N/A | N/A | Rehabilitation | MAS  FMA-LE  BI | Post-treatment | NR |
|  | G2: NMES | 17/14 | 51.74 (13.32) | NR | 54.10 (13.30) d | 0.1~0.5 Hz, 0.1~2.0 ms | Gastrocnemius and tibialis anterior | 20 min, 1 time/day, 6 days/week, for 8 weeks | Rehabilitation |  |  |  |
| Meng, 2009 | G1: Control | 48 | 65 (7) | 31/17 | 22 d | No stimulation | N/A | N/A | Comprehensive therapy | MAS  Brunnstrom stage  BI | Post-treatment | NR |
|  | G2: NMES | 48 |  | 32/16 | 22 d | 30 Hz, 0.2 ms | Antagonist of upper and lower limb | 20 min, each time, 2 times/day, 6 days/week, for 1 months | Comprehensive therapy |  |  |  |
| Song, 2014 | G1: Control | 20/15 | 61.9 (9.1) | NR | NR | No stimulation | N/A | N/A | Physiotherapy | MAS  FMA  BI | Post-treatment | No significant difference between groups was found. |
|  | G2: NMES | 19/12 | 60.7 (8.2) | NR | NR | 0.5~150 Hz, 100 ms | Triceps brachii, extensor carpi muscles, hamstring, and anterior tibial of the affected side | 25 min each time, for 3 months | Physiotherapy |  |  |  |
| Wang, 2016 (CR) | G1: Control | 15/2 | 56.76 (11.25) | 13/4 | 18.35 (7.04) w | No stimulation | N/A | N/A | Rehabilitation | FMA-LE  TUG  Strength  MAS  Gait analysis | Post-treatment | NR |
|  | G2: NMES | 13/4 | 54.41 (10.93) | 11/6 | 17.11 (8.12) w | 35 Hz, 0.1 ms | Lower limb | 20 min each time, 1 time/day, 5 day/week for 4 weeks | Rehabilitation |  |  |  |
| Wang, 2018 | G1: Control | 47/29 | 60.0 (6.2) | NR | NR | No stimulation | N/A | N/A | Baclofen | MAS, FMA, BI | Post-treatment | No significant difference between groups was found. |
|  | G2: NMES | 49/27 | 61.4 (4.8) | NR | NR | 100~150 Hz, 100 ms, 1000 pulses | Upper and lower limb | 25~30 min/time, 1 time/day, for 3 months | Baclofen |  |  |  |
| Yang, 2023 (JMMH) | G1: Control | 11/9 | 61.20 (6.20) | 10/10 | 51.35 (15.01) d | No stimulation | N/A | N/A | PNF + Usual therapy | MAS  FMA-UE  BI | Post-treatment and 2 weeks after treatment | NR |
|  | G2: NMES | 9/11 | 62.70 (4.91) | 11/9 | 50.50 (21.28) d | 25 Hz, 0.25 ms, 20~30 mA | Upper part of body | 30 min/time, 1 time/day, 6 days/week, for 4 weeks | PNF + Usual therapy |  |  |  |
| Yu, 2022 | G1: Control | 17/16 | 59.80 (6.50) | NR | 4.50 (1.50) w | No stimulation | N/A | N/A | Rehabilitation | MAS  FMA-LE  BBS  10MWT  ROM  Foot inversion symptom score | Post-treatment | NR |
|  | G2: NMES | 20/13 | 59.50 (5.20) | NR | 4.30 (1.70) w | 0.8 Hz, 20~30 mA | Motor points of peroneal longus, peroneal brevis, and third peroneal of affected side | 20 min/time, 1 time/day, 5 days/week, for 4 weeks | Rehabilitation |  |  |  |
| Zhang, 2018 | G1: Control | 14/6 | 64.53 (9.89) | 15/5 | 70.31 (15.55) d | No stimulation | N/A | N/A | Rehabilitation | FMA-UE, simple test for evaluating hand function, BI, and MAS | Post-treatment | NR |
|  | G2: NMES | 12/8 | 63.75 (11.27) | 14/6 | 67.64 (14.95) d | 50 Hz, 0.3 ms, 0~100 mA | Upper limb | 20 min/time, 1 time/day, 5 days/week, for 4 weeks | Rehabilitation |  |  |  |
| Zhou, 2023 | G1: Control | 16/4 | 66.9 (4.7) | 10/10 | 79.45 (32.44) d | No stimulation | N/A | N/A | Usual therapy | FMA-UE. MAS, iEMG, RMS, and BI | Post-treatment | NR |
|  | G2: NMES | 15/5 | 66.4 (5.3) | 9/11 | 81.10 (39.17) d | 20~45 Hz, 0.1~0.3 ms | Upper limb | 30 min/time, 1 time/day, 5 days/week, for 8 weeks | Usual therapy |  |  |  |
| Zhu, 2019 | G1: Control | 23/7 | 65.2 (8.5) | 19/11 | 32.1 (3.1) d | No stimulation | N/A | N/A | Catgut implantation + rehabilitation | MAS  FAM-UE  RMS | Post-treatment | No obvious side effects were recorded. |
|  | G2: NMES | 26/4 | 65.6 (8.0) | 21/9 | 31.5 (3.3) d | 0.5~5 Hz, 10 ms, 0~100 mA | Triceps brachii and extensor carpi | 25 min/time, 1 time/day, 5 days/week with an interval of 2 days, for 6 weeks | Catgut implantation + rehabilitation |  |  |  |
| Chen, 2005 | G1: Control | 12 | 57 (41, 69) | NR | 12~35 m | Sham stimulation (0 mA) | Gastrocnemius and Achilles tendon | 20 min/time, 1 time/day, 6 days/week for 1 month | Medication | MAS, F_max_/M_max_, H-reflex latency, H-reflex recovery curves, and 10MWT | Post-treatment | NR |
|  | G2: TENS | 12 |  | NR |  | 20 Hz, 0.2 ms |  |  | Medication |  |  |  |
| Kim, 2013 | G1: Control | 8/7 | 61.3 (9.97) | NR | 12.3 (5.26) m | Sham stimulation | Muscle belly of triceps and wrist extensors | 30 min/time, 1 time/day, 5 days/week for 4 months | Task-related training | FMA-UE, manual function test, BBT, and MAS | 1 day after treatment | NR |
|  | G2: TENS | 9/9 | 63.3 (8.30) | NR | 13.6 (4.36) m | 100 Hz, 0.2 ms, 2~3 times the sensory threshold |  |  | Task-related training |  |  |  |
| Park, 2014 | G1: Control | 8/6 | 71.14 (3.82) | NR | 18.57 (1.74) m | Sham stimulation | Lateral and medial quadriceps and gastrocnemius | 30 min/time, 1 time/day, 5 days/week for 6 weeks | Exercise | MAS, balance, TUG, and gait analysis | 1 weeks after intervention | NR |
|  | G2: TENS | 12/3 | 71.20 (3.46) | NR | 18.66 (2.46) m | 100 Hz, 0.2 ms |  |  | Exercise |  |  |  |
| Sonde, 1998 | G1: Control | 8/10 | 73 (3.5) | NR | 8.3 (2.1) m | No stimulation | N/A | N/A | Physiotherapy | FMA-UE  MAS  VAS  BI  ROM | Post-treatment and 3 months after treatment | NR |
|  | G2: TENS | 19/7 | 71 (6.0) | NR | 9.1 (2.2) m | 1.7 Hz, 14 ms | Wrist extensor (80% also at elbow extensors or shoulder abductors) | 60 min/time, 1 time/day, 5 days/week for 3 months | Physiotherapy |  |  |  |
| Tekeoğlu, 1998 | G1: Control | 14/16 | 52.2 (5.4) | NR | 44.3 (13.1) d | Sham stimulation | Musculus triceps brachii and common peroneal nerve posterior to head of the fibula | 30 min/time, 1 time/day, Monday to Friday, for 8 weeks | Exercise | BI  MAS | Post-treatment | NR |
|  | G2: TENS | 17/13 | 55.9 (7.0) | NR | 40.8 (11.4) d | 100 Hz, 0.2 ms |  |  | Exercise |  |  |  |
| Zhang, 2023 | G1: Control | 30 | 18~75 | NR | NR | Sham stimulation | Mid-belly of triceps brachii | 15 min/time, 1 time/day, 6 days/week, for 4 weeks | Usual care | MAS, FMA-UE, iEMG, and BI | Post-treatment | NR |
|  | G2: rPMS | 30 |  | NR | NR | 20 Hz |  |  | Usual care |  |  |  |
| Fujimura, 2024 | G1: Control | 17/7 | 61 (15) | 11/13 | 41 (20) d | No stimulation | N/A | N/A | Conventional rehabilitation | Acromio-humeral interval*, shoulder pain, MAS, ROM, FMA-UE | Post-treatment and 6 weeks after treatment | No obvious side effects were recorded. |
|  | G2: rPMS | 14/8 | 69 (13) | 12/10 | 34 (23) d | 2 s at 30 Hz with a 3-s off time, 0.35 ms | Supraspinatus and posterior deltoid/  infraspinatus | 17 min/time, for 6 weeks | Conventional rehabilitation |  |  |  |
| Liu, 2024 | G1: Control | 21/21 | 64.83 (8.71) | NR | 7.76 (2.35) m | No injection | N/A | N/A | Sit-to-stand training | MAS  FMA-LE  BBS  BI  SSQoL | Post-treatment and 4 and 8 weeks after treatment | NR |
|  | G2: BoNT | 20/22 | 62.64 (9.64) | NR | 8.07 (2.04) m | BoNTA: 2 to 3 site, maximum dose 100 U for each muscle | Lower limb | Electrostimulation guidance was used for the injections | Sit-to-stand training |  |  |  |
| Abdelkader, 2024 | G1: Control | 6/4 | 51.8 (15.7) | 6/4 | 5.6 (6.6) w | Sham stimulation | The coil perpendicular to the scalp hot spot | Five consecutives daily rTMS sessions | Conventional and task-oriented therapy | FMA  WFMT  MAS | Post-treatment and 4 weeks later | NR |
|  | G2: HFrTMS | 9/6 | 57.2 (16.0) | 11/4 | 3.87 (1.8) w | 5 Hz, 90% RMT | M1 of affected hemisphere |  |  |  |  |  |
|  | G3: LFrTMS | 10/5 | 52.3 (15.0) | 9/6 | 7.67 (8.5) w | 1 Hz, 90% RMT | M1 of unaffected hemisphere |  |  |  |  |  |

**Abbreviations:** ADL, Activities of daily living; AMT, Active motor threshold; AQoL, Assessment of Quality of Life scale; ARAT, Action Research Arm Test; atDCS, Anodal transcranial current stimulation; BBS, Berg balance scale; BBT, Box and block test; BDNF, Brain-derived neurotrophic factor; BI, Barthel index; BoNTA, Botulinum toxin type A; CMCT, central motor conduction time; CSI, Clinic spasticity index; cTBS, Continuous theta burst stimulation; ctDCS, Cathodal transcranial current stimulation; dtDCS, Dual transcranial current stimulation; EHL, extensor hallucis longus; EQ-5D, EuroQol-5 dimensions; FAC, Functional ambulation category scale; FCU, flexor carpi ulnaris; FCR, flexor carpi radialis; FDL, Flexor digitorum longus; FDP, flexor digitorum profundus; FDS, flexor digitorum superficialis; FHL, flexor hallucis longus; FIM, Functional independence measure; FMA, Fugl-Meyer assessment; FMA-UE, Fugl-Meyer assessment-upper extremity; FMA-LE, Fugl-Meyer assessment-lower extremity; FPL, flexor pollicis longus; GMFM, Gross motor function measure; HFrTMS, High-frequency repetitive transcranial magnetic stimulation; HRSD, Hamilton rating scale for depression; iEMG, Integrated electromyogram; iTBS, Intermittent theta burst stimulation; LFrTMS, low-frequency repetitive transcranial magnetic stimulation; MAL, Quality of movement and amount of use; MAS, Modified Ashworth scale; MEP, motor evoked potential; MMSE, Mini-mental status examination; MRC, Medical research council; MRP, Motor relearning program; MTS, Modified Tardieu scale; MVG, maximum voluntary grip strength; N/A, Not applicable; NGF, Neural growth factor; NHPT, Nine hole peg test; NIHSS, National Institute of Health stroke scale; NMES, Neuromuscular electrical stimulation; NR, Not reported; ORS, ordinal rating score the difficulties encountered in performing functional activities; PNF, Proprioceptive Neuromuscular Facilitation; REPAS, resistance to passive movement scale; RMA, Rivemead Motor Assessment; RMS, Root mean square; RMT, Resting motor threshold; ROM, Range of motion; rPMS, Repetitive peripheral magnetic stimulation; SAS, Self-rating anxiety scale; SDS, Self-rating depression scale; SIS, Stroke impact scale; SSQoL, stroke specific quality of life; TENS, Transcutaneous electrical nerve stimulation; TUG, Timed up and go; UEFT, Upper extremities function test; VAS, Visual analog scale; WMFT, Wolf motor function test; 6MWT, 6-minute walk test; 10MWT, 10-minute walk test;

* Indicates primary outcome.

# Appendix 8. Risk of bias graph summary of the included studies.


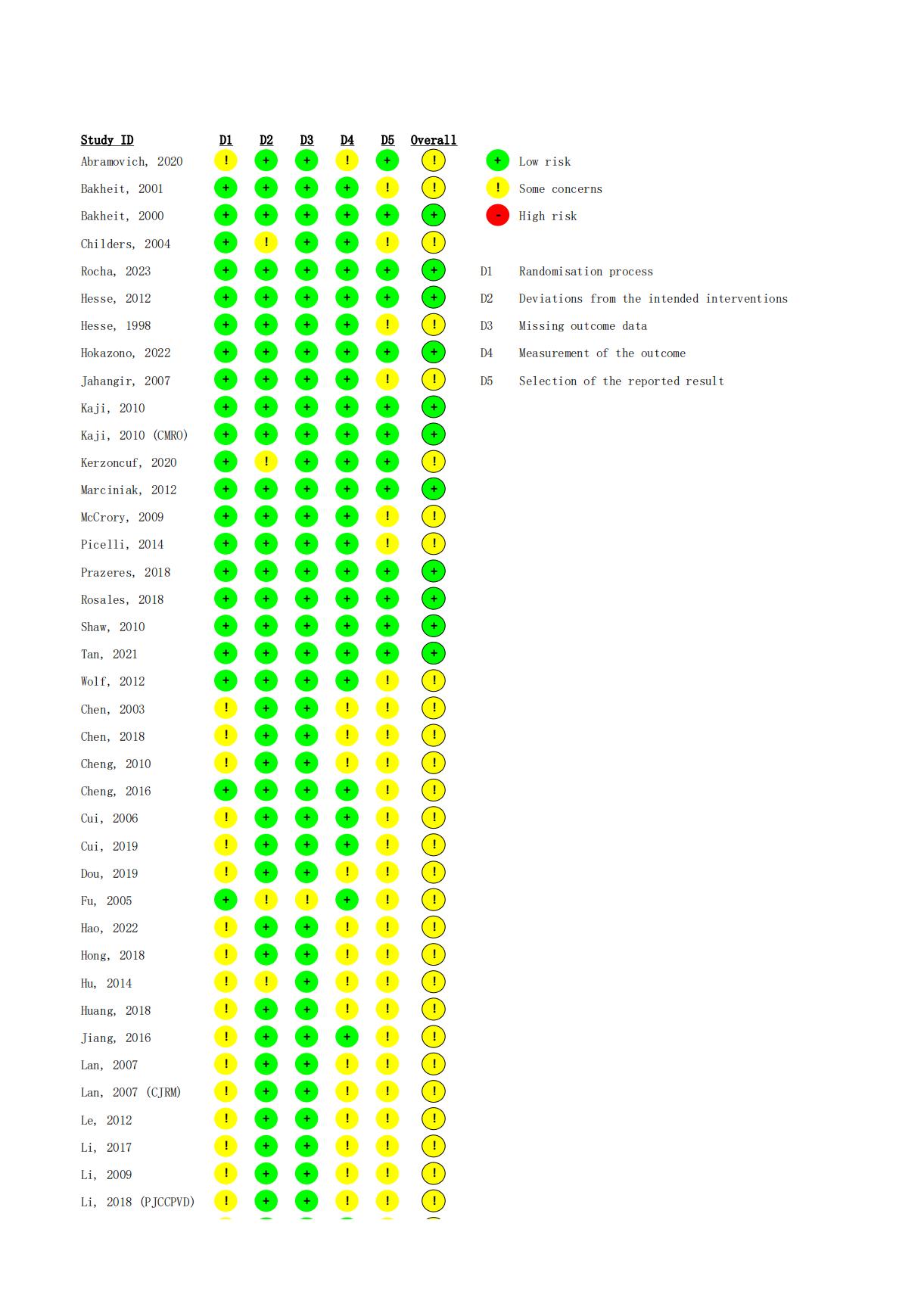

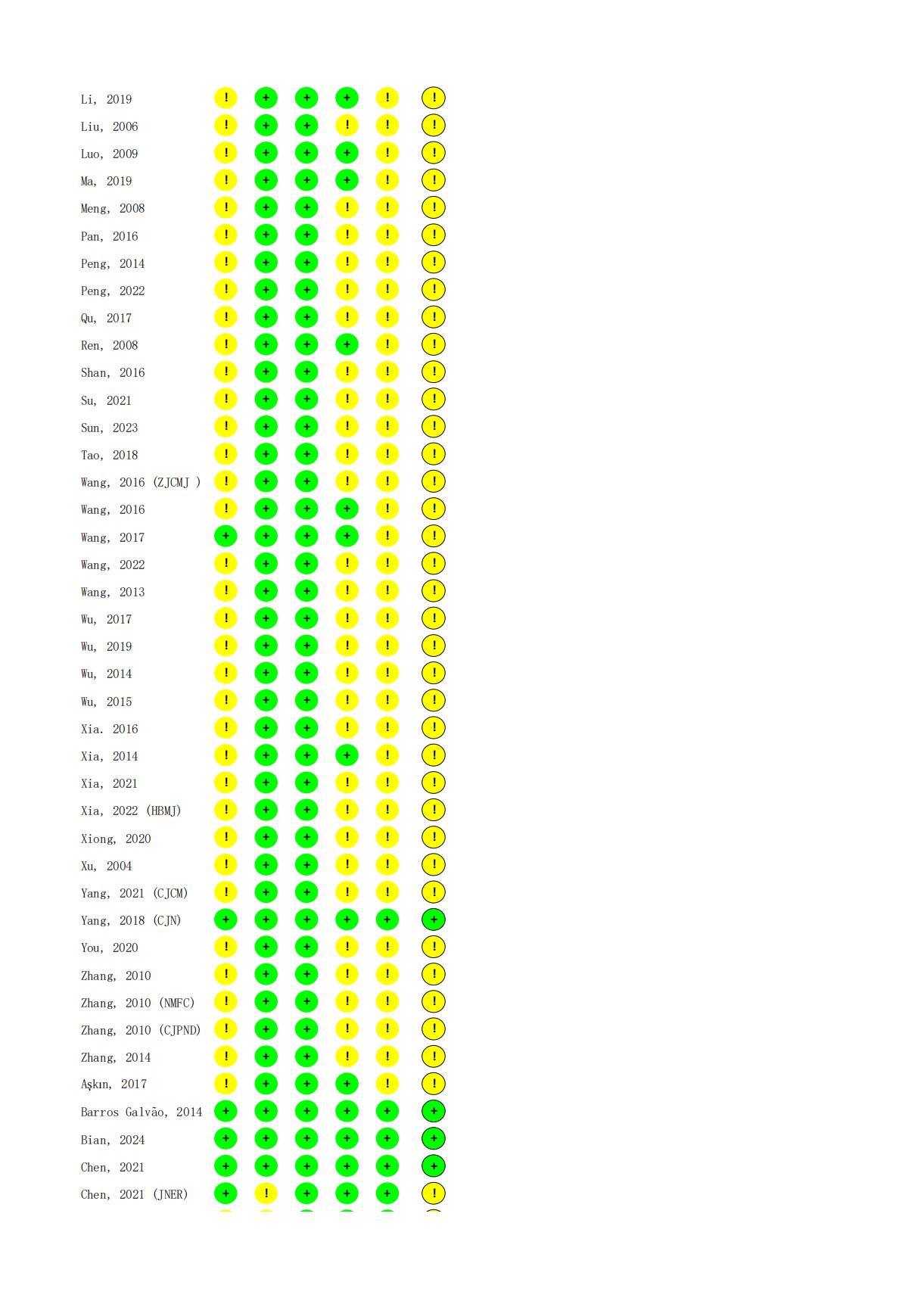


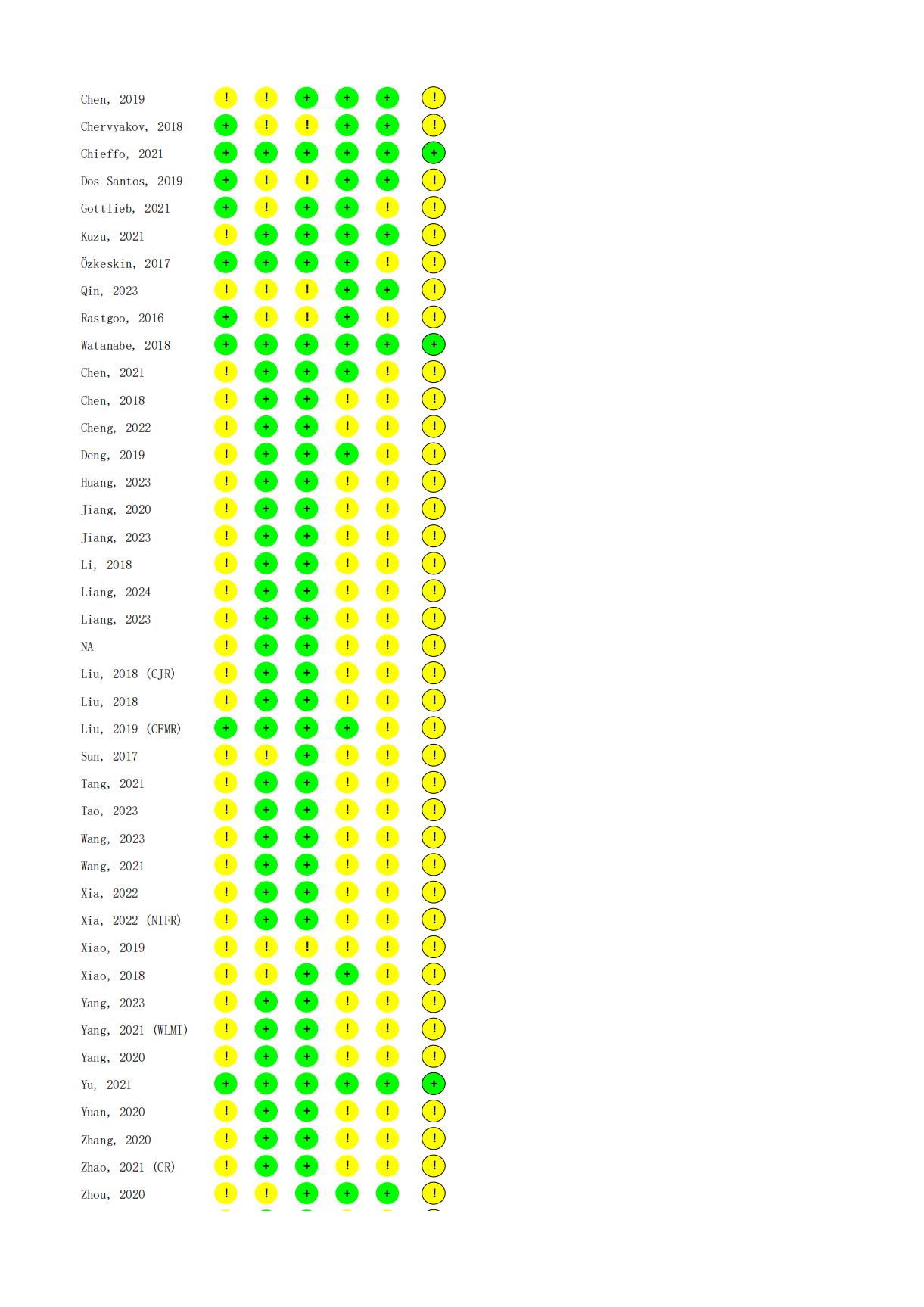


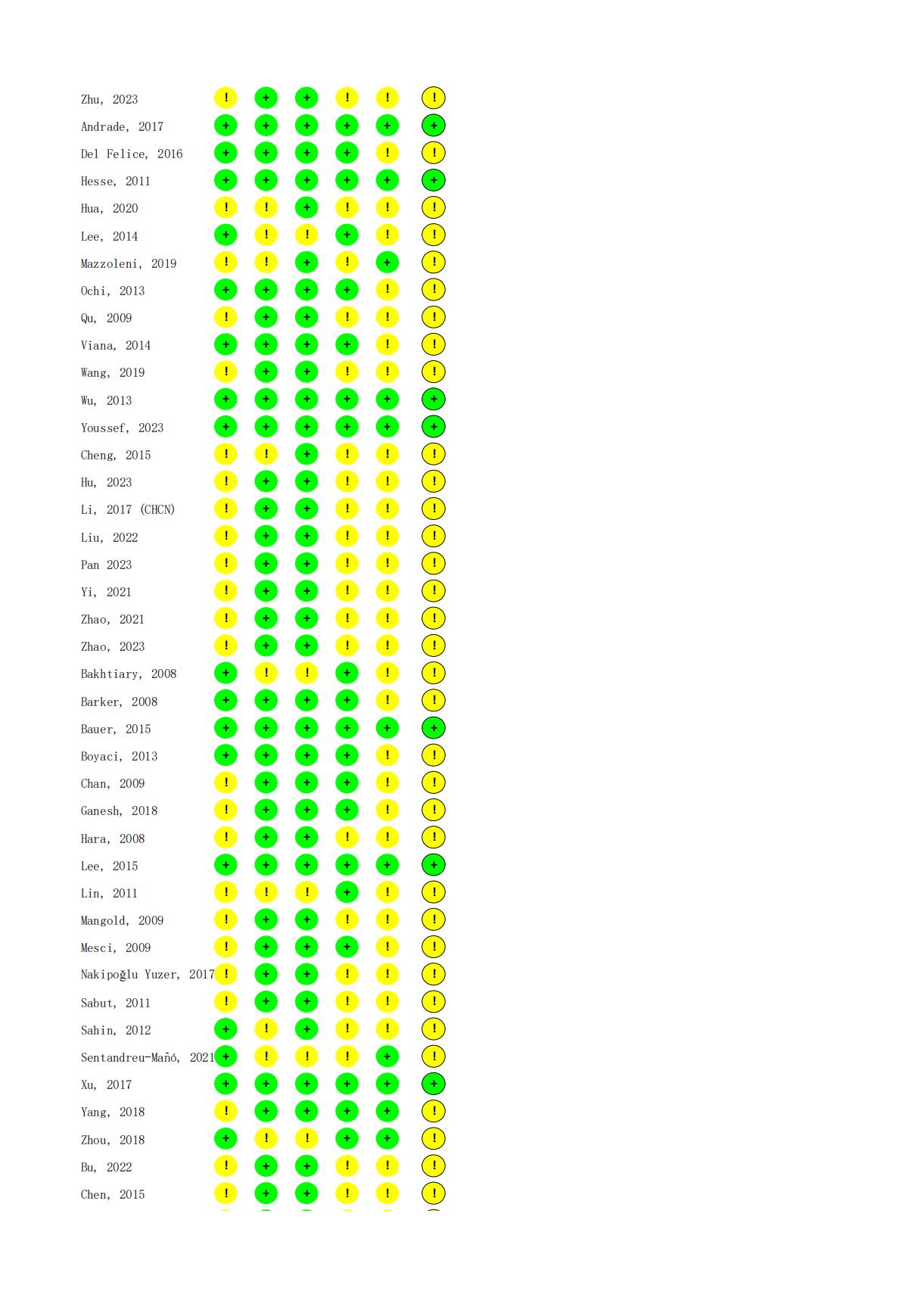


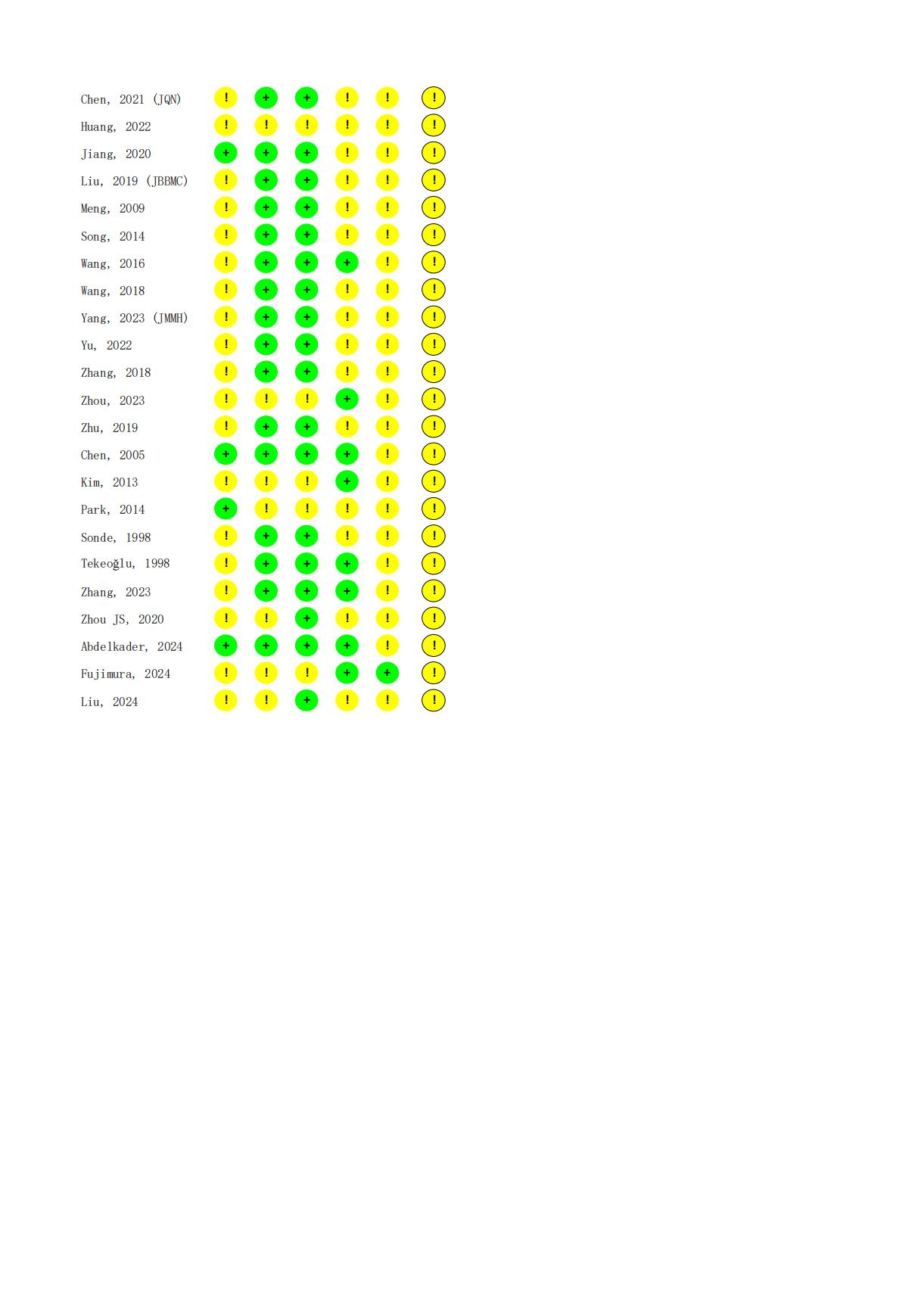


# Appendix 9. Assessment of transitivity assumption.

We assessed transitivity by assessing whether the synthesis of the direct comparisons of interventions was performed in comparable baseline clinical characteristics (age, sex proportion, ischemic stroke proportion, time since stroke, and baseline MAS value), considering intervention node, pairwise comparisons, and each network analysis (each outcome at each follow-up).

## 9.1 Studies and participants characteristics by interventions

The following characteristics have been evaluated in all trials included in the network irrespectively of the outcome being reported.

**A.** Age


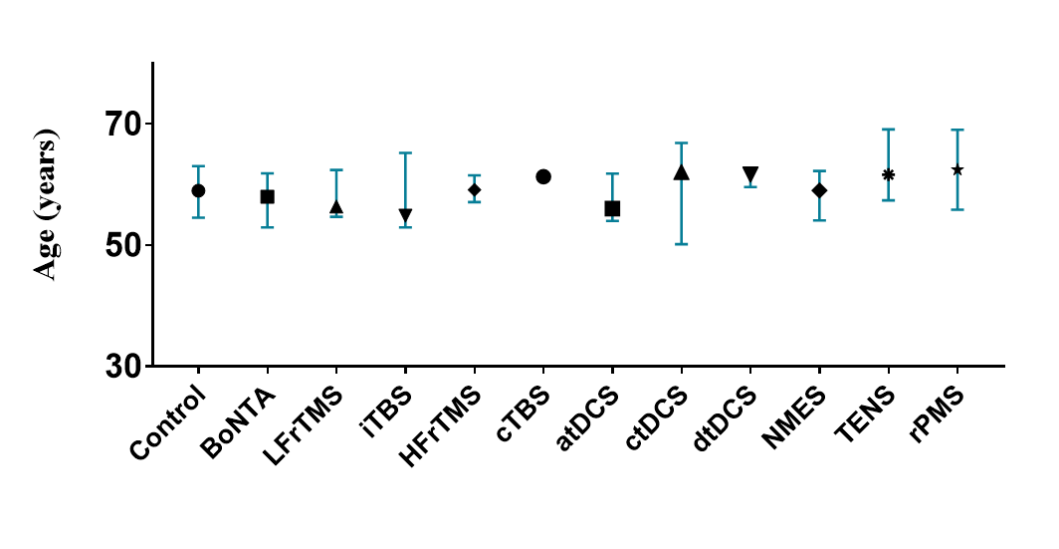


The transitivity regarding the age of participants based on intervention nodes appears acceptable, as there is an overlap of characteristics across different nodes, suggesting no significant differences among treatment comparisons (P=0.50).

Data are expressed as median (IQR). Kruskal-Wallis test was used for statistical analysis.

**Abbreviations:** BoNTA=botulinum toxin type A; LFrTMS=low-frequency transcranial magnetic stimulation; iTBS= intermittent theta-burst stimulation; HFrTMS = high-frequency transcranial magnetic stimulation; cTBS=continuous theta-burst stimulation; atDCS=anodal transcranial direct current stimulation; ctDCS=cathodal transcranial direct current stimulation; dtDCS=dual transcranial direct current stimulation; NMES=neuromuscular electrical stimulation; TENS=transcutaneous electrical nerve stimulation; rPMS= repetitive peripheral magnetic stimulation.

**B.** Percentage of male


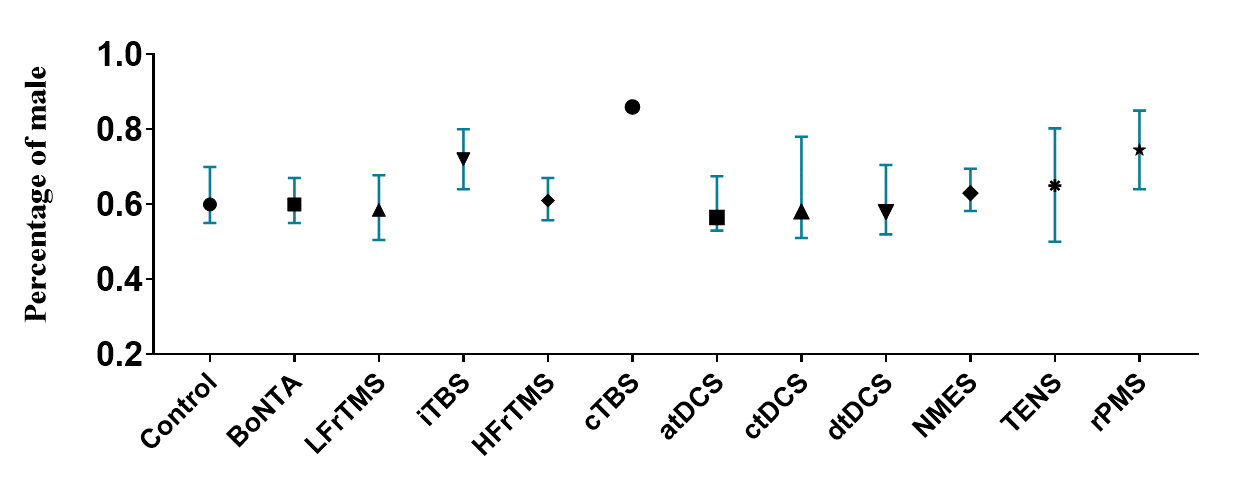


The transitivity regarding the percentage of male based on intervention nodes appears acceptable, as there is an overlap of characteristics across different nodes, suggesting no significant differences among treatment comparisons (P=0.12).

Data are expressed as median (IQR). Kruskal-Wallis test was used for statistical analysis.

**Abbreviations:** BoNTA=botulinum toxin type A; LFrTMS=low-frequency transcranial magnetic stimulation; iTBS= intermittent theta-burst stimulation; HFrTMS = high-frequency transcranial magnetic stimulation; cTBS=continuous theta-burst stimulation; atDCS=anodal transcranial direct current stimulation; ctDCS=cathodal transcranial direct current stimulation; dtDCS=dual transcranial direct current stimulation; NMES=neuromuscular electrical stimulation; TENS=transcutaneous electrical nerve stimulation; rPMS= repetitive peripheral magnetic stimulation.

**C.** Percentage of ischemic stroke


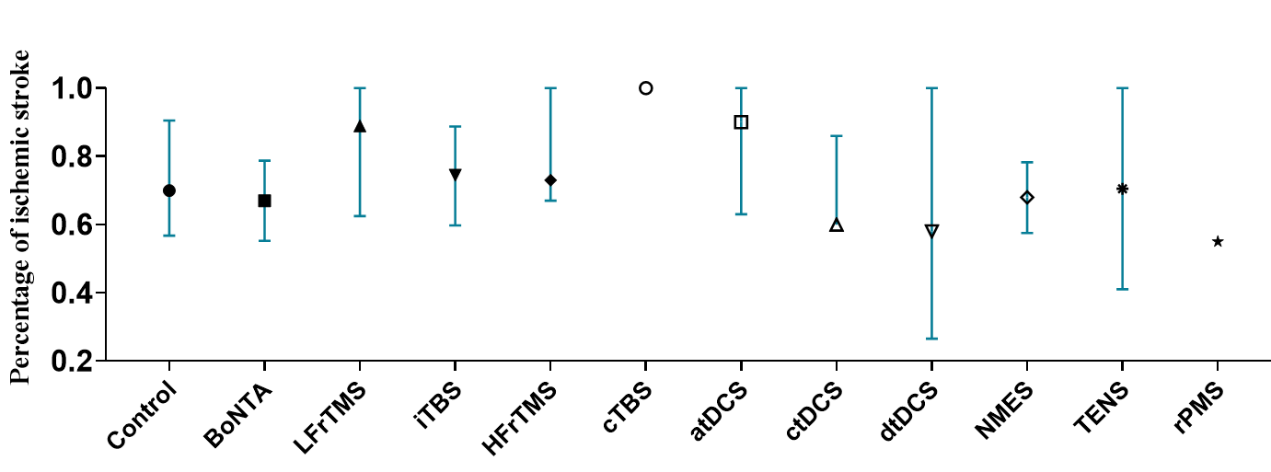


The transitivity regarding the percentage of ischemic stroke based on intervention nodes appears acceptable, as there is an overlap of characteristics across different nodes, suggesting no significant differences among treatment comparisons (P=0.16).

Data are expressed as median (IQR). Kruskal-Wallis test was used for statistical analysis.

**Abbreviations:** BoNTA=botulinum toxin type A; LFrTMS=low-frequency transcranial magnetic stimulation; iTBS= intermittent theta-burst stimulation; HFrTMS = high-frequency transcranial magnetic stimulation; cTBS=continuous theta-burst stimulation; atDCS=anodal transcranial direct current stimulation; ctDCS=cathodal transcranial direct current stimulation; dtDCS=dual transcranial direct current stimulation; NMES=neuromuscular electrical stimulation; TENS=transcutaneous electrical nerve stimulation; rPMS= repetitive peripheral magnetic stimulation.

**D.** Mean time since stroke


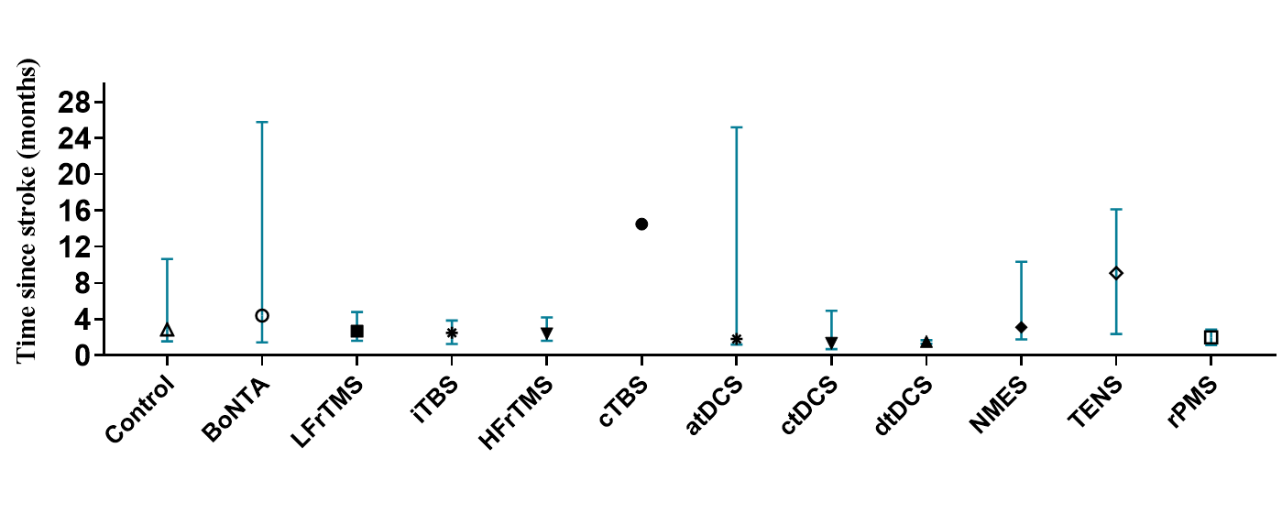
 The transitivity regarding time since stroke based on intervention nodes appears acceptable, as there is an overlap of characteristics across different nodes, suggesting no significant differences among treatment comparisons (P=0.42).

Data are expressed as median (IQR). Kruskal-Wallis test was used for statistical analysis.

**Abbreviations:** BoNTA=botulinum toxin type A; LFrTMS=low-frequency transcranial magnetic stimulation; iTBS= intermittent theta-burst stimulation; HFrTMS = high-frequency transcranial magnetic stimulation; cTBS=continuous theta-burst stimulation; atDCS=anodal transcranial direct current stimulation; ctDCS=cathodal transcranial direct current stimulation; dtDCS=dual transcranial direct current stimulation; NMES=neuromuscular electrical stimulation; TENS=transcutaneous electrical nerve stimulation; rPMS= repetitive peripheral magnetic stimulation.

**E.** Baseline mean MAS value


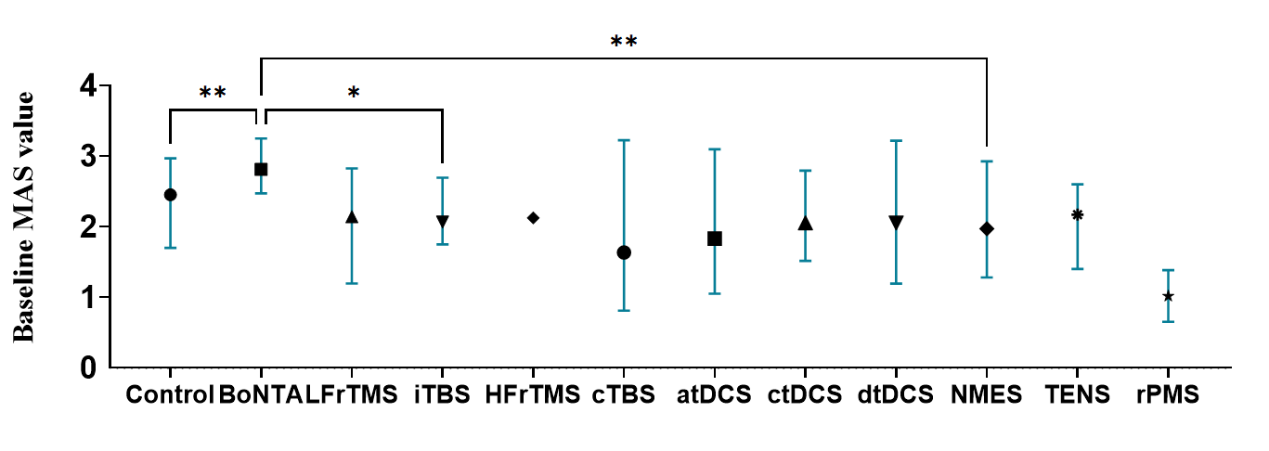
 Concerns about transitivity arise in the comparisons of BoNTA with Control, BoNTA with iTBS, and BoNTA with NMES, due to variations in the baseline MAS value. However, these observations stem from analysis based on intervention nodes of all studies included, which does not influence pairwise and network meta-analyses.

Data are expressed as median (IQR). Kruskal-Wallis test was used for statistical analysis. BoNTA vs Control (P=0.0020), BoNTA vs iTBS (P=0.010), BoNTA vs NMES (P=0.0062).

**Abbreviations:** BoNTA=botulinum toxin type A; LFrTMS=low-frequency transcranial magnetic stimulation; iTBS= intermittent theta-burst stimulation; HFrTMS = high-frequency transcranial magnetic stimulation; cTBS=continuous theta-burst stimulation; atDCS=anodal transcranial direct current stimulation; ctDCS=cathodal transcranial direct current stimulation; dtDCS=dual transcranial direct current stimulation; NMES=neuromuscular electrical stimulation; TENS=transcutaneous electrical nerve stimulation; rPMS= repetitive peripheral magnetic stimulation.

## 9.2 Studies and participants characteristics by pairwise comparisons

The following characteristics have been evaluated in all trials included in the network irrespectively of the outcome being reported.

### 9.2.1 Age


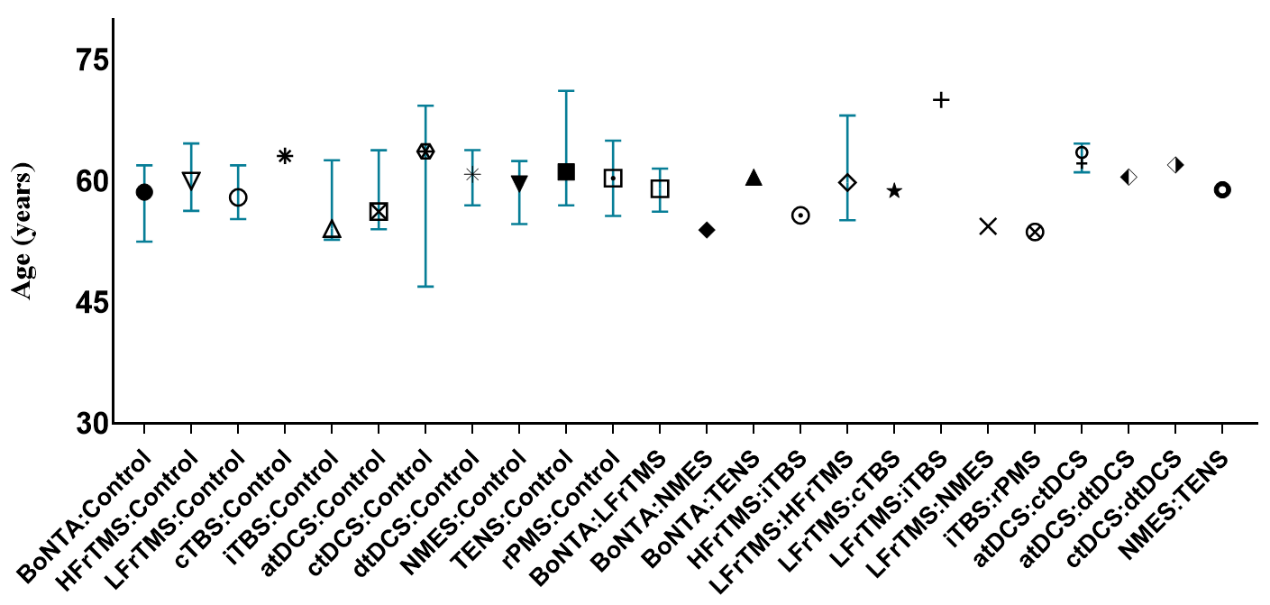
 The transitivity regarding the age of participants based on pairwise comparisons appears acceptable, as there is an overlap of characteristics across different comparisons, suggesting no significant differences among treatment comparisons (P=0.73).

Data are expressed as median (IQR). Kruskal-Wallis test was used for statistical analysis.

**Abbreviations:** BoNTA=botulinum toxin type A; LFrTMS=low-frequency transcranial magnetic stimulation; iTBS= intermittent theta-burst stimulation; HFrTMS = high-frequency transcranial magnetic stimulation; cTBS=continuous theta-burst stimulation; atDCS=anodal transcranial direct current stimulation; ctDCS=cathodal transcranial direct current stimulation; dtDCS=dual transcranial direct current stimulation; NMES=neuromuscular electrical stimulation; TENS=transcutaneous electrical nerve stimulation; rPMS= repetitive peripheral magnetic stimulation.

### 9.2.2 Percentage of male


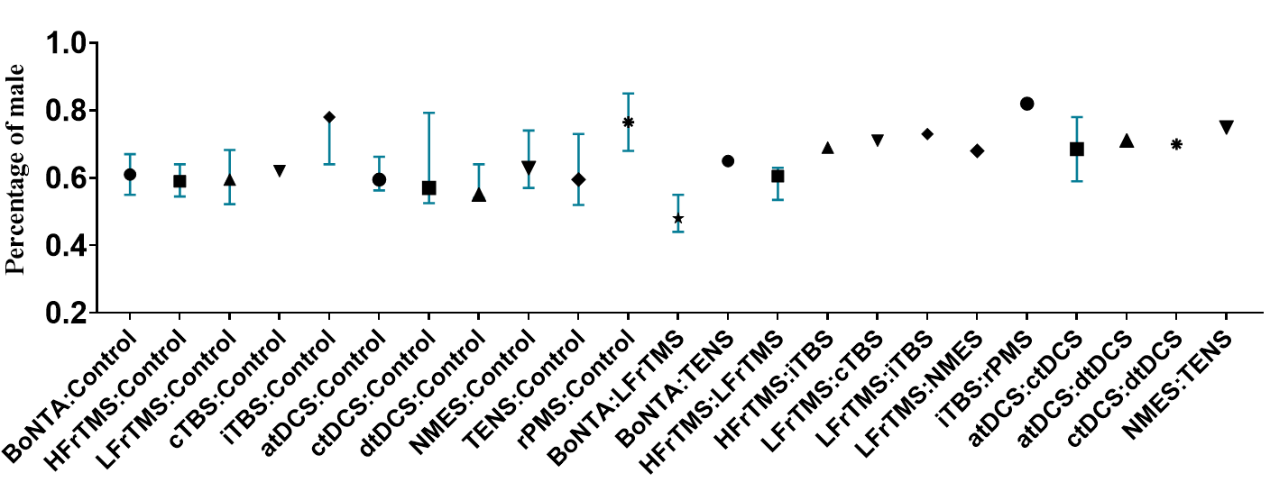
 The transitivity regarding the percentage of male based on pairwise comparisons appears acceptable, as there is an overlap of characteristics across different comparisons, suggesting no significant differences among treatment comparisons (P=0.064).

Data are expressed as median (IQR). Kruskal-Wallis test was used for statistical analysis.

**Abbreviations:** BoNTA=botulinum toxin type A; LFrTMS=low-frequency transcranial magnetic stimulation; iTBS= intermittent theta-burst stimulation; HFrTMS = high-frequency transcranial magnetic stimulation; cTBS=continuous theta-burst stimulation; atDCS=anodal transcranial direct current stimulation; ctDCS=cathodal transcranial direct current stimulation; dtDCS=dual transcranial direct current stimulation; NMES=neuromuscular electrical stimulation; TENS=transcutaneous electrical nerve stimulation; rPMS= repetitive peripheral magnetic stimulation.

### 9.2.3 Percentage of ischemic stroke


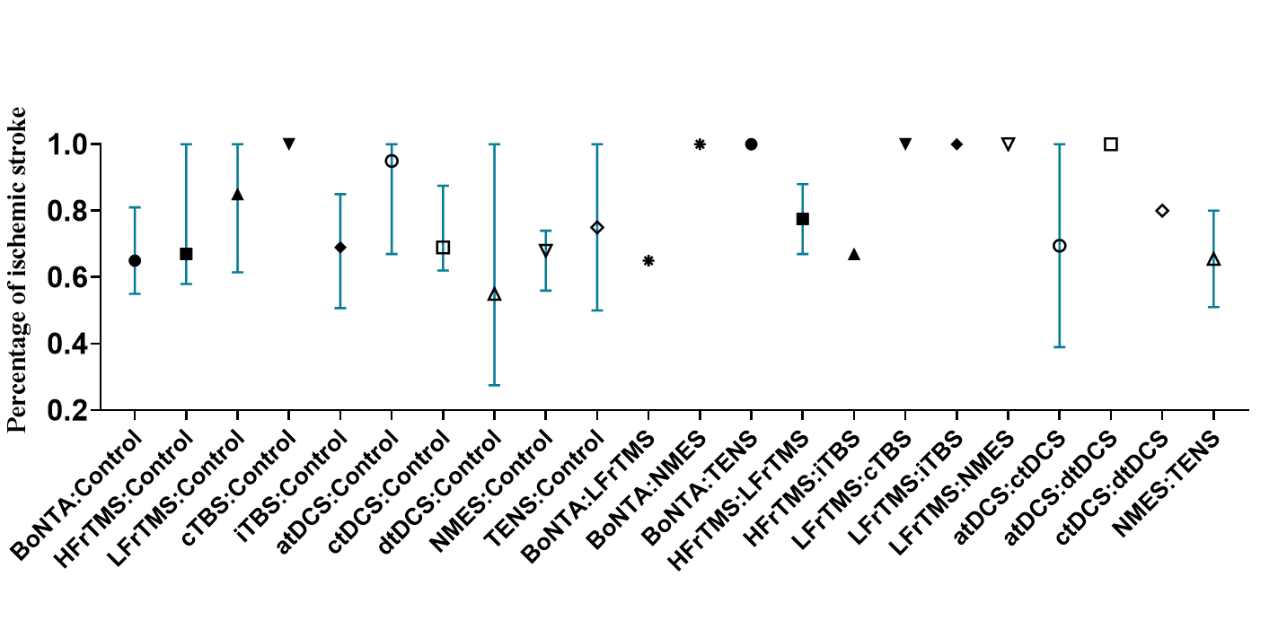
 The transitivity regarding the percentage of ischemic stroke based on pairwise comparisons appears acceptable, as there is an overlap of characteristics across different comparisons, suggesting no significant differences among treatment comparisons (P=0.28).

Data are expressed as median (IQR). Kruskal-Wallis test was used for statistical analysis.

**Abbreviations:** BoNTA=botulinum toxin type A; LFrTMS=low-frequency transcranial magnetic stimulation; iTBS= intermittent theta-burst stimulation; HFrTMS = high-frequency transcranial magnetic stimulation; cTBS=continuous theta-burst stimulation; atDCS=anodal transcranial direct current stimulation; ctDCS=cathodal transcranial direct current stimulation; dtDCS=dual transcranial direct current stimulation; NMES=neuromuscular electrical stimulation; TENS=transcutaneous electrical nerve stimulation; rPMS= repetitive peripheral magnetic stimulation.

### 9.2.4 Time since stroke


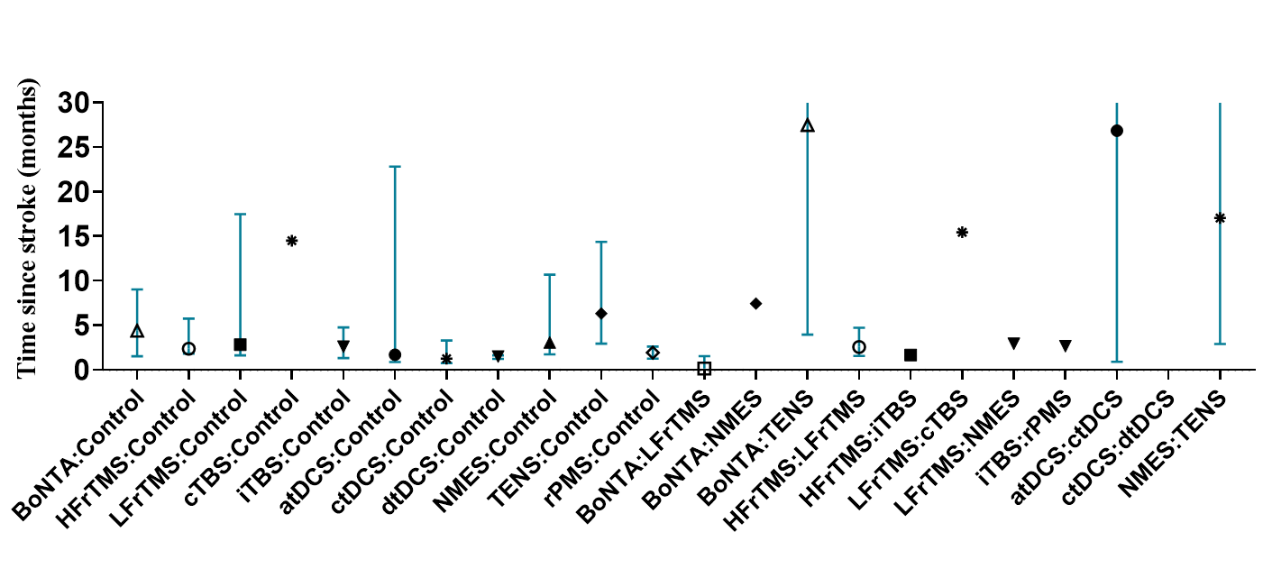
 The transitivity regarding the time since stroke based on pairwise comparisons appears acceptable, as there is an overlap of characteristics across different comparisons, suggesting no significant differences among treatment comparisons (P=0.15).

Data are expressed as median (IQR). Kruskal-Wallis test was used for statistical analysis.

**Abbreviations:** BoNTA=botulinum toxin type A; LFrTMS=low-frequency transcranial magnetic stimulation; iTBS= intermittent theta-burst stimulation; HFrTMS = high-frequency transcranial magnetic stimulation; cTBS=continuous theta-burst stimulation; atDCS=anodal transcranial direct current stimulation; ctDCS=cathodal transcranial direct current stimulation; dtDCS=dual transcranial direct current stimulation; NMES=neuromuscular electrical stimulation; TENS=transcutaneous electrical nerve stimulation; rPMS= repetitive peripheral magnetic stimulation.

### 9.2.5 Baseline MAS value


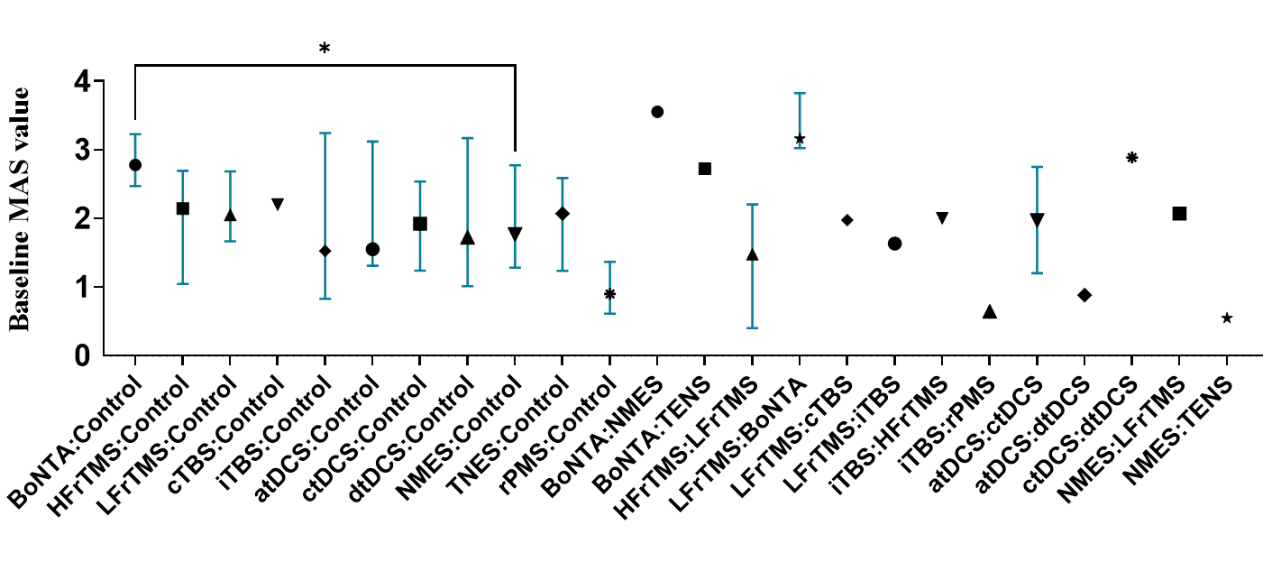
 Concerns about transitivity arise in the comparisons of BoNTA vs. Control with NMES vs. Control, due to variations in the baseline MAS value. However, these observations stem from analysis based on all trials included in the study, which does not influence pairwise and network meta-analyses.

Data are expressed as median (IQR). Kruskal-Wallis test was used for statistical analysis. * indicates P=0.016.

**Abbreviations:** BoNTA=botulinum toxin type A; LFrTMS=low-frequency transcranial magnetic stimulation; iTBS= intermittent theta-burst stimulation; HFrTMS = high-frequency transcranial magnetic stimulation; cTBS=continuous theta-burst stimulation; atDCS=anodal transcranial direct current stimulation; ctDCS=cathodal transcranial direct current stimulation; dtDCS=dual transcranial direct current stimulation; NMES=neuromuscular electrical stimulation; TENS=transcutaneous electrical nerve stimulation; rPMS= repetitive peripheral magnetic stimulation.

## 9.3 Studies and participants characteristics by network

### 9.3.1 Spasticity at short-term follow-up

**A.** Age


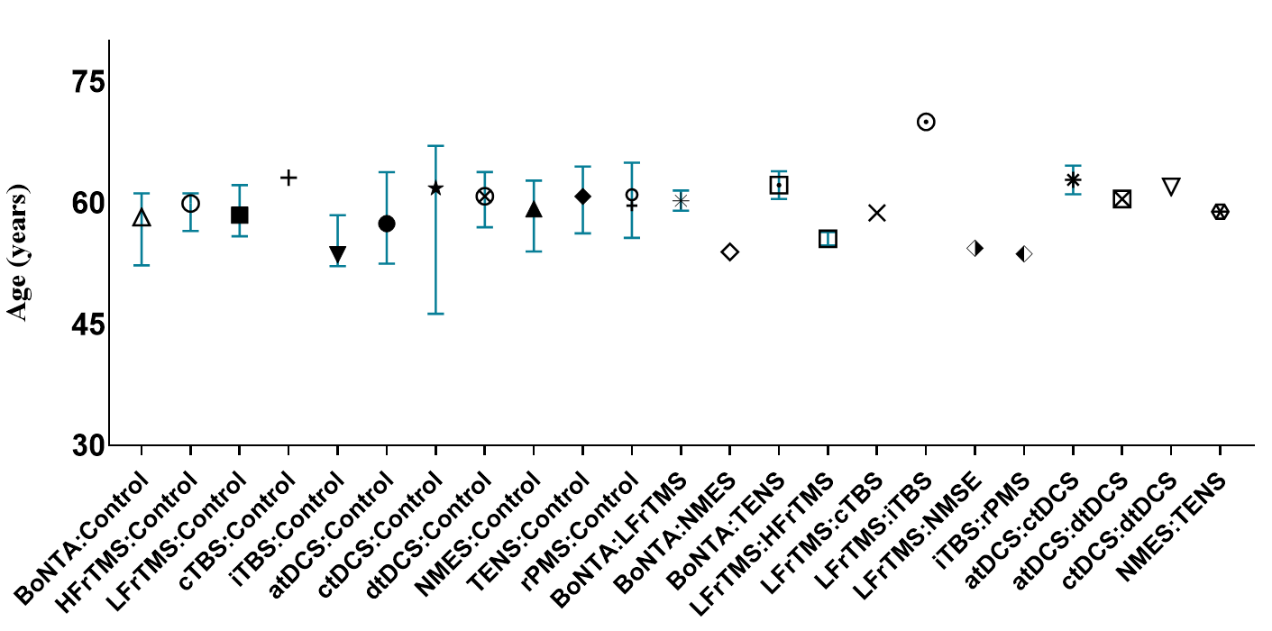
 The transitivity regarding the mean age based on network appears acceptable, as there is an overlap of characteristics across different comparisons, suggesting no significant differences among treatment comparisons (P=0.64).

Data are expressed as median (IQR). Kruskal-Wallis test was used for statistical analysis.

**Abbreviations:** BoNTA=botulinum toxin type A; LFrTMS=low-frequency transcranial magnetic stimulation; iTBS= intermittent theta-burst stimulation; HFrTMS = high-frequency transcranial magnetic stimulation; cTBS=continuous theta-burst stimulation; atDCS=anodal transcranial direct current stimulation; ctDCS=cathodal transcranial direct current stimulation; dtDCS=dual transcranial direct current stimulation; NMES=neuromuscular electrical stimulation; TENS=transcutaneous electrical nerve stimulation; rPMS= repetitive peripheral magnetic stimulation.

**B.** Percentage of male


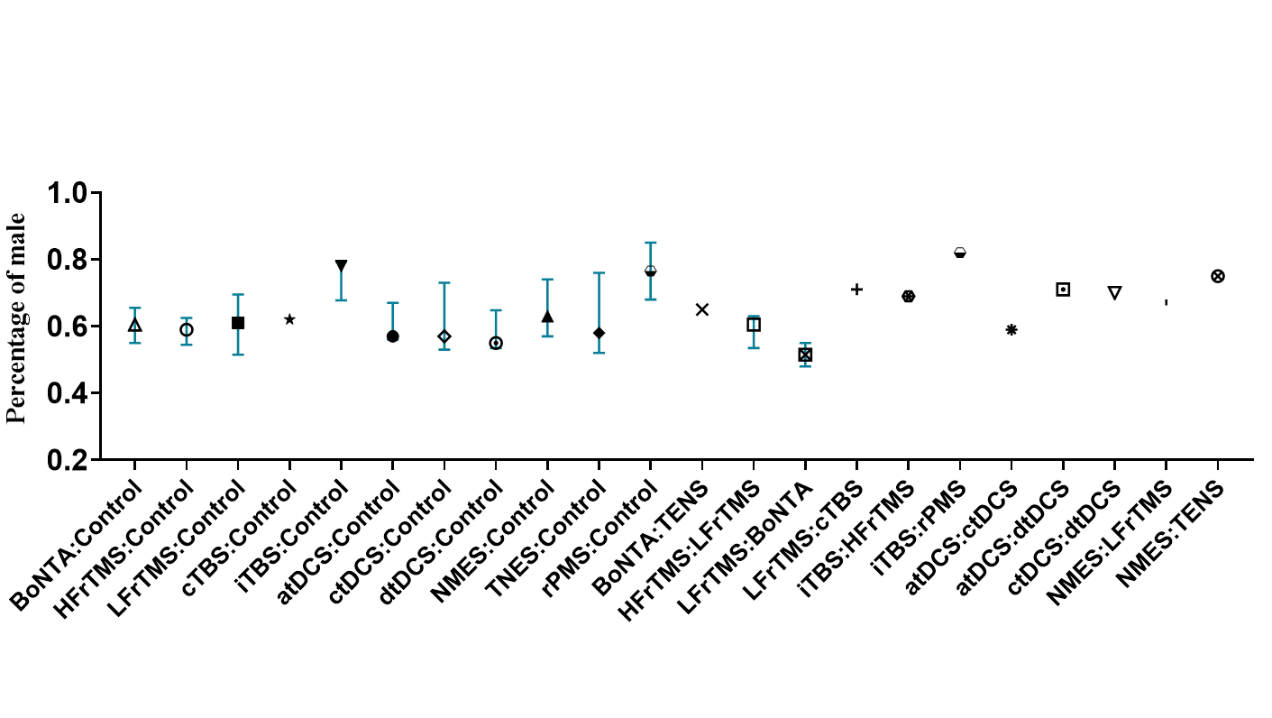
 The transitivity regarding the percentage of male based on network appears acceptable, as there is an overlap of characteristics across different comparisons, suggesting no significant differences among treatment comparisons (P=0.12).

Data are expressed as median (IQR). Kruskal-Wallis test was used for statistical analysis.

**Abbreviations:** BoNTA=botulinum toxin type A; LFrTMS=low-frequency transcranial magnetic stimulation; iTBS= intermittent theta-burst stimulation; HFrTMS = high-frequency transcranial magnetic stimulation; cTBS=continuous theta-burst stimulation; atDCS=anodal transcranial direct current stimulation; ctDCS=cathodal transcranial direct current stimulation; dtDCS=dual transcranial direct current stimulation; NMES=neuromuscular electrical stimulation; TENS=transcutaneous electrical nerve stimulation; rPMS= repetitive peripheral magnetic stimulation.

**C.** Percentage of ischemic stroke


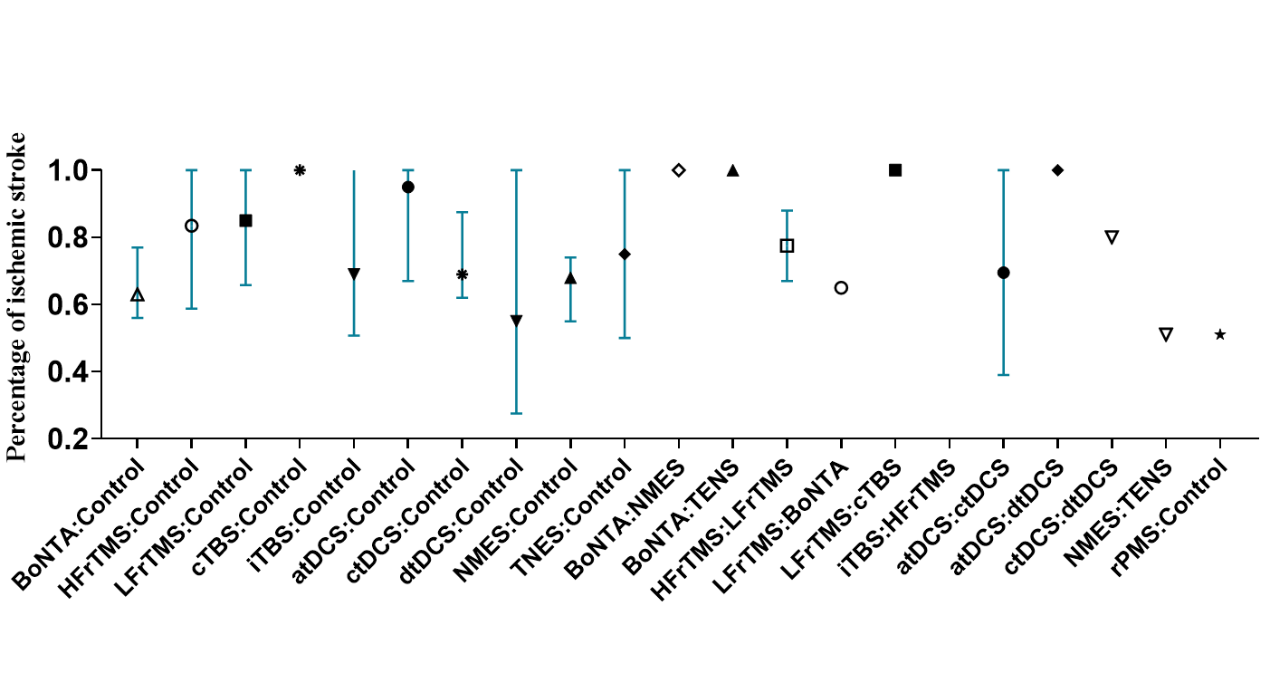
 The transitivity regarding the percentage of ischemic stroke based on network appears acceptable, as there is an overlap of characteristics across different comparisons, suggesting no significant differences among treatment comparisons (P=0.35).

Data are expressed as median (IQR). Kruskal-Wallis test was used for statistical analysis.

**Abbreviations:** BoNTA=botulinum toxin type A; LFrTMS=low-frequency transcranial magnetic stimulation; iTBS= intermittent theta-burst stimulation; HFrTMS = high-frequency transcranial magnetic stimulation; cTBS=continuous theta-burst stimulation; atDCS=anodal transcranial direct current stimulation; ctDCS=cathodal transcranial direct current stimulation; dtDCS=dual transcranial direct current stimulation; NMES=neuromuscular electrical stimulation; TENS=transcutaneous electrical nerve stimulation; rPMS= repetitive peripheral magnetic stimulation.

**D.** Time since stroke


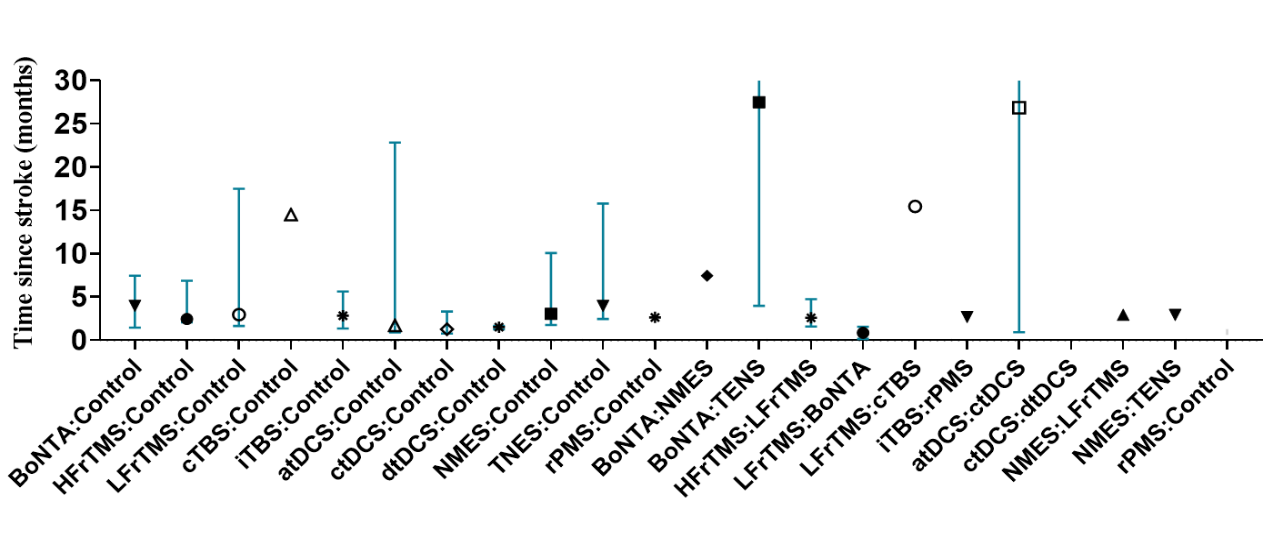
 The transitivity regarding the time since stroke based on network appears acceptable, as there is an overlap of characteristics across different comparisons, suggesting no significant differences among treatment comparisons (P=0.36).

Data are expressed as median (IQR). Kruskal-Wallis test was used for statistical analysis.

**Abbreviations:** BoNTA=botulinum toxin type A; LFrTMS=low-frequency transcranial magnetic stimulation; iTBS= intermittent theta-burst stimulation; HFrTMS = high-frequency transcranial magnetic stimulation; cTBS=continuous theta-burst stimulation; atDCS=anodal transcranial direct current stimulation; ctDCS=cathodal transcranial direct current stimulation; dtDCS=dual transcranial direct current stimulation; NMES=neuromuscular electrical stimulation; TENS=transcutaneous electrical nerve stimulation; rPMS= repetitive peripheral magnetic stimulation.

**E.** Baseline mean MAS value


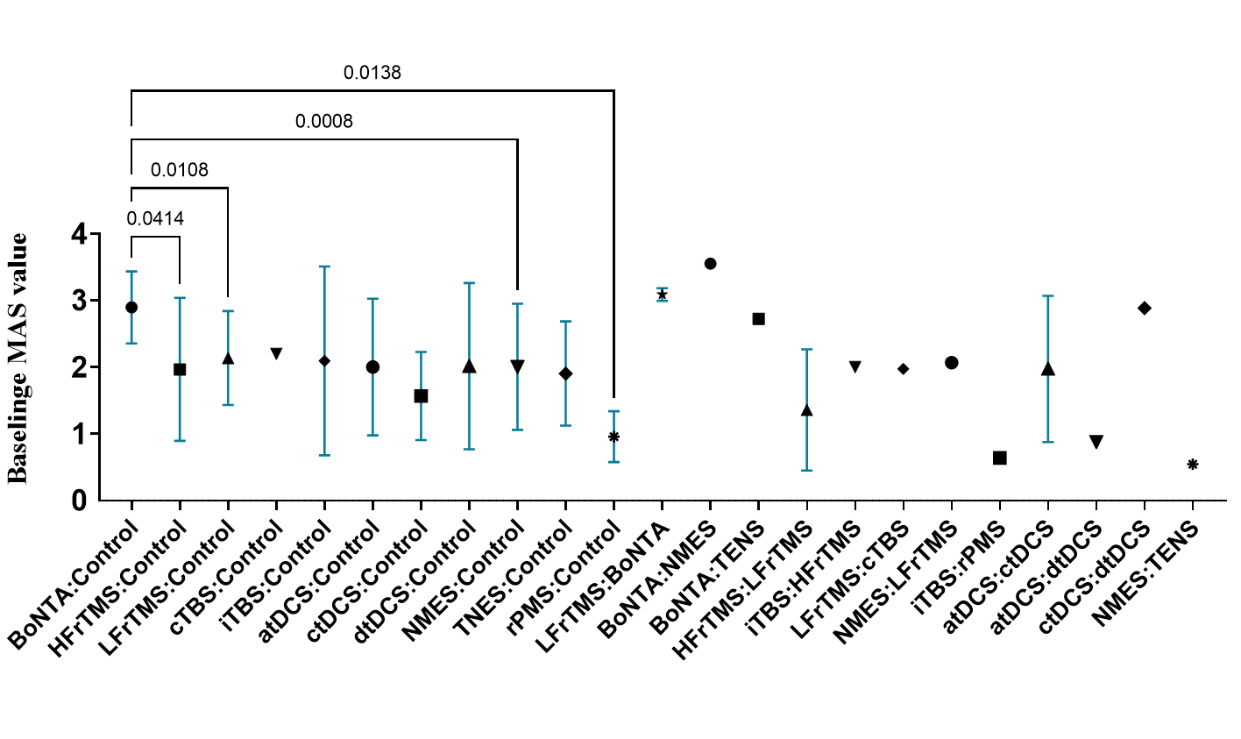
 Concerns about transitivity arise in the comparisons of BoNTA:Control with HFrTMS:Control, BoNTA:Control with LFrTMS:Control, BoNTA:Control with NMES:Control, and BoNTA:Control with rPMS:Control, due to variations in the baseline MAS value. Therefore, we conducted sensitivity by excluding trials with BoNTA:Control to test the effect of baseline MAS value.

Data are expressed as mean (SD). One-way ANOVA was used for statistical analysis. BoNTA:Control vs HFrTMS:Control (P=0.041), BoNTA:Control vs LFrTMS:Control (P=0.011), BoNTA:Control vs NMES:Control (P=0.008), BoNTA:Control vs rPMS:Control (P=0.014).

**Abbreviations:** BoNTA=botulinum toxin type A; LFrTMS=low-frequency transcranial magnetic stimulation; iTBS= intermittent theta-burst stimulation; HFrTMS = high-frequency transcranial magnetic stimulation; cTBS=continuous theta-burst stimulation; atDCS=anodal transcranial direct current stimulation; ctDCS=cathodal transcranial direct current stimulation; dtDCS=dual transcranial direct current stimulation; NMES=neuromuscular electrical stimulation; TENS=transcutaneous electrical nerve stimulation; rPMS= repetitive peripheral magnetic stimulation.

### 9.3.2 Spasticity at mid-term follow-up

**A.** Age


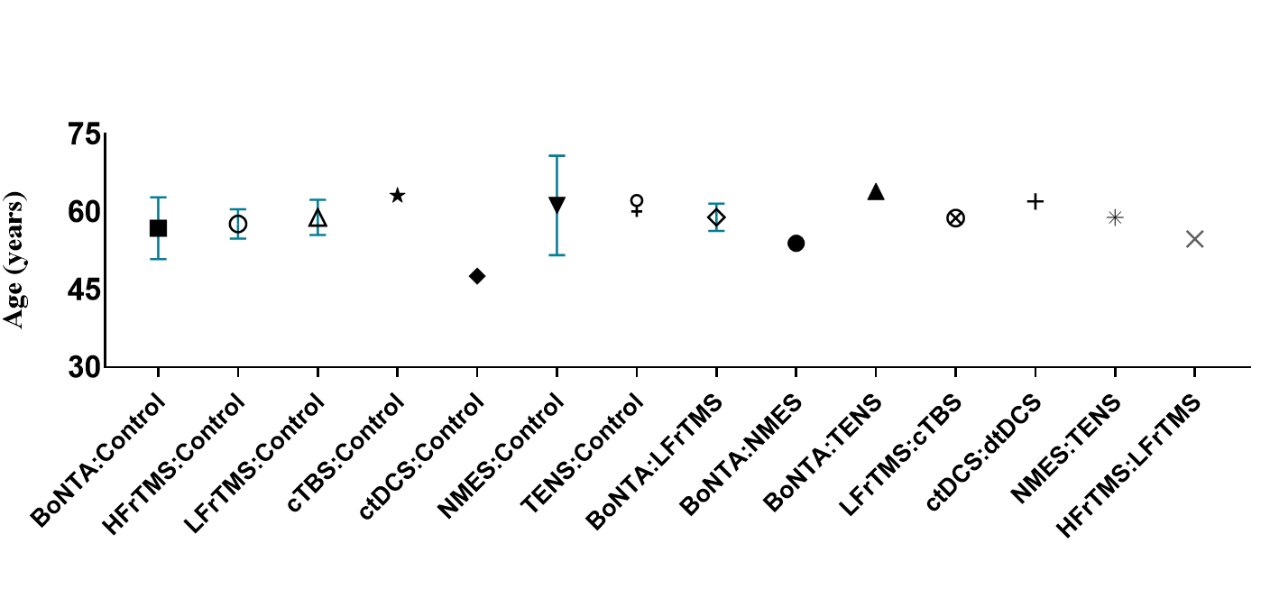
 The transitivity regarding the mean age based on network appears acceptable, as there is an overlap of characteristics across different comparisons, suggesting no significant differences among treatment comparisons (P=0.54).

Data are expressed as median (IQR). Kruskal-Wallis test was used for statistical analysis.

**Abbreviations:** BoNTA=botulinum toxin type A; LFrTMS=low-frequency transcranial magnetic stimulation; iTBS= intermittent theta-burst stimulation; HFrTMS = high-frequency transcranial magnetic stimulation; cTBS=continuous theta-burst stimulation; atDCS=anodal transcranial direct current stimulation; ctDCS=cathodal transcranial direct current stimulation; dtDCS=dual transcranial direct current stimulation; NMES=neuromuscular electrical stimulation; TENS=transcutaneous electrical nerve stimulation; rPMS= repetitive peripheral magnetic stimulation.

**B.** Percentage of male


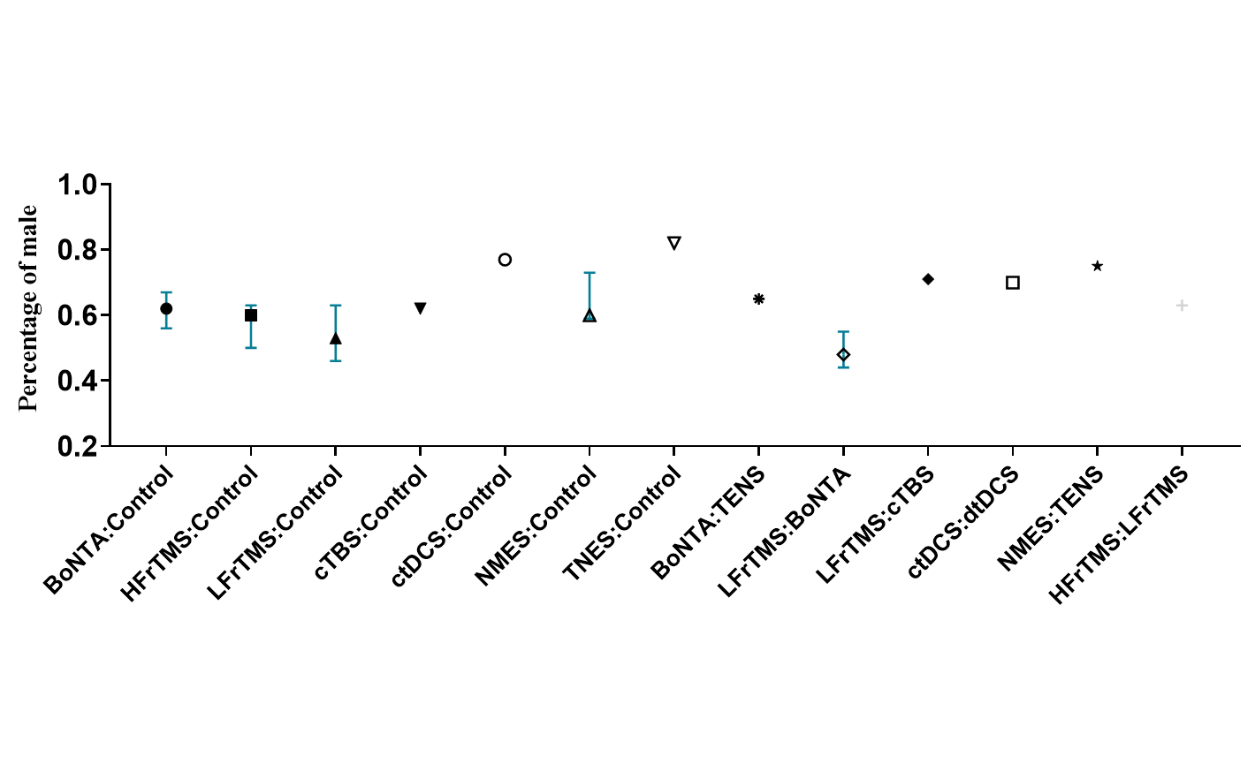
 The transitivity regarding the percentage of male based on network appears acceptable, as there is an overlap of characteristics across different comparisons, suggesting no significant differences among treatment comparisons (P=0.13).

Data are expressed as median (IQR). Kruskal-Wallis test was used for statistical analysis.

**Abbreviations:** BoNTA=botulinum toxin type A; LFrTMS=low-frequency transcranial magnetic stimulation; iTBS= intermittent theta-burst stimulation; HFrTMS = high-frequency transcranial magnetic stimulation; cTBS=continuous theta-burst stimulation; atDCS=anodal transcranial direct current stimulation; ctDCS=cathodal transcranial direct current stimulation; dtDCS=dual transcranial direct current stimulation; NMES=neuromuscular electrical stimulation; TENS=transcutaneous electrical nerve stimulation; rPMS= repetitive peripheral magnetic stimulation.

**C.** Percentage of ischemic stroke


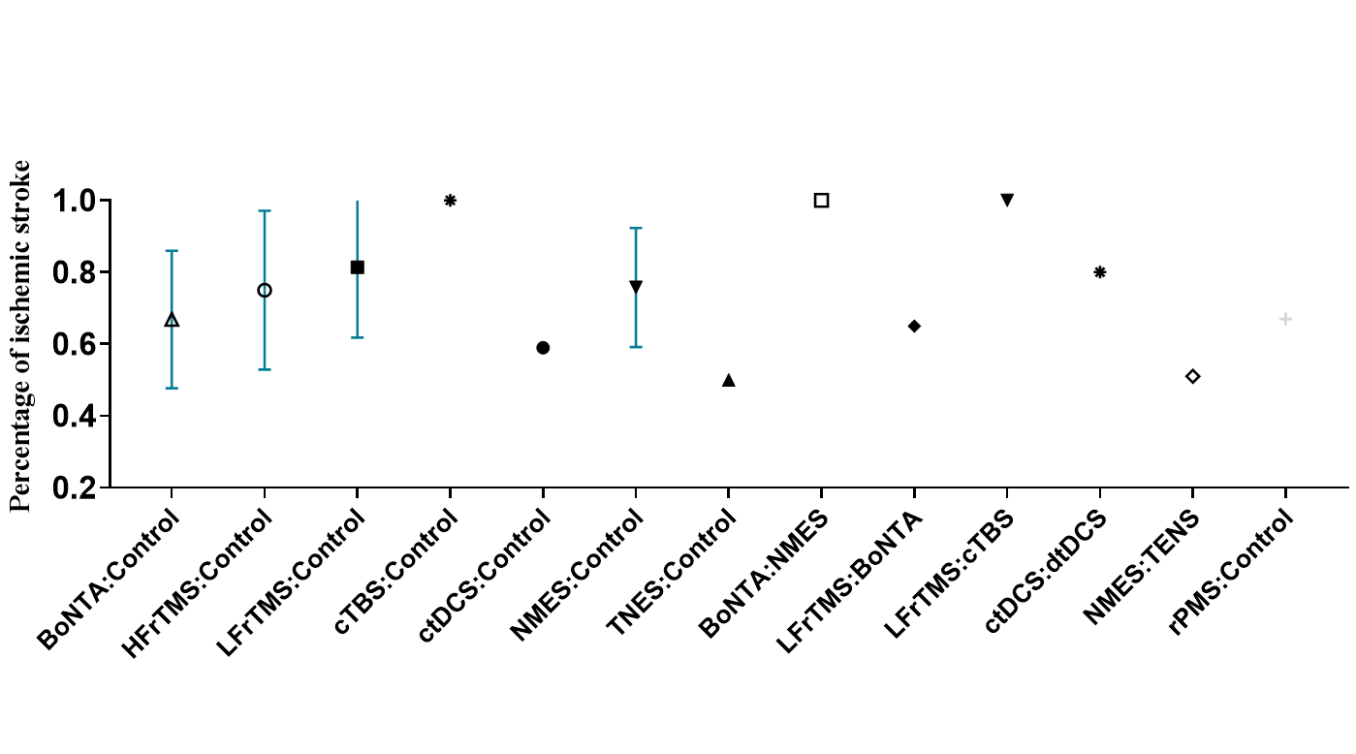
 The transitivity regarding the percentage of ischemic stroke based on network appears acceptable, as there is an overlap of characteristics across different comparisons, suggesting no significant differences among treatment comparisons (P=0.40).

Data are expressed as mean (SD). One-way ANOVA was used for statistical analysis.

**Abbreviations:** BoNTA=botulinum toxin type A; LFrTMS=low-frequency transcranial magnetic stimulation; iTBS= intermittent theta-burst stimulation; HFrTMS = high-frequency transcranial magnetic stimulation; cTBS=continuous theta-burst stimulation; atDCS=anodal transcranial direct current stimulation; ctDCS=cathodal transcranial direct current stimulation; dtDCS=dual transcranial direct current stimulation; NMES=neuromuscular electrical stimulation; TENS=transcutaneous electrical nerve stimulation; rPMS= repetitive peripheral magnetic stimulation.

**D.** Time since stroke


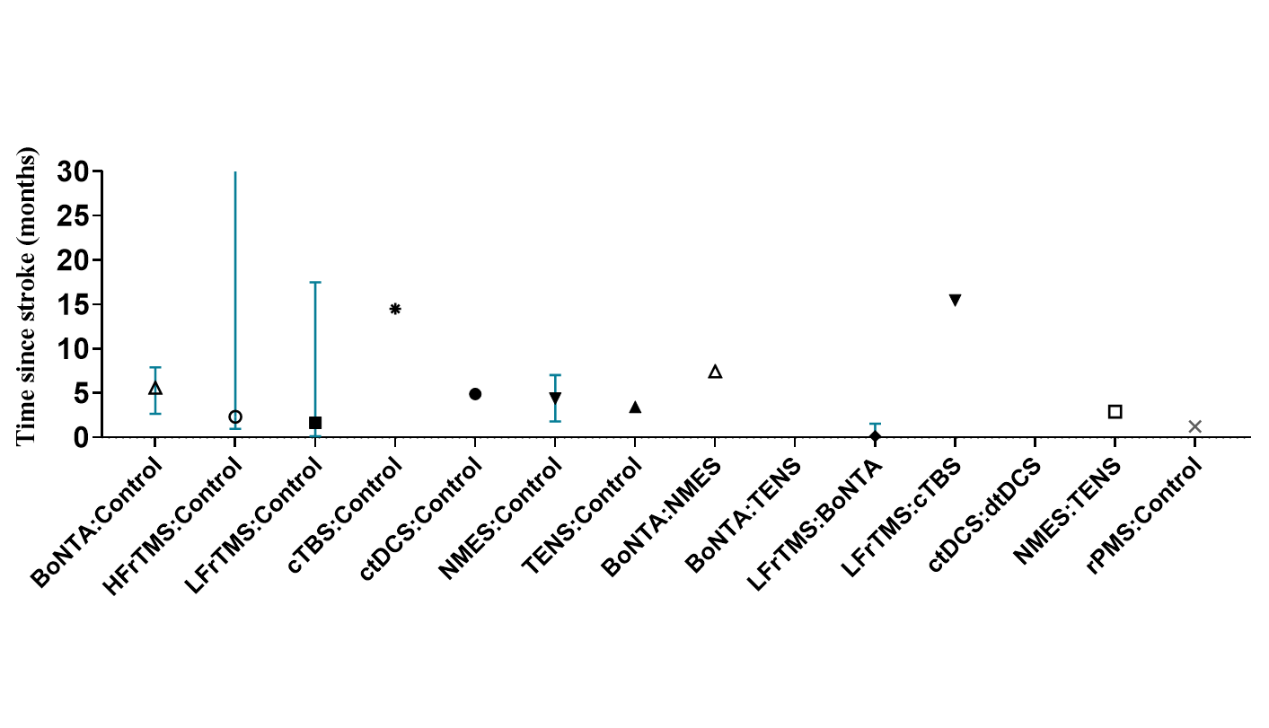
 The transitivity regarding time since stroke based on network appears acceptable, as there is an overlap of characteristics across different comparisons, suggesting no significant differences among treatment comparisons (P=0.43).

Data are expressed as median (IQR). Kruskal-Wallis test was used for statistical analysis.

**Abbreviations:** BoNTA=botulinum toxin type A; LFrTMS=low-frequency transcranial magnetic stimulation; iTBS= intermittent theta-burst stimulation; HFrTMS = high-frequency transcranial magnetic stimulation; cTBS=continuous theta-burst stimulation; atDCS=anodal transcranial direct current stimulation; ctDCS=cathodal transcranial direct current stimulation; dtDCS=dual transcranial direct current stimulation; NMES=neuromuscular electrical stimulation; TENS=transcutaneous electrical nerve stimulation; rPMS= repetitive peripheral magnetic stimulation.

**E.** Baseline mean MAS value


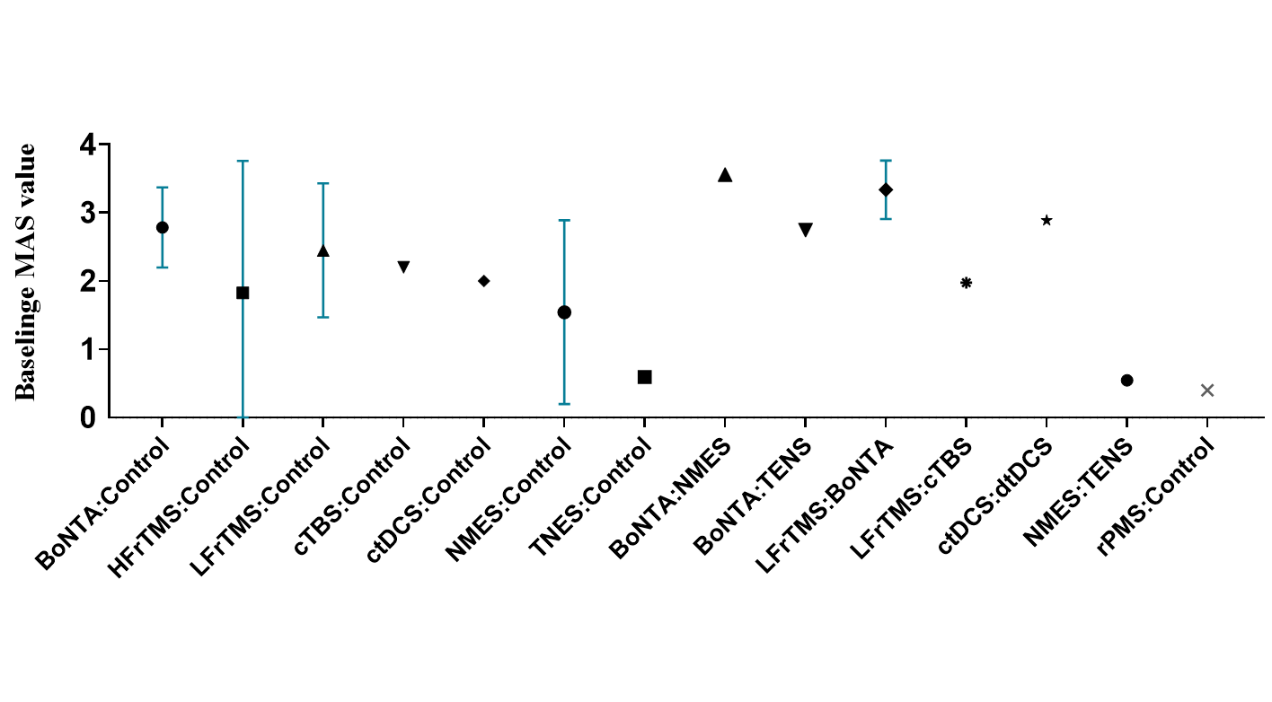


The transitivity regarding baseline MAS value based on network appears acceptable, as there is an overlap of characteristics across different comparisons, suggesting no significant differences among treatment comparisons (all P>0.05).

Data are expressed as mean (SD). One-way ANOVA test was used for statistical analysis.

**Abbreviations:** BoNTA=botulinum toxin type A; LFrTMS=low-frequency transcranial magnetic stimulation; iTBS= intermittent theta-burst stimulation; HFrTMS = high-frequency transcranial magnetic stimulation; cTBS=continuous theta-burst stimulation; atDCS=anodal transcranial direct current stimulation; ctDCS=cathodal transcranial direct current stimulation; dtDCS=dual transcranial direct current stimulation; NMES=neuromuscular electrical stimulation; TENS=transcutaneous electrical nerve stimulation; rPMS= repetitive peripheral magnetic stimulation.

### 9.3.3 Motor function at short-term follow-up

**A.** Age


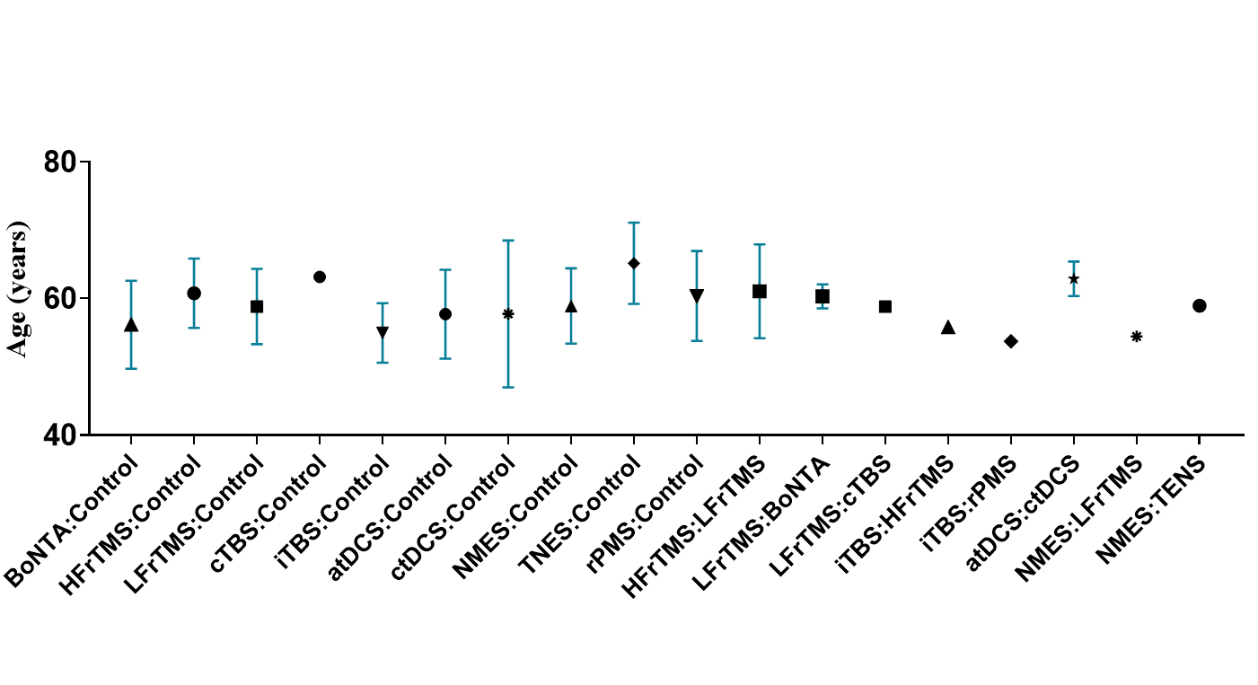


The transitivity regarding the age based on network appears acceptable, as there is an overlap of characteristics across different comparisons, suggesting no significant differences among treatment comparisons (P=0.93).

Data are expressed as mean (SD). One-way ANOVA test was used for statistical analysis.

**Abbreviations:** BoNTA=botulinum toxin type A; LFrTMS=low-frequency transcranial magnetic stimulation; iTBS= intermittent theta-burst stimulation; HFrTMS = high-frequency transcranial magnetic stimulation; cTBS=continuous theta-burst stimulation; atDCS=anodal transcranial direct current stimulation; ctDCS=cathodal transcranial direct current stimulation; dtDCS=dual transcranial direct current stimulation; NMES=neuromuscular electrical stimulation; TENS=transcutaneous electrical nerve stimulation; rPMS= repetitive peripheral magnetic stimulation.

**B.** Percentage of male


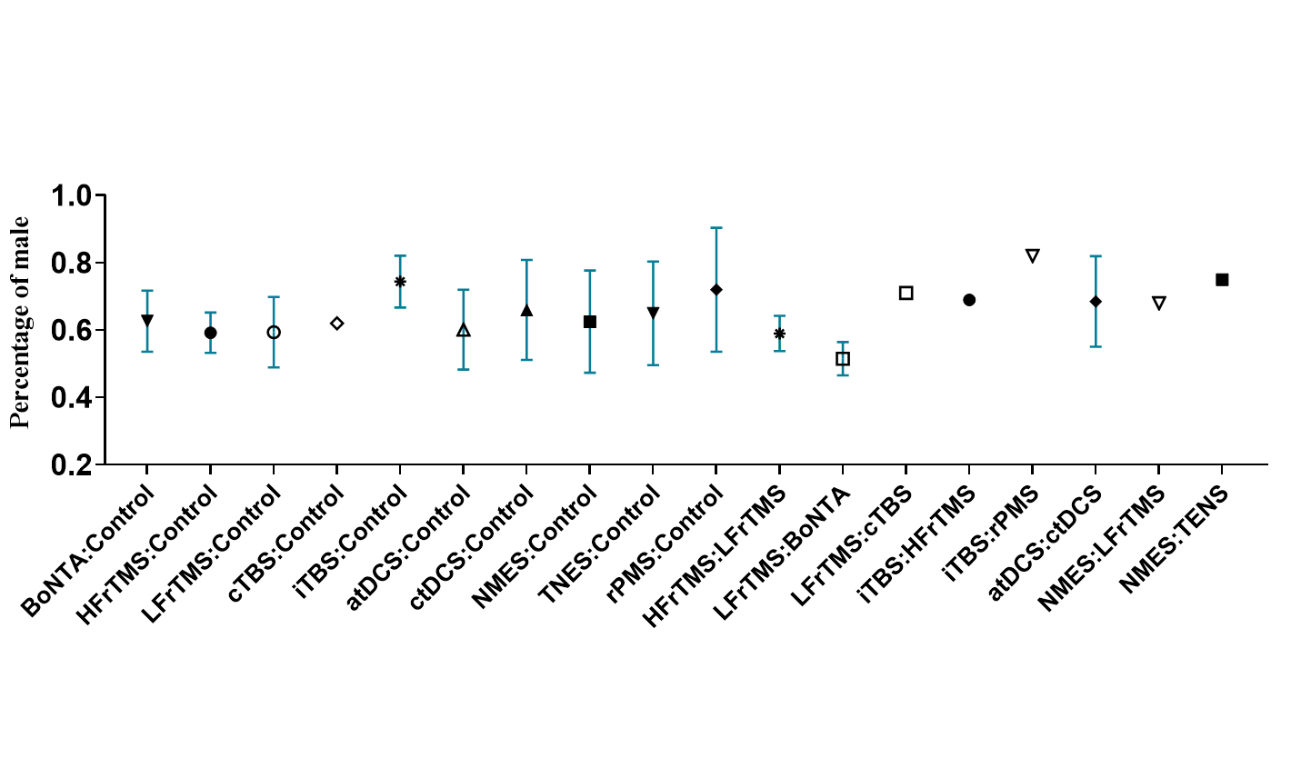
 The transitivity regarding the percentage of male based on network appears acceptable, as there is an overlap of characteristics across different comparisons, suggesting no significant differences among treatment comparisons (P=0.26).

Data are expressed as mean (SD). One-way ANOVA test was used for statistical analysis.

**Abbreviations:** BoNTA=botulinum toxin type A; LFrTMS=low-frequency transcranial magnetic stimulation; iTBS= intermittent theta-burst stimulation; HFrTMS = high-frequency transcranial magnetic stimulation; cTBS=continuous theta-burst stimulation; atDCS=anodal transcranial direct current stimulation; ctDCS=cathodal transcranial direct current stimulation; dtDCS=dual transcranial direct current stimulation; NMES=neuromuscular electrical stimulation; TENS=transcutaneous electrical nerve stimulation; rPMS= repetitive peripheral magnetic stimulation.

**C.** Percentage of ischemic stroke


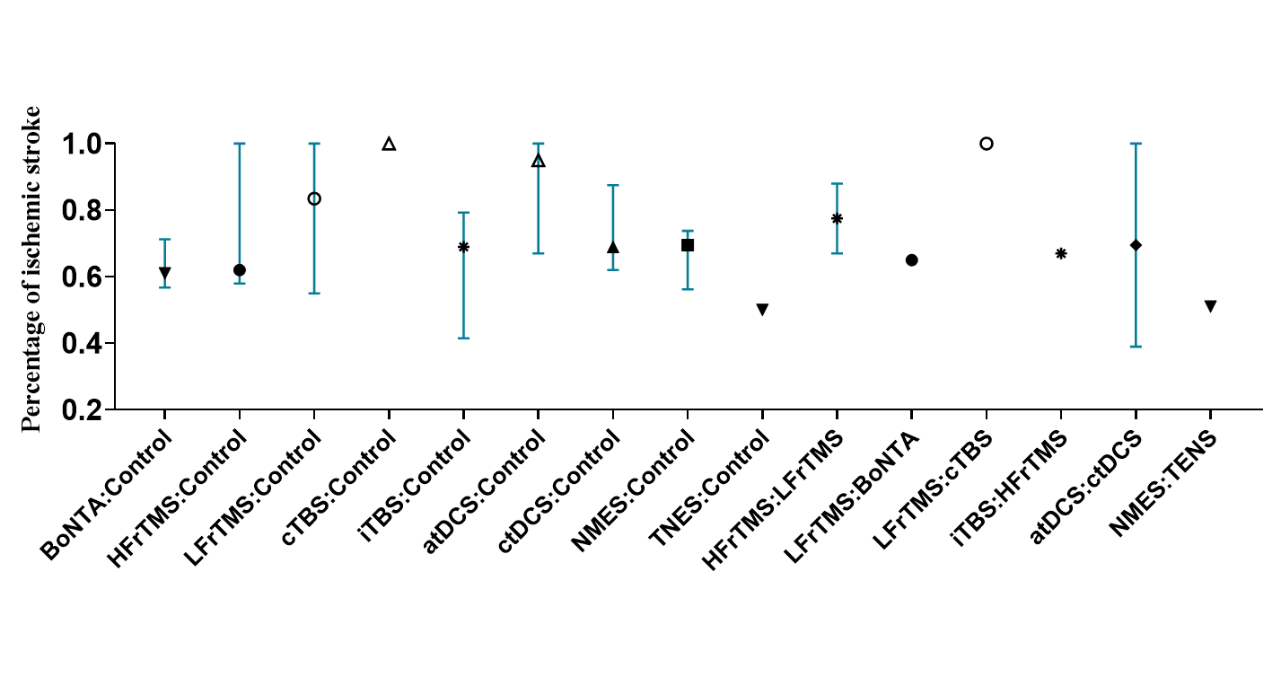
 The transitivity regarding the percentage of ischemic stroke based on network appears acceptable, as there is an overlap of characteristics across different comparisons, suggesting no significant differences among treatment comparisons (P=0.37).

Data are expressed as median (IQR). Kruskal-Wallis test was used for statistical analysis.

**Abbreviations:** BoNTA=botulinum toxin type A; LFrTMS=low-frequency transcranial magnetic stimulation; iTBS= intermittent theta-burst stimulation; HFrTMS = high-frequency transcranial magnetic stimulation; cTBS=continuous theta-burst stimulation; atDCS=anodal transcranial direct current stimulation; ctDCS=cathodal transcranial direct current stimulation; dtDCS=dual transcranial direct current stimulation; NMES=neuromuscular electrical stimulation; TENS=transcutaneous electrical nerve stimulation; rPMS= repetitive peripheral magnetic stimulation.

**D.** Time since stroke


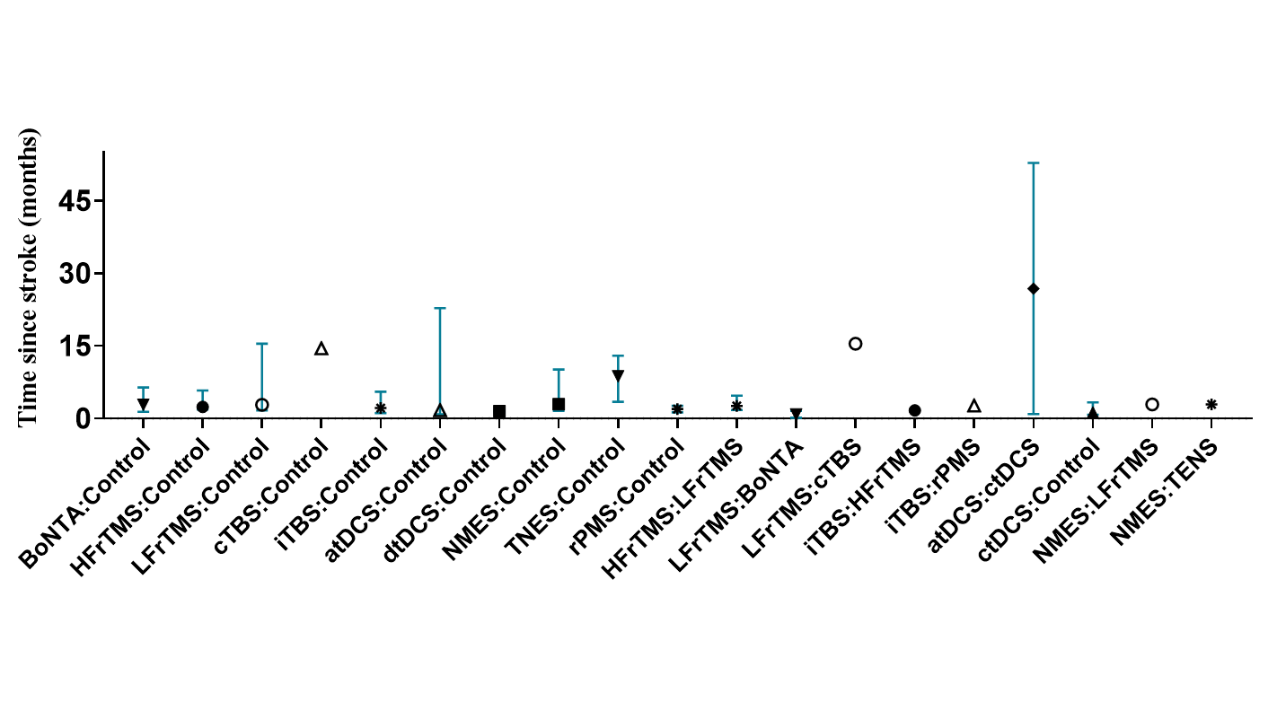
 The transitivity regarding time since stroke based on network appears acceptable, as there is an overlap of characteristics across different comparisons, suggesting no significant differences among treatment comparisons (P=0.37).

Data are expressed as median (IQR). Kruskal-Wallis test was used for statistical analysis.

**Abbreviations:** BoNTA=botulinum toxin type A; LFrTMS=low-frequency transcranial magnetic stimulation; iTBS= intermittent theta-burst stimulation; HFrTMS = high-frequency transcranial magnetic stimulation; cTBS=continuous theta-burst stimulation; atDCS=anodal transcranial direct current stimulation; ctDCS=cathodal transcranial direct current stimulation; dtDCS=dual transcranial direct current stimulation; NMES=neuromuscular electrical stimulation; TENS=transcutaneous electrical nerve stimulation; rPMS= repetitive peripheral magnetic stimulation.

### 9.3.4 Motor function at mid-term follow-up

**A.** Age


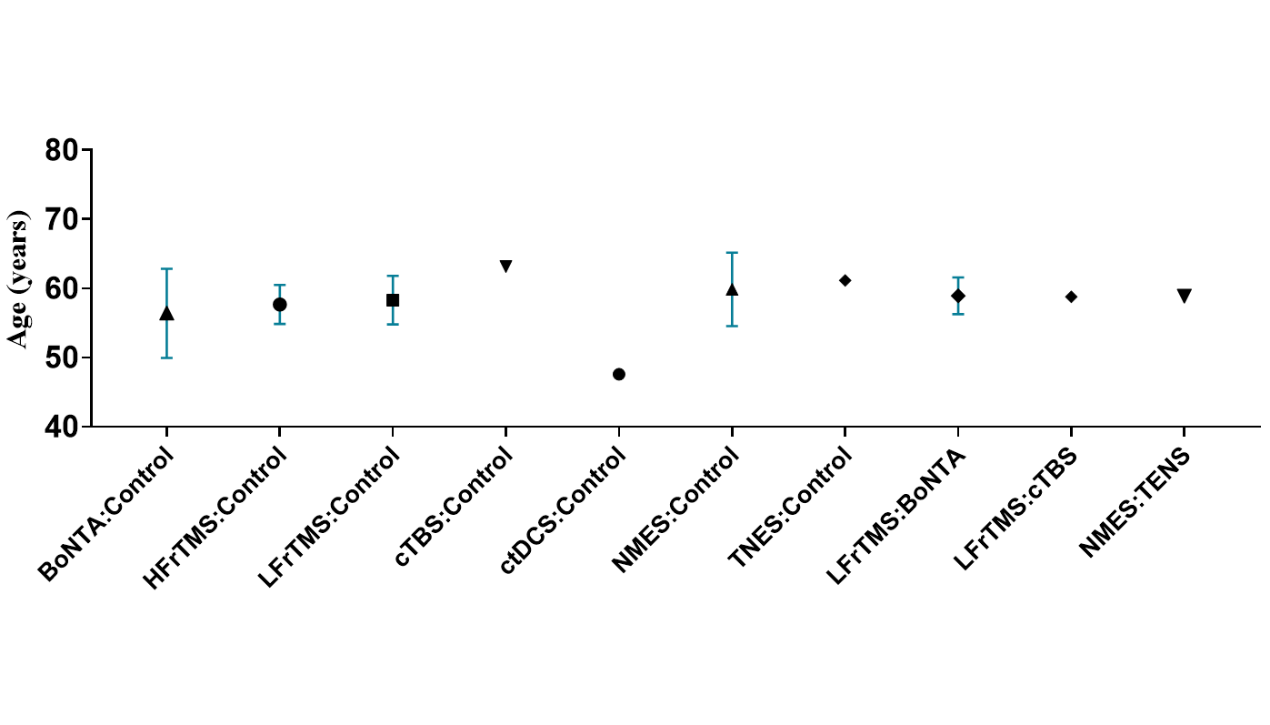


The transitivity regarding age based on network appears acceptable, as there is an overlap of characteristics across different comparisons, suggesting no significant differences among treatment comparisons (P=0.85).

Data are expressed as mean (SD). One-way ANOVA test was used for statistical analysis.

**Abbreviations:** BoNTA=botulinum toxin type A; LFrTMS=low-frequency transcranial magnetic stimulation; iTBS= intermittent theta-burst stimulation; HFrTMS = high-frequency transcranial magnetic stimulation; cTBS=continuous theta-burst stimulation; atDCS=anodal transcranial direct current stimulation; ctDCS=cathodal transcranial direct current stimulation; dtDCS=dual transcranial direct current stimulation; NMES=neuromuscular electrical stimulation; TENS=transcutaneous electrical nerve stimulation; rPMS= repetitive peripheral magnetic stimulation.

**B.** Percentage of male


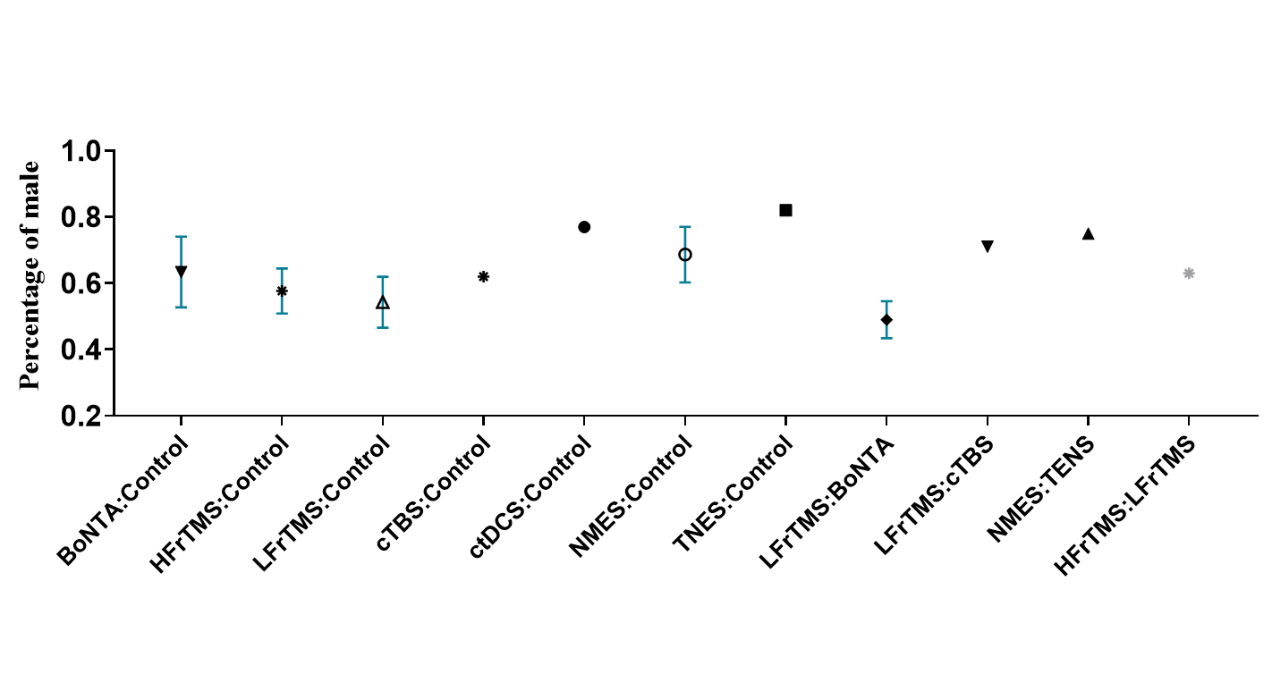
 The transitivity regarding the percentage of male based on network appears acceptable, as there is an overlap of characteristics across different comparisons, suggesting no significant differences among treatment comparisons (all P>0.05).

Data are expressed as mean (SD). One-way ANOVA test was used for statistical analysis.

**Abbreviations:** BoNTA=botulinum toxin type A; LFrTMS=low-frequency transcranial magnetic stimulation; iTBS= intermittent theta-burst stimulation; HFrTMS = high-frequency transcranial magnetic stimulation; cTBS=continuous theta-burst stimulation; atDCS=anodal transcranial direct current stimulation; ctDCS=cathodal transcranial direct current stimulation; dtDCS=dual transcranial direct current stimulation; NMES=neuromuscular electrical stimulation; TENS=transcutaneous electrical nerve stimulation; rPMS= repetitive peripheral magnetic stimulation.

**C.** Percentage of ischemic stroke


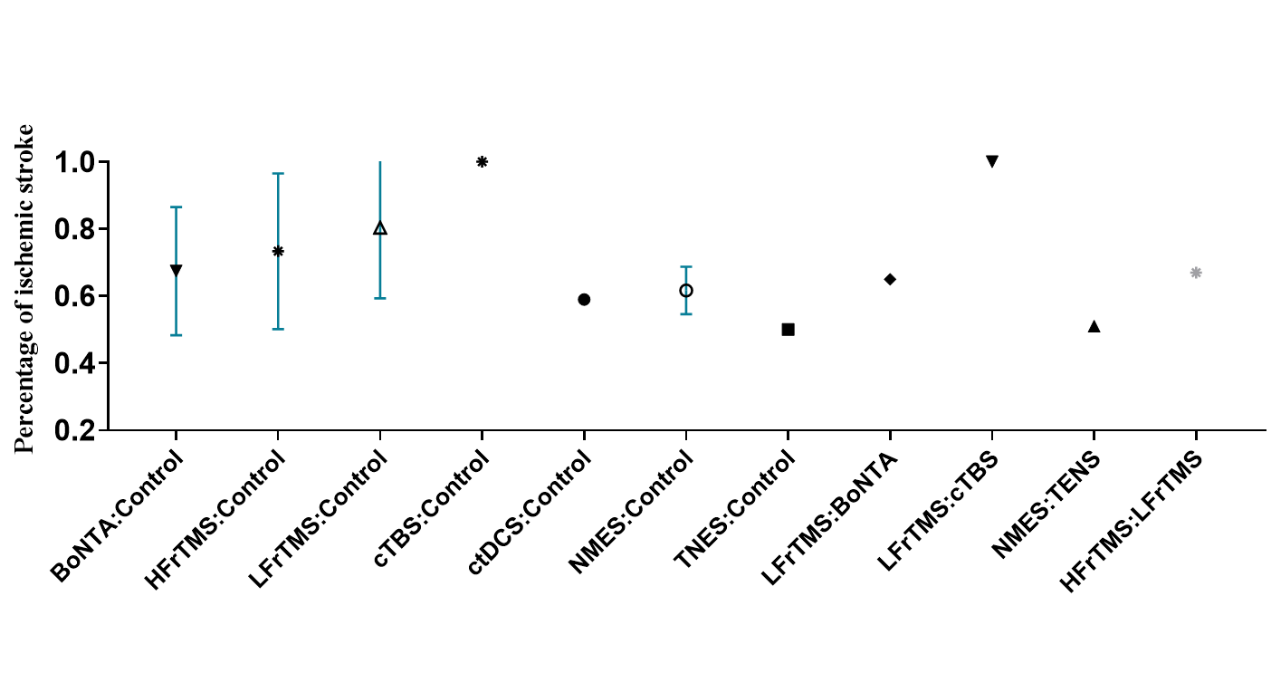
 The transitivity regarding the percentage of ischemic stroke based on network appears acceptable, as there is an overlap of characteristics across different comparisons, suggesting no significant differences among treatment comparisons (P=0.99).

Data are expressed as mean (SD). One-way ANOVA test was used for statistical analysis.

**Abbreviations:** BoNTA=botulinum toxin type A; LFrTMS=low-frequency transcranial magnetic stimulation; iTBS= intermittent theta-burst stimulation; HFrTMS = high-frequency transcranial magnetic stimulation; cTBS=continuous theta-burst stimulation; atDCS=anodal transcranial direct current stimulation; ctDCS=cathodal transcranial direct current stimulation; dtDCS=dual transcranial direct current stimulation; NMES=neuromuscular electrical stimulation; TENS=transcutaneous electrical nerve stimulation; rPMS= repetitive peripheral magnetic stimulation.

**D.** Time since stroke


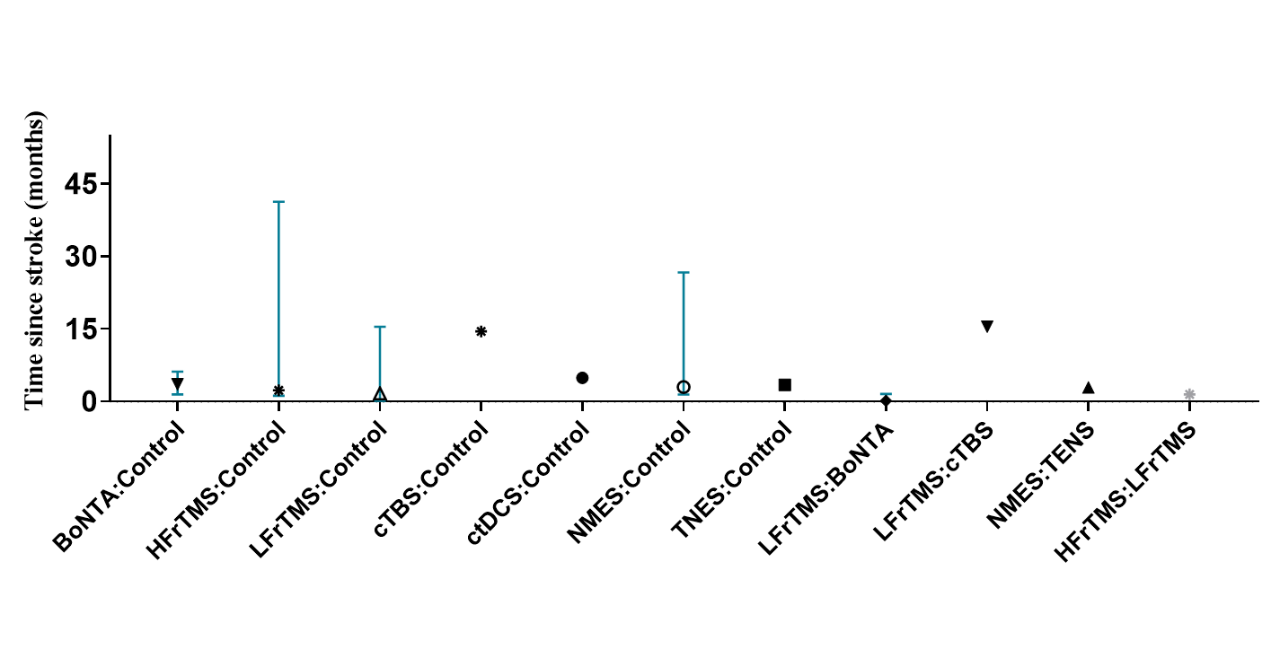
 The transitivity regarding time since stroke based on network appears acceptable, as there is an overlap of characteristics across different comparisons, suggesting no significant differences among treatment comparisons (P=0.60).

Data are expressed as median (IQR). Kruskal-Wallis test was used for statistical analysis.

**Abbreviations:** BoNTA=botulinum toxin type A; LFrTMS=low-frequency transcranial magnetic stimulation; iTBS= intermittent theta-burst stimulation; HFrTMS = high-frequency transcranial magnetic stimulation; cTBS=continuous theta-burst stimulation; atDCS=anodal transcranial direct current stimulation; ctDCS=cathodal transcranial direct current stimulation; dtDCS=dual transcranial direct current stimulation; NMES=neuromuscular electrical stimulation; TENS=transcutaneous electrical nerve stimulation; rPMS= repetitive peripheral magnetic stimulation.

### 9.3.5 Acceptability

**A.** Mean age


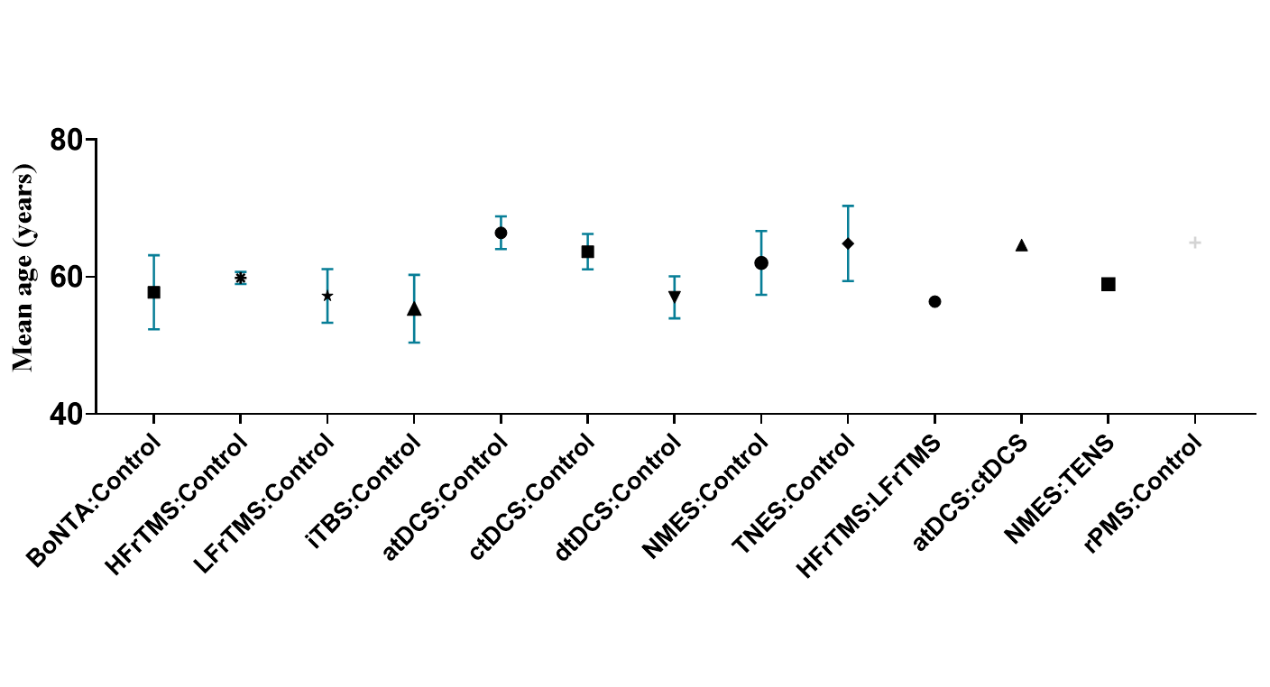
 The transitivity regarding mean age based on network appears acceptable, as there is an overlap of characteristics across different comparisons, suggesting no significant differences among treatment comparisons (P=0.062).

Data are expressed as mean (SD). One-way ANOVA was used for statistical analysis.

**Abbreviations:** BoNTA=botulinum toxin type A; LFrTMS=low-frequency transcranial magnetic stimulation; iTBS= intermittent theta-burst stimulation; HFrTMS = high-frequency transcranial magnetic stimulation; cTBS=continuous theta-burst stimulation; atDCS=anodal transcranial direct current stimulation; ctDCS=cathodal transcranial direct current stimulation; dtDCS=dual transcranial direct current stimulation; NMES=neuromuscular electrical stimulation; TENS=transcutaneous electrical nerve stimulation; rPMS= repetitive peripheral magnetic stimulation.

**B.** Percentage of male


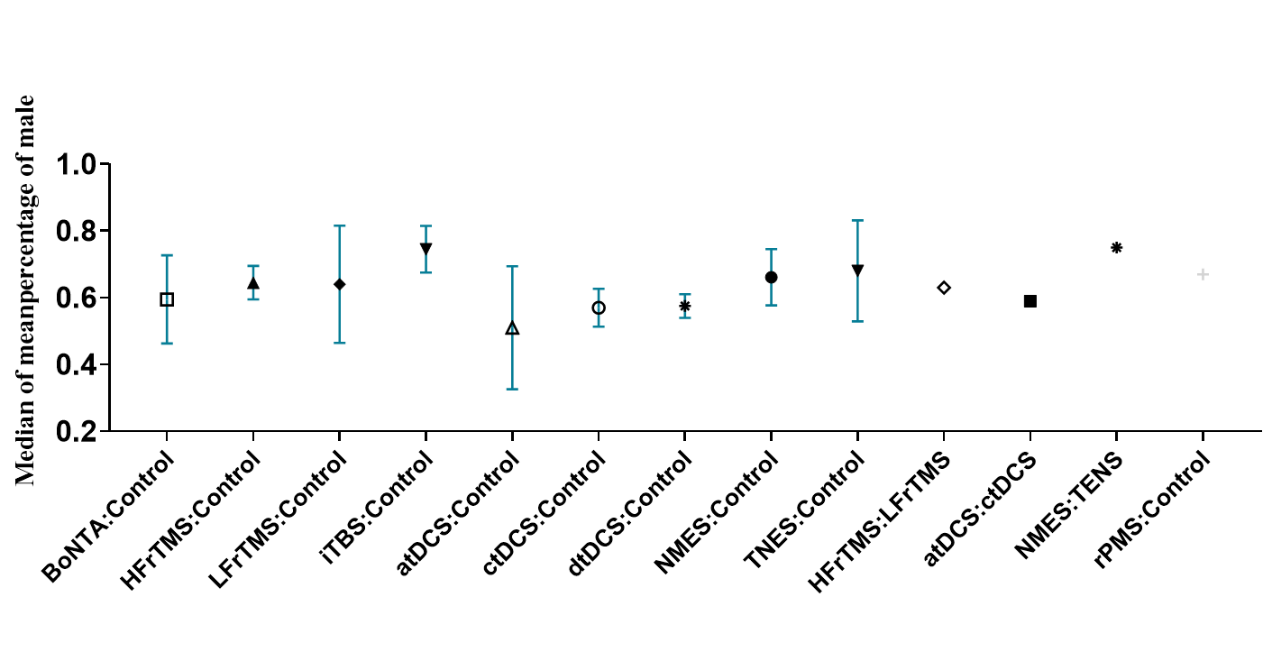
 The transitivity regarding the percentage of male based on network appears acceptable, as there is an overlap of characteristics across different comparisons, suggesting no significant differences among treatment comparisons (P=0.50).

Data are expressed as median (IQR). Kruskal-Wallis test was used for statistical analysis.

**Abbreviations:** BoNTA=botulinum toxin type A; LFrTMS=low-frequency transcranial magnetic stimulation; iTBS= intermittent theta-burst stimulation; HFrTMS = high-frequency transcranial magnetic stimulation; cTBS=continuous theta-burst stimulation; atDCS=anodal transcranial direct current stimulation; ctDCS=cathodal transcranial direct current stimulation; dtDCS=dual transcranial direct current stimulation; NMES=neuromuscular electrical stimulation; TENS=transcutaneous electrical nerve stimulation; rPMS= repetitive peripheral magnetic stimulation.

**C.** Percentage of ischemic stroke


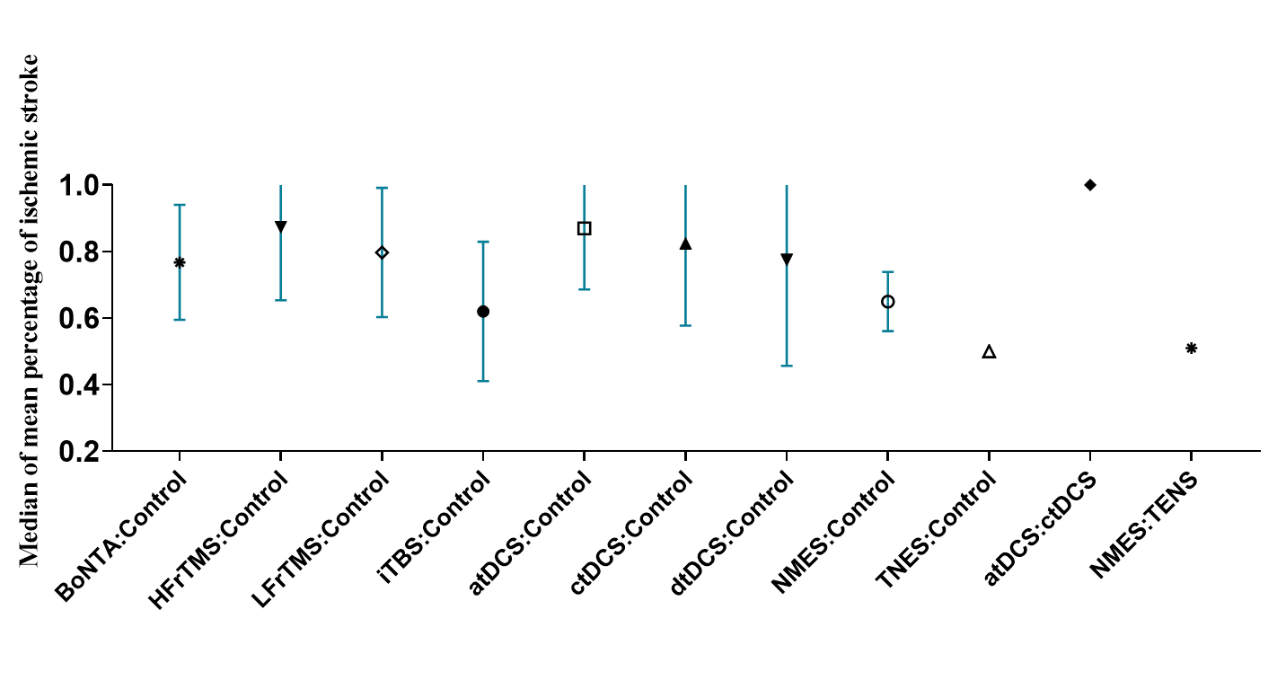
 The transitivity regarding the percentage of ischemic stroke based on network appears acceptable, as there is an overlap of characteristics across different comparisons, suggesting no significant differences among treatment comparisons (P=0.33).

Data are expressed as median (IQR). Kruskal-Wallis test was used for statistical analysis.

**Abbreviations:** BoNTA=botulinum toxin type A; LFrTMS=low-frequency transcranial magnetic stimulation; iTBS= intermittent theta-burst stimulation; HFrTMS = high-frequency transcranial magnetic stimulation; cTBS=continuous theta-burst stimulation; atDCS=anodal transcranial direct current stimulation; ctDCS=cathodal transcranial direct current stimulation; dtDCS=dual transcranial direct current stimulation; NMES=neuromuscular electrical stimulation; TENS=transcutaneous electrical nerve stimulation; rPMS= repetitive peripheral magnetic stimulation.

**D.** Mean time since stroke


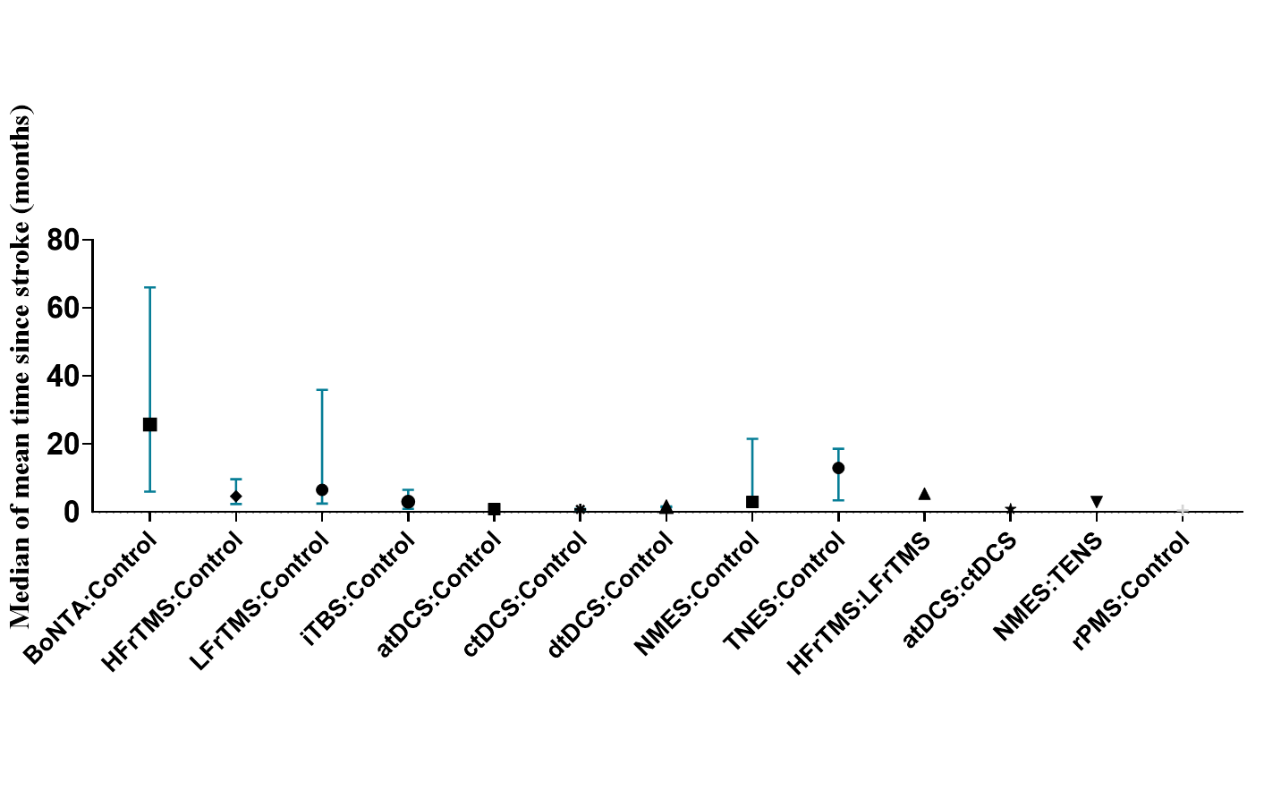
 The transitivity regarding the percentage of ischemic stroke based on network appears acceptable, as there is an overlap of characteristics across different comparisons, suggesting no significant differences among treatment comparisons (all P>0.05).

Data are expressed as median (IQR). Kruskal-Wallis test was used for statistical analysis.

**Abbreviations:** BoNTA=botulinum toxin type A; LFrTMS=low-frequency transcranial magnetic stimulation; iTBS= intermittent theta-burst stimulation; HFrTMS = high-frequency transcranial magnetic stimulation; cTBS=continuous theta-burst stimulation; atDCS=anodal transcranial direct current stimulation; ctDCS=cathodal transcranial direct current stimulation; dtDCS=dual transcranial direct current stimulation; NMES=neuromuscular electrical stimulation; TENS=transcutaneous electrical nerve stimulation; rPMS= repetitive peripheral magnetic stimulation.

# Appendix 10. Summary forest plots for each pairwise comparison.

## 10.1 Spasticity at short-term follow-up

A. Summary forest plots for each pairwise comparison for spasticity at short-term follow-up. Effects are expressed as the weighted mean difference (95% CI).

B. P value for each pairwise comparison for spasticity at mid-term follow-up.

| **Comparisons** | **P value** |
| --- | --- |
| atDCS-Control | 0.001 |
| LFrTMS-Control | <0.0001 |
| NMES-Control | <0.0001 |
| iTBS-Control | 0.20 |
| TENS-Control | 0.001 |
| HFrTMS-Control | 0.001 |
| LFrTMS-HFrTMS | 0.14 |
| BoNT-Control | <0.0001 |
| dtDCS-ctDCS | 0.60 |
| NMES-BoNT | 0.94 |
| ctDCS-Control | 0.009 |
| ctDCS-atDCS | 0.54 |
| cTBS-Control | 0.5 |
| cTBS-LFrTMS | 0.75 |
| TENS-BoNT | 0.008 |
| dtDCS-Control | <0.0001 |
| dtDCS-atDCS | 0.83 |
| TENS-NMES | 0.60 |
| LFrTMS-BoNT | <0.0001 |
| iTBS-HFrTMS | 0.99 |
| rPMS-Control | 0.031 |
| rPMS-iTBS | 0.74 |
| NMES-LFrTMS | 0.43 |

**Abbreviations:** BoNT=botulinum toxin; LFrTMS=low-frequency transcranial magnetic stimulation; iTBS= intermittent theta-burst stimulation; HFrTMS = high-frequency transcranial magnetic stimulation; cTBS=continuous theta-burst stimulation; atDCS=anodal transcranial direct current stimulation; ctDCS=cathodal transcranial direct current stimulation; dtDCS=dual transcranial direct current stimulation; NMES=neuromuscular electrical stimulation; TENS=transcutaneous electrical nerve stimulation; rPMS= repetitive peripheral magnetic stimulation.

## 10.2 Spasticity at mid-term follow-up

A. Summary forest plots for each pairwise comparison for spasticity at mid-term follow-up. Effects are expressed as the weighted mean difference (95% CI).

B. P value for each pairwise comparison for spasticity at mid-term follow-up.

| **Comparisons** | **P value** |
| --- | --- |
| LFrTMS-Control | <0.0001 |
| HFrTMS-Control | 0.034 |
| BoNT-Control | <0.0001 |
| dtDCS-ctDCS | 0.46 |
| NMES-Control | 0.014 |
| NMES-BoNT | 0.74 |
| cTBS-Control | 0.26 |
| cTBS-LFrTMS | 0.80 |
| TENS-BoNT | 0.12 |
| ctDCS-Control | <0.0001 |
| TENS-Control | 0.92 |
| TENS-NMES | 0.90 |
| LFrTMS-BoNT | <0.0001 |
| LFrTMS-HFrTMS | 0.69 |

**Abbreviations:** BoNT=botulinum toxin; LFrTMS=low-frequency transcranial magnetic stimulation; iTBS= intermittent theta-burst stimulation; HFrTMS = high-frequency transcranial magnetic stimulation; cTBS=continuous theta-burst stimulation; atDCS=anodal transcranial direct current stimulation; ctDCS=cathodal transcranial direct current stimulation; dtDCS=dual transcranial direct current stimulation; NMES=neuromuscular electrical stimulation; TENS=transcutaneous electrical nerve stimulation; rPMS= repetitive peripheral magnetic stimulation.

## 10.3 Motor function at short-term follow-up

A. Summary forest plots for each pairwise comparison for motor function at short-term follow-up. Effects are expressed as the weighted mean difference (95% CI).

 B. P value for each pairwise comparison for motor function at short-term follow-up.

| **Comparisons** | **P value** |
| --- | --- |
| atDCS-Control | <0.0001 |
| LFrTMS-Control | <0.0001 |
| iTBS-Control | 0.016 |
| NMES-Control | <0.0001 |
| HFrTMS-Control | <0.0001 |
| LFrTMS-HFrTMS | 0.052 |
| ctDCS-Control | <0.0001 |
| ctDCS-atDCS | 0.90 |
| TENS-Control | 0.0031 |
| cTBS-Control | 0.76 |
| cTBS-LFrTMS | 0.72 |
| BoNT-Control | <0.0001 |
| dtDCS-Control | <0.0001 |
| dtDCS-atDCS | 0.15 |
| TENS-NMES | 0.55 |
| LFrTMS-BoNT | 0.84 |
| NMES-LFrTMS | 0.14 |
| rPMS-Control | 0.24 |
| rPMS-iTBS | 0.72 |
| iTBS-HFrTMS | 0.79 |

## 10.4 Motor function at mid-term follow-up

A. Summary forest plots for each pairwise comparison for motor function at mid-term follow-up. Effects are expressed as the weighted mean difference (95% CI).

B. P value for each pairwise comparison for motor function at short-term follow-up.

| **Comparisons** | **P value** |
| --- | --- |
| LFrTMS-Control | 0.014 |
| HFrTMS-Control | <0.0001 |
| BoNT-Control | <0.0001 |
| cTBS-Control | 0.66 |
| cTBS-LFrTMS | 0.59 |
| NMES-Control | 0.41 |
| ctDCS-Control | 0.0021 |
| TENS-Control | 0.53 |
| TENS-NMES | 0.68 |
| LFrTMS-BoNT | 0.47 |
| LFrTMS-HFrTMS | 0.86 |

## 10.5 Acceptability

A. Summary forest plots for each pairwise comparison for acceptability. Effects are expressed as the risk ratio (95% CI). The hashed line indicates clinically important difference.

 B. P value for each pairwise comparison for motor function at short-term follow-up.

| **Comparisons** | **P value** |
| --- | --- |
| atDCS-Control | 1.00 |
| ctDCS-Control | 0.59 |
| ctDCS-atDCS | 0.51 |
| dtDCS-Control | 0.69 |
| TENS-Control | 0.95 |
| iTBS-Control | 0.61 |
| HFrTMS-Control | 0.40 |
| LFrTMS-Control | 0.76 |
| LFrTMS-HFrTMS | 0.62 |
| NMES-Control | 0.50 |
| TENS-NMES | 0.75 |
| BoNT-Control | 0.75 |

# Appendix 11. Detailed heterogeneities for direct meta-analyses.

## 11.1 Heterogeneity for spasticity at short-term follow-up

## 11.2 Heterogeneity for spasticity at mid-term follow-up

## 11.3 Heterogeneity for motor function at short-term follow-up

## 11.4 Heterogeneity for motor function at mid-term follow-up

## 11.5 Heterogeneity for acceptability

**Abbreviations:** BoNT=botulinum toxin; LFrTMS=low-frequency transcranial magnetic stimulation; iTBS= intermittent theta-burst stimulation; HFrTMS = high-frequency transcranial magnetic stimulation; cTBS=continuous theta-burst stimulation; atDCS=anodal transcranial direct current stimulation; ctDCS=cathodal transcranial direct current stimulation; dtDCS=dual transcranial direct current stimulation; NMES=neuromuscular electrical stimulation; TENS=transcutaneous electrical nerve stimulation; rPMS= repetitive peripheral magnetic stimulation.

# Appendix 12. Results of loop-specific heterogeneity estimates.

## 12.1 Loop-specific heterogeneity estimates for spasticity at short-term follow-up

## 12.2 Loop-specific heterogeneity estimates for spasticity at mid-term follow-up

## 12.3 Loop-specific heterogeneity estimates for motor function at short-term follow-up

**Abbreviations:** BoNT=botulinum toxin; LFrTMS=low-frequency transcranial magnetic stimulation; iTBS= intermittent theta-burst stimulation; HFrTMS=high-frequency transcranial magnetic stimulation; cTBS=continuous theta-burst stimulation; atDCS=anodal transcranial direct current stimulation; ctDCS=cathodal transcranial direct current stimulation; dtDCS=dual transcranial direct current stimulation; NMES=neuromuscular electrical stimulation; TENS=transcutaneous electrical nerve stimulation; rPMS= repetitive peripheral magnetic stimulation.

## 12.4 Loop-specific heterogeneity estimates for motor function at mid-term follow-up


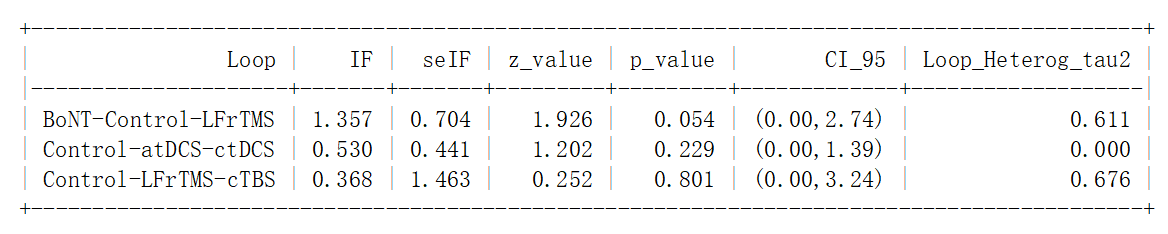


## 12.5 Loop-specific heterogeneity estimates for acceptability

**Abbreviations:** BoNT=botulinum toxin; LFrTMS=low-frequency transcranial magnetic stimulation; HFrTMS = high-frequency transcranial magnetic stimulation; cTBS=continuous theta-burst stimulation; atDCS=anodal transcranial direct current stimulation; ctDCS=cathodal transcranial direct current stimulation; NMES=neuromuscular electrical stimulation.

# Appendix 13. Results of node splitting.

## 13.1 Results of node splitting for spasticity at short-term follow-up

Note: A=Control; B=botulinum toxin; C=high-frequency transcranial magnetic stimulation; D=low-frequency transcranial magnetic stimulation; E= continuous theta-burst stimulation; F=intermittent theta-burst stimulation; G=anodal transcranial direct current stimulation; H=cathodal transcranial direct current stimulation; I=dual transcranial direct current stimulation; J=neuromuscular electrical stimulation; K=transcutaneous electrical nerve stimulation; L=repetitive peripheral magnetic stimulation.

## 13.2 Results of node splitting for spasticity at mid-term follow-up

Note: A=Control; B=botulinum toxin; C=high-frequency transcranial magnetic stimulation; D=low-frequency transcranial magnetic stimulation; E=continuous theta-burst stimulation; F=cathodal transcranial direct current stimulation; G=dual transcranial direct current stimulation, H=neuromuscular electrical stimulation; K=transcutaneous electrical nerve stimulation; i=repetitive peripheral magnetic stimulation.

## 13.3 Results of node splitting for motor function at short-term follow-up

Note: A=Control; B=botulinum toxin; C=high-frequency transcranial magnetic stimulation; D=low-frequency transcranial magnetic stimulation; E=continuous theta-burst stimulation; F= intermittent theta-burst stimulation; G=anodal transcranial direct current stimulation; H=cathodal transcranial direct current stimulation; I=neuromuscular electrical stimulation; J=transcutaneous electrical nerve stimulation; K=repetitive peripheral magnetic stimulation.

## 13.4 Results of node splitting for motor function at mid-term follow-up

Note: A=Control; B=botulinum toxin; C=high-frequency transcranial magnetic stimulation; D=low-frequency transcranial magnetic stimulation; E=continuous theta-burst stimulation; F=cathodal transcranial direct current stimulation; G=neuromuscular electrical stimulation; H=transcutaneous electrical nerve stimulation.

## 13.5 Results of node splitting for acceptability

Note: A=Control; B=botulinum toxin; C=high-frequency transcranial magnetic stimulation; D=low-frequency transcranial magnetic stimulation; E=continuous theta-burst stimulation; F= intermittent theta-burst stimulation; G=anodal transcranial direct current stimulation; H=cathodal transcranial direct current stimulation; I=dual transcranial direct current stimulation; J=neuromuscular electrical stimulation.

# Appendix 14. Results of design-by-treatment interaction model.

| **Outcome** | **df** | **χ^2^** | **P** |
| --- | --- | --- | --- |
| Spasticity (short-term) | 24 | 15.42 | 0.91 |
| Spasticity (mid-term) | 9 | 10.19 | 0.34 |
| Motor function (short-term)* | 16 | 20.18 | 0.21 |
| Motor function (mid-term) | 6 | 0.85 | 0.99 |
| Acceptability | 6 | 4.74 | 0.58 |

*Global inconsistency was adjusted by removing trials with dtDCS versus Control.

Strategy to explore global inconsistency for motor function at short-term.

| **STRATEGY** | **Study removed** | \| **χ^2^** \|  \|  \| \| --- \| --- \| --- \| | **Prob > χ^2^** | **Resolving inconsistency** |
| --- | --- | --- | --- | --- | --- | --- | --- |
| All studies | - | χ^2^ (18) = 52.37 | Prob > χ^2^ = 0.0000 | - |
| Node split | Trials with dtDCS versus Control | χ^2^ (16) = 20.18 | Prob > χ^2^ = 0.21 | Resolved |

Significant local inconsistency was identified in A-I (dtDCS-Control).

# Appendix 15. Network meta-analysis results for each intervention for each outcome.

## 15.1 Spasticity at short-term follow-up

A. Summary forest plots for network meta-analysis results for spasticity at short-term follow-up. Effects are expressed as the weighted mean difference (95% CI). The hashed line indicates clinically important difference.

B. P value for each comparison for spasticity at mid-term follow-up.

| **Comparisons** | **P value** |
| --- | --- |
| BoNT vs Control | <0.0001 |
| HFrTMS vs Control | 0.0024 |
| LFrTMS vs Control | <0.0001 |
| cTBS vs Control | 0.48 |
| iTBS vs Control | 0.12 |
| atDCS vs Control | 0.0008 |
| ctDCS vs Control | 0.0069 |
| dtDCS vs Control | 0.0071 |
| NMES vs Control | <0.0001 |
| TENS vs Control | 0.0037 |
| rPMS vs Control | 0.39 |
| HFrTMS vs BoNT | <0.0001 |
| LFrTMS vs BoNT | <0.0001 |
| cTBS vs BoNT | 0.17 |
| iTBS vs BoNT | 0.0032 |
| atDCS vs BoNT | 0.013 |
| ctDCS vs BoNT | 0.032 |
| dtDCS vs BoNT | 0.038 |
| NMES vs BoNT | <0.0001 |
| TENS vs BoNT | 0.018 |
| rPMS vs BoNT | 0.0045 |
| LFrTMS vs HFrTMS | 0.49 |
| cTBS vs HFrTMS | 0.93 |
| iTBS vs HFrTMS | 0.84 |
| atDCS vs HFrTMS | 0.40 |
| ctDCS vs HFrTMS | 0.49 |
| dtDCS vs HFrTMS | 0.48 |
| NMES vs HFrTMS | 0.26 |
| TENS vs HFrTMS | 0.48 |
| rPMS vs HFrTMS | 0.60 |
| cTBS vs LFrTMS | 0.77 |
| iTBS vs LFrTMS | 0.52 |
| atDCS vs LFrTMS | 0.68 |
| ctDCS vs LFrTMS | 0.77 |
| dtDCS vs LFrTMS | 0.75 |
| NMES vs LFrTMS | 0.54 |
| TENS vs LFrTMS | 0.77 |
| rPMS vs LFrTMS | 0.36 |
| iTBS vs cTBS | 0.99 |
| atDCS vs cTBS | 0.67 |
| ctDCS vs cTBS | 0.69 |
| dtDCS vs cTBS | 0.68 |
| NMES vs cTBS | 0.66 |
| TENS vs cTBS | 0.70 |
| rPMS vs cTBS | 0.84 |
| atDCS vs iTBS | 0.41 |
| ctDCS vs iTBS | 0.47 |
| dtDCS vs iTBS | 0.46 |
| NMES vs iTBS | 0.34 |
| TENS vs iTBS | 0.47 |
| rPMS vs iTBS | 0.74 |
| ctDCS vs atDCS | 0.95 |
| dtDCS vs atDCS | 0.98 |
| NMES vs atDCS | 0.99 |
| TENS vs atDCS | 0.94 |
| rPMS vs atDCS | 0.29 |
| dtDCS vs ctDCS | 0.98 |
| NMES vs ctDCS | 0.96 |
| TENS vs ctDCS | 0.99 |
| rPMS vs ctDCS | 0.34 |
| NMES vs dtDCS | 0.99 |
| TENS vs dtDCS | 0.96 |
| rPMS vs dtDCS | 0.33 |
| TENS vs NMES | 0.94 |
| rPMS vs NMES | 0.24 |
| rPMS vs TENS | 0.33 |

**Abbreviations:** BoNT=botulinum toxin; LFrTMS=low-frequency transcranial magnetic stimulation; iTBS= intermittent theta-burst stimulation; HFrTMS = high-frequency transcranial magnetic stimulation; cTBS=continuous theta-burst stimulation; atDCS=anodal transcranial direct current stimulation; ctDCS=cathodal transcranial direct current stimulation; dtDCS=dual transcranial direct current stimulation; NMES=neuromuscular electrical stimulation; TENS=transcutaneous electrical nerve stimulation; rPMS= repetitive peripheral magnetic stimulation.

## 15.2 Spasticity at mid-term follow-up

A. Summary forest plots for network meta-analysis results for spasticity at mid-term follow-up. Effects are expressed as the weighted mean difference (95% CI). The hashed line indicates clinically important difference.

B. P value for each comparison for spasticity at mid-term follow-up.

| **Comparisons** | **P value** |
| --- | --- |
| BoNT vs Control | <0.0001 |
| HFrTMS vs Control | 0.32 |
| LFrTMS vs Control | 0.53 |
| cTBS vs Control | 0.55 |
| ctDCS vs Control | 0.0001 |
| dtDCS vs Control | 0.047 |
| NMES vs Control | 0.096 |
| TENS vs Control | 0.44 |
| HFrTMS vs BoNT | 0.0083 |
| LFrTMS vs BoNT | <0.0001 |
| cTBS vs BoNT | 0.22 |
| ctDCS vs BoNT | 0.022 |
| dtDCS vs BoNT | 0.30 |
| NMES vs BoNT | 0.048 |
| TENS vs BoNT | 0.048 |
| LFrTMS vs HFrTMS | 0.64 |
| cTBS vs HFrTMS | 0.92 |
| ctDCS vs HFrTMS | 0.00 |
| dtDCS vs HFrTMS | 0.09 |
| NMES vs HFrTMS | 0.63 |
| TENS vs HFrTMS | 0.98 |
| cTBS vs LFrTMS | 0.70 |
| ctDCS vs LFrTMS | 0.0005 |
| dtDCS vs LFrTMS | 0.065 |
| NMES vs LFrTMS | 0.32 |
| TENS vs LFrTMS | 0.70 |
| ctDCS vs cTBS | 0.0099 |
| dtDCS vs cTBS | 0.14 |
| NMES vs cTBS | 0.84 |
| TENS vs cTBS | 0.94 |
| dtDCS vs ctDCS | 0.54 |
| NMES vs ctDCS | 0.0037 |
| TENS vs ctDCS | 0.0029 |
| NMES vs dtDCS | 0.13 |
| TENS vs dtDCS | 0.10 |
| TENS vs NMES | 0.66 |

**Abbreviations:** BoNT=botulinum toxin; LFrTMS=low-frequency transcranial magnetic stimulation; iTBS= intermittent theta-burst stimulation; HFrTMS = high-frequency transcranial magnetic stimulation; cTBS=continuous theta-burst stimulation; atDCS=anodal transcranial direct current stimulation; ctDCS=cathodal transcranial direct current stimulation; dtDCS=dual transcranial direct current stimulation; NMES=neuromuscular electrical stimulation; TENS=transcutaneous electrical nerve stimulation; rPMS= repetitive peripheral magnetic stimulation.

## 15.3 Motor function at short-term follow-up

A. Summary forest plots for network meta-analysis results for motor function at short-term follow-up. Effects are expressed as the weighted mean difference (95% CI). The hashed line indicates clinically important difference.

B. P value for each comparison for motor function at short-term follow-up.

| **Comparisons** | **P value** |
| --- | --- |
| BoNT vs Control | <0.0001 |
| HFrTMS vs Control | <0.0001 |
| LFrTMS vs Control | <0.0001 |
| cTBS vs Control | 0.72 |
| iTBS vs Control | 0.07 |
| atDCS vs Control | <0.0001 |
| ctDCS vs Control | 0.0026 |
| NMES vs Control | <0.0001 |
| TENS vs Control | 0.09 |
| rPMS vs Control | 0.15 |
| HFrTMS vs BoNT | 0.38 |
| LFrTMS vs BoNT | 0.59 |
| cTBS vs BoNT | 0.67 |
| iTBS vs BoNT | 0.35 |
| atDCS vs BoNT | 0.53 |
| ctDCS vs BoNT | 0.82 |
| NMES vs BoNT | 0.98 |
| TENS vs BoNT | 0.35 |
| rPMS vs BoNT | 0.56 |
| LFrTMS vs HFrTMS | 0.63 |
| cTBS vs HFrTMS | 0.78 |
| iTBS vs HFrTMS | 0.68 |
| atDCS vs HFrTMS | 0.26 |
| ctDCS vs HFrTMS | 0.75 |
| NMES vs HFrTMS | 0.45 |
| TENS vs HFrTMS | 0.68 |
| rPMS vs HFrTMS | 0.85 |
| cTBS vs LFrTMS | 0.72 |
| iTBS vs LFrTMS | 0.49 |
| atDCS vs LFrTMS | 0.37 |
| ctDCS vs LFrTMS | 0.96 |
| NMES vs LFrTMS | 0.68 |
| TENS vs LFrTMS | 0.49 |
| rPMS vs LFrTMS | 0.69 |
| iTBS vs cTBS | 0.88 |
| atDCS vs cTBS | 0.57 |
| ctDCS vs cTBS | 0.72 |
| NMES vs cTBS | 0.67 |
| TENS vs cTBS | 0.89 |
| rPMS vs cTBS | 0.85 |
| atDCS vs iTBS | 0.24 |
| ctDCS vs iTBS | 0.56 |
| NMES vs iTBS | 0.38 |
| TENS vs iTBS | 0.98 |
| rPMS vs iTBS | 0.91 |
| ctDCS vs atDCS | 0.49 |
| NMES vs atDCS | 0.55 |
| TENS vs atDCS | 0.24 |
| rPMS vs atDCS | 0.39 |
| NMES vs ctDCS | 0.84 |
| TENS vs ctDCS | 0.55 |
| rPMS vs ctDCS | 0.71 |
| TENS vs NMES | 0.36 |
| rPMS vs NMES | 0.58 |
| rPMS vs TENS | 0.90 |

**Abbreviations:** BoNT=botulinum toxin; LFrTMS=low-frequency transcranial magnetic stimulation; iTBS= intermittent theta-burst stimulation; HFrTMS = high-frequency transcranial magnetic stimulation; cTBS=continuous theta-burst stimulation; atDCS=anodal transcranial direct current stimulation; ctDCS=cathodal transcranial direct current stimulation; dtDCS=dual transcranial direct current stimulation; NMES=neuromuscular electrical stimulation; TENS=transcutaneous electrical nerve stimulation; rPMS= repetitive peripheral magnetic stimulation.

## 15.4 Motor function at mid-term follow-up

A. Summary forest plots for network meta-analysis results for motor function at mid-term follow-up. Effects are expressed as the weighted mean difference (95% CI). The hashed line indicates clinically important difference.

B. P value for each comparison for motor function at mid-term follow-up.

| **Comparisons** | **P value** |
| --- | --- |
| BoNT vs Control | <0.0001 |
| HFrTMS vs Control | 0.031 |
| LFrTMS vs Control | 0.014 |
| cTBS vs Control | 0.85 |
| ctDCS vs Control | 0.014 |
| NMES vs Control | 0.49 |
| TENS vs Control | 0.98 |
| HFrTMS vs BoNT | 0.91 |
| LFrTMS vs BoNT | 0.32 |
| cTBS vs BoNT | 0.60 |
| ctDCS vs BoNT | 0.13 |
| NMES vs BoNT | 0.16 |
| TENS vs BoNT | 0.16 |
| LFrTMS vs HFrTMS | 0.52 |
| cTBS vs HFrTMS | 0.59 |
| ctDCS vs HFrTMS | 0.17 |
| NMES vs HFrTMS | 0.27 |
| TENS vs HFrTMS | 0.22 |
| cTBS vs LFrTMS | 0.75 |
| ctDCS vs LFrTMS | 0.08 |
| NMES vs LFrTMS | 0.48 |
| TENS vs LFrTMS | 0.36 |
| ctDCS vs cTBS | 0.17 |
| NMES vs cTBS | 0.97 |
| TENS vs cTBS | 0.88 |
| NMES vs ctDCS | 0.046 |
| TENS vs ctDCS | 0.039 |
| TENS vs NMES | 0.65 |

## 15.5 Acceptability

A. Summary forest plots for network meta-analysis results for acceptability. Effects are expressed as the risk ratio (95% CI). The hashed line indicates clinically important difference.

B. P value for each comparison for acceptability.

| **Comparisons** | **P value** |
| --- | --- |
| BoNT vs Control | 0.39 |
| HFrTMS vs Control | 0.42 |
| LFrTMS vs Control | 0.36 |
| iTBS vs Control | 0.46 |
| atDCS vs Control | 0.72 |
| ctDCS vs Control | 0.86 |
| dtDCS vs Control | 0.56 |
| NMES vs Control | 0.33 |
| TENS vs Control | 0.54 |
| HFrTMS vs BoNT | 0.41 |
| LFrTMS vs BoNT | 0.36 |
| iTBS vs BoNT | 0.46 |
| atDCS vs BoNT | 0.71 |
| ctDCS vs BoNT | 0.86 |
| dtDCS vs BoNT | 0.55 |
| NMES vs BoNT | 0.33 |
| TENS vs BoNT | 0.54 |
| LFrTMS vs HFrTMS | 0.48 |
| iTBS vs HFrTMS | 0.59 |
| atDCS vs HFrTMS | 0.78 |
| ctDCS vs HFrTMS | 0.89 |
| dtDCS vs HFrTMS | 0.66 |
| NMES vs HFrTMS | 0.53 |
| TENS vs HFrTMS | 0.67 |
| iTBS vs LFrTMS | 0.60 |
| atDCS vs LFrTMS | 0.79 |
| ctDCS vs LFrTMS | 0.89 |
| dtDCS vs LFrTMS | 0.66 |
| NMES vs LFrTMS | 0.52 |
| TENS vs LFrTMS | 0.67 |
| atDCS vs iTBS | 0.84 |
| ctDCS vs iTBS | 0.92 |
| dtDCS vs iTBS | 0.74 |
| NMES vs iTBS | 0.67 |
| TENS vs iTBS | 0.76 |
| ctDCS vs atDCS | 0.89 |
| dtDCS vs atDCS | 0.72 |
| NMES vs atDCS | 0.67 |
| TENS vs atDCS | 0.76 |
| dtDCS vs ctDCS | 0.42 |
| NMES vs ctDCS | 0.34 |
| TENS vs ctDCS | 0.50 |
| NMES vs dtDCS | 0.74 |
| TENS vs dtDCS | 0.81 |
| TENS vs NMES | 0.63 |

# Appendix 16. Surface Under the Cumulative Ranking (SUCRA) and mean rank.

## 16.1 Relative ranking of treatments of interest on spasticity at short-term follow-up

Ranking-cumulative probability plots for different treatment options for spasticity at short-term follow-up.

Abbreviations: BoNT=botulinum toxin; LFrTMS=low-frequency transcranial magnetic stimulation; HFrTMS=high-frequency transcranial magnetic stimulation; cTBS=continuous theta-burst stimulation; iTBS=intermittent theta-burst stimulation; atDCS=anodal transcranial direct current stimulation; ctDCS=cathodal transcranial direct current stimulation; dtDCS=dual transcranial direct current stimulation; NMES=neuromuscular electrical stimulation; TENS=transcutaneous electrical nerve stimulation; rPMS=repetitive peripheral magnetic stimulation.

## 16.2 Relative ranking of treatments of interest on spasticity at mid-term follow-up

Ranking-cumulative probability plots for different treatment options for spasticity at mid-term follow-up.

Abbreviations: BoNT=botulinum toxin; LFrTMS=low-frequency transcranial magnetic stimulation; iTBS= intermittent theta-burst stimulation; HFrTMS = high-frequency transcranial magnetic stimulation; cTBS=continuous theta-burst stimulation; atDCS=anodal transcranial direct current stimulation; ctDCS=cathodal transcranial direct current stimulation; dtDCS=dual transcranial direct current stimulation; NMES=neuromuscular electrical stimulation; TENS=transcutaneous electrical nerve stimulation; rPMS= repetitive peripheral magnetic stimulation.

## 16.3 Relative ranking of treatments of interest on motor function at short-term follow-up

Ranking-cumulative probability plots for different treatment options for motor function at mid-term follow-up.

Abbreviations: BoNT=botulinum toxin; HFrTMS = high-frequency transcranial magnetic stimulation; LFrTMS=low-frequency transcranial magnetic stimulation; cTBS=continuous theta-burst stimulation; iTBS= intermittent theta-burst stimulation; atDCS=anodal transcranial direct current stimulation; ctDCS=cathodal transcranial direct current stimulation; dtDCS=dual transcranial direct current stimulation; NMES=neuromuscular electrical stimulation; TENS=transcutaneous electrical nerve stimulation; rPMS= repetitive peripheral magnetic stimulation.

## 16.4 Relative ranking of treatments of interest on motor function at mid-term follow-up

Ranking-cumulative probability plots for different treatment options for motor function at mid-term follow-up.

**Abbreviations:** BoNT=botulinum toxin; LFrTMS=low-frequency transcranial magnetic stimulation; iTBS= intermittent theta-burst stimulation; HFrTMS = high-frequency transcranial magnetic stimulation; cTBS=continuous theta-burst stimulation; atDCS=anodal transcranial direct current stimulation; ctDCS=cathodal transcranial direct current stimulation; NMES=neuromuscular electrical stimulation; TENS=transcutaneous electrical nerve stimulation.

## 16.5 Relative ranking of treatments of interest on acceptability

Ranking-cumulative probability plots for different treatment options for acceptability.

**Abbreviations:** BoNT=botulinum toxin LFrTMS=low-frequency transcranial magnetic stimulation; iTBS= intermittent theta-burst stimulation; HFrTMS = high-frequency transcranial magnetic stimulation; atDCS=anodal transcranial direct current stimulation; ctDCS=cathodal transcranial direct current stimulation; dtDCS=dual transcranial direct current stimulation; NMES=neuromuscular electrical stimulation; TENS=transcutaneous electrical nerve stimulation.

# Appendix 17. Sensitivity analysis.

## 17.1 Exclusion of studies that measured spasticity on lower extremity

### 17.1.1 Spasticity at short-term follow-up

*a. Network plots of available comparisons*

Network plots of available comparisons between different types of therapies on spasticity at short-term follow-up. The size of the nodes is proportional to the number of studies included in each intervention, and the line width corresponds to studies directly comparing the two interventions.

**Abbreviations:** BoNT=botulinum toxin; LFrTMS=low-frequency transcranial magnetic stimulation; iTBS= intermittent theta-burst stimulation; HFrTMS = high-frequency transcranial magnetic stimulation; cTBS=continuous theta-burst stimulation; atDCS=anodal transcranial direct current stimulation; ctDCS=cathodal transcranial direct current stimulation; dtDCS=dual transcranial direct current stimulation; NMES=neuromuscular electrical stimulation; TENS=transcutaneous electrical nerve stimulation; rPMS= repetitive peripheral magnetic stimulation.

*b. Results of design-by-treatment interaction model*

*c. Result of node sidesplit*

Note: A=Control; B=botulinum toxin; C=high-frequency transcranial magnetic stimulation; D=low-frequency transcranial magnetic stimulation; E=continuous theta-burst stimulation; F= intermittent theta-burst stimulation; G=anodal transcranial direct current stimulation; H=cathodal transcranial direct current stimulation; I=dual transcranial direct current stimulation; J=neuromuscular electrical stimulation; K=transcutaneous electrical nerve stimulation; L=repetitive peripheral magnetic stimulation.

*****d. Results of loop-specific heterogeneity estimates*

**Abbreviations:** BoNT=botulinum toxin; LFrTMS=low-frequency transcranial magnetic stimulation; iTBS= intermittent theta-burst stimulation; HFrTMS = high-frequency transcranial magnetic stimulation; cTBS=continuous theta-burst stimulation; atDCS=anodal transcranial direct current stimulation; ctDCS=cathodal transcranial direct current stimulation; dtDCS=dual transcranial direct current stimulation; NMES=neuromuscular electrical stimulation; TENS=transcutaneous electrical nerve stimulation; rPMS= repetitive peripheral magnetic stimulation.

*e. Summary forest plots for network meta-analysis results for spasticity at short-term follow-up. Effects are expressed as the weighted mean difference (95% CI). The hashed line indicates clinically important difference.*

**Abbreviations:** BoNT=botulinum toxin; LFrTMS=low-frequency transcranial magnetic stimulation; iTBS= intermittent theta-burst stimulation; HFrTMS = high-frequency transcranial magnetic stimulation; cTBS=continuous theta-burst stimulation; atDCS=anodal transcranial direct current stimulation; ctDCS=cathodal transcranial direct current stimulation; dtDCS=dual transcranial direct current stimulation; NMES=neuromuscular electrical stimulation; TENS=transcutaneous electrical nerve stimulation; rPMS= repetitive peripheral magnetic stimulation.

*f. P value for each comparison for spasticity at short-term follow-up.*

| **Comparisons** | **P value** |
| --- | --- |
| BoNT vs Control | <0.0001 |
| HFrTMS vs Control | 0.0016 |
| LFrTMS vs Control | <0.0001 |
| cTBS vs Control | 0.47 |
| iTBS vs Control | 0.12 |
| atDCS vs Control | 0.0011 |
| ctDCS vs Control | 0.008 |
| dtDCS vs Control | 0.0055 |
| NMES vs Control | <0.0001 |
| TENS vs Control | 0.13 |
| rPMS vs Control | 0.36 |
| HFrTMS vs BoNT | 0.0093 |
| LFrTMS vs BoNT | 0.0002 |
| cTBS vs BoNT | 0.21 |
| iTBS vs BoNT | 0.014 |
| atDCS vs BoNT | 0.036 |
| ctDCS vs BoNT | 0.06 |
| dtDCS vs BoNT | 0.072 |
| NMES vs BoNT | 0.0005 |
| TENS vs BoNT | 0.016 |
| rPMS vs BoNT | 0.0084 |
| LFrTMS vs HFrTMS | 0.98 |
| cTBS vs HFrTMS | 0.77 |
| iTBS vs HFrTMS | 0.62 |
| atDCS vs HFrTMS | 0.79 |
| ctDCS vs HFrTMS | 0.83 |
| dtDCS vs HFrTMS | 0.80 |
| NMES vs HFrTMS | 0.90 |
| TENS vs HFrTMS | 0.64 |
| rPMS vs HFrTMS | 0.40 |
| cTBS vs LFrTMS | 0.76 |
| iTBS vs LFrTMS | 0.59 |
| atDCS vs LFrTMS | 0.73 |
| ctDCS vs LFrTMS | 0.79 |
| dtDCS vs LFrTMS | 0.76 |
| NMES vs LFrTMS | 0.89 |
| TENS vs LFrTMS | 0.61 |
| rPMS vs LFrTMS | 0.36 |
| iTBS vs cTBS | 0.99 |
| atDCS vs cTBS | 0.68 |
| ctDCS vs cTBS | 0.70 |
| dtDCS vs cTBS | 0.68 |
| NMES vs cTBS | 0.80 |
| TENS vs cTBS | 0.98 |
| rPMS vs cTBS | 0.84 |
| atDCS vs iTBS | 0.48 |
| ctDCS vs iTBS | 0.53 |
| dtDCS vs iTBS | 0.50 |
| NMES vs iTBS | 0.66 |
| TENS vs iTBS | 0.99 |
| rPMS vs iTBS | 0.71 |
| ctDCS vs atDCS | 0.97 |
| dtDCS vs atDCS | 0.99 |
| NMES vs atDCS | 0.67 |
| TENS vs atDCS | 0.50 |
| rPMS vs atDCS | 0.30 |
| dtDCS vs ctDCS | 0.96 |
| NMES vs ctDCS | 0.73 |
| TENS vs ctDCS | 0.55 |
| rPMS vs ctDCS | 0.34 |
| NMES vs dtDCS | 0.70 |
| TENS vs dtDCS | 0.52 |
| rPMS vs dtDCS | 0.33 |
| TENS vs NMES | 0.67 |
| rPMS vs NMES | 0.41 |
| rPMS vs TENS | 0.72 |

*g. SUCRA value and mean rank*

*h. Ranking-cumulative probability plot*

**Abbreviations:** BoNT=botulinum toxin; LFrTMS=low-frequency transcranial magnetic stimulation; iTBS= intermittent theta-burst stimulation; HFrTMS = high-frequency transcranial magnetic stimulation; cTBS=continuous theta-burst stimulation; atDCS=anodal transcranial direct current stimulation; ctDCS=cathodal transcranial direct current stimulation; dtDCS=dual transcranial direct current stimulation; NMES=neuromuscular electrical stimulation; TENS=transcutaneous electrical nerve stimulation; rPMS= repetitive peripheral magnetic stimulation.

*i. Results of pairwise meta-analysis. Effects are expressed as the weighted mean difference (95% CI).*

Summary forest plots of each intervention for spasticity at short-term follow-up. Effects are expressed as the weighted mean difference (95% CI).

*j. P value for each pairwise comparison for spasticity at short-term follow-up.*

| **Comparisons** | **P value** |
| --- | --- |
| atDCS-Control | 0.0030 |
| LFrTMS-Control | <0.0001 |
| NMES-Control | <0.0001 |
| iTBS-Control | 0.20 |
| HFrTMS-Control | <0.0001 |
| LFrTMS-HFrTMS | 0.018 |
| BoNT-Control | <0.0001 |
| dtDCS-ctDCS | 0.60 |
| NMES-BoNT | 0.94 |
| ctDCS-Control | 0.0090 |
| ctDCS-atDCS | 0.54 |
| TENS-Control | 0.072 |
| cTBS-Control | 0.5 |
| cTBS-LFrTMS | 0.75 |
| TENS-BoNT | 0.0080 |
| dtDCS-Control | <0.0001 |
| dtDCS-atDCS | 0.83 |
| TENS-NMES | 0.60 |
| LFrTMS-BoNT | <0.0001 |
| iTBS-HFrTMS | 1.00 |
| rPMS-Control | 0.024 |
| rPMS-iTBS | 0.74 |
| NMES-LFrTMS | 0.43 |

**Abbreviations:** BoNT=botulinum toxin; LFrTMS=low-frequency transcranial magnetic stimulation; iTBS= intermittent theta-burst stimulation; HFrTMS = high-frequency transcranial magnetic stimulation; cTBS=continuous theta-burst stimulation; atDCS=anodal transcranial direct current stimulation; ctDCS=cathodal transcranial direct current stimulation; dtDCS=dual transcranial direct current stimulation; NMES=neuromuscular electrical stimulation; TENS=transcutaneous electrical nerve stimulation; rPMS= repetitive peripheral magnetic stimulation.

### 17.1.2 Spasticity at mid-term follow-up

*a. Network plots of available comparisons*

**

Network plots of available comparisons between different types of therapies on spasticity at mid-term follow-up. The size of the nodes is proportional to the number of studies included in each intervention, and the line width corresponds to studies directly comparing the two interventions.

**Abbreviations:** BoNT=botulinum toxin; LFrTMS=low-frequency transcranial magnetic stimulation; iTBS= intermittent theta-burst stimulation; HFrTMS = high-frequency transcranial magnetic stimulation; cTBS=continuous theta-burst stimulation; atDCS=anodal transcranial direct current stimulation; ctDCS=cathodal transcranial direct current stimulation; dtDCS=dual transcranial direct current stimulation; NMES=neuromuscular electrical stimulation; TENS=transcutaneous electrical nerve stimulation.

*b. Results of design-by-treatment interaction model*

******

*c. Result of node sidesplit*

Note: A=Control; B=botulinum toxin; C=high-frequency transcranial magnetic stimulation; D=low-frequency transcranial magnetic stimulation; E=continuous theta-burst stimulation; F=cathodal transcranial direct current stimulation; G=dual transcranial direct current stimulation, H=neuromuscular electrical stimulation; K=transcutaneous electrical nerve stimulation; i=repetitive peripheral magnetic stimulation.

*d. Results of loop-specific heterogeneity estimates*

*e. Summary forest plots for network meta-analysis results for spasticity at mid-term follow-up. Effects are expressed as the weighted mean difference (95% CI). The hashed line indicates clinically important difference.*

**

**Abbreviations:** BoNT=botulinum toxin; LFrTMS=low-frequency transcranial magnetic stimulation; iTBS= intermittent theta-burst stimulation; HFrTMS = high-frequency transcranial magnetic stimulation; cTBS=continuous theta-burst stimulation; atDCS=anodal transcranial direct current stimulation; ctDCS=cathodal transcranial direct current stimulation; dtDCS=dual transcranial direct current stimulation; NMES=neuromuscular electrical stimulation; TENS=transcutaneous electrical nerve stimulation; rPMS= repetitive peripheral magnetic stimulation.

*f. P value for each comparison for spasticity at mid-term follow-up.*

| **Comparisons** | **P value** |
| --- | --- |
| BoNT vs Control | <0.0001 |
| HFrTMS vs Control | 0.38 |
| LFrTMS vs Control | 0.49 |
| cTBS vs Control | 0.51 |
| ctDCS vs Control | 0.0002 |
| dtDCS vs Control | 0.049 |
| NMES vs Control | 0.12 |
| TENS vs Control | 0.61 |
| HFrTMS vs BoNT | 0.30 |
| LFrTMS vs BoNT | 0.0058 |
| cTBS vs BoNT | 0.30 |
| ctDCS vs BoNT | 0.02 |
| dtDCS vs BoNT | 0.29 |
| NMES vs BoNT | 0.077 |
| TENS vs BoNT | 0.12 |
| LFrTMS vs HFrTMS | 0.66 |
| cTBS vs HFrTMS | 0.93 |
| ctDCS vs HFrTMS | 0.012 |
| dtDCS vs HFrTMS | 0.16 |
| NMES vs HFrTMS | 0.99 |
| TENS vs HFrTMS | 0.76 |
| cTBS vs LFrTMS | 0.74 |
| ctDCS vs LFrTMS | 0.0012 |
| dtDCS vs LFrTMS | 0.083 |
| NMES vs LFrTMS | 0.52 |
| TENS vs LFrTMS | 0.93 |
| ctDCS vs cTBS | 0.013 |
| dtDCS vs cTBS | 0.15 |
| NMES vs cTBS | 0.91 |
| TENS vs cTBS | 0.85 |
| dtDCS vs ctDCS | 0.54 |
| NMES vs ctDCS | 0.0041 |
| TENS vs ctDCS | 0.0048 |
| NMES vs dtDCS | 0.13 |
| TENS vs dtDCS | 0.11 |
| TENS vs NMES | 0.65 |

*g. SUCRA value and mean rank*

**

*h. Ranking-cumulative probability plot*

*i. Results of pairwise meta-analysis. Effects are expressed as the weighted mean difference (95% CI).*

*j. P value for each pairwise comparison for spasticity at mid-term follow-up.*

| **Comparisons** | **P value** |
| --- | --- |
| BoNT-Control | <0.0001 |
| cTBS-Control | 0.26 |
| cTBS-LFrTMS | 0.80 |
| ctDCS-Control | <0.0001 |
| dtDCS-ctDCS | 0.46 |
| HFrTMS-Control | 0.020 |
| LFrTMS-BoNT | <0.0001 |
| LFrTMS-Control | <0.0001 |
| NMES-BoNT | 0.74 |
| NMES-Control | 0.014 |
| TENS-Control | 0.92 |
| TENS-NMES | 0.90 |

**Abbreviations:** BoNT=botulinum toxin; LFrTMS=low-frequency transcranial magnetic stimulation; iTBS= intermittent theta-burst stimulation; HFrTMS = high-frequency transcranial magnetic stimulation; cTBS=continuous theta-burst stimulation; atDCS=anodal transcranial direct current stimulation; ctDCS=cathodal transcranial direct current stimulation; dtDCS=dual transcranial direct current stimulation; NMES=neuromuscular electrical stimulation; TENS=transcutaneous electrical nerve stimulation; rPMS= repetitive peripheral magnetic stimulation.

### 17.1.3 Motor function at short-term follow-up

*a. Network plots of available comparisons*

Network plots of available comparisons between different types of therapies on motor function at short-term follow-up. The size of the nodes is proportional to the number of studies included in each intervention, and the line width corresponds to studies directly comparing the two interventions.

**Abbreviations:** BoNT=botulinum toxin; LFrTMS=low-frequency transcranial magnetic stimulation; iTBS= intermittent theta-burst stimulation; HFrTMS = high-frequency transcranial magnetic stimulation; cTBS=continuous theta-burst stimulation; atDCS=anodal transcranial direct current stimulation; ctDCS=cathodal transcranial direct current stimulation; NMES=neuromuscular electrical stimulation; TENS=transcutaneous electrical nerve stimulation; rPMS= repetitive peripheral magnetic stimulation.

*b. Results of design-by-treatment interaction model*

*chi2( 14) = 19.51*

*Prob > chi2 = 0.1464*

*c. Result of node sidesplit*

Note: A=Control; B=botulinum toxin; C=high-frequency transcranial magnetic stimulation; D=low-frequency transcranial magnetic stimulation; E=continuous theta-burst stimulation; F= intermittent theta-burst stimulation; G=anodal transcranial direct current stimulation; H=cathodal transcranial direct current stimulation; I=dual transcranial direct current stimulation; J=neuromuscular electrical stimulation; K=transcutaneous electrical nerve stimulation; L=repetitive peripheral magnetic stimulation.

*d. Results of loop-specific heterogeneity estimates*

*e. Summary forest plots for network meta-analysis results for motor function at short-term follow-up. Effects are expressed as the weighted mean difference (95% CI). The hashed line indicates clinically important difference.*

**

*f. P value for each comparison for motor function at short-term follow-up.*

| **Comparisons** | **P value** |
| --- | --- |
| BoNT vs Control | <0.0001 |
| HFrTMS vs Control | 0.0002 |
| LFrTMS vs Control | <0.0001 |
| cTBS vs Control | 0.72 |
| iTBS vs Control | 0.41 |
| atDCS vs Control | 0.0004 |
| ctDCS vs Control | 0.0055 |
| NMES vs Control | <0.0001 |
| TENS vs Control | 0.11 |
| rPMS vs Control | 0.23 |
| HFrTMS vs BoNT | 0.45 |
| LFrTMS vs BoNT | 0.60 |
| cTBS vs BoNT | 0.65 |
| iTBS vs BoNT | 0.17 |
| atDCS vs BoNT | 0.95 |
| ctDCS vs BoNT | 0.68 |
| NMES vs BoNT | 0.99 |
| TENS vs BoNT | 0.35 |
| rPMS vs BoNT | 0.44 |
| LFrTMS vs HFrTMS | 0.72 |
| cTBS vs HFrTMS | 0.77 |
| iTBS vs HFrTMS | 0.37 |
| atDCS vs HFrTMS | 0.53 |
| ctDCS vs HFrTMS | 0.88 |
| NMES vs HFrTMS | 0.54 |
| TENS vs HFrTMS | 0.67 |
| rPMS vs HFrTMS | 0.70 |
| cTBS vs LFrTMS | 0.72 |
| iTBS vs LFrTMS | 0.26 |
| atDCS vs LFrTMS | 0.68 |
| ctDCS vs LFrTMS | 0.93 |
| NMES vs LFrTMS | 0.70 |
| TENS vs LFrTMS | 0.50 |
| rPMS vs LFrTMS | 0.57 |
| iTBS vs cTBS | 0.95 |
| atDCS vs cTBS | 0.64 |
| ctDCS vs cTBS | 0.74 |
| NMES vs cTBS | 0.66 |
| TENS vs cTBS | 0.89 |
| rPMS vs cTBS | 0.91 |
| atDCS vs iTBS | 0.21 |
| ctDCS vs iTBS | 0.36 |
| NMES vs iTBS | 0.21 |
| TENS vs iTBS | 0.67 |
| rPMS vs iTBS | 0.67 |
| ctDCS vs atDCS | 0.67 |
| NMES vs atDCS | 0.95 |
| TENS vs atDCS | 0.39 |
| rPMS vs atDCS | 0.46 |
| NMES vs ctDCS | 0.72 |
| TENS vs ctDCS | 0.62 |
| rPMS vs ctDCS | 0.65 |
| TENS vs NMES | 0.37 |
| rPMS vs NMES | 0.47 |
| rPMS vs TENS | 0.98 |

*g. SUCRA value and mean rank*

*h. Ranking-cumulative probability plot*

*i. Results of pairwise meta-analysis. Effects are expressed as the weighted mean difference (95% CI).*

*j. P value for each pairwise comparison for motor function at short-term follow-up.*

| **Comparisons** | **P value** |
| --- | --- |
| atDCS-Control | 0.0030 |
| LFrTMS-Control | <0.0001 |
| iTBS-Control | 0.0070 |
| dtDCS-Control | 0.0070 |
| HFrTMS-Control | <0.0001 |
| LFrTMS-HFrTMS | 0.069 |
| ctDCS-Control | <0.0001 |
| ctDCS-atDCS | 0.90 |
| NMES-Control | 0.0030 |
| cTBS-Control | 0.76 |
| cTBS-LFrTMS | 0.72 |
| BoNT-Control | <0.0001 |
| NMES-dtDCS | 0.55 |
| LFrTMS-BoNT | <0.0001 |
| dtDCS-LFrTMS | 0.14 |
| TENS-Control | 0.24 |
| TENS-iTBS | 0.72 |

### 17.1.4 Motor function at mid-term follow-up

*a. Network plots of available comparisons*

**

*b. Results of design-by-treatment interaction model*

******

*c. Result of node sidesplit*

Note: A=Control; B=botulinum toxin; C=high-frequency transcranial magnetic stimulation; D=low-frequency transcranial magnetic stimulation; E=continuous theta-burst stimulation; F=cathodal transcranial direct current stimulation; G=neuromuscular electrical stimulation; H=transcutaneous electrical nerve stimulation.

*d. Results of loop-specific heterogeneity estimates*

*e. Summary forest plots for network meta-analysis results for motor function at mid-term follow-up. Effects are expressed as the weighted mean difference (95% CI). The hashed line indicates clinically important difference.*

**

*f. P value for each comparison for motor function at mid-term follow-up.*

| **Comparisons** | **P value** |
| --- | --- |
| BoNT vs Control | <0.0001 |
| HFrTMS vs Control | 0.20 |
| LFrTMS vs Control | 0.011 |
| cTBS vs Control | 0.71 |
| ctDCS vs Control | 0.02 |
| NMES vs Control | 0.54 |
| TENS vs Control | 0.99 |
| HFrTMS vs BoNT | 0.72 |
| LFrTMS vs BoNT | 0.69 |
| cTBS vs BoNT | 0.75 |
| ctDCS vs BoNT | 0.15 |
| NMES vs BoNT | 0.21 |
| TENS vs BoNT | 0.21 |
| LFrTMS vs HFrTMS | 0.86 |
| cTBS vs HFrTMS | 0.64 |
| ctDCS vs HFrTMS | 0.42 |
| NMES vs HFrTMS | 0.37 |
| TENS vs HFrTMS | 0.30 |
| cTBS vs LFrTMS | 0.65 |
| ctDCS vs LFrTMS | 0.23 |
| NMES vs LFrTMS | 0.20 |
| TENS vs LFrTMS | 0.18 |
| ctDCS vs cTBS | 0.25 |
| NMES vs cTBS | 0.88 |
| TENS vs cTBS | 0.74 |
| NMES vs ctDCS | 0.06 |

*g. SUCRA value and mean rank*

**

*g. Ranking-cumulative probability plot*

*h. Results of pairwise meta-analysis. Effects are expressed as the weighted mean difference (95% CI).*

*j. P value for each pairwise comparison for motor function at mid-term follow-up.*

| **Comparisons** | **P value** |
| --- | --- |
| LFrTMS-Control | 0.025 |
| BoNT-Control | <0.0001 |
| cTBS-Control | 0.66 |
| cTBS-LFrTMS | 0.59 |
| NMES-Control | 0.41 |
| ctDCS-Control | 0.0020 |
| TENS-Control | 0.53 |
| TENS-NMES | 0.68 |
| LFrTMS-BoNT | 0.47 |
| HFrTMS-Control | 0.08 |

### 17.1.5 Acceptability

*a. Network plots of available comparisons*

**

*b. Results of design-by-treatment interaction model*

******

*c. Result of node sidesplit*

Note: A=Control; B=botulinum toxin; C=high-frequency transcranial magnetic stimulation; D=low-frequency transcranial magnetic stimulation; E=continuous theta-burst stimulation; F= intermittent theta-burst stimulation; G=anodal transcranial direct current stimulation; H=cathodal transcranial direct current stimulation; I=dual transcranial direct current stimulation; J=neuromuscular electrical stimulation.

*****d. Results of loop-specific heterogeneity estimates*

*e. Summary forest plots for network meta-analysis results for acceptability. Effects are expressed as the risk ratio (95% CI). The hashed line indicates clinically important difference.*

**

*f. P value for each comparison for acceptability.*

| **Comparisons** | **P value** |
| --- | --- |
| BoNT vs Control | 0.35 |
| HFrTMS vs Control | 0.57 |
| LFrTMS vs Control | 0.42 |
| iTBS vs Control | 0.59 |
| atDCS vs Control | 0.98 |
| ctDCS vs Control | 0.46 |
| dtDCS vs Control | 0.68 |
| NMES vs Control | 0.49 |
| TENS vs Control | 0.85 |
| HFrTMS vs BoNT | 0.32 |
| LFrTMS vs BoNT | 0.23 |
| iTBS vs BoNT | 0.38 |
| atDCS vs BoNT | 0.78 |
| ctDCS vs BoNT | 0.66 |
| dtDCS vs BoNT | 0.49 |
| NMES vs BoNT | 0.26 |
| TENS vs BoNT | 0.54 |
| LFrTMS vs HFrTMS | 0.87 |
| iTBS vs HFrTMS | 0.88 |
| atDCS vs HFrTMS | 0.85 |
| ctDCS vs HFrTMS | 0.36 |
| dtDCS vs HFrTMS | 0.89 |
| NMES vs HFrTMS | 1.00 |
| TENS vs HFrTMS | 0.85 |
| iTBS vs LFrTMS | 0.94 |
| atDCS vs LFrTMS | 0.80 |
| ctDCS vs LFrTMS | 0.32 |
| dtDCS vs LFrTMS | 0.94 |
| NMES vs LFrTMS | 0.89 |
| TENS vs LFrTMS | 0.76 |
| atDCS vs iTBS | 0.79 |
| ctDCS vs iTBS | 0.36 |
| dtDCS vs iTBS | 0.99 |
| NMES vs iTBS | 0.87 |
| TENS vs iTBS | 0.77 |
| ctDCS vs atDCS | 0.53 |
| dtDCS vs atDCS | 0.80 |
| NMES vs atDCS | 0.85 |
| TENS vs atDCS | 0.95 |
| dtDCS vs ctDCS | 0.41 |
| NMES vs ctDCS | 0.35 |
| TENS vs ctDCS | 0.46 |
| NMES vs dtDCS | 0.88 |
| TENS vs dtDCS | 0.79 |
| TENS vs NMES | 0.80 |

*g. SUCRA value and mean rank*

*h. Ranking-cumulative probability plot*

*i. Results of pairwise meta-analysis. Effects are expressed as the risk ratio (95% CI).*

*j. P value for each pairwise comparison for acceptability.*

| **Comparisons** | **P value** |
| --- | --- |
| atDCS-Control | 1.00 |
| ctDCS-Control | 0.59 |
| ctDCS-atDCS | 0.51 |
| dtDCS-Control | 0.69 |
| TENS-Control | 0.60 |
| iTBS-Control | 0.61 |
| HFrTMS-Control | 0.30 |
| LFrTMS-Control | 0.73 |
| LFrTMS-HFrTMS | 0.62 |
| NMES-Control | 0.51 |
| TENS-NMES | 0.75 |
| BoNT-Control | 0.37 |

## 17.2 Exclusion of studies without additional co-intervention

### 17.2.1 Spasticity at short-term follow-up

*a. Network plots of available comparisons*

*b. Results of design-by-treatment interaction model*

*chi2( 24) = 15.02*

*Prob > chi2 = 0.9201*

*c. Result of node sidesplit*

Note: A=Control; B=botulinum toxin; C=high-frequency transcranial magnetic stimulation; D=low-frequency transcranial magnetic stimulation; E=intermittent theta-burst stimulation; F=continuous theta-burst stimulation; G=anodal transcranial direct current stimulation; H=cathodal transcranial direct current stimulation; I=dual transcranial direct current stimulation; J=neuromuscular electrical stimulation; K=transcutaneous electrical nerve stimulation; L=repetitive peripheral magnetic stimulation.

*d. Results of loop-specific heterogeneity estimates*

*e. Summary forest plots for network meta-analysis results for spasticity at short-term follow-up. Effects are expressed as the weighted mean difference (95% CI). The hashed line indicates clinically important difference.*

**

*f. P value for each comparison for spasticity at short-term follow-up.*

| **Comparisons** | **P value** |
| --- | --- |
| BoNT vs Control | <0.0001 |
| HFrTMS vs Control | 0.0026 |
| LFrTMS vs Control | <0.0001 |
| cTBS vs Control | 0.49 |
| iTBS vs Control | 0.13 |
| atDCS vs Control | 0.0008 |
| ctDCS vs Control | 0.007 |
| dtDCS vs Control | 0.0073 |
| NMES vs Control | <0.0001 |
| TENS vs Control | 0.0036 |
| rPMS vs Control | 0.39 |
| HFrTMS vs BoNT | <0.0001 |
| LFrTMS vs BoNT | <0.0001 |
| cTBS vs BoNT | 0.17 |
| iTBS vs BoNT | 0.0031 |
| atDCS vs BoNT | 0.013 |
| ctDCS vs BoNT | 0.03 |
| dtDCS vs BoNT | 0.036 |
| NMES vs BoNT | <0.0001 |
| TENS vs BoNT | 0.017 |
| rPMS vs BoNT | 0.0043 |
| LFrTMS vs HFrTMS | 0.53 |
| cTBS vs HFrTMS | 0.92 |
| iTBS vs HFrTMS | 0.85 |
| atDCS vs HFrTMS | 0.39 |
| ctDCS vs HFrTMS | 0.49 |
| dtDCS vs HFrTMS | 0.48 |
| NMES vs HFrTMS | 0.21 |
| TENS vs HFrTMS | 0.47 |
| rPMS vs HFrTMS | 0.60 |
| cTBS vs LFrTMS | 0.77 |
| iTBS vs LFrTMS | 0.56 |
| atDCS vs LFrTMS | 0.64 |
| ctDCS vs LFrTMS | 0.73 |
| dtDCS vs LFrTMS | 0.71 |
| NMES vs LFrTMS | 0.40 |
| TENS vs LFrTMS | 0.72 |
| rPMS vs LFrTMS | 0.39 |
| iTBS vs cTBS | 1.00 |
| atDCS vs cTBS | 0.66 |
| ctDCS vs cTBS | 0.68 |
| dtDCS vs cTBS | 0.67 |
| NMES vs cTBS | 0.62 |
| TENS vs cTBS | 0.68 |
| rPMS vs cTBS | 0.85 |
| atDCS vs iTBS | 0.41 |
| ctDCS vs iTBS | 0.47 |
| dtDCS vs iTBS | 0.46 |
| NMES vs iTBS | 0.31 |
| TENS vs iTBS | 0.46 |
| rPMS vs iTBS | 0.74 |
| ctDCS vs atDCS | 0.95 |
| dtDCS vs atDCS | 0.98 |
| NMES vs atDCS | 0.94 |
| TENS vs atDCS | 0.95 |
| rPMS vs atDCS | 0.29 |
| dtDCS vs ctDCS | 0.98 |
| NMES vs ctDCS | 0.90 |
| TENS vs ctDCS | 1.00 |
| rPMS vs ctDCS | 0.34 |
| NMES vs dtDCS | 0.93 |
| TENS vs dtDCS | 0.97 |
| rPMS vs dtDCS | 0.33 |
| TENS vs NMES | 0.88 |
| rPMS vs NMES | 0.22 |
| rPMS vs TENS | 0.33 |

*h. SUCRA value and mean rank*

*i. Ranking-cumulative probability plot.*

*h. Results of pairwise meta-analysis. Effects are expressed as the weighted mean difference (95% CI).*

*j. P value for each pairwise comparison for spasticity at short-term follow-up.*

| **Comparisons** | **P value** |
| --- | --- |
| atDCS-Control | 0.0010 |
| LFrTMS-Control | <0.0001 |
| NMES-Control | <0.0001 |
| iTBS-Control | 0.201 |
| TENS-Control | 0.001 |
| HFrTMS-Control | 0.001 |
| LFrTMS-HFrTMS | 0.14 |
| BoNT-Control | <0.0001 |
| dtDCS-ctDCS | 0.60 |
| NMES-BoNT | 0.94 |
| ctDCS-Control | 0.0090 |
| ctDCS-atDCS | 0.54 |
| cTBS-Control | 0.50 |
| cTBS-LFrTMS | 0.75 |
| TENS-BoNT | 0.0080 |
| dtDCS-Control | <0.0001 |
| dtDCS-atDCS | 0.83 |
| TENS-NMES | 0.60 |
| LFrTMS-BoNT | <0.0001 |
| iTBS-HFrTMS | 1.00 |
| rPMS-Control | 0.031 |
| rPMS-iTBS | 0.74 |
| NMES-LFrTMS | 0.43 |

### 17.2.2 Spasticity at mid-term follow-up

*a. Network plots of available comparisons*

**

*b. Results of design-by-treatment interaction model*


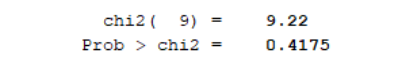


*c. Result of node sidesplit*

Note: A=Control; B=botulinum toxin; C=high-frequency transcranial magnetic stimulation; D=low-frequency transcranial magnetic stimulation; E=continuous theta-burst stimulation; F=cathodal transcranial direct current stimulation; G=dual transcranial direct current stimulation, H=neuromuscular electrical stimulation; K=transcutaneous electrical nerve stimulation; i=repetitive peripheral magnetic stimulation.

*d. Results of loop-specific heterogeneity estimates*

**

*e. Summary forest plots for network meta-analysis results for spasticity at mid-term follow-up. Effects are expressed as the weighted mean difference (95% CI). The hashed line indicates clinically important difference.*

**

*f. P value for each comparison for spasticity at mid-term follow-up.*

| **Comparisons** | **P value** |
| --- | --- |
| BoNT vs Control | <0.0001 |
| HFrTMS vs Control | 0.34 |
| LFrTMS vs Control | 0.53 |
| cTBS vs Control | 0.55 |
| ctDCS vs Control | 0.0002 |
| dtDCS vs Control | 0.051 |
| NMES vs Control | 0.10 |
| TENS vs Control | 0.45 |
| HFrTMS vs BoNT | 0.01 |
| LFrTMS vs BoNT | <0.0001 |
| cTBS vs BoNT | 0.23 |
| ctDCS vs BoNT | 0.025 |
| dtDCS vs BoNT | 0.32 |
| NMES vs BoNT | 0.054 |
| TENS vs BoNT | 0.054 |
| LFrTMS vs HFrTMS | 0.67 |
| cTBS vs HFrTMS | 0.91 |
| ctDCS vs HFrTMS | 0.00 |
| dtDCS vs HFrTMS | 0.10 |
| NMES vs HFrTMS | 0.63 |
| TENS vs HFrTMS | 0.97 |
| cTBS vs LFrTMS | 0.70 |
| ctDCS vs LFrTMS | 0.00 |
| dtDCS vs LFrTMS | 0.07 |
| NMES vs LFrTMS | 0.33 |
| TENS vs LFrTMS | 0.71 |
| ctDCS vs cTBS | 0.01 |
| dtDCS vs cTBS | 0.15 |
| NMES vs cTBS | 0.84 |
| TENS vs cTBS | 0.94 |
| dtDCS vs ctDCS | 0.55 |
| NMES vs ctDCS | 0.00 |
| TENS vs ctDCS | 0.00 |
| NMES vs dtDCS | 0.14 |
| TENS vs dtDCS | 0.11 |
| TENS vs NMES | 0.67 |

*g. SUCRA value and mean rank*


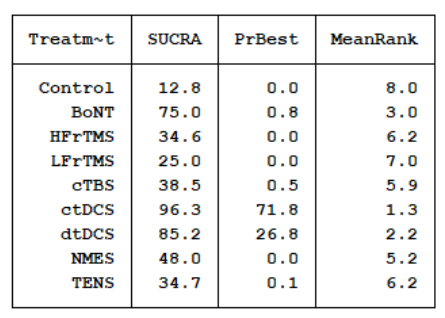


*h. Ranking-cumulative probability plot*

**

*i. Results of pairwise meta-analysis. Effects are expressed as the weighted mean difference (95% CI).*

**

*j. P value for each pairwise comparison for spasticity at mid-term follow-up.*

| **Comparisons** | **P value** |
| --- | --- |
| LFrTMS-Control | <0.0001 |
| HFrTMS-Control | 0.034 |
| BoNT-Control | <0.0001 |
| dtDCS-ctDCS | 0.46 |
| NMES-Control | 0.014 |
| NMES-BoNT | 0.74 |
| cTBS-Control | 0.26 |
| cTBS-LFrTMS | 0.80 |
| TENS-BoNT | 0.12 |
| ctDCS-Control | <0.0001 |
| TENS-Control | 0.92 |
| TENS-NMES | 0.90 |
| LFrTMS-BoNT | <0.0001 |
| LFrTMS-HFrTMS | 0.69 |

### 17.2.3 Motor function at short-term follow-up

*a. Network plots of available comparisons*

*b. Results of design-by-treatment interaction model.*

***chi2( 16) = 22.18***

***Prob > chi2 = 0.1375***

*c. Result of node sidesplit*

Note: A=Control; B=botulinum toxin; C=high-frequency transcranial magnetic stimulation; D=low-frequency transcranial magnetic stimulation; E=continuous theta-burst stimulation; F= intermittent theta-burst stimulation; G=anodal transcranial direct current stimulation; H=cathodal transcranial direct current stimulation; I=neuromuscular electrical stimulation; J=transcutaneous electrical nerve stimulation; K=repetitive peripheral magnetic stimulation.

*d. Results of loop-specific heterogeneity estimates*

*e. Summary forest plots for network meta-analysis results for motor function at short-term follow-up. Effects are expressed as the weighted mean difference (95% CI). The hashed line indicates clinically important difference.*

**

*f. P value for each comparison for motor function at short-term follow-up.*

| **Comparisons** | **P value** |
| --- | --- |
| BoNT vs Control | <0.0001 |
| HFrTMS vs Control | <0.0001 |
| LFrTMS vs Control | <0.0001 |
| cTBS vs Control | 0.71 |
| iTBS vs Control | 0.065 |
| atDCS vs Control | <0.0001 |
| ctDCS vs Control | 0.0021 |
| NMES vs Control | <0.0001 |
| TENS vs Control | 0.083 |
| rPMS vs Control | 0.14 |
| HFrTMS vs BoNT | 0.27 |
| LFrTMS vs BoNT | 0.48 |
| cTBS vs BoNT | 0.65 |
| iTBS vs BoNT | 0.29 |
| atDCS vs BoNT | 0.62 |
| ctDCS vs BoNT | 0.72 |
| NMES vs BoNT | 0.81 |
| TENS vs BoNT | 0.29 |
| rPMS vs BoNT | 0.50 |
| LFrTMS vs HFrTMS | 0.58 |
| cTBS vs HFrTMS | 0.78 |
| iTBS vs HFrTMS | 0.68 |
| atDCS vs HFrTMS | 0.25 |
| ctDCS vs HFrTMS | 0.74 |
| NMES vs HFrTMS | 0.45 |
| TENS vs HFrTMS | 0.67 |
| rPMS vs HFrTMS | 0.84 |
| cTBS vs LFrTMS | 0.71 |
| iTBS vs LFrTMS | 0.46 |
| atDCS vs LFrTMS | 0.38 |
| ctDCS vs LFrTMS | 1.00 |
| NMES vs LFrTMS | 0.73 |
| TENS vs LFrTMS | 0.46 |
| rPMS vs LFrTMS | 0.66 |
| iTBS vs cTBS | 0.89 |
| atDCS vs cTBS | 0.57 |
| ctDCS vs cTBS | 0.72 |
| NMES vs cTBS | 0.68 |
| TENS vs cTBS | 0.89 |
| rPMS vs cTBS | 0.86 |
| atDCS vs iTBS | 0.23 |
| ctDCS vs iTBS | 0.55 |
| NMES vs iTBS | 0.38 |
| TENS vs iTBS | 0.98 |
| rPMS vs iTBS | 0.91 |
| ctDCS vs atDCS | 0.48 |
| NMES vs atDCS | 0.54 |
| TENS vs atDCS | 0.23 |
| rPMS vs atDCS | 0.38 |
| NMES vs ctDCS | 0.84 |
| TENS vs ctDCS | 0.54 |
| rPMS vs ctDCS | 0.70 |
| TENS vs NMES | 0.35 |
| rPMS vs NMES | 0.57 |
| rPMS vs TENS | 0.91 |

*g. SUCRA value and mean rank*

*h. Ranking-cumulative probability plot*

*i. Results of pairwise meta-analysis. Effects are expressed as the weighted mean difference (95% CI).*

*j. P value for each pairwise comparison for motor function at short-term follow-up.*

| **Comparisons** | **P value** |
| --- | --- |
| atDCS-Control | 0.0010 |
| LFrTMS-Control | <0.0001 |
| iTBS-Control | 0.016 |
| dtDCS-Control | <0.0001 |
| HFrTMS-Control | <0.0001 |
| LFrTMS-HFrTMS | 0.052 |
| ctDCS-Control | <0.0001 |
| ctDCS-atDCS | 0.90 |
| NMES-Control | 0.0030 |
| cTBS-Control | 0.76 |
| cTBS-LFrTMS | 0.72 |
| BoNT-Control | <0.0001 |
| NMES-dtDCS | 0.55 |
| LFrTMS-BoNT | 0.84 |
| dtDCS-LFrTMS | 0.14 |
| TENS-Control | 0.24 |
| TENS-iTBS | 0.72 |
| iTBS-HFrTMS | 0.79 |

### 17.2.4 Motor function at mid-term follow-up

*a. Network plots of available comparisons*

**

*b. Results of design-by-treatment interaction model*

***chi2( 6) = 0.93***

***Prob > chi2 = 0.9883***

*c. Result of node sidesplit*

Note: A=Control; B=botulinum toxin; C=high-frequency transcranial magnetic stimulation; D=low-frequency transcranial magnetic stimulation; E=continuous theta-burst stimulation; F=cathodal transcranial direct current stimulation; G=neuromuscular electrical stimulation; H=transcutaneous electrical nerve stimulation.

*d. Results of loop-specific heterogeneity estimates*

**

*e. Summary forest plots for network meta-analysis results for motor function at mid-term follow-up. Effects are expressed as the weighted mean difference (95% CI). The hashed line indicates clinically important difference.*

**

*f. P value for each comparison for motor function at mid-term follow-up.*

| **Comparisons** | **P value** |
| --- | --- |
| BoNT vs Control | <0.0001 |
| HFrTMS vs Control | 0.024 |
| LFrTMS vs Control | 0.0073 |
| cTBS vs Control | 0.85 |
| ctDCS vs Control | 0.012 |
| NMES vs Control | 0.46 |
| TENS vs Control | 0.97 |
| HFrTMS vs BoNT | 0.98 |
| LFrTMS vs BoNT | 0.23 |
| cTBS vs BoNT | 0.56 |
| ctDCS vs BoNT | 0.13 |
| NMES vs BoNT | 0.11 |
| TENS vs BoNT | 0.12 |
| LFrTMS vs HFrTMS | 0.53 |
| cTBS vs HFrTMS | 0.59 |
| ctDCS vs HFrTMS | 0.16 |
| NMES vs HFrTMS | 0.25 |
| TENS vs HFrTMS | 0.20 |
| cTBS vs LFrTMS | 0.74 |
| ctDCS vs LFrTMS | 0.07 |
| NMES vs LFrTMS | 0.43 |
| TENS vs LFrTMS | 0.32 |
| ctDCS vs cTBS | 0.17 |
| NMES vs cTBS | 0.97 |
| TENS vs cTBS | 0.87 |
| NMES vs ctDCS | 0.04 |
| TENS vs ctDCS | 0.033 |
| TENS vs NMES | 0.64 |

*g. SUCRA value and mean rank*

**

*h. Ranking-cumulative probability plot*

**

*i. Results of pairwise meta-analysis. Effects are expressed as the weighted mean difference (95% CI).*

*j. P value for each pairwise comparison for motor function at mid-term follow-up.*

| **Comparisons** | **P value** |
| --- | --- |
| LFrTMS-Control | <0.0001 |
| HFrTMS-Control | <0.0001 |
| BoNT-Control | <0.0001 |
| cTBS-Control | 0.59 |
| cTBS-LFrTMS | 0.97 |
| NMES-Control | 0.52 |
| ctDCS-Control | 0.0020 |
| TENS-Control | 0.54 |
| TENS-NMES | <0.0001 |
| LFrTMS-BoNT | 0.064 |

### 17.2.5 Acceptability

*a. Network plots of available comparisons*

*b. Results of design-by-treatment interaction model.*

*c. Result of node sidesplit*

Note: A=Control; B=botulinum toxin; C=high-frequency transcranial magnetic stimulation; D=low-frequency transcranial magnetic stimulation; E=intermittent theta-burst stimulation; F=anodal transcranial direct current stimulation; G=cathodal transcranial direct current stimulation; H=dual transcranial direct current stimulation; I=neuromuscular electrical stimulation; J=transcutaneous electrical nerve stimulation.

*d. Results of loop-specific heterogeneity estimates*

*e. Summary forest plots for network meta-analysis results for acceptability. Effects are expressed as the risk ratio (95% CI). The hashed line indicates clinically important difference.*

*f. P value for each comparison for motor function at acceptability.*

| **Comparisons** | **P value** |
| --- | --- |
| BoNT vs Control | 0.40 |
| HFrTMS vs Control | 0.40 |
| LFrTMS vs Control | 0.35 |
| iTBS vs Control | 0.46 |
| atDCS vs Control | 0.72 |
| ctDCS vs Control | 0.86 |
| dtDCS vs Control | 0.56 |
| NMES vs Control | 0.33 |
| TENS vs Control | 0.54 |
| HFrTMS vs BoNT | 0.40 |
| LFrTMS vs BoNT | 0.35 |
| iTBS vs BoNT | 0.46 |
| atDCS vs BoNT | 0.71 |
| ctDCS vs BoNT | 0.86 |
| dtDCS vs BoNT | 0.55 |
| NMES vs BoNT | 0.33 |
| TENS vs BoNT | 0.54 |
| LFrTMS vs HFrTMS | 0.47 |
| iTBS vs HFrTMS | 0.61 |
| atDCS vs HFrTMS | 0.79 |
| ctDCS vs HFrTMS | 0.90 |
| dtDCS vs HFrTMS | 0.67 |
| NMES vs HFrTMS | 0.54 |
| TENS vs HFrTMS | 0.68 |
| iTBS vs LFrTMS | 0.62 |
| atDCS vs LFrTMS | 0.80 |
| ctDCS vs LFrTMS | 0.90 |
| dtDCS vs LFrTMS | 0.69 |
| NMES vs LFrTMS | 0.56 |
| TENS vs LFrTMS | 0.69 |
| atDCS vs iTBS | 0.84 |
| ctDCS vs iTBS | 0.92 |
| dtDCS vs iTBS | 0.74 |
| NMES vs iTBS | 0.67 |
| TENS vs iTBS | 0.76 |
| ctDCS vs atDCS | 0.89 |
| dtDCS vs atDCS | 0.72 |
| NMES vs atDCS | 0.67 |
| TENS vs atDCS | 0.76 |
| dtDCS vs ctDCS | 0.42 |
| NMES vs ctDCS | 0.34 |
| TENS vs ctDCS | 0.50 |
| NMES vs dtDCS | 0.74 |
| TENS vs dtDCS | 0.81 |
| TENS vs NMES | 0.63 |

*g. SUCRA value and mean rank*

*h. Ranking-cumulative probability plot*

*i. Results of pairwise meta-analysis*

*j. P value for each pairwise comparison for acceptability.*

| **Comparisons** | **P value** |
| --- | --- |
| atDCS-Control | 1.00 |
| ctDCS-Control | 0.59 |
| ctDCS-atDCS | 0.51 |
| dtDCS-Control | 0.69 |
| TENS-Control | 0.95 |
| iTBS-Control | 0.61 |
| HFrTMS-Control | 0.40 |
| LFrTMS-Control | 0.72 |
| LFrTMS-HFrTMS | 0.62 |
| NMES-Control | 0.50 |
| TENS-NMES | 0.75 |
| BoNT-Control | 0.73 |

## 17.3 Exclusion of studies with BoNT vs. Control comparison at short-term follow-up

### 17.3.1 Spasticity at short-term follow-up

*a. Network plots of available comparisons*

*b. Results of design-by-treatment interaction model*

***chi2 (18) = 13.93***

***Prob > chi2 = 0.7336***

*c. Result of node sidesplit*

Note: A=Control; B=botulinum toxin; C=high-frequency transcranial magnetic stimulation; D=low-frequency transcranial magnetic stimulation; E=intermittent theta-burst stimulation; F=continuous theta-burst stimulation; G=anodal transcranial direct current stimulation; H=cathodal transcranial direct current stimulation; I=dual transcranial direct current stimulation; J=neuromuscular electrical stimulation; K=transcutaneous electrical nerve stimulation; L=repetitive peripheral magnetic stimulation.

*d. Results of loop-specific heterogeneity estimates*

*e. Summary forest plots for network meta-analysis results for spasticity at short-term follow-up. Effects are expressed as the weighted mean difference (95% CI). The hashed line indicates clinically important difference.*

*f. P value for each comparison for spasticity at short-term follow-up.*

| **Comparisons** | **P value** |
| --- | --- |
| BoNT vs Control | 0.0001 |
| HFrTMS vs Control | <0.0001 |
| LFrTMS vs Control | <0.0001 |
| cTBS vs Control | 0.37 |
| iTBS vs Control | 0.06 |
| atDCS vs Control | <0.0001 |
| ctDCS vs Control | 0.0011 |
| dtDCS vs Control | 0.0006 |
| NMES vs Control | <0.0001 |
| TENS vs Control | 0.0002 |
| rPMS vs Control | 0.19 |
| HFrTMS vs BoNT | 0.0061 |
| LFrTMS vs BoNT | 0.013 |
| cTBS vs BoNT | 0.052 |
| iTBS vs BoNT | 0.008 |
| atDCS vs BoNT | 0.029 |
| ctDCS vs BoNT | 0.026 |
| dtDCS vs BoNT | 0.031 |
| NMES vs BoNT | 0.02 |
| TENS vs BoNT | 0.02 |
| rPMS vs BoNT | 0.0092 |
| LFrTMS vs HFrTMS | 0.25 |
| cTBS vs HFrTMS | 0.97 |
| iTBS vs HFrTMS | 0.82 |
| atDCS vs HFrTMS | 0.25 |
| ctDCS vs HFrTMS | 0.44 |
| dtDCS vs HFrTMS | 0.36 |
| NMES vs HFrTMS | 0.16 |
| TENS vs HFrTMS | 0.28 |
| rPMS vs HFrTMS | 0.73 |
| cTBS vs LFrTMS | 0.72 |
| iTBS vs LFrTMS | 0.37 |
| atDCS vs LFrTMS | 0.69 |
| ctDCS vs LFrTMS | 0.91 |
| dtDCS vs LFrTMS | 0.80 |
| NMES vs LFrTMS | 0.67 |
| TENS vs LFrTMS | 0.69 |
| rPMS vs LFrTMS | 0.37 |
| iTBS vs cTBS | 0.95 |
| atDCS vs cTBS | 0.63 |
| ctDCS vs cTBS | 0.71 |
| dtDCS vs cTBS | 0.66 |
| NMES vs cTBS | 0.65 |
| TENS vs cTBS | 0.63 |
| rPMS vs cTBS | 0.89 |
| atDCS vs iTBS | 0.30 |
| ctDCS vs iTBS | 0.43 |
| dtDCS vs iTBS | 0.37 |
| NMES vs iTBS | 0.27 |
| TENS vs iTBS | 0.31 |
| rPMS vs iTBS | 0.88 |
| ctDCS vs atDCS | 0.83 |
| dtDCS vs atDCS | 0.94 |
| NMES vs atDCS | 0.90 |
| TENS vs atDCS | 0.97 |
| rPMS vs atDCS | 0.30 |
| dtDCS vs ctDCS | 0.91 |
| NMES vs ctDCS | 0.90 |
| TENS vs ctDCS | 0.83 |
| rPMS vs ctDCS | 0.41 |
| NMES vs dtDCS | 0.98 |
| TENS vs dtDCS | 0.92 |
| rPMS vs dtDCS | 0.35 |
| TENS vs NMES | 0.87 |
| rPMS vs NMES | 0.29 |
| rPMS vs TENS | 0.31 |

*g. SUCRA value and mean rank*

*h. Ranking-cumulative probability plot*

*i. Results of pairwise meta-analysis. Effects are expressed as the weighted mean difference (95% CI).*

*j. P value for each pairwise comparison for spasticity at short-term follow-up.*

| **Comparisons** | **P value** |
| --- | --- |
| atDCS-Control | 0.0030 |
| LFrTMS-Control | <0.0001 |
| NMES-Control | <0.0001 |
| iTBS-Control | 0.20 |
| TENS-Control | 0.0010 |
| HFrTMS-Control | <0.0001 |
| LFrTMS-HFrTMS | 0.13 |
| dtDCS-ctDCS | 0.60 |
| NMES-BoNT | 0.94 |
| ctDCS-Control | 0.053 |
| ctDCS-atDCS | 0.54 |
| cTBS-Control | 0.50 |
| cTBS-LFrTMS | 0.75 |
| TENS-BoNT | 0.0080 |
| dtDCS-Control | <0.0001 |
| dtDCS-atDCS | 0.83 |
| TENS-NMES | 0.60 |
| LFrTMS-BoNT | <0.0001 |
| iTBS-HFrTMS | 1.00 |
| rPMS-Control | 0.024 |
| rPMS-iTBS | 0.74 |
| NMES-LFrTMS | 0.43 |

### 17.3.2 Motor function at short-term follow-up

*a. Network plots of available comparisons*

*b. Results of design-by-treatment interaction model*

***chi2( 14) = 21.49***

***Prob > chi2 = 0.0898***

*c. Result of node sidesplit*

Note: A=Control; B=high-frequency transcranial magnetic stimulation; C=low-frequency transcranial magnetic stimulation; D=continuous theta-burst stimulation; E=intermittent theta-burst stimulation; F=anodal transcranial direct current stimulation; G=cathodal transcranial direct current stimulation; H=neuromuscular electrical stimulation; I=transcutaneous electrical nerve stimulation; J=repetitive peripheral magnetic stimulation.

*d. Results of loop-specific heterogeneity estimates*

*e. Summary forest plots for network meta-analysis results for motor function at short-term follow-up. Effects are expressed as the weighted mean difference (95% CI). The hashed line indicates clinically important difference.*

*f. P value for each comparison for motor function at short-term follow-up.*

| **Comparisons** | **P value** |
| --- | --- |
| HFrTMS vs Control | <0.0001 |
| LFrTMS vs Control | <0.0001 |
| cTBS vs Control | 0.71 |
| iTBS vs Control | 0.043 |
| atDCS vs Control | <0.0001 |
| ctDCS vs Control | 0.0008 |
| NMES vs Control | <0.0001 |
| TENS vs Control | 0.055 |
| rPMS vs Control | 0.12 |
| LFrTMS vs HFrTMS | 0.50 |
| cTBS vs HFrTMS | 0.79 |
| iTBS vs HFrTMS | 0.68 |
| atDCS vs HFrTMS | 0.18 |
| ctDCS vs HFrTMS | 0.69 |
| NMES vs HFrTMS | 0.40 |
| TENS vs HFrTMS | 0.66 |
| rPMS vs HFrTMS | 0.82 |
| cTBS vs LFrTMS | 0.71 |
| iTBS vs LFrTMS | 0.42 |
| atDCS vs LFrTMS | 0.32 |
| ctDCS vs LFrTMS | 0.99 |
| NMES vs LFrTMS | 0.76 |
| TENS vs LFrTMS | 0.41 |
| rPMS vs LFrTMS | 0.60 |
| iTBS vs cTBS | 0.88 |
| atDCS vs cTBS | 0.56 |
| ctDCS vs cTBS | 0.72 |
| NMES vs cTBS | 0.68 |
| TENS vs cTBS | 0.89 |
| rPMS vs cTBS | 0.86 |
| atDCS vs iTBS | 0.18 |
| ctDCS vs iTBS | 0.52 |
| NMES vs iTBS | 0.35 |
| TENS vs iTBS | 0.97 |
| rPMS vs iTBS | 0.94 |
| ctDCS vs atDCS | 0.42 |
| NMES vs atDCS | 0.45 |
| TENS vs atDCS | 0.17 |
| rPMS vs atDCS | 0.31 |
| NMES vs ctDCS | 0.85 |
| TENS vs ctDCS | 0.50 |
| rPMS vs ctDCS | 0.65 |
| TENS vs NMES | 0.31 |
| rPMS vs NMES | 0.53 |
| rPMS vs TENS | 0.92 |

*g. SUCRA value and mean rank*

*h. Ranking-cumulative probability plot*

### 17.3.3 Acceptability

*a. Network plots of available comparisons*

*b. Results of design-by-treatment interaction model.*

*c. Result of node sidesplit*

Note: A=Control; B=high-frequency transcranial magnetic stimulation; C=low-frequency transcranial magnetic stimulation; D=intermittent theta-burst stimulation; E=anodal transcranial direct current stimulation; F=cathodal transcranial direct current stimulation; G=dual transcranial direct current stimulation; H=neuromuscular electrical stimulation; I=transcutaneous electrical nerve stimulation.

*d. Results of loop-specific heterogeneity estimates*

*e. Summary forest plots for network meta-analysis results for acceptability. Effects are expressed as the risk ratio (95% CI). The hashed line indicates clinically important difference.*

*f. P value for each comparison for spasticity at short-term follow-up.*

| **Comparisons** | **P value** |
| --- | --- |
| HFrTMS vs Control | 0.77 |
| LFrTMS vs Control | 0.78 |
| iTBS vs Control | 0.81 |
| atDCS vs Control | 0.72 |
| ctDCS vs Control | 0.54 |
| dtDCS vs Control | 0.82 |
| NMES vs Control | 0.78 |
| TENS vs Control | 0.70 |
| LFrTMS vs HFrTMS | 0.72 |
| iTBS vs HFrTMS | 0.76 |
| atDCS vs HFrTMS | 0.66 |
| ctDCS vs HFrTMS | 0.50 |
| dtDCS vs HFrTMS | 0.76 |
| NMES vs HFrTMS | 0.72 |
| TENS vs HFrTMS | 0.65 |
| iTBS vs LFrTMS | 0.75 |
| atDCS vs LFrTMS | 0.65 |
| ctDCS vs LFrTMS | 0.49 |
| dtDCS vs LFrTMS | 0.75 |
| NMES vs LFrTMS | 0.71 |
| TENS vs LFrTMS | 0.64 |
| atDCS vs iTBS | 0.62 |
| ctDCS vs iTBS | 0.47 |
| dtDCS vs iTBS | 0.72 |
| NMES vs iTBS | 0.68 |
| TENS vs iTBS | 0.61 |
| ctDCS vs atDCS | 0.53 |
| dtDCS vs atDCS | 0.81 |
| NMES vs atDCS | 0.77 |
| TENS vs atDCS | 0.69 |
| dtDCS vs ctDCS | 0.96 |
| NMES vs ctDCS | 0.94 |
| TENS vs ctDCS | 0.89 |
| NMES vs dtDCS | 0.67 |
| TENS vs dtDCS | 0.60 |
| TENS vs NMES | 0.64 |

*g. SUCRA value and mean rank*

*h. Ranking-cumulative probability plot*

# Appendix 18. Meta-regression analysis.

## 18.1 Univariate meta-analysis analysis based on limb measured

### 18.1.1 Spasticity at short-term follow-up

**A.** BoNT-Control

**B.** HFrTMS-Control

**C.** LFrTMS-Control

**D.** iTBS-Control

**E.** atDCS-Control

**F.** NMES-Control

**G.** TENS-Control

**H.** LFrTMS-HFrTMS

### 18.1.2 Spasticity at mid-term follow-up

**A.** BoNT-Control

**B.** HFrTMS-Control

**C.** LFrTMS-Control

### 18.1.3 Motor function at short-term follow-up

**A.** BoNT-Control

**B.** HFrTMS-Control

**C.** LFrTMS-Control

**D.** iTBS-Control

**E.** atDCS-Control

**F.** NMES-Control

**G.** HFrTMS-LFrTMS

### 18.1.4 Motor function at mid-term follow-up

**A.** BoNT-Control

**B.** LFrTMS-Control

**C.** LFrTMS-BoNT

**D.** HFrTMS-Control

### 18.1.5 Acceptability

**A.** BoNT-Control

**B.** LFrTMS-Control

**C.** NMES-Control

**D.** TENS-Control

## 18.2 Univariate meta-analysis analysis based on with or without cointervention

### 18.2.1 Spasticity at short-term follow-up

**A.** BoNT-Control

**B.** LFrTMS-Control

**C.** NMES-Control

### 18.2.2 Spasticity at mid-term follow-up

**A.** BoNT-Control

**B.** HFrTMS-Control

### 18.2.3 Motor function at short-term follow-up

**A.** BoNT-Control

**B.** LFrTMS-Control

### 18.2.4 Motor function at mid-term follow-up

**A.** BoNT-Control

### 18.2.5 Acceptability

**A.** BoNT-Control

**B.** LFrTMS-Control

## 18.3 Univariate meta-analysis analysis based on stroke stage

### 18.3.1 Spasticity at short-term follow-up

**A.** BoNT-Control

**B.** HFrTMS-Control

**C.** LFrTMS-Control

**D.** iTBS-Control

**E.** atDCS-Control

**F.** NMES-Control

**G.** TENS-Control

**H.** rPMS-Control

**I.** LFrTMS-HFrTMS

### 18.3.2 Spasticity at mid-term follow-up

**A.** BoNT-Control

**B.** LFrTMS-Control

**C.** NMES-Control

### 18.3.3 Motor function at short-term follow-up

**A.** BoNT-Control

**B.** HFrTMS-Control

**C.** LFrTMS-Control

**D.** iTBS-Control

**E.** atDCS-Control

**F.** ctDCS-Control

**G.** NMES-Control

**H.** TENS-Control

**I.** rPMS-Control

**J.** LFrTMS-HFrTMS

### 18.3.4 Motor function at mid-term follow-up

**A.** BoNT-Control

**B.** HFrTMS-Control

**C.** LFrTMS-Control

**D.** NMES-Control

**E.** LFrTMS-BoNT

### 18.3.5 Acceptability

**A.** BoNT-Control

**B.** LFrTMS-Control

**C.** HFrTMS-Control

**D.** iTBS-Control

**E.** NMES-Control

**F.** TENS-Control

## 18.4 Multivariate meta-analysis analysis based on limb measured, with or without cointervention, and stroke stage

### 18.4.1 Spasticity at short-term follow-up

**A.** BoNT-Control

**B.** HFrTMS-Control

**C.** LFrTMS-Control

**D.** atDCS-Control

**E.** iTBS-Control

**F.** NMES-Control

**G.** TENS-Control

**H.** LFrTMS-HFrTMS

### 18.4.2 Spasticity at mid-term follow-up

**A.** LFrTMS-Control

### 18.4.3 Motor function at short-term follow-up

**A.** BoNT-Control

**B.** HFrTMS-Control

**B.** LFrTMS-Control

**C.** iTBS-Control

**D.** atDCS-Control

**E.** NMES-Control

### 18.4.4 Motor function at mid-term follow-up

**A.** BoNT-Control

**B.** LFrTMS-Control

### 18.4.5 Acceptability

**A.** BoNT-Control

**B.** NMES-Control

# Appendix 19. Publication bias.

## 19.1 Spasticity at short-term follow-up

**Abbreviations:** BoNT=botulinum toxin; LFrTMS=low-frequency transcranial magnetic stimulation; iTBS= intermittent theta-burst stimulation; HFrTMS = high-frequency transcranial magnetic stimulation; cTBS=continuous theta-burst stimulation; atDCS=anodal transcranial direct current stimulation; ctDCS=cathodal transcranial direct current stimulation; dtDCS=dual transcranial direct current stimulation; NMES=neuromuscular electrical stimulation; TENS=transcutaneous electrical nerve stimulation; rPMS= repetitive peripheral magnetic stimulation.

## 19.2 Spasticity at mid-term follow-up

**Abbreviations:** BoNT=botulinum toxin; LFrTMS=low-frequency transcranial magnetic stimulation; iTBS= intermittent theta-burst stimulation; HFrTMS = high-frequency transcranial magnetic stimulation; cTBS=continuous theta-burst stimulation; ctDCS=cathodal transcranial direct current stimulation; dtDCS=dual transcranial direct current stimulation; NMES=neuromuscular electrical stimulation; TENS=transcutaneous electrical nerve stimulation.

## 19.3 Motor function at short-term follow-up

**Abbreviations:** BoNT=botulinum toxin; LFrTMS=low-frequency transcranial magnetic stimulation; iTBS= intermittent theta-burst stimulation; HFrTMS = high-frequency transcranial magnetic stimulation; cTBS=continuous theta-burst stimulation; atDCS=anodal transcranial direct current stimulation; ctDCS=cathodal transcranial direct current stimulation; dtDCS=dual transcranial direct current stimulation; NMES=neuromuscular electrical stimulation; TENS=transcutaneous electrical nerve stimulation; rPMS= repetitive peripheral magnetic stimulation.

## 19.4 Motor function at mid-term follow-up

**Abbreviations:** BoNT=botulinum toxin; LFrTMS=low-frequency transcranial magnetic stimulation; iTBS= intermittent theta-burst stimulation; HFrTMS = high-frequency transcranial magnetic stimulation; cTBS=continuous theta-burst stimulation; ctDCS=cathodal transcranial direct current stimulation; NMES=neuromuscular electrical stimulation; TENS=transcutaneous electrical nerve stimulation.

## 19.5 Acceptability

**Abbreviations:** BoNT=botulinum toxin; LFrTMS=low-frequency transcranial magnetic stimulation; iTBS= intermittent theta-burst stimulation; HFrTMS = high-frequency transcranial magnetic stimulation; atDCS=anodal transcranial direct current stimulation; ctDCS=cathodal transcranial direct current stimulation; dtDCS=dual transcranial direct current stimulation; NMES=neuromuscular electrical stimulation; TENS=transcutaneous electrical nerve stimulation.

# Appendix 20. Grading for certainty of evidence (CINeMA assessment).

## 20.1 Spasticity at short-term follow-up


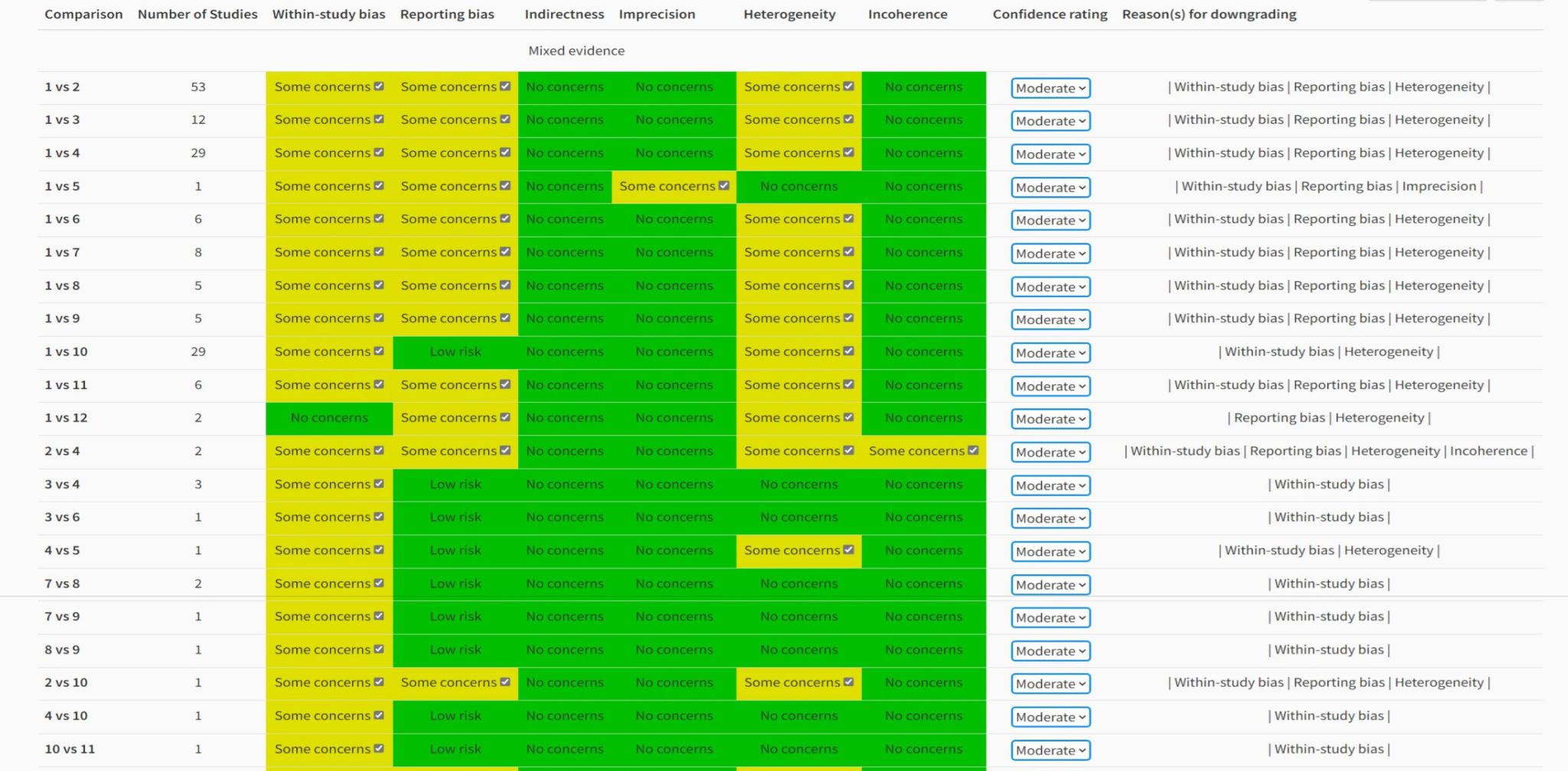


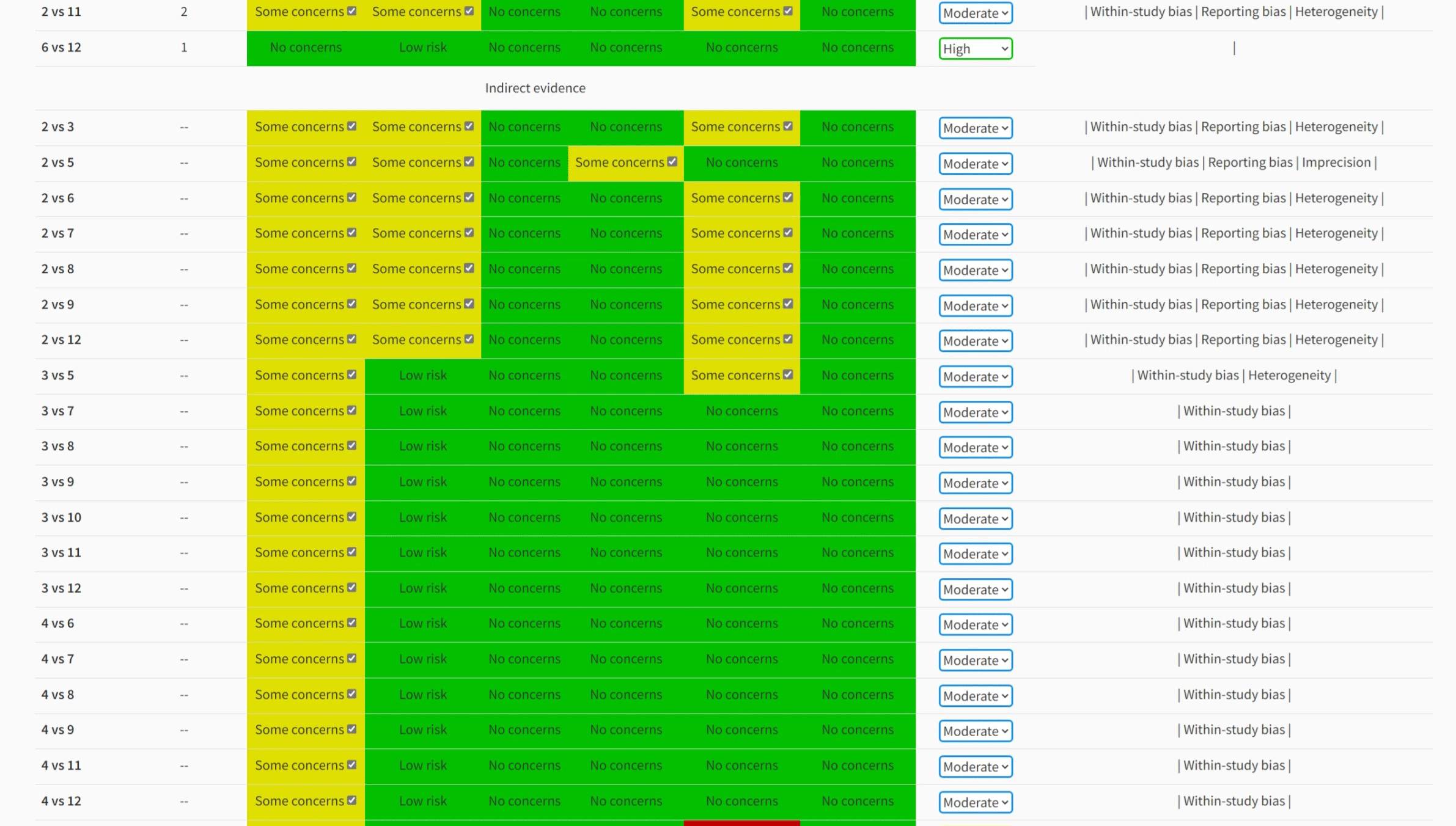


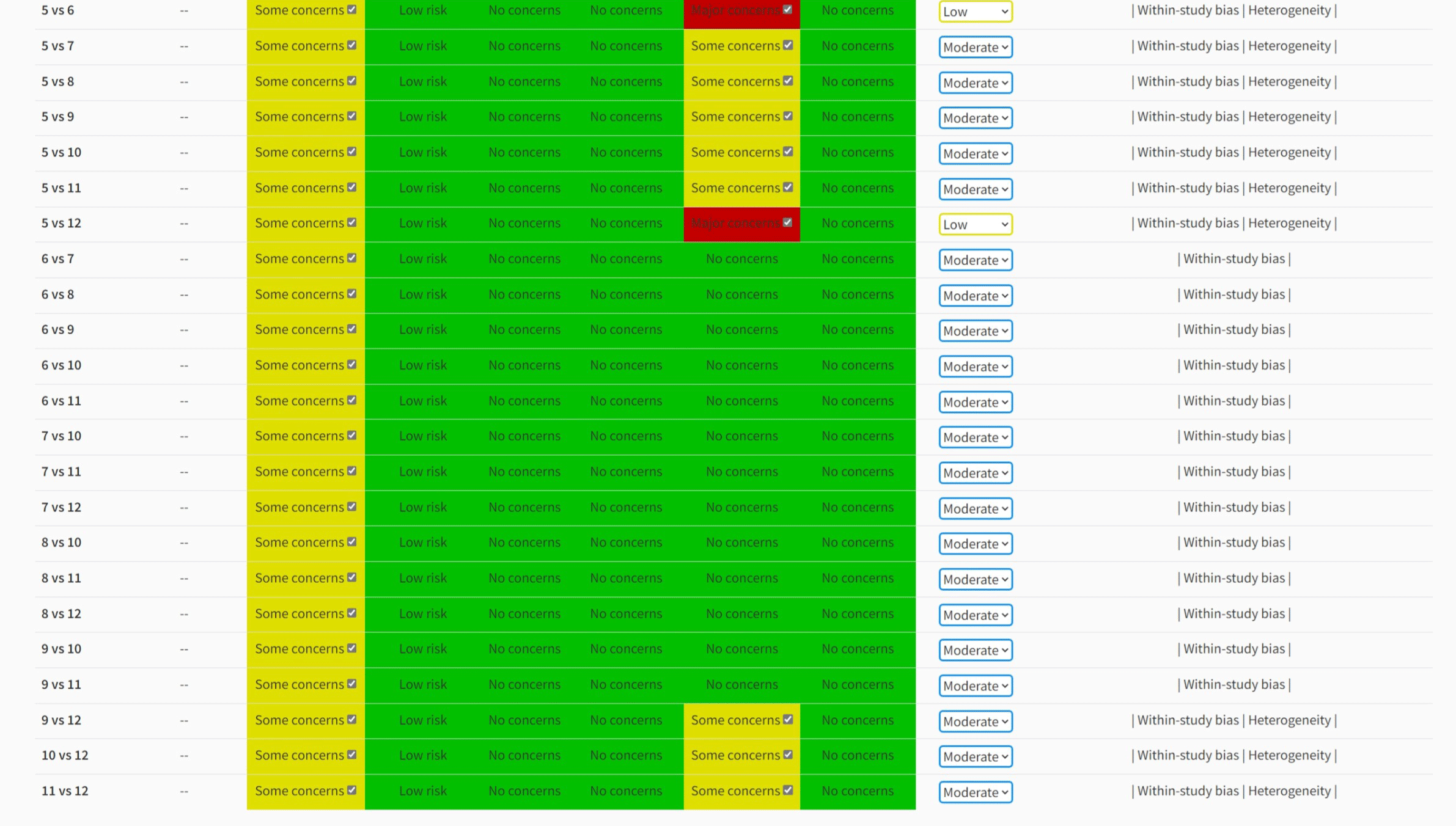


Note: 1=Control; 2=botulinum toxin; 3=high-frequency transcranial magnetic stimulation; 4=low-frequency transcranial magnetic stimulation; 5=intermittent theta-burst stimulation; 6=continuous theta-burst stimulation; 7=anodal transcranial direct current stimulation; 8=cathodal transcranial direct current stimulation; 9=dual transcranial direct current stimulation; 10=neuromuscular electrical stimulation; 11=transcutaneous electrical nerve stimulation; 12=repetitive peripheral magnetic stimulation.

## 20.2 Spasticity at mid-term follow-up

**
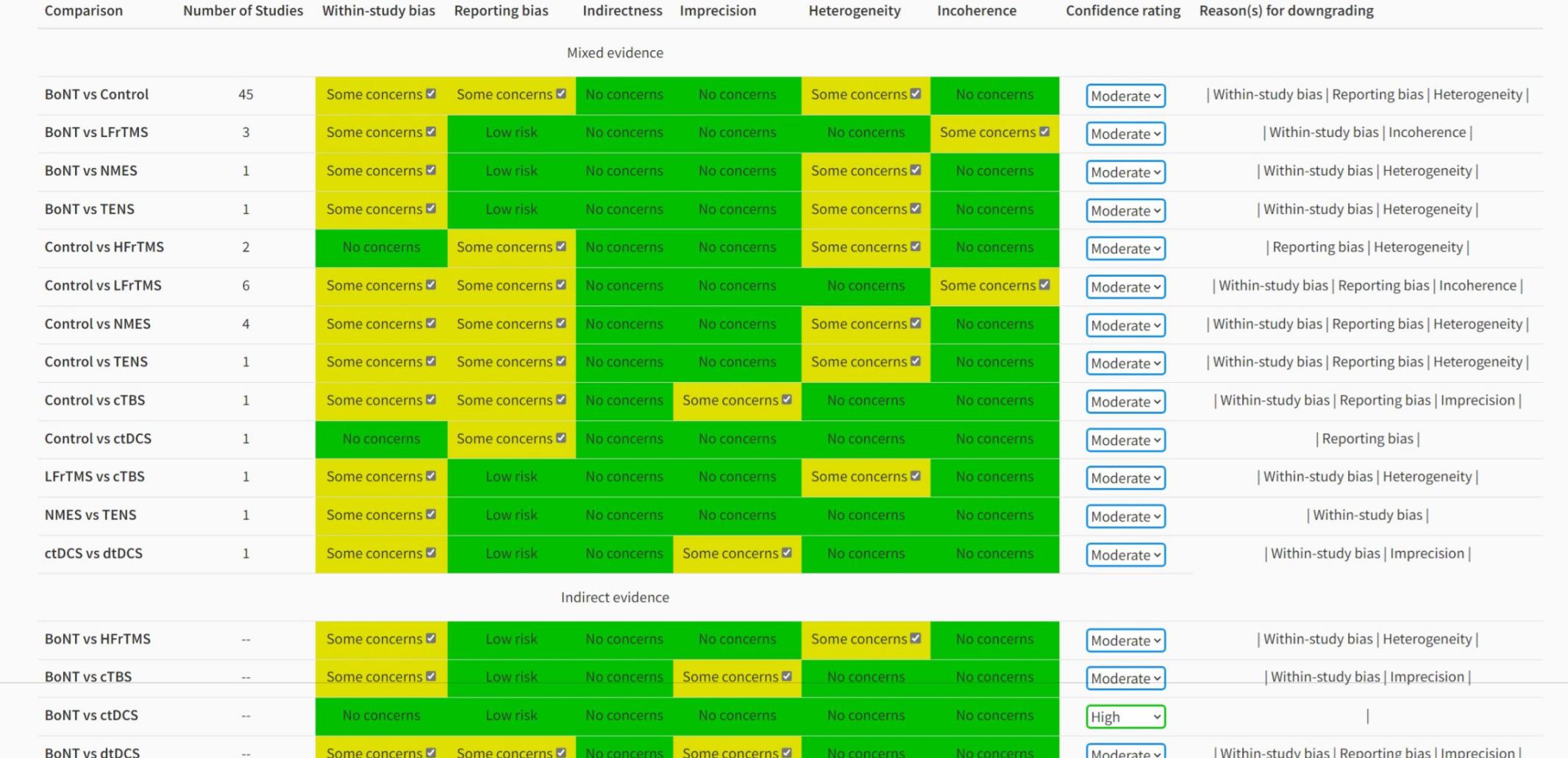
**

**
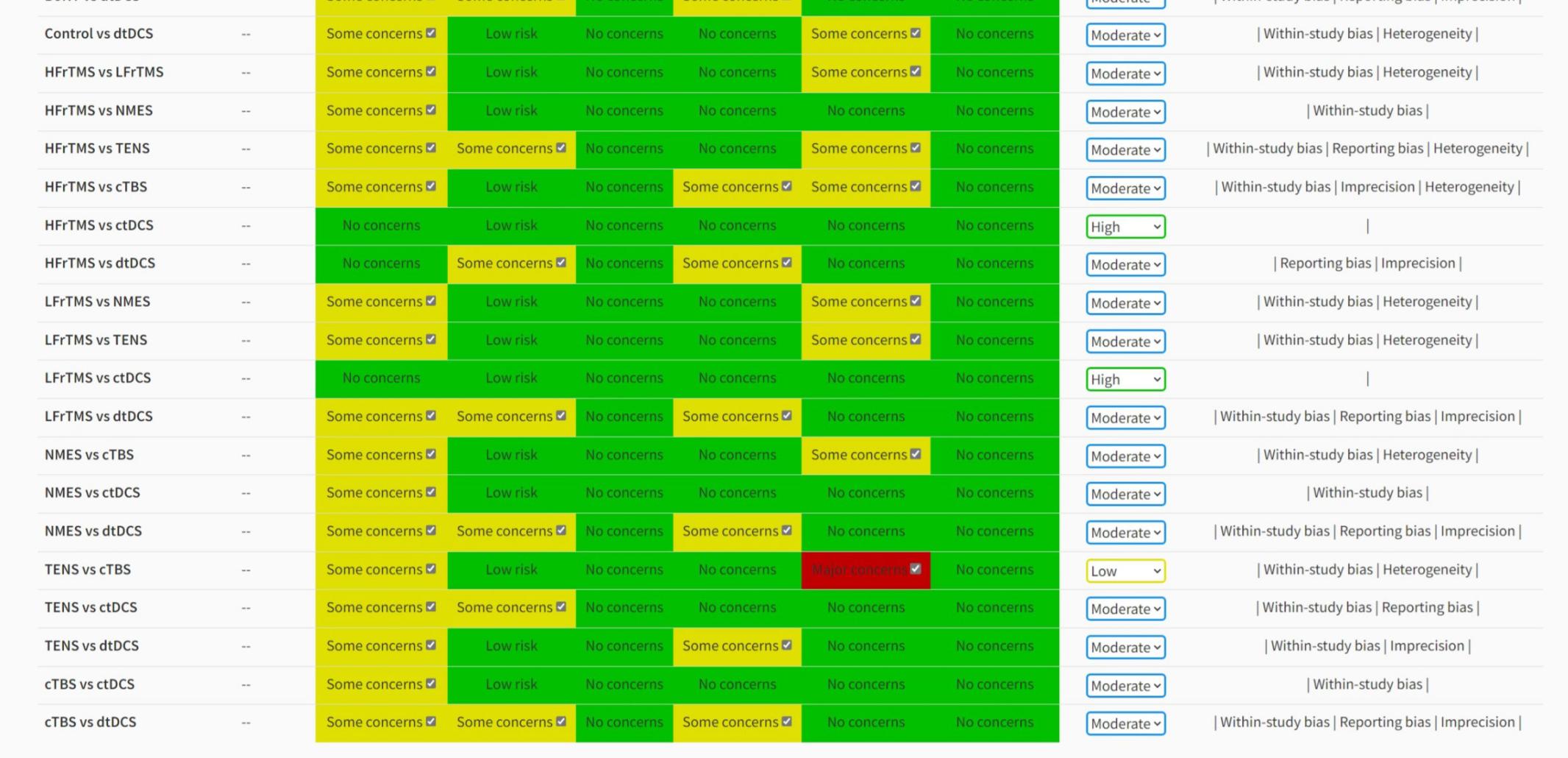
**

**Abbreviations:** BoNT=botulinum toxin; LFrTMS=low-frequency transcranial magnetic stimulation; iTBS= intermittent theta-burst stimulation; HFrTMS = high-frequency transcranial magnetic stimulation; atDCS=anodal transcranial direct current stimulation; ctDCS=cathodal transcranial direct current stimulation; dtDCS=dual transcranial direct current stimulation; NMES=neuromuscular electrical stimulation; TENS=transcutaneous electrical nerve stimulation.

## 20.3 Motor function at short-term follow-up


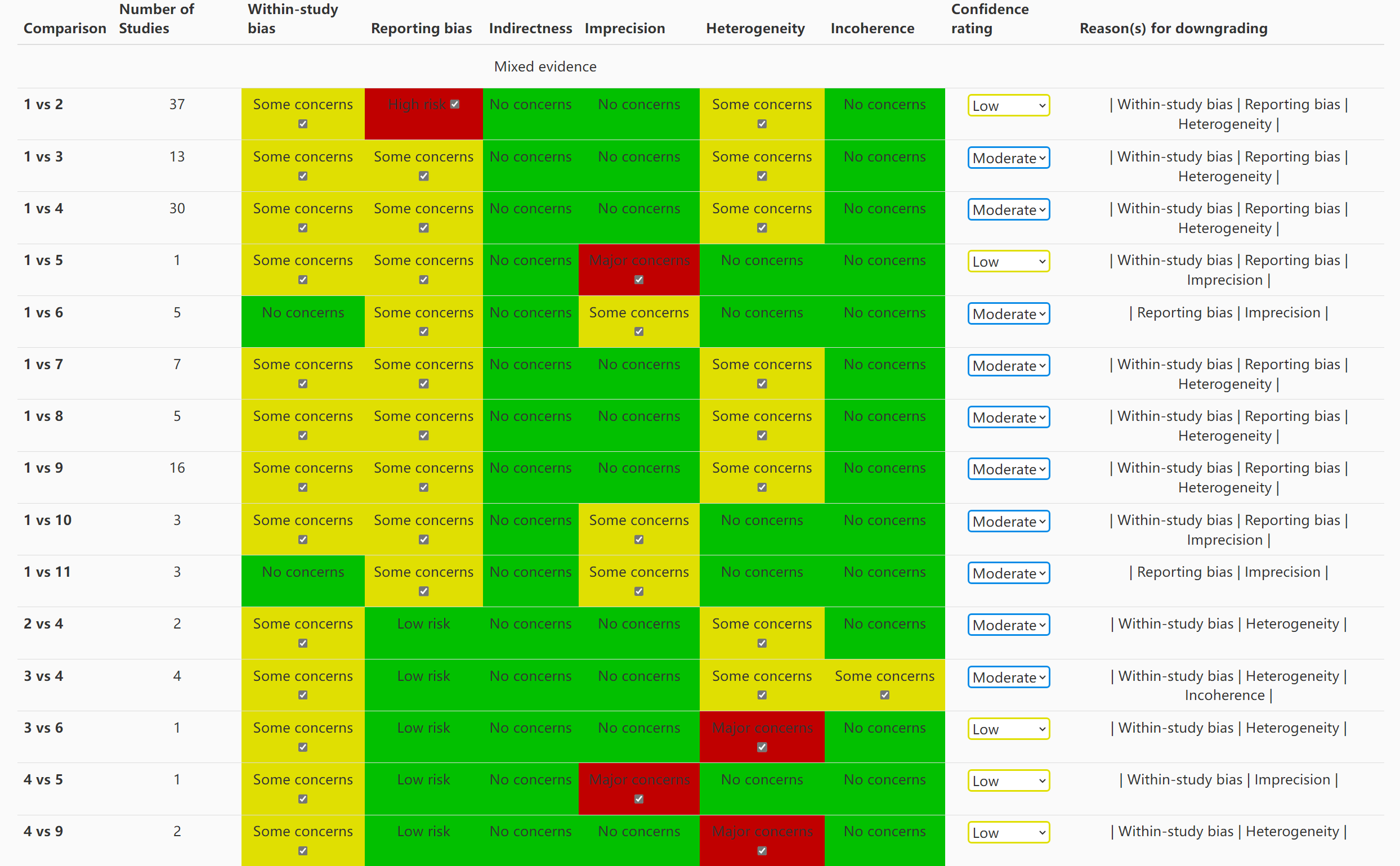


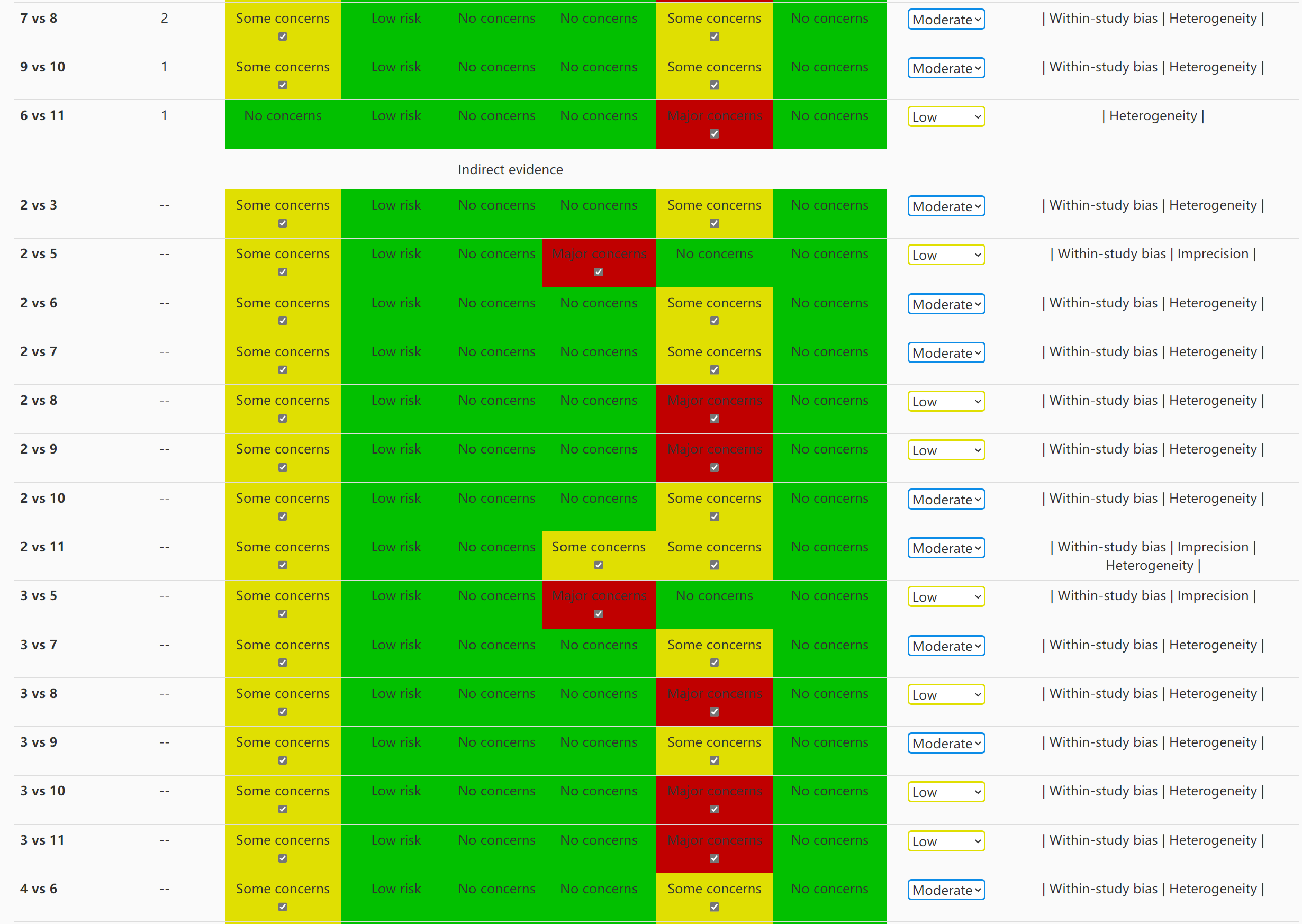


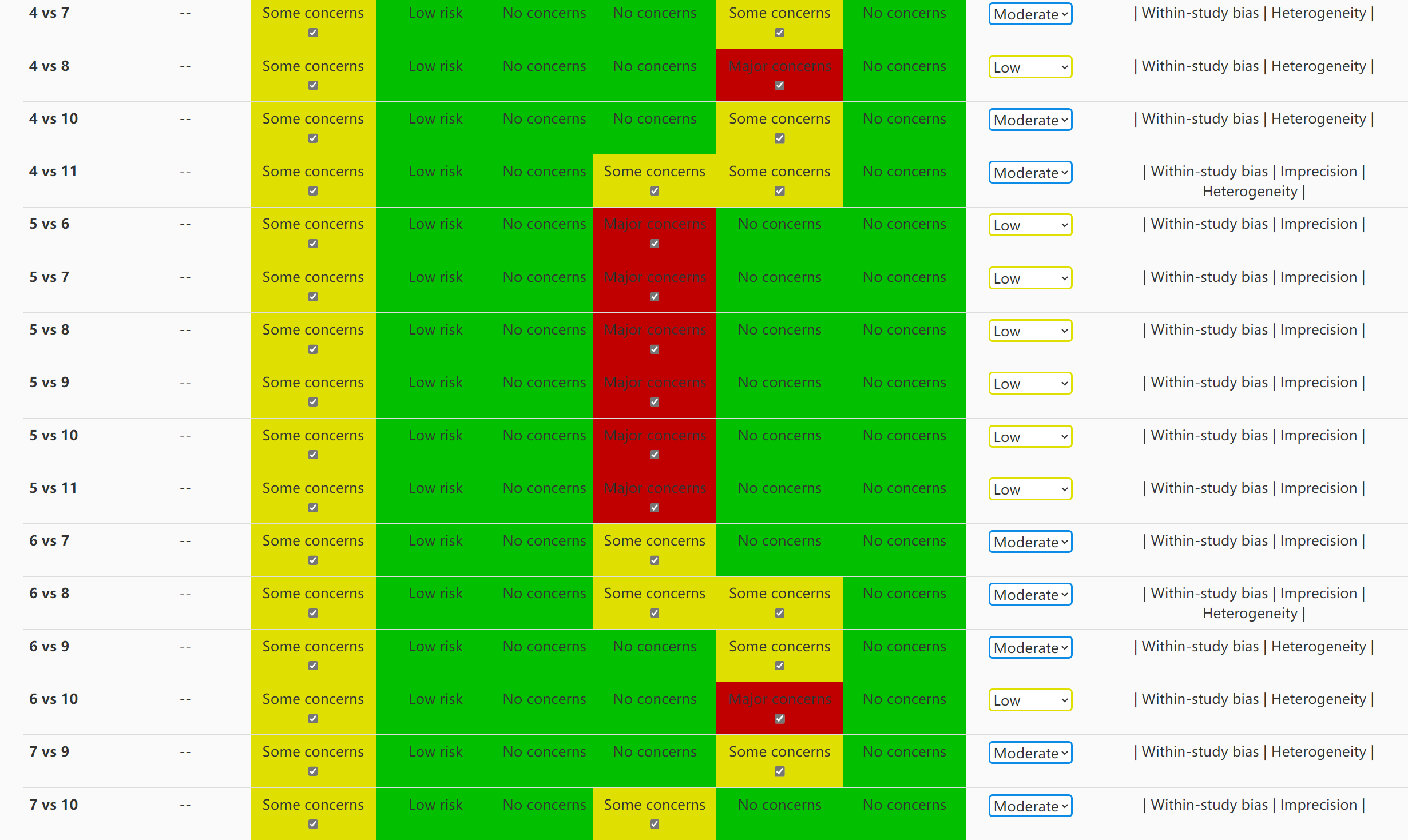


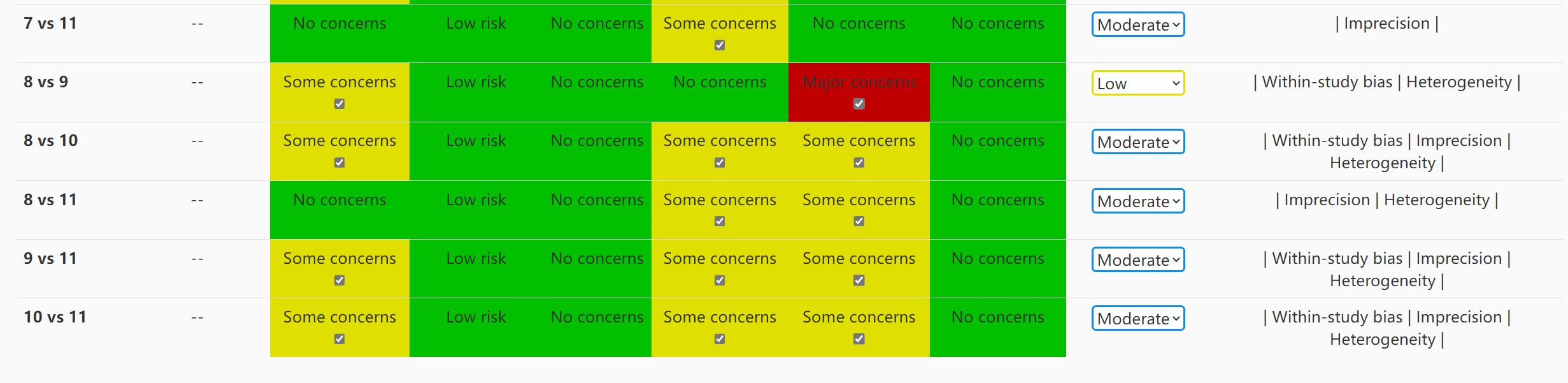


Note: 1=Control; 2=botulinum toxin; 3=high-frequency transcranial magnetic stimulation; 4=low-frequency transcranial magnetic stimulation; 5=intermittent theta-burst stimulation; 6=continuous theta-burst stimulation; 7=anodal transcranial direct current stimulation; 8=cathodal transcranial direct current stimulation; 9=neuromuscular electrical stimulation; 10=transcutaneous electrical nerve stimulation; 11=repetitive peripheral magnetic stimulation.

## 20.4 Motor function at mid-term follow-up


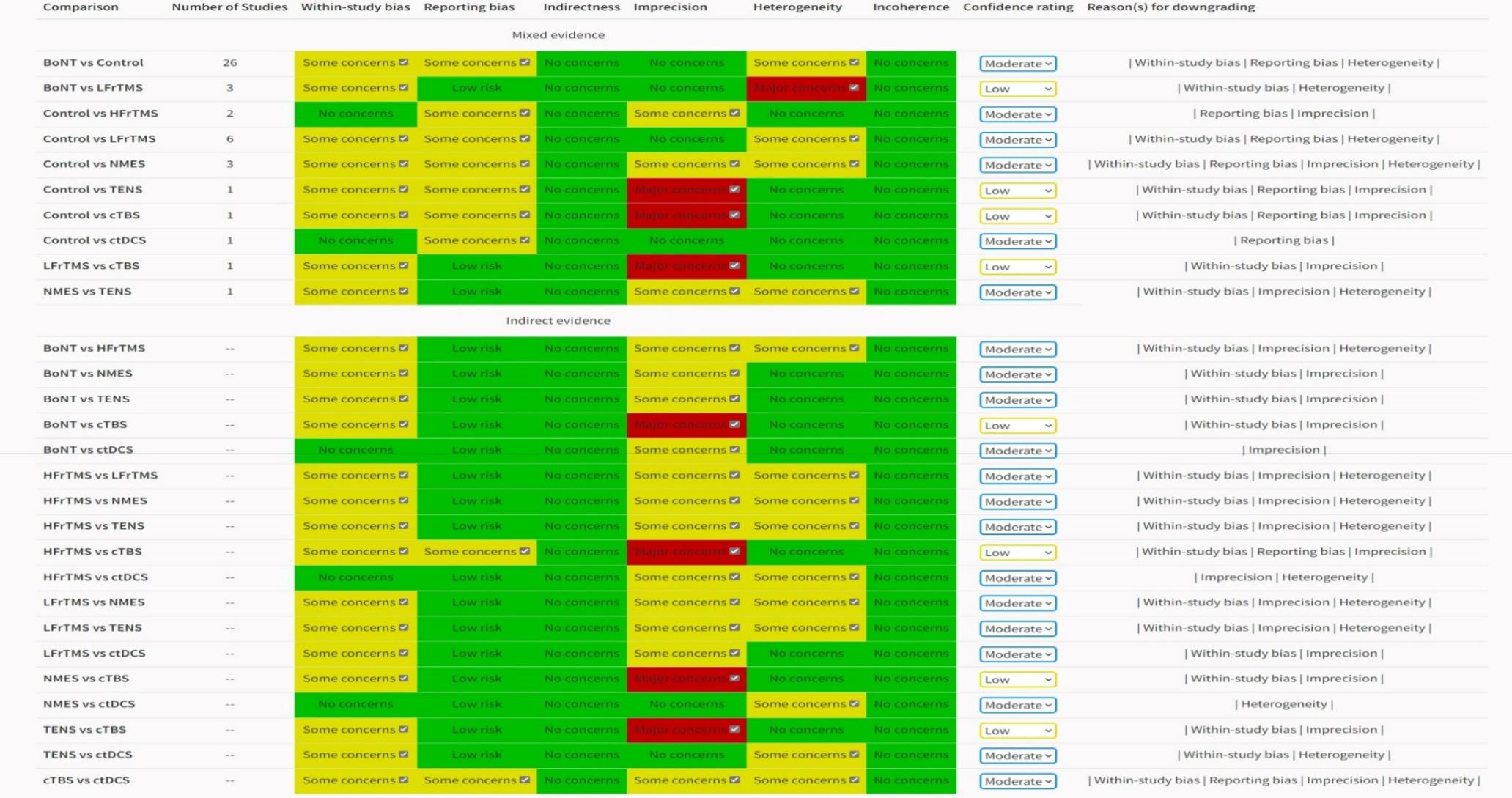


**Abbreviations:** BoNT=botulinum toxin LFrTMS=low-frequency transcranial magnetic stimulation; HFrTMS = high-frequency transcranial magnetic stimulation; ctDCS=cathodal transcranial direct current stimulation; NMES=neuromuscular electrical stimulation; TENS=transcutaneous electrical nerve stimulation.

## 20.5 Acceptability


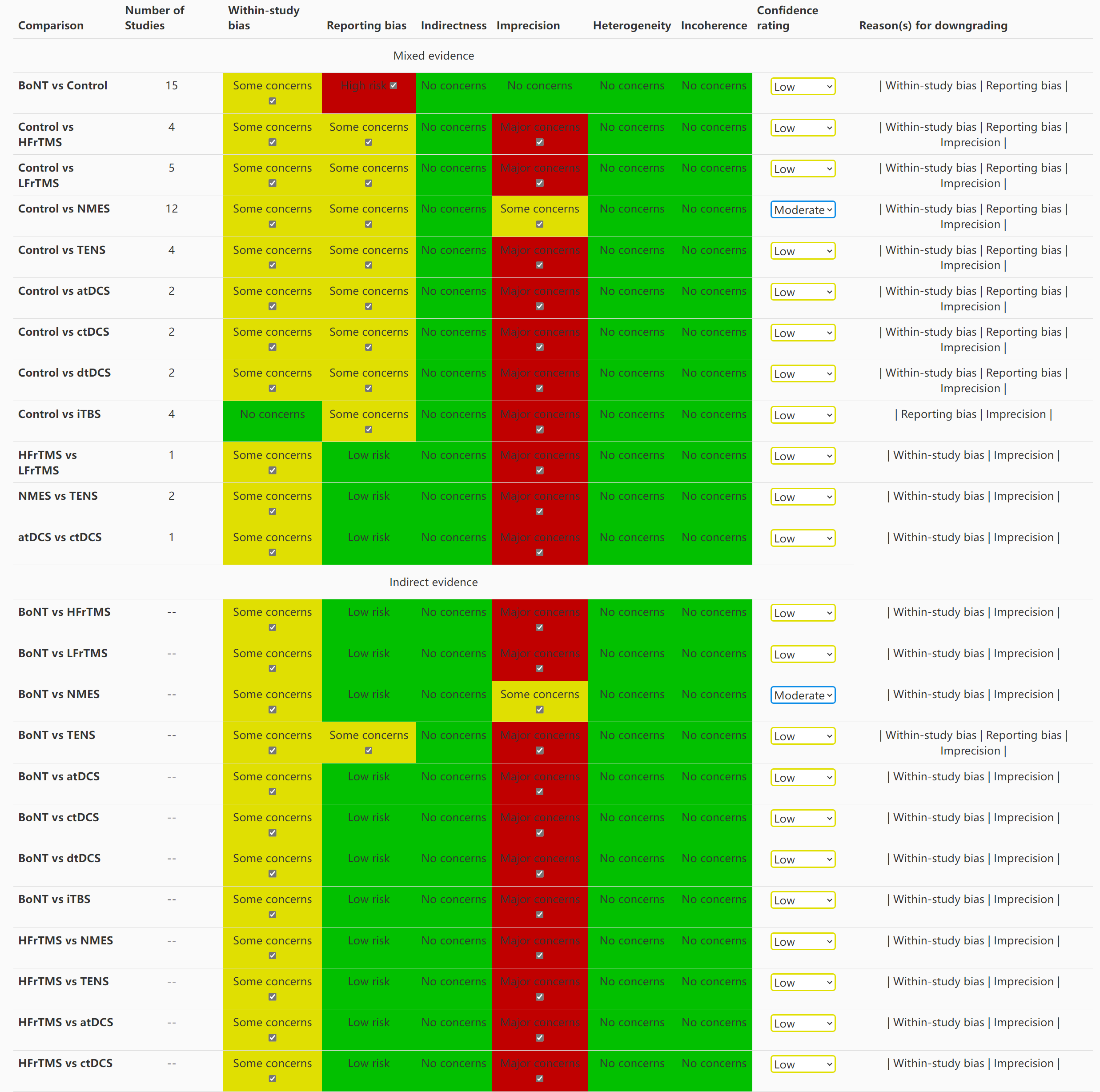


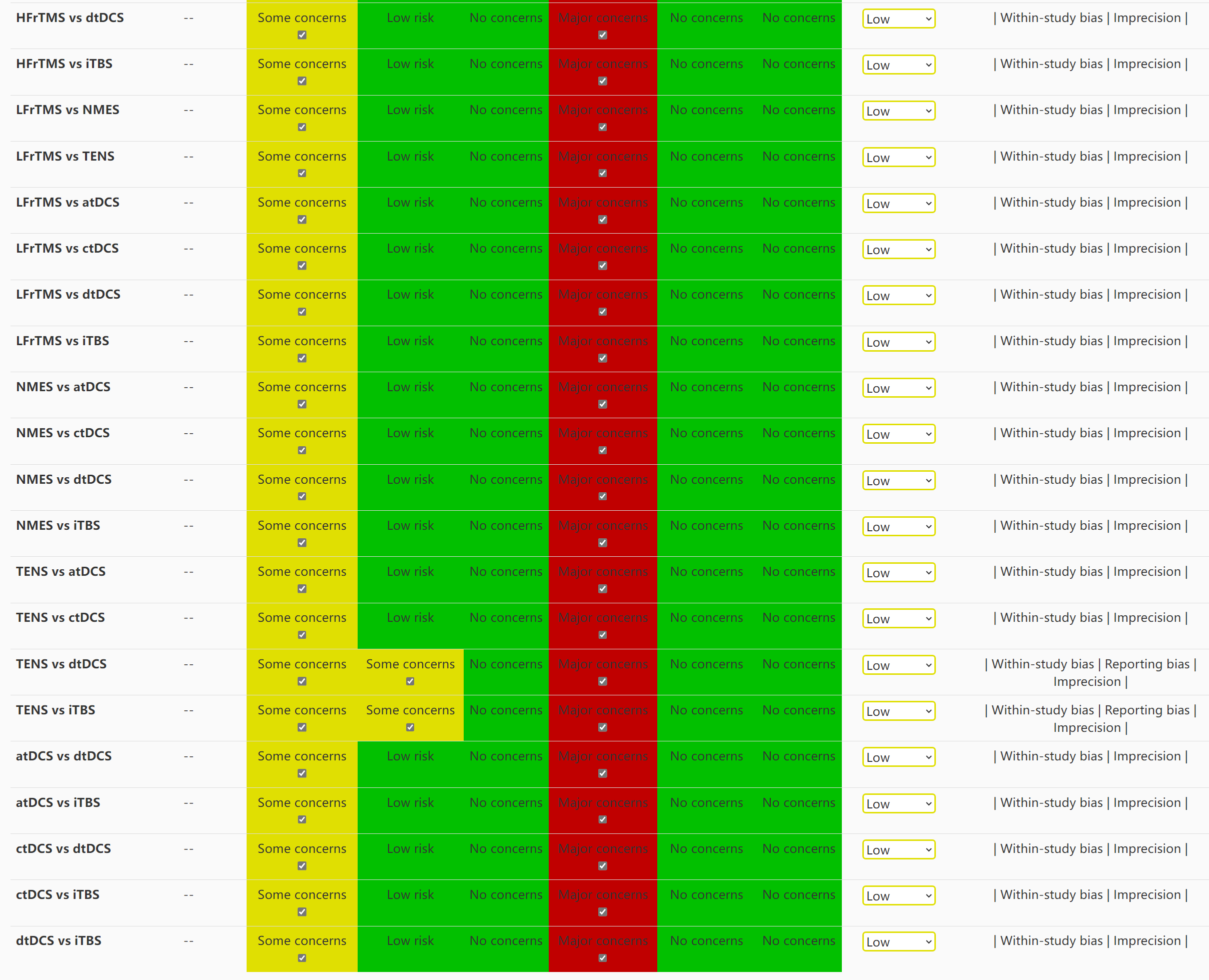


BoNT=botulinum toxin; LFrTMS=low-frequency transcranial magnetic stimulation; iTBS= intermittent theta-burst stimulation; HFrTMS = high-frequency transcranial magnetic stimulation; iTBS= intermittent theta-burst stimulation; atDCS=anodal transcranial direct current stimulation; ctDCS=cathodal transcranial direct current stimulation; NMES=neuromuscular electrical stimulation; TENS=transcutaneous electrical nerve stimulation.
